# Supplementary material for: Antibody escape and global spread of SARS-CoV-2 lineage A.27
Source: Nat Commun. 2022 Mar 3;13:1152. doi: 10.1038/s41467-022-28766-y (PMC8894356; doi:10.1038/s41467-022-28766-y)
Supplement: Supplementary file 1 — Supplementary Information [file 41467_2022_28766_MOESM1_ESM.pdf]

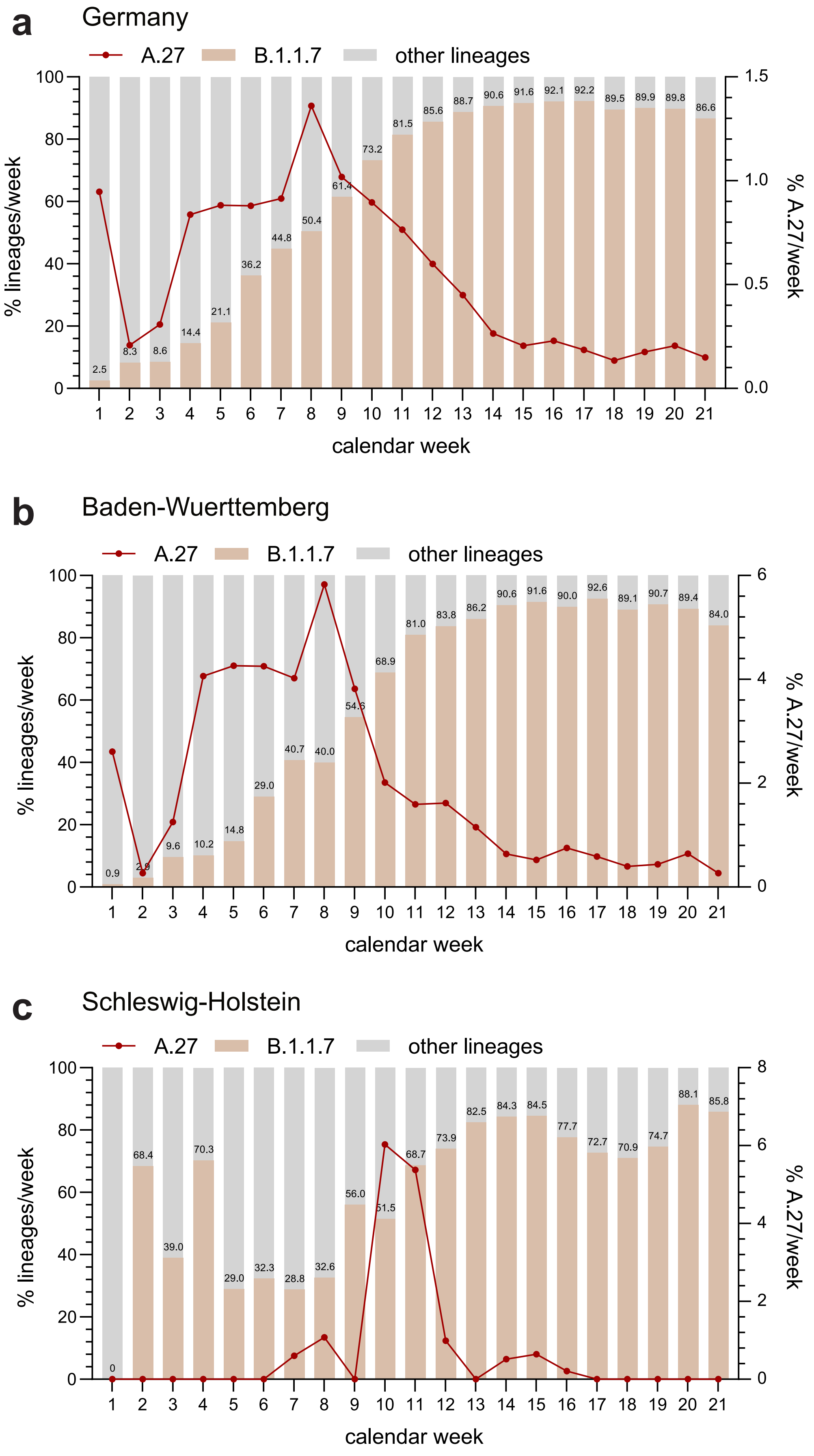

Supplementary Fig. 1 Decrease in sequenced A.27 patient samples in comparison to the increase of B.1.1.7 sequences. Frequencies of detected sequences belonging to the A.27, B.1.1.7 or other lineages between calendar week 1 and 21 of 2021 for (a) Germany, (b) Baden-Wuerttemberg and (c) Schleswig-Holstein based on all available data from the RKI.

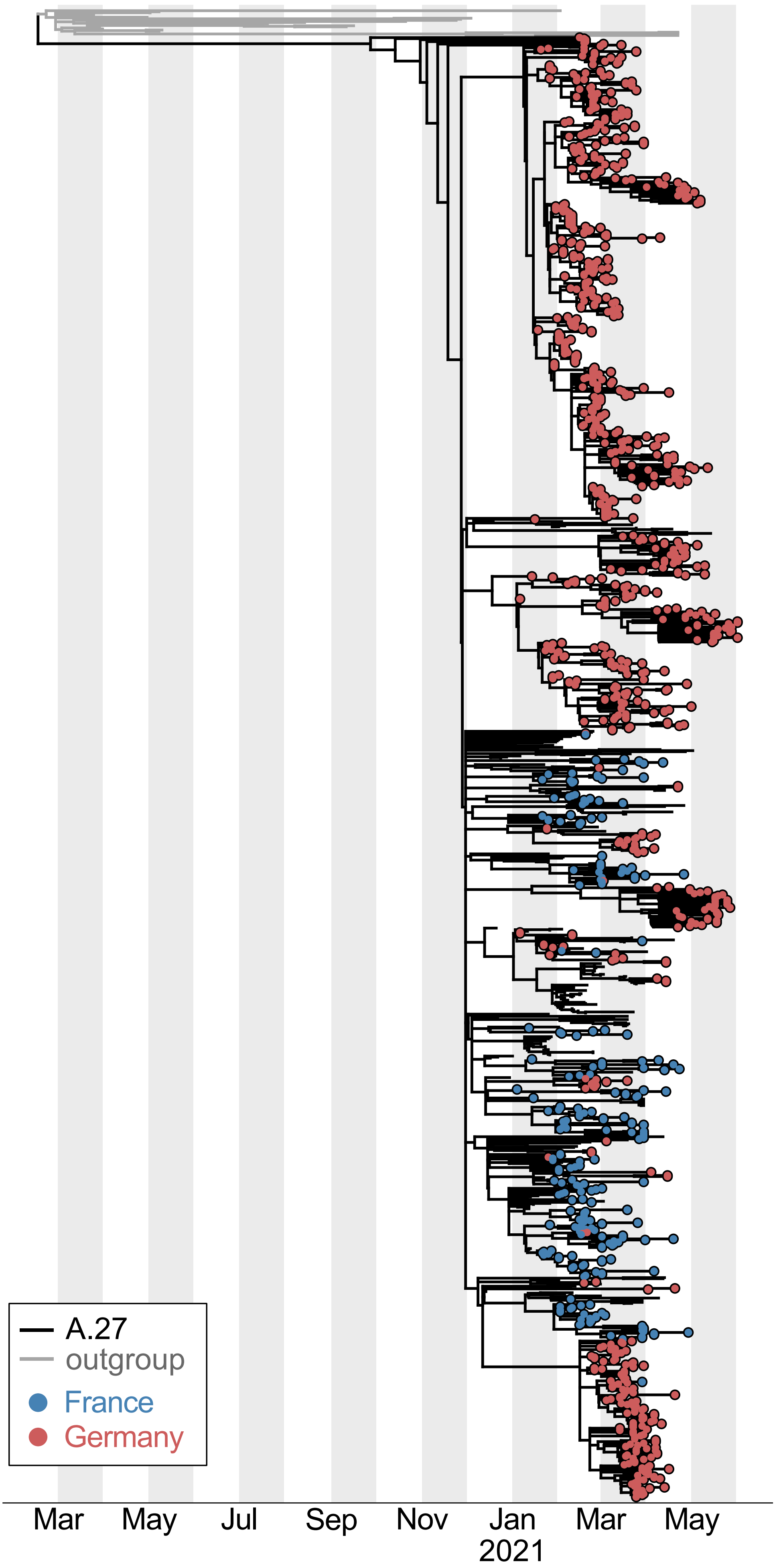

Supplementary Fig. 2 Maximum-likelihood phylogeny of the A.27 lineage. Time-calibrated phylogeny on the entire dataset of 1,383 sequences, with French and German nodes coloured, and an outgroup consisting of 25 non-A27 ancestral sequences, coloured in grey.

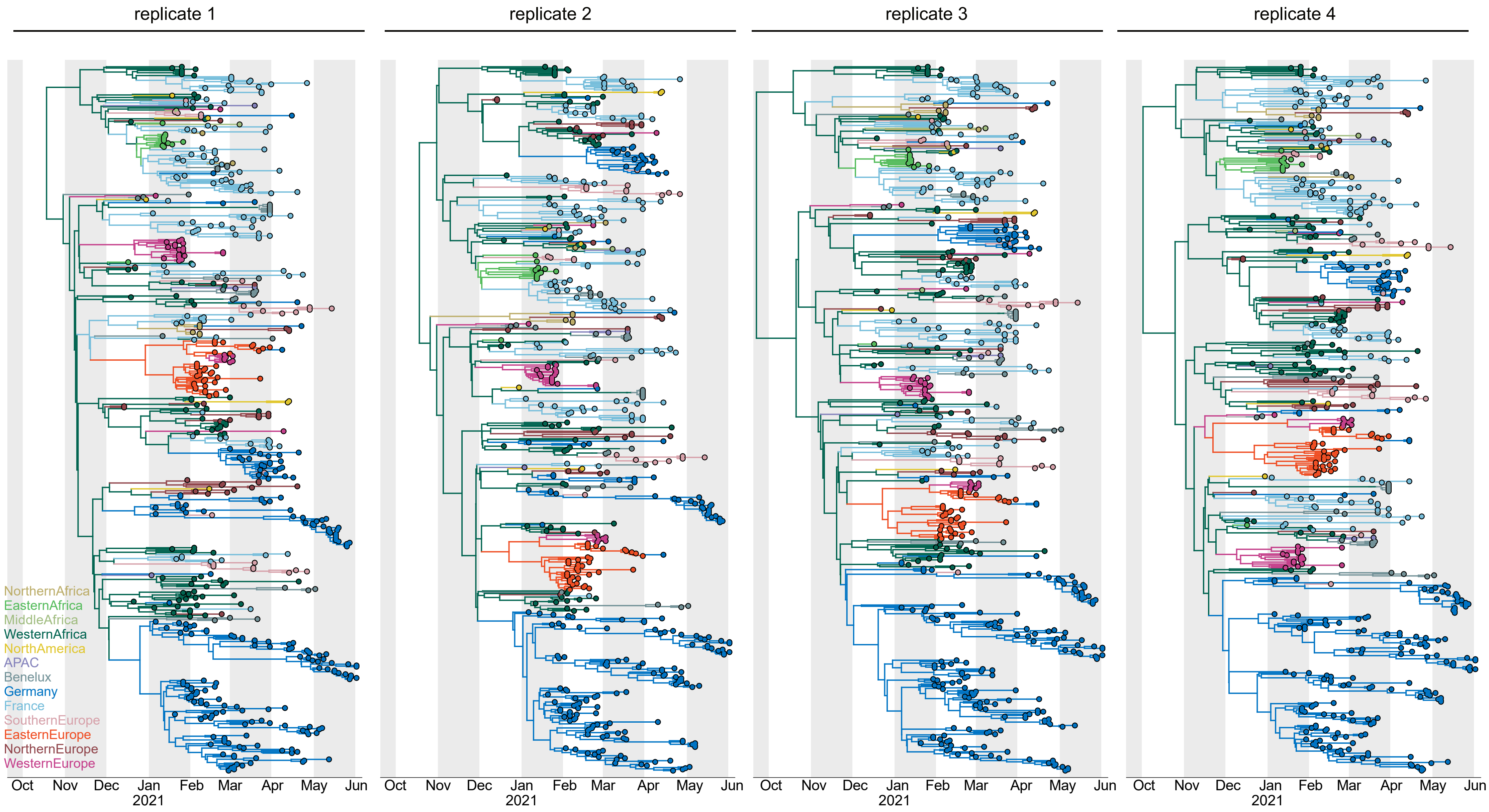

Supplementary Fig. 3 Region-annotated phylogenies for four additional replicate phylogeographic analyses. Four additional replicates of the analysis shown in Fig. 2 (c) confirm an inferred origin of the A.27 lineage in Western Africa with subsequent spread to most of the other regions. Smaller white circles represent posterior support >0.5, whereas bigger black circles represent posterior support >0.95. Colours correspond to those in Fig. 2 (c).

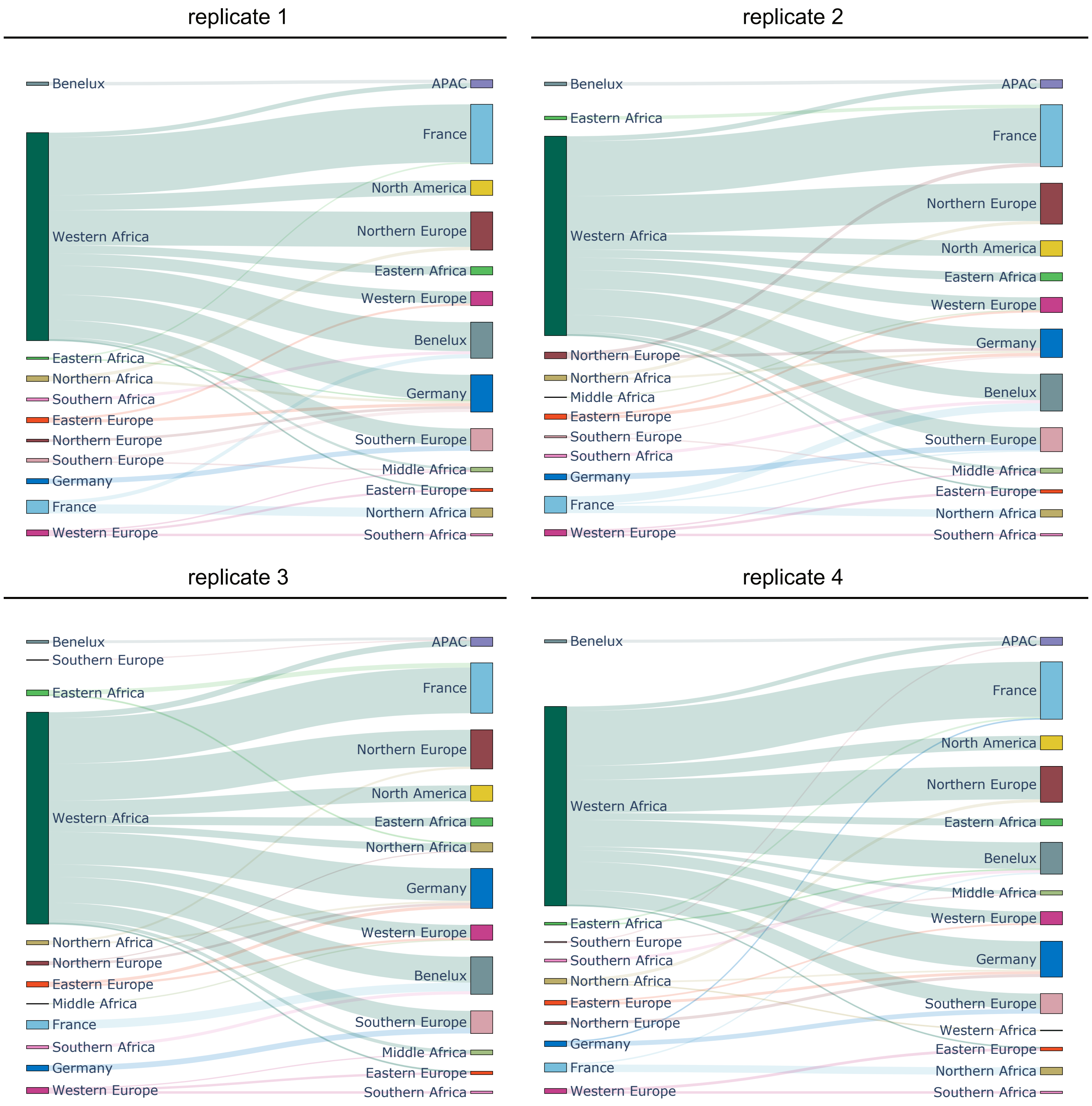

Supplementary Fig. 4 Sankey plots for four additional replicate phylogeographic analyses. In order to test the effects of possible sampling bias, we performed the travel history-aware phylogeographic analysis an additional four times, using a different random sampling selection each time. Transitions are shown from the regions on the left into the regions on the right. Line thickness corresponds to the number of estimated Markov jumps between these regions. As for Fig. 2 (b), only transitions with a Bayes Factor above 3 are shown, pointing to strong support for an inferred origin of the A.27 lineage in Western Africa across all replicates. Colours correspond to those in Fig. 2.

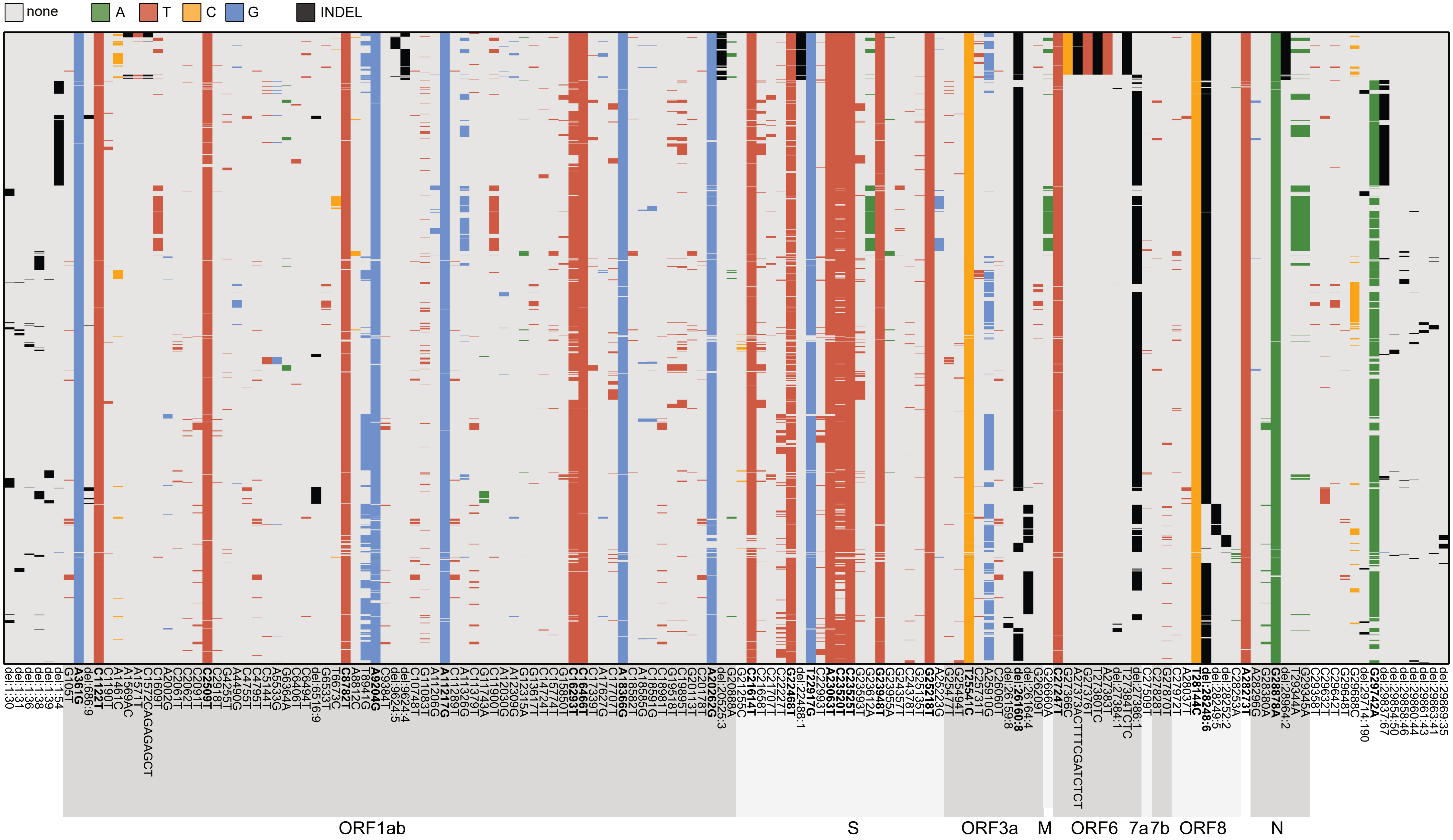

Supplementary Fig. 5 Nucleotide substitutions found in more than 1% of A.27 sequences. Nucleotide genome profiles of all A.27 sequences were defined in comparison to Wuhan-Hu-1 (NC_045512.2) using covSonar (https://gitlab.com/s.fuchs/covsonar). All mutations exceeding a total frequency of 1 % were visualized using the R pheatmap package. Mutations exceeding a frequency of 75 % are indicated in bold font.

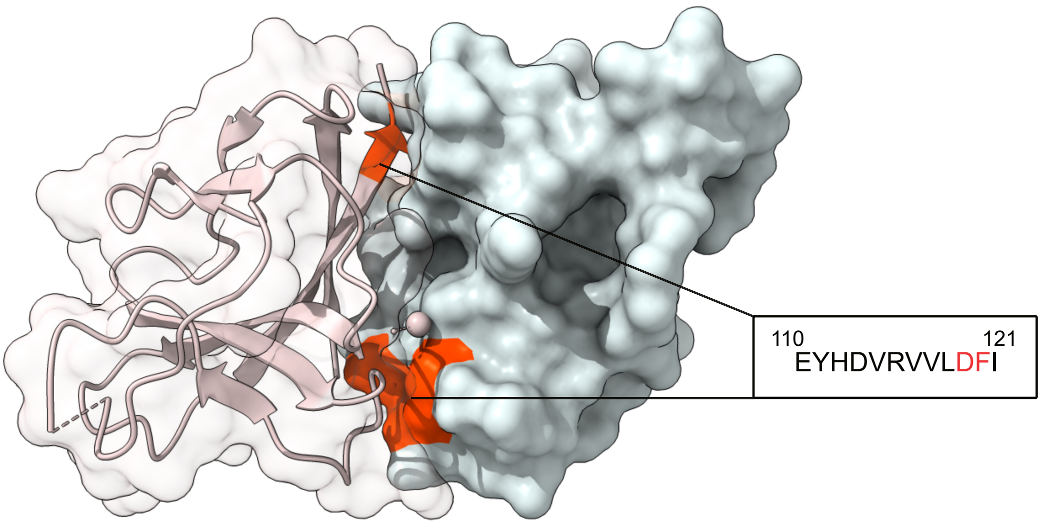

Supplementary Fig. 6 3D structure of homodimeric ORF8. ORF8 homodimer of SARS-CoV-2 (PDB accession number: 7jtl) with one monomer shown in surface presentation (grey) and the other monomer shown in transparent surface/cartoon presentation. The region of the 119/120 deletion is marked in red.

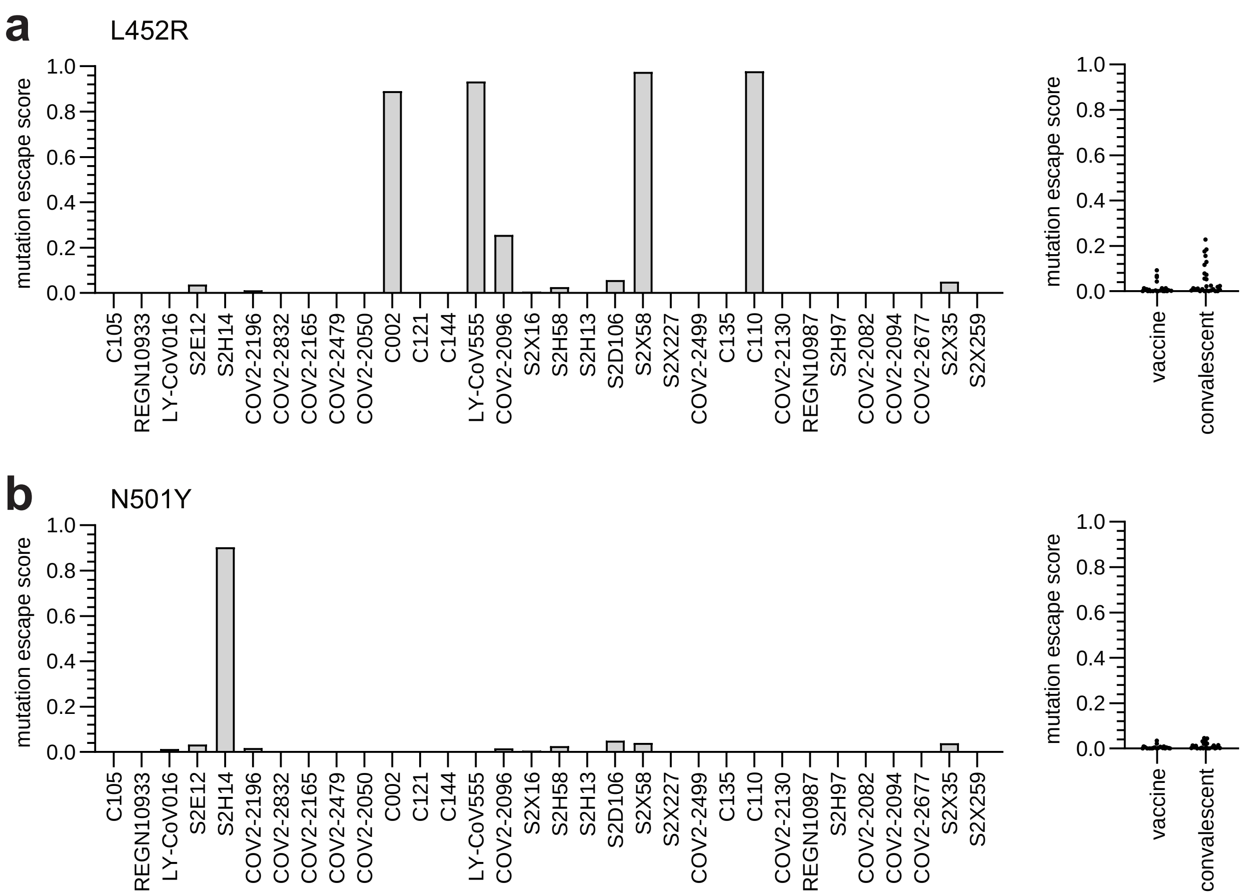

Supplementary Fig. 7 Binding data of different monoclonal antibodies and polyclonal sera with the RBD spike mutants L452R and N501Y. Binding data between SARS-CoV-2 RBD mutants and different monoclonal antibodies generated by the Bloom lab (https://github.com/jbloomlab/SARS2_RBD_Ab_escape_maps) were subset for the L452R and N501Y mutation. Shown are the mutation escape scores.

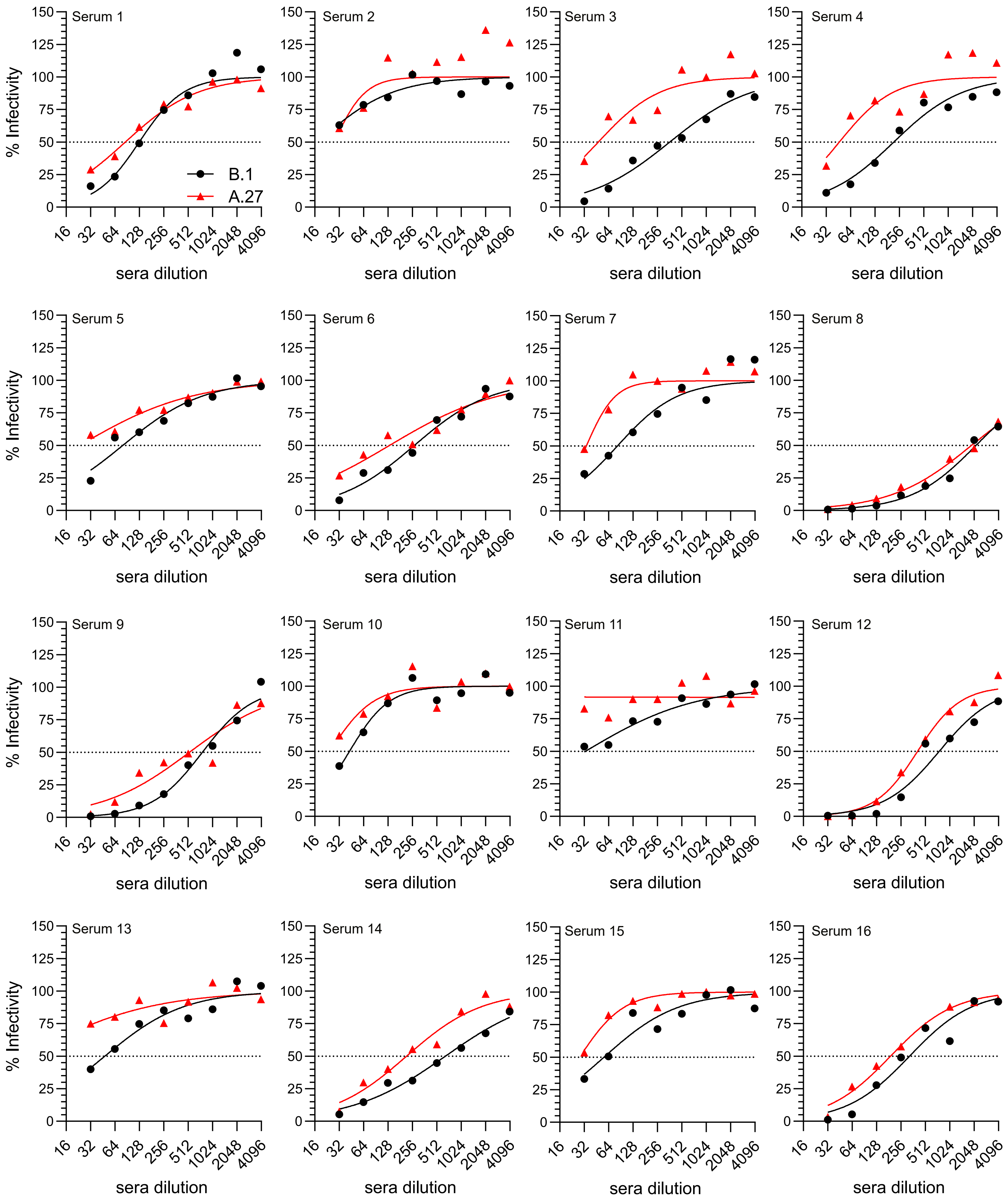

Supplementary Fig. 8 Plaque reduction assays of convalescent sera. Neutralizing activity of convalescent sera against B.1 (grey) or A.27 (red). 100 pfu of each virus was incubated with serial 2-fold sera dilutions and analysed by plaque assay in biological duplicates. Shown are the mean of the individual dilutions and the respective curve fits.

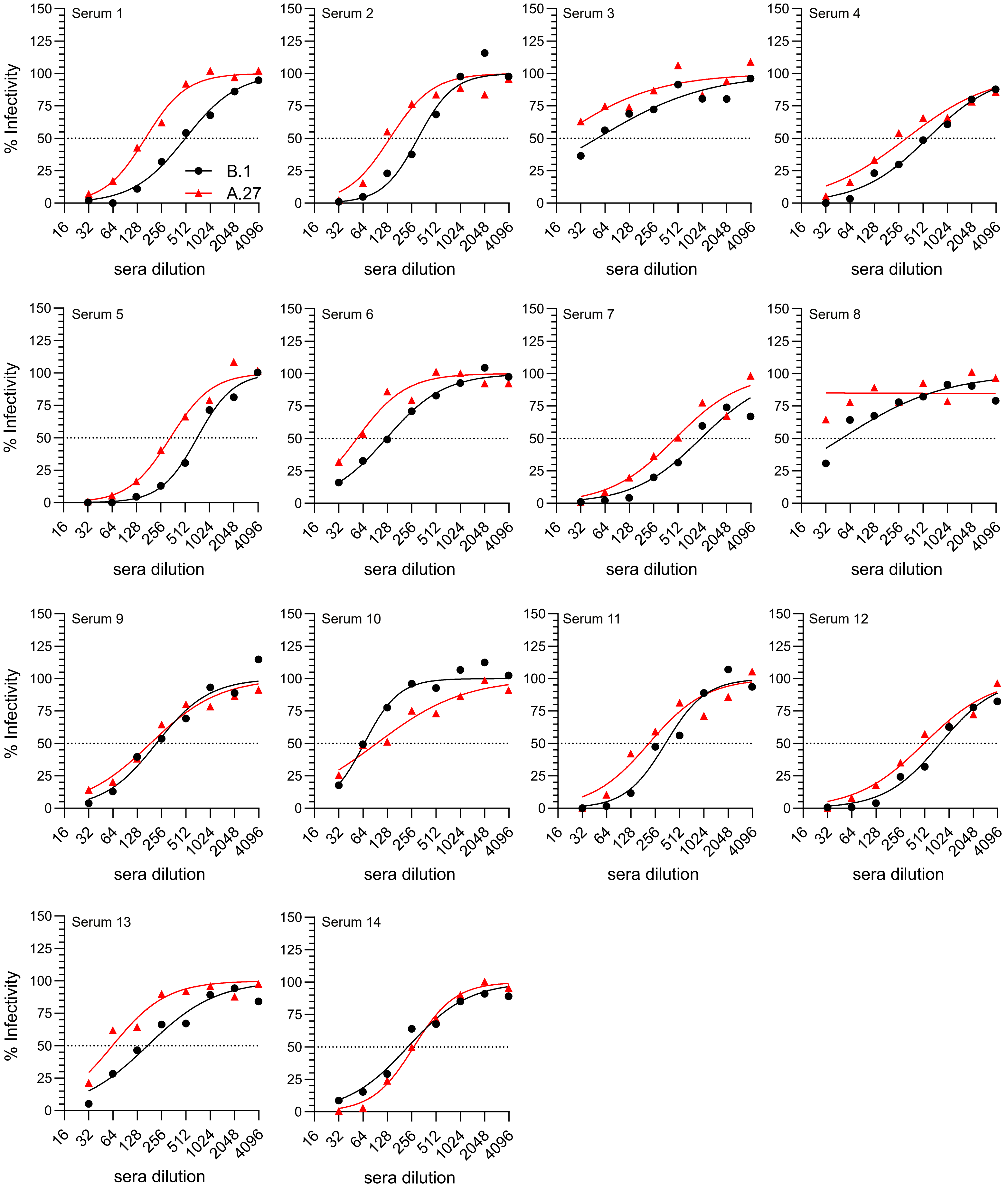

Supplementary Fig. 9 Plaque reduction assays of sera from BioNTech BNT162b2 vaccinees. Neutralizing activity of sera from BioNTech BNT162b2 vaccinees against B.1 (grey) or A.27 (red). 100 pfu of each virus was incubated with serial 2-fold sera dilutions and analysed by plaque assay in biological duplicates. Shown are the mean of the individual dilutions and the respective curve fits.

Supplementary Table 1 Individual travel histories collected for the core A.27 genomic data set analysed in this study. These documented travel cases, all from Western and Southern Africa into Europe, were retrieved from the labs that submitted the corresponding genomes to GISAID.

Sequence name	Accession ID	Sampling date	Sampling location	Travel origin
hCoV-19/Belgium/rega-6126/2021|EPI_ISL_2404564|2021-03-30|Benelux	EPI_ISL_2404564	Belgium (Benelux)	2021-03-30	Mali (Western Africa)
hCoV-19/Belgium/rega-6127/2021|EPI_ISL_2404565|2021-03-30|Benelux	EPI_ISL_2404565	Belgium (Benelux)	2021-03-30	Mali (Western Africa)
hCoV-19/Belgium/rega-6128/2021|EPI_ISL_2404566|2021-03-30|Benelux	EPI_ISL_2404566	Belgium (Benelux)	2021-03-30	Mali (Western Africa)
hCoV-19/Belgium/rega-6129/2021|EPI_ISL_2404567|2021-03-30|Benelux	EPI_ISL_2404567	Belgium (Benelux)	2021-03-30	Mali (Western Africa)
hCoV-19/Belgium/rega-6130/2021|EPI_ISL_2404568|2021-03-30|Benelux	EPI_ISL_2404568	Belgium (Benelux)	2021-03-30	Mali (Western Africa)
hCoV-19/Belgium/rega-6131/2021|EPI_ISL_2404569|2021-03-30|Benelux	EPI_ISL_2404569	Belgium (Benelux)	2021-03-30	Mali (Western Africa)
hCoV-19/Belgium/rega-6132/2021|EPI_ISL_2404570|2021-03-30|Benelux	EPI_ISL_2404570	Belgium (Benelux)	2021-03-30	Mali (Western Africa)
hCoV-19/Belgium/rega-5023/2021|EPI_ISL_1382686|2021-03-03|Benelux	EPI_ISL_1382686	Belgium (Benelux)	2021-03-3	Burkina Faso (Western Africa)
hCoV-19/Belgium/rega-5443/2021|EPI_ISL_2403956|2021-03-22|Benelux	EPI_ISL_2403956	Belgium (Benelux)	2021-03-22	Burkina Faso (Western Africa)
hCoV-19/Belgium/rega-5484/2021|EPI_ISL_2403997|2021-03-05|Benelux	EPI_ISL_2403997	Belgium (Benelux)	2021-03-5	Burkina Faso (Western Africa)
hCoV-19/Netherlands/ZH-EMC-1312/2020|EPI_ISL_801441|2020-12-24|Benelux	EPI_ISL_801441	Netherlands (Benelux)	2020-12-24	South Africa (Southern Africa)
hCoV-19/Netherlands/ZH-EMC-1313/2020|EPI_ISL_801442|2020-12-28|Benelux	EPI_ISL_801442	Netherlands (Benelux)	2020-12-28	South Africa (Southern Africa)

Supplementary Table 2 GISAID accession numbers. We gratefully acknowledge the following authors from the originating laboratories responsible for obtaining the specimens, as well as the submitting laboratories where the genome data were generated and shared via GISAID, on which this research is based. All submitters of data may be contacted directly via www.gisaid.org. Authors are sorted alphabetically.

Accession ID	Originating Laboratory	Submitting Laboratory	Authors
EPI_ISL_1167046, EPI_ISL_1167049, EPI_ISL_1167053	"Dr. Andrija Stampar" Teaching Institute of Public Health, Department of Clinical Microbiology	Istituto di Genomica Applicata	Davide Scaglione; Eleonora Paparelli; Fedrica Cattonaro; Gabriele Magris; Irena Jurman; Jasmina Vranes; Michele Morgante; Slobodanka Radovic; Vera Vendramin
EPI_ISL_996004	"Dr. Andrija Stampar" Teaching Institute of Public Health, Department of Clinical Microbiology	Institute of Applied Genomics	Federica Cattonaro; Jasmina Vranes; Michele Morgante
EPI_ISL_2438635	"National Center of Expertise" CSEC MH RK in Karaganda region	Reference laboratory for the control of viral infections	Aidar Usserbayev; Aknur Mutaliyeva; Andrey Komissarov; Artem Fadeev; Azamat Kenessov; Bekzhan Maikotov; Gaukhar Nussupbayeva; Madina Tleubergenova; Maria Pisareva; Nazym Tleumbetova
EPI_ISL_2438606	"National Center of Expertise" CSEC MH RK in Kyzylorda Region	Reference laboratory for the control of viral infections	Aidar Usserbayev; Aknur Mutaliyeva; Andrey Komissarov; Artem Fadeev; Azamat Kenessov; Bekzhan Maikotov; Gaukhar Nussupbayeva; Madina Tleubergenova; Maria Pisareva; Nazym Tleumbetova
EPI_ISL_635059, EPI_ISL_707698, EPI_ISL_707791, EPI_ISL_733500, EPI_ISL_763067, EPI_ISL_794738, EPI_ISL_1208403			
see above	1-Laboratory of Microbiology, National Reference Lab, Charles Nicolle Hospital; 2-University of Tunis ElManar, Faculty of Medicine of Tunis, LR99ES09, Tunis, Tunisia	1-Clinical and Experimental Pharmacology Lab, LR16SP02, National Center of Pharmacovigilance, University of Tunis El Manar, Tunis, Tunisia. 2-Neurodegenerative diseases and psychiatric troubles, LR18SP03, Razi Hospital, University of Tunis El Manar, Tunis, Tunisia. 3- Ministry of Health, National Observatory of New and Emerging Diseases, 1006, Tunis, Tunisia	Alia Ben Kahla; Alia BenKahla; Asma Ferjani; Awatef El MOussi; Gaies Emna; Guedi Ali Barreh; Habiba Ben Romdhane; Hafsia Ladhari; Hanen El Jebari; Houssem Guedouar; Ilhem Boutiba-Ben Boubaker; Ilhem Boutiba-Ben Boubaker.; Imen Kacem; Imen Mkada; Ines Mdini; Jalila Ben Khelil; Maher Kharrat; Manel Ben Sassi; Mouna Ben Sassi; Mouna Safer; Nissaf Ben Alaya; Riadh Daghfous; Riadh Gouider; Riadh Gouider.; Roua Ben Othman; Rouaa Ben Othman; Salma Abid; Sameh Trabelsi; Sana Ferjani; Sara Chammam; Sarra Chamman; Souissi Amira; Soumaya Rammeh; Zaineb Hamzaoui
EPI_ISL_1411583	1. Glowny Inspektorat Sanitarny. 2. Diagnostyka. Laboratoria Medyczne.	1. ViroGenetics - BSL3 Laboratory of Virology, Malopolska Centre of Biotechnology, Jagiellonian University; 2. genXone SA, Research & Development Laboratory	Aleksandra Gidlewicz; Anna Brylak; Grzegorz Nowicki; Jakub Grabowski; Karol Szeszko; Krzysztof Pyrc; Lukasz Krych; Maciej Sykulski; Michal Kaszuba; Michal Kowalski; Natalia Drweska-Matelska; Natalia Mazur-Panasiuk; Pawel P Labaj; Piotr Szulc; Sylwia Januszczak; Tomasz Gromowski
EPI_ISL_1499330	1. Glowny Inspektorat Sanitarny. 2. Diagnostyka. Laboratoria Medyczne.	1. ViroGenetics - BSL3 Laboratory of Virology, Małopolska Centre of Biotechnology, Jagiellonian University; 2. genXone SA, Research & Development Laboratory	Aleksandra Gidlewicz; Anna Brylak; Gromowski, T.; Grzegorz Nowicki; Jakub Grabowski; Karol Szeszko; Kowalski, M.; Labaj; Maciej Sykulski; Mazur-Panasiuk, N.; Michał Kaszuba; Natalia Drweska-Matelska; P.P.; Pyrc, K.; Sylwia Januszczak; Szulc, P.; Łukasz Krych
EPI_ISL_1040771, EPI_ISL_2140694, EPI_ISL_2621134	2 Military Hospital wc MAA	NHLS/UCT	Arash Iranzadeh; Bruna Galvao; Carolyn Williamson; Deelan Doolabh; Diana Hardie; Gert Marais; Innocent Mudau; Kruger Marais; Lynn Tyers; Marvin Hsiao; Stephen Korsman
EPI_ISL_3275166	AA. Rasdhoo	Indira Gandhi Memorial Hospital	Dr. Milza Abdul Muhsin; Mr. Ibrahim Nishan Ahmed; Ms. Aishath Shuhudha; Ms. Aminath Nazfa; Ms. Fathimath Zimna
EPI_ISL_3347177	ADILAB	Laboratorio Departamental de Salud Publica de Antioquia	Andres F. Cardona-Rios; Gloria Isabel Escobar; Idabely Betancur Ortiz; Juan P. Hernandez-Ortiz; Maria Stella López
EPI_ISL_2621213	ADILAB	Universidad Nacional de Colombia - Laboratorio Genómico One Health	Andres F. Cardona-Rios; Carlos Franco-Muñoz; Carolina Muñoz-Arango; Celeny Ortiz; Daniel O. Maldonado-Perez; Diego A. Álvarez-Díaz; Hector Alejandro Ruiz-Moreno; Idabely Betancur Ortiz; Jorge E. Osorio; Juan P. Hernandez-Ortiz; Karl A Ciuoderis; Katherine Laiton-Donato; Laura Silvana Perez; Lina M. Hurtado; Marcela Mercado-Reyes; Maria Angélica Maya; Maria Stella López; Rita Almanza Payares; Sandra Ines Cano; Simón Villegas Velásquez
EPI_ISL_3118670	AFRICA_CDC - Angola	KRISP, KZn Research Innovation and Sequencing Platform	Emmanuel SJ; Giandhari J; Lessells R; Pillay S; Tegally H; Wilkinson E; Yajna R; de Oliveira T
EPI_ISL_2617056, EPI_ISL_2617092	AFRICA_CDC - Angola (Ship 12)	CERI, Centre for Epidemic Response and Innvoation, Stellenbosch University and KRISP, KZN Research Innovation and Sequencing Platform, UKZN.	Afonso P; David K; Emmanuel SJ; Freitas RH; Giandhari J; Inglês L; Lutucuta S; Miranda J; Morais J; Mufinda M; Naidoo Y; Neto Z; Paulo A Carralero RR Paixão JP; Pereira A; Pillay S; Tegally H; Wilkinson E; de Oliveira T
EPI_ISL_2087004	AMPATH LABORATORIES	National Institute for Communicable Diseases of the National Health Laboratory Service	Amoako DG; Bhiman JN; Ismail A; Mahlangu B; Mohale T; Ntuli N; Scheepers C
EPI_ISL_467456, EPI_ISL_467461	AMPATH-DBN	KRISP, KZN Research Innovation and Sequencing Platform	Chimukangara B; Giandhari J; Khan S; Lessells R; Mdlalose K; Pillay S; Tegally H; Wilkinson E; York D; de Oliveira T
EPI_ISL_2272984	AREA DE SALUD GOICOECHEA 2 - CLINICA DR. JIMENEZ NUÑEZ	Inciensa, Instituto Costarricense de Investigación y Enseñanza en Nutrición y Salud	Adriana Godínez; Claudio Soto-Garita; Estela Cordero; Francisco Duarte; Hebleen Porras; Joselyn Prado & Juan Carlos Cartes Parra; José Luis Vargas; Mariela Gutiérrez; Melany Calderón
EPI_ISL_1712386	AREA DE SALUD OREAMUNO-PACAYAS-TIERRA BLANCA	Inciensa, Instituto Costarricense de Investigación y Enseñanza en Nutrición y Salud	Adriana Godínez; Claudio Soto-Garita; Estela Cordero; Francisco Duarte; Hebleen Porras; Joselyn Prado & Carolina Loría Acosta; José Luis Vargas; Mariela Gutiérrez; Melany Calderón
EPI_ISL_2502750	AREA DE SALUD PEREZ ZELEDON	Inciensa, Instituto Costarricense de InvestigaciOn y Enseñanza en NutriciOn y Salud	Adriana Godínez; Claudio Soto-Garita; Estela Cordero; Francisco Duarte; Hebleen Porras; Jose Luis Vargas; Joselyn Prado & Fabricio Aguilar; Mariela Gutierrez; Melany CalderOn
EPI_ISL_2658275	AREA DE SALUD SAN RAFAEL	Inciensa, Instituto Costarricense de Investigación y Enseñanza en Nutrición y Salud	Adriana Godínez; Claudio Soto-Garita; Estela Cordero; Francisco Duarte; Hebleen Porras; Joselyn Prado & Javier Zárate-León; José Luis Vargas; Mariela Gutiérrez; Melany Calderón
EPI_ISL_1155709, EPI_ISL_1155711, EPI_ISL_1155713	AX BIO OCEAN	CNR Virus des Infections Respiratoires - France SUD	Antonin Bal; Bruno Lina; Gregory Destras; Gwendolyne Burfin; Hadrien Regue; Laurence Josset; Martine Valette; Quentin Semanas
EPI_ISL_1290839, EPI_ISL_1290840	AXBIO	CNR Virus des Infections Respiratoires - France SUD	Antonin Bal; Bruno Lina; Gregory Destras; Gwendolyne Burfin; Hadrien Regue; Laurence Josset; Martine Valette; Quentin Semanas
EPI_ISL_517616	Academic Hospital Paramaribo	Erasmus Medical Center	Bas Oude Munnink; Dion Gajadin; Ed Ijzerman; Emmanuelle Munger; Gary Gummels; Ingrid Krishnadath; Lycke Woittiez; Marion Koopmans; Mireille Van de Veer; Princes Wongsowidjojo; Radjesh Ori; Rohma Banwari; Stephen Vreden
EPI_ISL_2149802	Aegis Sciences Corporation	Centers for Disease Control and Prevention Division of Viral Diseases, Pathogen Discovery	Adrian Paskey; Alec Vest; Benjamin Rambo-Martin; Christopher Gulvick; Clinton R. Paden; Cyndi Clark; Dakota Howard; Darlene Wagner; Dhwani Batra; Dillon Nall; Duncan MacCannell; Ethan Sanders; Holly Houdeshell; Jason Caravas; Kara Moser; Matthew Hardison; Matthew Schmerer; Ola Kvalvaag; Patrick Campbell; Peter W. Cook; Rob Case; Scott Sammons; Shatavia Morrison; Shaun Westlund; Vikramsinha Ghorpade; Yvette Unoarumhi
EPI_ISL_2617060	Africa_CDC - Malawi P4 S2	CERI, Centre for Epidemic Response and Innvoation, Stellenbosch University and KRISP, KZN Research Innovation and Sequencing Platform, UKZN.	Auld A; Chilima B; Chiwaula M; Emmanuel SJ; Giandhari J; Kaba M; Kampira E; Kasambara W; Kim L; Lessells R; Maida A; Mvula B; Mwangomba W; Naidoo Y; Panja L; Pillay S; Tegally H; Wadonda N; Wilkinson E; de Oliveira T
EPI_ISL_2617046, EPI_ISL_2617090	Africa_CDC - Malawi P6 S2	CERI, Centre for Epidemic Response and Innvoation, Stellenbosch University and KRISP, KZN Research Innovation and Sequencing Platform, UKZN.	Auld A; Chilima B; Chiwaula M; Emmanuel SJ; Giandhari J; Kaba M; Kampira E; Kasambara W; Kim L; Lessells R; Maida A; Mvula B; Mwangomba W; Naidoo Y; Panja L; Pillay S; Tegally H; Wadonda N; Wilkinson E; de Oliveira T
EPI_ISL_2360250, EPI_ISL_2360251	Afzalipoor Hospital	National Influenza Center	A Nejati; F Ajaminejad and T Mokhtari Azad; J Yavarian; K Sadeghi; N Ghavvami; NZ Shafiei Jandaghi; V Salimi
EPI_ISL_3231383, EPI_ISL_3231404	Airport Health Laboratory/Central Health Laboratory	UMR PIMIT	Bahadoor BS; David Wilkinson; Manraj SS; Patrick Mavingui; Pattoo M; Ramuth M; Sonoo J
EPI_ISL_775388, EPI_ISL_775390	Akershus University Hospital, Department for Microbiology and Infectious Disease Control	Norwegian Institute of Public Health, Department of Virology	Atiya R Ali; Hilde Elshaug; Hilde Vollan; Kamilla Heddeland Instefjord; Karoline Bragstad; Kathrine Stene-Johansen; Marie Paulsen Madsen; Olav Hungnes; Rasmus Riis Kopperud
EPI_ISL_2164366	Alberta Precision Labs (APL)	Public Health Agency of Canada (PHAC) National Microbiology Laboratory	Buss; Croxen M; Deo A; Dieu P; E; Ferrato C; Gill K; Khan F; Koleva P; Li V; Lloyd C; Lynch T; Ma R; Murphy S; Pabbaraju K; Shokoples S; Thayer J; Tipples G; Whitehouse M; Wong A; Yu C; Zelyas N
EPI_ISL_2375924	Albow Gardens Clinic wc ALB	NHLS/UCT	Arash Iranzadeh; Bruna Galvao; Carolyn Williamson; Deelan Doolabh; Diana Hardie; Innocent Mudau; Kruger Marais; Lynn Tyers; Marvin Hsiao; Stephen Korsman
EPI_ISL_1385811	Alfa Diagnostica LLC	ONCOGENE LLC	ONCOGENE LLC
EPI_ISL_2375992, EPI_ISL_2621060	Alma CDC wc AHC	NHLS/UCT	Arash Iranzadeh; Bruna Galvao; Carolyn Williamson; Deelan Doolabh; Diana Hardie; Gert Marais; Innocent Mudau; Kruger Marais; Lynn Tyers; Marvin Hsiao; Stephen Korsman
EPI_ISL_2178782, EPI_ISL_2178789, EPI_ISL_2178792, EPI_ISL_2178793, EPI_ISL_2178794	Ampath Laboratories	National Institute for Communicable Diseases of the National Health Laboratory Service	Amoako DG; Bhiman JN; Glass A; Gottberg A; Mahlangu B; Marshall T; Mohale T; Ntuli N; Scheepers C; Viana R
EPI_ISL_3207515, EPI_ISL_3207516, EPI_ISL_3207520	Ampath lab	NHLS/UCT	Arash Iranzadeh; Bruna Galvao; Carolyn Williamson; Deelan Doolabh; Diana Hardie; Gert Marais; Innocent Mudau; Lynn Tyers; Marvin Hsiao; Rageema Joseph; Stephen Korsman
EPI_ISL_527760	Area De Salud La Cruz	Inciensa, Instituto Costarricense de Investigación y Enseñanza	Adriana Godinez & Melany Calderon; Claudio Soto-Garita; Estela Cordero; Francisco Duarte; Hebleen Porras

		en Nutrición y Salud	
EPI_ISL_1121974, EPI_ISL_1121975	Area of Virology, Serology and Virology Division (SAViD), New South Wales Health Pathology Randwick	Virology Research Laboratory; Area of Virology, Serology and Virology Division (SAViD), New South Wales Health Pathology Randwick	Au, J.; Bull, R.; Deveson, I.; Foster, C.; Rawlinson, W.; Ruiz Silva, M.; Van Hal, S.
EPI_ISL_3071136, EPI_ISL_3071137	Armauer Hansen Research Institute	Armauer Hansen Research Institute	Dr. Abebe Genetu Bayih; Dr. Abel Abera Negash; Dr. Adane Mihret; Dr. Alemseged Abdissa; Dr. Andargachew Mulu; Dr. Getachew Tesfaye Beyene; Dr. Markos Markos Abebe; Dr. Tesfaye Gelanew; Mr. Dawit Hailu Alemayehu; Mr. Dessalegn Abeje Tefera; Mr. Fekadu Alemu; Mr. Tamrayehu Seyoum; Mrs. Bethlehem Adnew
EPI_ISL_1315065	Armed Forces Medical Research Laboratories and Blood Bank, Egypt	Department of Microbial Biotechnology, Genetic Engineering Division, National Research Centre	Ahmed Elsayed; Ahmed Taha; Ayman Farghaly; Mohamed Khalifa; Mohamed Shemis; Reham Dawood
EPI_ISL_1315064	Armed Forces Medical Research Laboratories and Blood Bank, Egypt	Department of Microbial Biotechnology, Genetic Engineering Division, National Research Centre,	Ahmed Elsayed; Ahmed Taha; Ayman Farghaly; Mohamed Khalifa; Mohamed Shemis; Reham Dawood
EPI_ISL_2423528	Atlanta VA Medical Center	Genomics and Discovery, Respiratory Viruses Branch, Division of Viral Diseases, Centers for Disease Control and Prevention	Adam Retchless; Anna Kelleher; Anna Uehara; Brian Lynch; Clinton R. Paden; Dhwani Batra; Haibin Wang; Han Jia Justin Ng; Jasmine Padilla; Jing Zhang; Justin Lee; Krista Queen; Mark Burroughs; Mili Sheth; Morgan Davis; Peter Cook; Rachel Marine; Sarah Nobles; Suxiang Tong; Tara Coalter; Yan Li; Ying Tao
EPI_ISL_854034, EPI_ISL_1117950, EPI_ISL_2427167, EPI_ISL_2427170	Austrian Agency for Health and Food Safety (AGES)	Bergthaler laboratory, CeMM Research Center for Molecular Medicine of the Austrian Academy of Sciences	Alexander Lercher; Alexandra Popa; Andreas Bergthaler; Anna Schedl; Bekir Erguner; Benedikt Agerer; Christoph Bock; Christoph Bock; Fabian Amman; Jakob-Wendelin Genger; Jan Laine; Lukas Endler; Maelle Le Moing; Martin Senekowitsch; Martin Senekowitsch; Michael Schuster; Michael Schuster; Petr Triska; Thomas Penz
EPI_ISL_882639	Azerbaijan National Hematology Center Division of Medical Genetics	Azerbaijan National Hematology Center Division of Medical Genetics	Aghayev Agha Rza; Bayramlı Ramin
EPI_ISL_1110299	Azienda Sanitaria dell'Alto Adige Laboratorio Aziendale di Microbiologia e Virologia	Istituto di Genomica Applicata	Davide Scaglione; Eleonora Paparelli; Elisa Masi; Elisabetta Giacobazzi; Elisabetta Pagani; Gabriele Magris; Irena Jurman; Irene Bianconi; Michele Morgante; Stefanie Wieser; Vera Vendramin
EPI_ISL_2710308, EPI_ISL_2710311	BARC / Lancet	KRISP, KZN Research Innovation and Sequencing Platform	Giandhari Jennifer; Naidoo Yeshnee; Pillay Sureshnee; San James; Sisonke; Tegally Houriiyah; Tshabuila Derek; Wilkinson Eduan; Yajna Ramphal; de Oliveira Tulio
EPI_ISL_1754503, EPI_ISL_2259273, EPI_ISL_2259279	BIOMNIS EUROFINS IVRY	Department of Virology, Henri Mondor University Hospital, Assistance Publique Hôpitaux de Paris, Université Paris-Est Créteil, INSERM U955	Alexandre Soulier; Christophe Rodriguez; Elisabeth Trawinski; Guillaume Gricourt; Jean-Michel Pawlotsky; Melissa N'Debi; Slim Fourati; Vanessa Demontant
EPI_ISL_1239445, EPI_ISL_1253541, EPI_ISL_1253542, EPI_ISL_1253544, EPI_ISL_1253549, EPI_ISL_1253550, EPI_ISL_1253551, EPI_ISL_1290837, EPI_ISL_1290841, EPI_ISL_1312377, EPI_ISL_1312378, EPI_ISL_1312379			
see above	BIOMNIS LYON	CNR Virus des Infections Respiratoires - France SUD	Antonin Bal; Bruno Lina; Gregory Destras; Gwendolyne Burfin; Hadrien Regue; Laurence Josset; Martine Valette; Quentin Semanas
EPI_ISL_1312372, EPI_ISL_1312373, EPI_ISL_1312374, EPI_ISL_1312375	BIOMNIS PARIS	CNR Virus des Infections Respiratoires - France SUD	Antonin Bal; Bruno Lina; Gregory Destras; Gwendolyne Burfin; Hadrien Regue; Laurence Josset; Martine Valette; Quentin Semanas
EPI_ISL_2141965	BIOR	Latvian Biomedical Research and Study Centre	Daina Pule; Davids Fridmanis; Elina Dimina; Guntars Zarins; Irena Meistere; Ivars Silamikelis; Janis Klovins; Janis Pjalkovskis; Jurijs Perevoscikovs; Kaspars Megnis; Laila Silamikele; Lauma Freimane; Laura Ansone; Liga Birzniece; Monta Ustinova; Nikita Zrelovs; Uga Dumpis; Una Krumina; Vita Rovite
EPI_ISL_1253568, EPI_ISL_1253569, EPI_ISL_1253570, EPI_ISL_1253571, EPI_ISL_1253572, EPI_ISL_1253573, EPI_ISL_1253577, EPI_ISL_1253578			
see above	BOURG LES VALENCE	CNR Virus des Infections Respiratoires - France SUD	Antonin Bal; Bruno Lina; Gregory Destras; Gwendolyne Burfin; Hadrien Regue; Laurence Josset; Martine Valette; Quentin Semanas
EPI_ISL_1695975, EPI_ISL_1696011, EPI_ISL_1696013, EPI_ISL_2179502	BROUSSAIS	Department of Virology, Henri Mondor University Hospital, Assistance Publique Hôpitaux de Paris, Université Paris-Est Créteil, INSERM U955	Alexandre Soulier; Christophe Rodriguez; Elisabeth Trawinski; Guillaume Gricourt; Jean-Michel Pawlotsky; Melissa N'Debi; Slim Fourati; Vanessa Demontant
EPI_ISL_859580, EPI_ISL_859646	BTC, Khalifa University	BTC, Khalifa University	Al Safar et al
EPI_ISL_2970354	Bacterial and Viral Diseases & Biotechnology and Bioinformatics, Armauer Hansen Research Institute	Bacterial and Viral Diseases & Biotechnology and Bioinformatics, Armauer Hansen Research Institute	A.A.; A.G.; Abdissa, A.; Abebe, M.; Adnew, B.; Alemayehu; Alemu, F.; Bayih; Beyene; D.A.; D.H.; G.T.; Gelanew, T.; Mihrete, A.; Mulu, A.; Negash; Seyoum, T.; Tefera
EPI_ISL_775599	Balai Besar Veteriner Maros	National Institute of Health Research and Development	AA; HA; HD; Ikawati; KD; KNA; Mangidi; Muflihanah; N; Nugraha; Pangesti; Pawestri; Puspa; Puspandari; R; Setiawaty; Soekarso; Subangkit; T; V
EPI_ISL_2859263	Bataan General Hospital and Medical Center	Philippine Genome Center	Alethea R. de Guzman; Anna Ong-Lim; Arianne A. Zamora; Benedict A. Maralit; Carlo M. Lapid; Celia Carlos; Devon Ray Pacial; Diomedes A. Carino; Edsel Maurice Salvaña; El King D. Morado; Elcid Aaron R. Pangilinan; Eva Maria Cutiongco-de la Paz; Francis A. Tablizo; Henrietta Marie Rodriguez; Jaime C. Montoya; Jan Michael C. Yap; Jarvin E. Nipales; Jo-Hannah S. Llames; John Q. Wong; Joshua Gregor A. Dizon; Juan Antonio R. Magalang; Karol Sophia Agape R. Padilla; Kenneth M. Kim; Kris P. Punayan; Krisitna Patriz Dela Cruz; Lindsay Claire D.L. Carandang; Ma. Exanil Plantig; Marc Edsel C. Ayes; Maria Rosario Singh-Vergeire and Cynthia P. Saloma; Maria Sofia L. Yangzon; Marielle M Gamboa; Marissa Alejandria; Nina Francesca Bustamante; Razel Nikka M. Hao; Renato Jacinto Q. Mantaring; Rianna Patricia S. Cruz; Sheila Mae M. Araiza; Yvonne Valerie Austria; Zipporah Mariebelle R. Enriquez; Zyrel V. Mollejon
EPI_ISL_2406459	Battambang Provincial Laboratory	Virology Unit, Institut Pasteur du Cambodge	Cecile Troupin; Chau Darapheak; Chin Savuth; Erik A Karlsson; Jurre Y Siegers; Kraing Sidonn; Leakhena Pum; Ly Sovann; Veasna Duong; Yi Sengdoeurn
EPI_ISL_780383	Bermuda Government Molecular Diagnostics Laboratory (MDL)	Respiratory Virus Unit, National Infection Service, Public Health England	Dr Ayoola Oyinloye (Bermuda); Dr Carika Weldon (Bermuda); PHE Covid Sequencing Team
EPI_ISL_2802860	BioMoLab	Microbiologia Molecular, Instituto SELADIS, Universidad Mayor de San Andrés	Aneth Vasquez Michel; Carmen Delgado Barrera; Oscar M. Rollano-Peñaloza; Sandra Miranda Sardon
EPI_ISL_2802859	BioMoLab	Molecular Genetics Laboratory, Instituto de Investigaciones Químicas, Universidad Mayor de San Andrés	Aneth Vasquez Michel; Carmen Delgado Barrera; Oscar M. Rollano-Peñaloza; Sandra Miranda Sardon
EPI_ISL_1502575	Biochemistry and Molecular Biology Department-Faculty of Medicine, Al-Quds University	Biochemistry and Molecular Biology Department-Faculty of Medicine, Al-Quds University	Al-Jawabreh, A.; Dumaidi, K.; Ereqat, S.; Nasereddin, A.
EPI_ISL_1707275, EPI_ISL_1707792, EPI_ISL_2259323	Biogroup Bio Lam-LCD Saint-Denis	Department of Virology, Henri Mondor University Hospital, Assistance Publique Hôpitaux de Paris, Université Paris-Est Créteil, INSERM U955	Alexandre Soulier; Christophe Rodriguez; Elisabeth Trawinski; Guillaume Gricourt; Jean-Michel Pawlotsky; Melissa N'Debi; Slim Fourati; Vanessa Demontant
EPI_ISL_429995, EPI_ISL_430008, EPI_ISL_878571	Biolab Diagnostic Laboratories	Andersen lab at Scripps Research	Ahmad Tibi; Amid Abdelnour with SEARCH Alliance San Diego; Issa Abu-Dayyeh; Lama Hussein; Lina Mohammad; Zein Naber
EPI_ISL_1406195, EPI_ISL_2105672, EPI_ISL_2105673, EPI_ISL_2105674, EPI_ISL_2658760, EPI_ISL_3149713	Biolab Diagnostic Laboratories	Biolab Diagnostic Laboratories	Ahmad Tibi; Amid Abdelnour; Badia Saddedin; Eiad Atwa; Issa Abu-Dayyeh; Lama Hussein; Shaima Ali; Shayma Ali
EPI_ISL_510532	Biological prevention, army	Biological prevention, army	A.A.; A.F.; A.M. and Soliman; Ali; Amer; B.E.; ELnabrawy; ElGohary; Elhoseiny; Elnagdy; Elnakeeb; Gad; H.A.; Harty; Hassan; Kandeil; Karam; M.A.; M.D.; M.F.; M.G.; Raouf; Seadawy; Shamel; T.A.; W.A.; Y.A.; k.E.
EPI_ISL_2931134, EPI_ISL_2931135	Biology Department, College of Science, Al Muthanna University and Public Health Laboratory, Al-Muthanna Health Directorate	Department of Virology, Faculty of Medicine, University of Helsinki, Helsinki, Finland	Ali Jasim; Batool Kadham Salman; Hussein Alburkat; Hussein Riadh Kitab; Murad Munahi; Nihad Al-Rashedi; Olli Vapalahti; Tarja Sironen; Teemu Smura
EPI_ISL_1749368	Biology Lab, HIA BEGIN	IRBA, 2MI	CHAPUS C.; DEPEILLE A.; GORGE O.; GRANDPERRET V.; JARJAVAL F.; MERENS-GONTIER A.; NOLENT F.; SARILAR V.; VERGUET N.
EPI_ISL_1749312	Biology Lab, HIA LAVERAN	IRBA, 2MI	CHAPUS C.; DEPEILLE A.; FOISSAUD V.; GORGE O.; GRANDPERRET V.; JARJAVAL F.; NOLENT F.; SARILAR V.; VERGUET N.
EPI_ISL_1015369	Biology Lab, HIA PERCY	IRBA, 2MI	FOISSAUD V.; GORGE O.; NOLENT F.; SARILAR V.
EPI_ISL_1749369	Biology Lab, HIA ROBERT PICQUE	IRBA, 2MI	CHAPUS C.; DEPEILLE A.; FILLANCQ C.; GORGE O.; GRANDPERRET V.; JARJAVAL F.; NOLENT F.; SARILAR V.; VERGUET N.
EPI_ISL_2153106	Biology, Gaziantep University	Biology, Gaziantep University	Al-Attar; Khailany; M.O.; M.S.; Ozaslan, M.; R.A.; Rahman
EPI_ISL_1731827	Biomedical Research Foundation of the Academy of Athens (BRFAA)	Greek Genome Center, Biomedical Research Foundation of the Academy of Athens (BRFAA)	Dimitrios Thanos; Emmanouil Athanasiadis; Ioannis Vatsellas; Katerina Zoi; Theodoros Loupis
EPI_ISL_2401498, EPI_ISL_2401515	BioneXt Lab	Laboratoire national de sante, Microbiology, Microbial Genomics Platform	Anke Wienecke-Baldacchino; Catherine Ragimbeau; Fatu Djabi; Jessica Tapp; Lise Pignon; Raoul Salmon; Tamir Abdelrahman; Thibault Ferrandon
EPI_ISL_985062, EPI_ISL_985238	Biorepository and Clinical Virology Laboratory	Ozer Lab	Adeola A. Fowotade; Babafemi O. Taiwo; Egon A. Ozer; Ewean C. Omoruyi; Johnson A. Adeniji; Judd F. Hultquist; Lacy M. Simons; Olubusuyi M. Adewumi; Ramon Lorenzo-Redondo
EPI_ISL_2125904	Bioscientia MVZ Labor Karlsruhe GmbH	Robert Koch Institute	
EPI_ISL_1534276	Bongolethu Clinic wc BLC	NHLS/UCT	Arash Iranzadeh; Bruna Galvao; Carolyn Williamson; Deelan Doolabh; Diana Hardie; Innocent Mudau; Kruger Marais; Lynn Tyers; Marvin Hsiao; Stephen Korsman

EPI_ISL_2360256	Boshehr University of Medical Sciences	National Influenza Center	A Nejati; F Ajaminejad; J Yavarian; K Sadeghi; N Ghavvami and T Mokhtari Azad; NZ Shafiei Jandaghi; V Salimi; Mostafa Salehi-Vaziri
EPI_ISL_965178	Botswana Harvard HIV Reference Laboratory	Botswana Harvard AIDS Institute Partnership	Boitumelo Zuze; Botshelo Radibe; David Lawrence; Dorcas Maruapula; Joseph Makhema; Mosepele Mosepele; Roger Shapiro; Shahin Lockman; Sikhulile Moyo; Simani Gaseitsiwe; Wonderful T. Choga
EPI_ISL_871872, EPI_ISL_935042, EPI_ISL_968214, EPI_ISL_1516857, EPI_ISL_1516867, EPI_ISL_1516870, EPI_ISL_2153476, EPI_ISL_2372269, EPI_ISL_2372271, EPI_ISL_2372273, EPI_ISL_2372276, EPI_ISL_2372277, EPI_ISL_2372280, EPI_ISL_2372283, EPI_ISL_2372286, EPI_ISL_2372291, EPI_ISL_2372297, EPI_ISL_2372299, EPI_ISL_2372301, EPI_ISL_2386152, EPI_ISL_2386153, EPI_ISL_2386154, EPI_ISL_2504067, EPI_ISL_2504068, EPI_ISL_2504069, EPI_ISL_2504071, EPI_ISL_2504072, EPI_ISL_2504073, EPI_ISL_2504074, EPI_ISL_2566206, EPI_ISL_2566207, EPI_ISL_2566208, EPI_ISL_2566209			
see above	Botswana Harvard HIV Reference Laboratory	Botswana Harvard HIV Reference Laboratory	Boitumelo Zuze; Botshelo Radibe; David Lawrence; Dorcas Maruapula; Joseph; Joseph Makhema; Keoratile Ntshambiwa; Kgomotso Moruisi; Legodile Kooepile; Madisa Mine; Makhema; Modisa Motswaledi; Mosepele Mosepele; Ontlametse T. Bareng; Pamela Smith-Lawrence; Roger Shapiro; Shahin Lockman; Sikhulile Dorcas Maruapula; Sikhulile Moyo; Simani Gaseitsiwe; Thongbotho Mphoyakgosi; Wonderful Choga; Wonderful T. Choga
EPI_ISL_2547410	Brandwacht Sat Clinic wc BBC	National Health Laboratory Service/University of Cape Town (NHLS/UCT)	Arash Iranzadeh; Bruna Galvao; Carolyn Williamson; Deelan Doolabh; Diana Hardie; Gert Marais; Innocent Mudau; Lynn Tyers; Marvin Hsiao; Stephen Korsman
EPI_ISL_1001001, EPI_ISL_1001003	Bundeswehr Institute of Microbiology	Bundeswehr Institute of Microbiology	Alexandra Rehn; Enrico Georgi; Malena Bestehorn-Willmann; Markus Antwerpen; Mathias Walter; Roman Wölfel; Sabine Zange
EPI_ISL_1364993, EPI_ISL_1364994	Bundeswehrkrankenhaus Hamburg	Bundeswehr Institute of Microbiology	Alexandra Rehn; Enrico Georgi; Malena Bestehorn-Willmann; Markus Antwerpen; Mathias Walter; Mike Pillukat; Roman Wölfel; Sabine Zange
EPI_ISL_1364949, EPI_ISL_1364962, EPI_ISL_1364968, EPI_ISL_1364969	Bundeswehrzentralkrankenhaus Koblenz	Bundeswehr Institute of Microbiology	Alexandra Rehn; Enrico Georgi; Malena Bestehorn-Willmann; Markus Antwerpen; Mathias Walter; Mike Pillukat; Roman Wölfel; Sabine Zange
EPI_ISL_2779345	Bungoma County Referral Hospital	USAMRD-A, Basic Science Laboratory	Alan Lemtudo; Beth Mutai; Brian Andika; Carol Kifude; Clement Masakwe; Eric Muthanje; Esther Omuseni; Faith Sigei; Gathii Kimita; George Awinda; John Waitumbi; Josphat Nyataya; Rachel Githii; Rehema Liyai; Stephen Ochola
EPI_ISL_2558062	Bureau Of Quarantine (BOQ)	Philippine Genome Center	Alethea R. de Guzman; Anna Ong-Lim; Arianne A. Zamora; Asia Louisa U. Chong; Benedict A. Maralit; Candice Francheska B. Tambaoan; Carlo M. Lapid; Celia Carlos; Devon Ray Pacial; Edsel Maurice Salvaña; El King D. Morado; Elcid Aaron R. Pangilinan; Eva Maria Cutiongco-de la Paz; Francis A. Tablizo; Irish Coleen A. Asin; Jaime C. Montoya; Jan Michael C. Yap; Jo-Hannah S. Llames; John Q. Wong; Joshua Gregor A. Dizon; Juan Antonio R. Magalang; Karol Sophia Agape R. Padilla; Kenneth M. Kim; Kris P. Punayan; Marc Edsel C. Ayes; Maria Rosario Singh-Vergeire and Cynthia P. Saloma; Maria Sofia L. Yangzon; Marissa Alejandria; Razel Nikka M. Hao; Renato Jacinto Q. Mantaring; Rianna Patricia S. Cruz; Sheila Mae M. Araiza
EPI_ISL_2779387, EPI_ISL_2779396, EPI_ISL_3031380	Busia County Referral Hospital	USAMRD-A, Basic Science Laboratory	Alan Lemtudo; Beth Mutai; Brian Andika; Carol Kifude; Clement Masakwe; Eric Muthanje; Esther Omuseni; Faith Sigei; Gathii Kimita; George Awinda; John Waitumbi; Josphat Nyataya; Rachel Githii; Rehema Liyai; Stephen Ochola
EPI_ISL_2779289	Busia border point	USAMRD-A, Basic Science Laboratory	Alan Lemtudo; Beth Mutai; Brian Andika; Carol Kifude; Clement Masakwe; Eric Muthanje; Esther Omuseni; Faith Sigei; Gathii Kimita; George Awinda; John Waitumbi; Josphat Nyataya; Rachel Githii; Rehema Liyai; Stephen Ochola
EPI_ISL_1371903	C H DE LA POLYNESIE FRANCAISE	CNR Virus des Infections Respiratoires - France SUD	Antonin Bal; Bruno Lina; Gregory Destras; Gwendolyne Burfin; Hadrien Regue; Laurence Josset; Martine Valette; Quentin Semanas
EPI_ISL_1706829, EPI_ISL_1706830, EPI_ISL_1706832, EPI_ISL_1706833, EPI_ISL_1706835	C.H.C.B KERIO	Department of Virology, Henri Mondor University Hospital, Assistance Publique Hôpitaux de Paris, Université Paris-Est Créteil, INSERM U955	Alexandre Soulier; Christophe Rodriguez; Elisabeth Trawinski; Guillaume Gricourt; Jean-Michel Pawlotsky; Melissa N'Debi; Slim Fourati; Vanessa Demontant
EPI_ISL_1287759	CDP HUSSEL VIENNE	CNR Virus des Infections Respiratoires - France SUD	Antonin Bal; Bruno Lina; Gregory Destras; Gwendolyne Burfin; Hadrien Regue; Laurence Josset; Martine Valette; Quentin Semanas
EPI_ISL_605780	CEIRS Data Processing and Coordinating Center, St. Jude Center of Excellence for Influenza Research and Surveillance (CEIRS)	CEIRS Data Processing and Coordinating Center, St. Jude Center of Excellence for Influenza Research and Surveillance (CEIRS)	A.E.; Ali; El-Guindy; El-Sayes, M.; El-Shesheny, R.; El-Taweel, A.; Gomaa, M.; Kamel; Kandeil, A.; Kayali, G.; Kayed; Kutkat, O.; M.A.; M.N.; Mahmoud; Mahrous, N.; Moatasim, Y.; Mostafa, A.; N.M.; Naguib, A.; Roshdy; S.H.; Shehata, M.; Showky, S.; W.H.; Webby, R.
EPI_ISL_1287752	CERBALLIANCE PACA	CNR Virus des Infections Respiratoires - France SUD	Antonin Bal; Bruno Lina; Gregory Destras; Gwendolyne Burfin; Hadrien Regue; Laurence Josset; Martine Valette; Quentin Semanas
EPI_ISL_1313012, EPI_ISL_1313014	CERBALLIANCE RHONE ALPES	CNR Virus des Infections Respiratoires - France SUD	Antonin Bal; Bruno Lina; Gregory Destras; Gwendolyne Burfin; Hadrien Regue; Laurence Josset; Martine Valette; Quentin Semanas
EPI_ISL_1910917	CH Bethune	CHU Lille	AIT YAHYA Emilie; ALIDJINOU Enagnon Kazali; BOCKET Laurence; CREPIN Michel; DEMAY Christophe; ENGELMANN Ilka; GEFFROY Sandrine; GUIGON Aurélie; LAMBERT Valérie; LAZREK Mouna; NOBILLIAUX Florian; PREVOST Brigitte; THUILLIER Caroline; TINEZ Claire
EPI_ISL_1671818	CH Bethune	CHU Lille - Laboratoire de Virologie	AIT YAHYA Emilie; ALIDJINOU Enagnon Kazali; BOCKET Laurence; CREPIN Michel; DEMAY Christophe; ENGELMANN Ilka; GEFFROY Sandrine; GUIGON Aurélie; LAMBERT Valérie; LAZREK Mouna; NOBILLIAUX Florian; PREVOST Brigitte; THUILLIER Caroline; TINEZ Claire
EPI_ISL_1448016	CH MAUBEUGE	CHU Lille - Laboratoire de Virologie	AIT YAHYA Emilie; ALIDJINOU Enagnon Kazali; BOCKET Laurence; CREPIN Michel; DEMAY Christophe; ENGELMANN Ilka; GEFFROY Sandrine; GUIGON Aurélie; LAMBERT Valérie; LAZREK Mouna; NOBILLIAUX Florian; PREVOST Brigitte; TCHANTCHOU NJOSSE YANICK; THUILLIER Caroline; TINEZ Claire
EPI_ISL_2562031, EPI_ISL_2562045	CH Princesse Grace	Cerba lab	Aude Lessenne; Bénédicte Roquebert; Emmanuel Lecorche; Kader Merah; Laura Verdurme; Patrice Herisson; Sabine Trombert-Paolantoni; Stéphanie Haïm-Boukobza; Thierry Collin
EPI_ISL_1707498	CH VALENCE	CNR Virus des Infections Respiratoires - France SUD	Antonin Bal; Bruno Lina; Gregory Destras; Gwendolyne Burfin; Hadrien Regue; Laurence Josset; Martine Valette; Quentin Semanas
EPI_ISL_894220, EPI_ISL_894221, EPI_ISL_894222, EPI_ISL_894226, EPI_ISL_894227, EPI_ISL_894228, EPI_ISL_894229, EPI_ISL_894230, EPI_ISL_912403			
see above	CH de Mayotte - Laboratoire de Biologie	National Reference Center for Viruses of Respiratory Infections, Institut Pasteur, Paris	Angela Brisebarre; Camille Capel; Combe Patrice; Etienne Simon-Lorière; Marion Barbet; Maud Vanpeene; Méline Bizard; Sylvie Behillil; Sylvie van der Werf; Vincent Enouf
EPI_ISL_1672661, EPI_ISL_2333978	CH.INTERCOMMUNAL DE CRETEIL	Department of Virology, Henri Mondor University Hospital, Assistance Publique Hôpitaux de Paris, Université Paris-Est Créteil, INSERM U955	Alexandre Soulier; Christophe Rodriguez; Elisabeth Trawinski; Guillaume Gricourt; Jean-Michel Pawlotsky; Melissa N'Debi; Slim Fourati; Vanessa Demontant
EPI_ISL_2004292	CHLC	Instituto Nacional de Saude (INSA)	Borges et al
EPI_ISL_2273012, EPI_ISL_2273037, EPI_ISL_2273053, EPI_ISL_2382226, EPI_ISL_2382228, EPI_ISL_2382230, EPI_ISL_2382236, EPI_ISL_2382237, EPI_ISL_2382249, EPI_ISL_2382258, EPI_ISL_2382692, EPI_ISL_2454653, EPI_ISL_2454656, EPI_ISL_2582812, EPI_ISL_2662643, EPI_ISL_2662650, EPI_ISL_2662656, EPI_ISL_3024680			
see above	CHRIS HANI BARAGWANATH LABORATORY	National Institute for Communicable Diseases of the National Health Laboratory Service	Amoako DG; Bhiman JN; Everatt J; Ismail A; Mahlangu B; Mnguni A; Mohale T; Ntuli N; Scheepers C
EPI_ISL_660356	CHU Clermont-Ferrand	CNR Virus des Infections Respiratoires - France SUD	Amélie Brebion; Antonin Bal; Audrey Mirand; Bruno Lina; Christel Regagnon; Christine Archimbaud; Cécile Henquell; Gregory Destras; Gwendolyne Burfin; Hadrien Règue; Hélène Chabrolles; Laurence Josset; Martine Chambon; Martine Valette; Maxime Bisseux; Patricia Combes; Quentin Semanas
EPI_ISL_1425079, EPI_ISL_1448425	CHU Purpan - Laboratoire de Virologie - Institut Fédératif de Biologie	CHU Purpan - Laboratoire de Virologie - Institut Fédératif de Biologie	Agnès Harter; Jacques Izopet; Justine Latour; Martine Dubois; Nicolas Jeanne; Noémie Ranger; Pauline Boyer; Pauline Tremeaux; Romain Carcenac
EPI_ISL_1290836	CHU Rennes	CNR Virus des Infections Respiratoires - France SUD	Antonin Bal; Bruno Lina; Gregory Destras; Gwendolyne Burfin; Hadrien Regue; Laurence Josset; Martine Valette; Quentin Semanas
EPI_ISL_1313709, EPI_ISL_1707485	CHU ST ETIENNE HOPITAL NORD	CNR Virus des Infections Respiratoires - France SUD	Antonin Bal; Bruno Lina; Gregory Destras; Gwendolyne Burfin; Hadrien Regue; Laurence Josset; Martine Valette; Quentin Semanas
EPI_ISL_660670	CHU Toulouse	CNR Virus des Infections Respiratoires - France SUD	Antonin Bal; Bruno Lina; Gregory Destras; Gwendolyne Burfin; Hadrien Règue; Jean Michel Mansuy; Laurence Josset; Martine Valette; Quentin Semanas
EPI_ISL_1370388, EPI_ISL_1370392	CHUV	Laboratory of genomics and metagenomics, Institute of Microbiology, University Hospital Centre and University of Lausanne, Switzerland	Claire Bertelli; Damien Jacot; Gilbert Greub; Sébastien Aeby; Trestan Pillonel
EPI_ISL_683835	CICM	Malaria Research and Training Center (MRTC-Parasito)	Abdoulaye Djimde; Antoine Dara
EPI_ISL_2682597	CICM, Bamako	Malaria Research and Training Center-Bamako	Abdoul Karim Sangare; Abdoulaye Djimde; Amadou Daou; Antoine Dara; Bourema Kouriba
EPI_ISL_487446	CICM-Mali	Bundeswehr Institut of Microbiology	Antwerpen; Bestehorn-Willmann; Dürr; Heitzer; Kouriba; Maiga; Quedraogo; Rehn; Sangaré; Sogodogo; Traoré; Walter; Wölfel; Zimmermann
EPI_ISL_1299497, EPI_ISL_1591100	CMA Dano	Centre Muraz	Abdoul-Salam Ouedraogo; Ange Badjo; Armel Poda; Arsène Somé; Arsène Zongo; Essia Belarbi; Fabian Leendertz; Firmin Kaboré; Grit Schubert; Jasmin Schlotterbeck; Soumeya Ouangraoua; Thérèse Kagone; Yacouba Sawadogo
EPI_ISL_1239446, EPI_ISL_1253567, EPI_ISL_1312825, EPI_ISL_1313667, EPI_ISL_1313693, EPI_ISL_1314016, EPI_ISL_1707523, EPI_ISL_1707526, EPI_ISL_1707528, EPI_ISL_1707535, EPI_ISL_1707536, EPI_ISL_1707537			
see above	CNR Virus des Infections Respiratoires - France SUD	CNR Virus des Infections Respiratoires - France SUD	Antonin Bal; Bruno Lina; Gregory Destras; Gwendolyne Burfin; Hadrien Regue; Laurence Josset; Martine Valette; Quentin Semanas
EPI_ISL_450841	COVID-19 Laboratory	DNA Solution Ltd	ABM Khademul Islam; AHM Nurun Nabi; Abu Sufian; Gazi Nurun Nahar; Habibul Bari Shozib; Haseena Khan; Imran Khan; Latiful Bari; M Anwar Hossain.; MA Malek; Mamun Ahmed; Md Imdadul Hoque; Md Ismail Hosen; Md Mizanur Rahman; Mohammad Riazul Islam; Nazmul Ahsan; Richard Malo; Sabita Rezwana Rahman; Sabrina Moriom Elius; Shahryar Nabi; Sharif Akhteruzzaman; Zeba Islam Seraj
EPI_ISL_450840	COVID-19 Laboratory	DNA Solution Ltd. L-5	ABM Khademul Islam; AHM Nurun Nabi; Abu Sufian; Gazi Nurun Nahar; Habibul Bari Shozib; Haseena Khan; Imran Khan; Latiful Bari; M Anwar Hossain.; MA Malek; Mamun Ahmed; Md Imdadul Hoque; Md Ismail Hosen; Md Mizanur Rahman; Mohammad Riazul Islam; Nazmul Ahsan; Richard Malo; Sabita Rezwana Rahman; Sabrina Moriom Elius; Shahryar Nabi; Sharif Akhteruzzaman; Zeba Islam Seraj
EPI_ISL_2227271	COVID-19 National Reference Laboratory, Pasteur Institute of Iran.	Genetics Research Center, University of Social Welfare and Rehabilitation Sciences	Hossein Najmabadi.; Kimia Kahrizi; Mahsa Tavakoli; Marzieh Mohseni; Mohammad Hassan Pouriayevali; Mostafa Salehi-Vaziri; Tahmineh Jalali; Zohreh Fattahi
EPI_ISL_3274158, EPI_ISL_3274163, EPI_ISL_3274164	CPHL/MOH/EGYPT	CPHL/MOH/EGYPT	Wael H. Roshdy/ Mohamed Kamal / Shymaa s. Ahmed/ Ramy Galal/Nancy el guindy/ Amel nagiub/yasser el hady/salma sayed/ Abd Monaem Adel/Galal Mahmoud/Dalia Ramadan/Rabeh ..R. El/Shesheny/ Mohamed A Ali/Mohamed Hassany; Wael H. Roshdy/ Mohamed Kamal / Shymaa s. Ahmed/ Ramy Galal/Nancy el guindy/ Amelia nagiub/salma sayed/ Abd Monaem Adel/Galal Mahmoud/Dalia Ramadan/Rabeh ..R. El/Shesheny/ Mohamed A Ali/Mohamed Hassany
EPI_ISL_1190754	CREMER(Centre de Rechercherches sur les Maladies Emergentes et Ré-émergentes)	TransVIHMI(Recherches Translationnelles sur le VIH et les Maladies Infectieuses)	Ahidjo Ayouba; Celestin Godwe; Christelle Butel; Dowbiss Meta Djomsi; Eitel Mpoudi Ngole; Eric Delaporte; Esemu Livo; Laetitia Serrano; Marcel Tongo; Marie Amougou; Martin Maidadi Foudi; Martine Peeters; Nicole Vidal; Rodrigue Kamga
EPI_ISL_636980	CS Xai Xai	KRISP, KZN Research Innovation and Sequencing Platform	Giandhari J; Ismael N; Nadia Sitoe; Nedio Mabunda; Paulo Arnaldo; Pillay S; Tegally H; Wilkinson E; de Oliveira T

EPI_ISL_910038	CSIR-Centre for Cellular and Molecular Biology	CSIR-Centre for Cellular and Molecular Biology	Archana Bharadwaj Siva; B Himasri; Blessy B John; Divya Tej Sowpati; Karthik Bharadwaj Tallapaka; Lamuk Zaveri; Namami Gaur; Payel Mukherjee; Pratheusa Maccha; Priya Singh; Purushotham Vodnala; Rakesh K Mishra; Sofia Banu; Tulasi Nagabandi; Viswagithe S L
EPI_ISL_2754313	CURE	Institut Pasteur de Montevideo	Andres Lizasoain; Belén González; Cecilia Alonso; Daiana Mir; Emiliano Pereira; Gonzalo Bello; Ighor Arantes; Juan Zanetti; Lucia Bilbao; Luciana Griffero; Lucía Spangenberg; Mailen Arleo; Mariana Brandes; María José Benítez-Galeano; Matías Castells; Matías Salvo; Matías Victoria; Mauricio Méndez; Melissa Duquía; Natalia Rego; Natalia Reyes; Odhille Chappos; Pablo Smircich; Pía Techera; Rodney Colina; Tamara Fernández-Calero; Tania Possi; Verónica Noya
EPI_ISL_2547362	Calvinia RSC Clinic	National Health Laboratory Service/University of Cape Town (NHLS/UCT)	Arash Iranzadeh; Bruna Galvao; Carolyn Williamson; Deelan Doolabh; Diana Hardie; Gert Marais; Innocent Mudau; Lynn Tyers; Marvin Hsiao; Stephen Korsman
EPI_ISL_1096139, EPI_ISL_1096140	Cambodian National Public Health Laboratory, National Institute of Public Health	Virology Unit, Institut Pasteur du Cambodge	Chau Darapheak; Chin Savuth; Erik A Karlsson; Kraing Sidonn; Ly Sovann; Sokhoun Yann; Veasna Duong; Yi Sengdoeurn
EPI_ISL_862797	Cancer Biology Department, National Cancer Institute	Cancer Biology Department, National Cancer Institute	A.A.; A.E.; A.N.; Abouelhoda, M.; Ahmed; Bahnassy; Elhosieny; F.W.; Gad; H.K.; Hafez; Hamdy; M.G.; M.M.; M.S.; O.S.; Sedawy; Soliman; Soliman, L.; Zekri
EPI_ISL_622786	Canterbury Health Laboratories	Institute of Environmental Science and Research (ESR)	Anja Werno; Antje van der Linden; Arlo Upton; Chris Mansell; David Hammer; Dragana Drinkovic; Erasmus Smit; Gary McAuliffe; Hana Sofia Andersson; Hermes Perez; James Ussher; Jill Sherwood; Jing Wang; Joep de Ligt; Josh Freeman; Julia Howard; Juliet Elvy; Lauren Jelly; Mary DeAlmeida; Matt Blakiston; Matt Storey; Matthew Rogers; Max Bloomfield; Michael Addidle; Michelle Balm; Muhammad Faisal; Nikki Freed; Olin Silander; Sally Roberts; Sarah Jefferies; Sharmini Muttaiyah; Susan Morpeth; Susan Taylor; Timothy Blackmore; Vani Sathyendran; Veronica Playle; Virginia Hope; Xiaoyun Ren
EPI_ISL_811127	Cantonal Hospital Winterthur	Institute of Medical Virology, University of Zurich	Alexander Wepf; Alexandra Trkola; Annette Audigé; Cyril Shah; Guido Bloemberg; Jon Huder; Jürg Böni; Kevin Steiner; Maria Grünberg; Maryam Zaheri; Michael Huber; Riccarda Capaul; Stefan Schmutz; Urs Karrer; Verena Kufner
EPI_ISL_977656	Caribbean Public Health Agency	Carrington Lab, Department of PreClinical Sciences, Building 36, First Floor Biochemistry Unit, Faculty of Medical Sciences, The University of the West Indies	Adesh Ramsubhag; Arianne Brown-Jordan; Avery Hinds; Chinna Chinnadurai; Christine V. F. Carrington; Christopher Oura; Gabriel Escobar; Jaya Jayaraman; Jerome Foster; Karla Georges; Kenneth George; Marsha Ivey; Naresh Nandram; Nikita S. D. Sahadeo; Nuno Faria; Oliver Pybus; Rahul Naidu; Rajini Haraksingh; Risha Singh; Sarah Hill; Stanley Giddings; SueMin Nathaniel; Vernie Ramkissoon
EPI_ISL_872193	Caribbean Public Health Agency	Carrington Lab, Department ofBuilding 36, First Floor Biochemistry Unit, Faculty of Medical Sciences, The University of the West Indies	Adesh Ramsubhag; Arianne Brown-Jordan; Avery Hinds; Chinna Chinnadurai; Christine V. F. Carrington; Christopher Oura; Gabriel Escobar; Jaya Jayaraman; Jerome Foster; Karla Georges; Marsha Ivey; Naresh Nandram; Nikita S. D. Sahadeo; Nuno Faria; Oliver Pybus; Rahul Naidu; Rajini Haraksingh; Risha Singh; Sarah Hill; Stanley Giddings; SueMin Nathaniel; Vernie Ramkissoon
EPI_ISL_522446	Center for Laboratory Control of Infectious Diseases, Korea Centers for Diseases Control and Prevention	Center for Laboratory Control of Infectious Diseases, Korea Centers for Diseases Control and Prevention	Ae Kyung Park; Eunkyung Shin; Heui Man Kim; Jeong-Min Kim; Jin Sun No; Junyoung Kim; Myung Guk Han; Yoon-Seok Chung
EPI_ISL_2835622, EPI_ISL_2835623, EPI_ISL_2835625, EPI_ISL_2835635, EPI_ISL_2835636, EPI_ISL_2835637, EPI_ISL_2835640			
see above	Center for Medical and Sanitary Research (CERMES)	CDC Atlanta	Adamou Lagare; Dhwani Batra; Justin Lee
EPI_ISL_475805	Center for Virology, Medical University of Vienna	Bergthaler laboratory, CeMM Research Center for Molecular Medicine of the Austrian Academy of Sciences	Alexander Lercher; Alexandra Popa; Andreas Bergthaler; Benedikt Agerer; Christoph Bock; Daniela Schmid; Dorothee von Laer; Elisabeth Puchhammer-Stoeckl; Franz Allerberger; Gregor Hörmann; Guenter Weiss; Henrique Colaco; Jakob-Wendelin Genger; Jan Laine; Judith Aberle; Kinga Rigler-Hohenwarter; Lukas Endler; Manfred Nairz; Mark Smyth; Martin Senekowitsch; Michael Schuster; Peter Hufnagl; Rainer Gattringer; Stephan Aberle; Thomas Penz; Wegene Borena
EPI_ISL_678248	Center for public health - Skopje	Research Center for Genetic Engineering and Biotechnology "Georgi D. Efremov" , Macedonian Academy of Sciences and Arts	RCGEB - MASA
EPI_ISL_2227328, EPI_ISL_2227329, EPI_ISL_2227331, EPI_ISL_2227339, EPI_ISL_2227350, EPI_ISL_2232273, EPI_ISL_2232407, EPI_ISL_2380078			
see above	Center of Scientific Excellence for Influenza Viruses (CSEIV), National Research Centre	Center of Scientific Excellence for Influenza Viruses (CSEIV), National Research Centre	Ahmed E Kayed; Ahmed El-Taweel; Ahmed Kandeil; Ahmed Mostafa; Amal Naguib; Ghazi Kayali; M K Khalifa; Mahmoud Shehata; Mina Kamel; Mohamed Ahmed Ali; Mohamed El Sayes; Mohamed Hassany; Mokhtar Gomaa; Nancy M. El Guindy; Noura M Abo Shama; Omnia Kutkat; Rabeh El-Shesheny; Richard Webby; Sara Mahmoud; Shymaa S Ahmed; Wael Roshdy; Yassmin Moatasim
EPI_ISL_815255, EPI_ISL_815297	Centogene	Centogene	Krishna Kumar Kandaswamy; Peter Bauer; Vivi Hue-Trang Lieu
EPI_ISL_2834910, EPI_ISL_2834911, EPI_ISL_2834914, EPI_ISL_2834915	Central Health Laboratory	Central Health Laboratory ,Victoria Hospital, Candos,Ministry of Health and Wellness, Mauritius	Bahadoor BS; Jannoo N; Manraj SS; Mathur H; Ramuth M; Sonoo J; Sujeewon C
EPI_ISL_2502387, EPI_ISL_2502426, EPI_ISL_2502435, EPI_ISL_2502441	Central Laboratory, Bureau of Public Health (BOG) and Academic Hospital Paramaribo	Erasmus Medical Center	Bas B Oude Munnink; Cherise Beek; Consuella Partowidjojo; Dion Gajadin; Ed PF IJzerman; Emmanuelle Munger; Gary Gummels; Ingrid SK Krishnadath; Lycke Woittiez; Marion PG Koopmans; Mireille Van de Veer; Phyllis Pinas; Princes Wongsowidjojo; Radjesh Ori; Ranisha Doerbalie; Rohma Banwari; Soeradj Harkisoen; Stephen Vreden; Tilotmadebie Ramlal; Verne Nanhoe
EPI_ISL_1822739, EPI_ISL_1822744, EPI_ISL_1822754, EPI_ISL_1822766	Central Medical Laboratory	Texas Children's Microbiome Center	Adrianna Maliga; Francis Morey; Gerhaldine Morazan; Jennifer K. Spinler; Kristy O. Murray; Melissa Diaz-Musa; Miguel Saldana; Russell I Manzanero; Ruth Ann Luna; Sarah M. Gunter; Shannon E. Ronca
EPI_ISL_1322330	Central Public Health Laboratory	National Public Health Laboratory, National Centre for Infectious Diseases	Esorom Daoni; Lin Cui; Raymond Tzer Pin Lin; Theresa Palou; Tze Minn Mak; Zhenyang Zhou
EPI_ISL_2786923	Central Virology Laboratory, Ministry of Health	Central Virology Laboratory, Ministry of Health	Amos Adler; Efrat Bucris; Ella Mendelson; Michal Mandelboim; Moran Shwartswort-Cohen; Neta S. Zuckerman; Noam Protter; Oran Erster; Orna Mor; Saar Burstein
EPI_ISL_885143	Central public health laboratory	Molecular Diagnostics Department, Central public health laboratory	Dalia, F.; Dler, H.; Dlshad, H.; F.-A.; Fahmi, A.; Furat, S.; Hemdad, A.; Hemn; M. and Idrees, H.; Mohsen, A.; Sharmeen
EPI_ISL_1483780	Centre De Prelevement COVID RIOM	CHU Clermont-Ferrand, service de virologie	Bisseux Maxime; Combes Patricia; Henquell Cécile; Mirand Audrey
EPI_ISL_1357604	Centre For Biotechnology Research And Development	THE AFRICA GENOMICS CENTRE AND CONSULTANCY	Adede Hawi; Cecilia Waruhiu; Damaris Matoke-Muhia; George Michuki; John Njuguna; Lilian Kanjau; Ravena Mubichi
EPI_ISL_1706459	Centre Hospitalier Eure Seine	Centre Hospitalier Universitaire de Rouen Laboratoire de Virologie	Alice Moisan; Fabienne De Oliveira; Marie Leoz
EPI_ISL_1483774, EPI_ISL_1483776, EPI_ISL_1483786, EPI_ISL_1490236	Centre Hospitalier Universitaire Clermont-Ferrand	CHU Clermont-Ferrand, service de virologie	Bisseux Maxime; Combes Patricia; Henquell Cécile; Mirand Audrey
EPI_ISL_1760556	Centre Hospitalier Universitaire Mère-Enfant, Fondation Jeanne Ebori (CHUMEFJE)	Centre de recherches médicales de Lambaréné (CERMEL)	Ayola A. Adegnika; Ayong Moure; Bertrand Lell; Bénédicte Ndeboko; Emilio Skarwan; Georgelin Nguema Ondo; Gédéon P. Manouana; Haruka Abe; Jiro Yasuda; Joel Fleury Djoba Siawaya; Rodrigue Bikangui; Rotimi Myrabelle Avome Houechenou; Samira Zoa-Assoumou; Yuri Ushijima
EPI_ISL_660442	Centre Muraz	Project group Epidemology of Highly Pathogenic Microorganisms, Robert Koch Institut	Abdoul-Salam Ouedraogo; Arsène Zongo; Essia Belarbi; Fabian Leendertz; Grit Schubert; Soumeya Ouangraoua; Yacouba Sawadogo
EPI_ISL_1790093, EPI_ISL_1790101	Centre Pasteur Annexe de Garoua	Institut Pasteur de Dakar	Carniel Elisabeth; Dia Ndongo; Diagne Moussa Moïse; Diallo Amadou; Diop Mamadou; Faye Ousmane; Loucoubar Cheikh; Ndiaye Ndack; Njouom Richard; Sall Amadou Alpha; Sankhe Safietou
EPI_ISL_1001043, EPI_ISL_1001049, EPI_ISL_1001052, EPI_ISL_1001056, EPI_ISL_1001057	Centre Pasteur du Cameroun	Institut Pasteur de Dakar	Njouom Richard
EPI_ISL_788935, EPI_ISL_788938	Centre de Recherche et de Formation en Infectiologie Guinée	TransVIHMI, IRD/INSERM/Monpellier University	Abdoul Karim SOUMAH; Abdoulaye TOURE; Ahidjo AYOUBA; Alimou CAMARA; Alpha Kabinet KEITA; Bouna Yatassaye; Christelle BUTEL; Eric DELAPORTE; Jean-louis MONEMOU; Joel KOIVOGUI; Kaba KOUROUMA; Laetitia SERRANO; Mamadou Bhoye KEITA; Mamadou Saliou BAH; Mamadou Saliou SOW; Mandiou DIAKITE; Martine PEETERS; Moriba POVOGUI; Penda Malhado DIALLO; Sakoba KEITA
EPI_ISL_1913035, EPI_ISL_1913078, EPI_ISL_1913080, EPI_ISL_2097228, EPI_ISL_2097229, EPI_ISL_2097230, EPI_ISL_2097231, EPI_ISL_2424136, EPI_ISL_2424137, EPI_ISL_2424159, EPI_ISL_2424160, EPI_ISL_2424161, EPI_ISL_2424162, EPI_ISL_2424163, EPI_ISL_2434970, EPI_ISL_2434971, EPI_ISL_2434974, EPI_ISL_2434975, EPI_ISL_2434979, EPI_ISL_2434980, EPI_ISL_2442203, EPI_ISL_2442276, EPI_ISL_2442277, EPI_ISL_2442279, EPI_ISL_2442338, EPI_ISL_2442363, EPI_ISL_2442383, EPI_ISL_2442384			
see above	Centre de Recherches Médicales de Lambaréné (CERMEL)	Centre de Recherches Médicales de Lambaréné (CERMEL)	Anicet Mouity Matoumba; Bertrand Lell and Ayola Akim Adegnika; Georgelin Nguema Ondo; Gédéon Prince Manouana; Jean Bernard Lekana-Douki; Joël-Fleury Djoba Siawaya; Michel Ngonga Dikongo; Moustapha Nzamba Maloum; Rodrigue Bikangui; Sam O'neilla Oye Bingono; Samira Zoa Assoumou; Srinivas reddy Pallerla; Steffen Borrmann; Thirumalaisamy P. Velavan
EPI_ISL_2968051	Centre de Virologie des Maladies infectueuses Tropicales	Functional Genomic Platform UATRS-biology, CNRST	Ahmed REGGAD; Elmostafa EL FAHIME; Hemlali Mouhssine; Hicham EL ANNAZ; Idriss-Amine LAHLOU; Khalid ENNIBI; Marouane MELLOUL; Mly Abdelaziz ELALAOUI; Mostafa ELOUENNASS; Nadia TOUIL; Rachid ABI; Rida TAGAJDID; Safae ELKOCHRI; Sanaa ALAOUI-Amine; Youssef AKHOUAD; Zohour KASMY
EPI_ISL_1233112, EPI_ISL_1233116, EPI_ISL_1717101, EPI_ISL_1717106, EPI_ISL_1970392, EPI_ISL_2481326, EPI_ISL_2481406			
see above	Centre for Dengue Research and AICBU, Department of Immunology and Molecular Medicine	Centre for Dengue Research and AICBU, Department of Immunology and Molecular Medicine	Chandima Jeewandara; Deshan Madhusanka; Deshni Jayathilaka; Dinuka Ariyaratne; Diyanath Ranasinghe; Gathsaurie Neelika Malavige; Laksiri Gomes; Tibutius Thanesh Pramanayagam
EPI_ISL_668452	Centre for Dengue Research, Department of Immunology and Molecular Medicine	Centre for Dengue Research, Department of Immunology and Molecular Medicine	Chandima Jeewandara; Deshni Jayathilaka; Dinuka Ariyaratne; Diyanath Ranasinghe; Gathsaurie Neelika Malavige; Laksiri Gomes
EPI_ISL_654794	Centre for Human Virology &	Centre for Human Virology & Genomics, Nigerian Institute of	Shaibu, J.

	Genomics, Nigerian Institute of Medical Research	Medical Research	
EPI_ISL_2399462, EPI_ISL_2501079, EPI_ISL_2501085, EPI_ISL_2502021	Centre for Human Virology and Genomics, Microbiology Department, Nigerian Institute of Medical Research	Central Research Laboratory, Nigerian Institute of Medical Research	Ayorinde Babatunde James; Azuka Patrick Okwuraiwe; Babatunde Lawal Salako; Bamidele Iwalokun; Chika Kingsley Onwuamah; Grace Oni; Joseph Ojonugwa Shaibu; Josiah Ayoola Isong; Joy Ayoola; Muinah Adenike Fowora; Ngozi Mirabel Otuonye; Nyam Itse Yusuf; Olufemi Samuel Amoo; Phasha-Muchemenye Mmatshepho; Rahaman A. Ahmed; Rosemary Ajuma Audu; Sharon Abimbola; Sola Ajibaye; Uyi Emokpae; Yusuf Jimoh
EPI_ISL_413550	Centre for Human and Zoonotic Virology (CHAZVY), College of Medicine University of Lagos/Lagos University Teaching Hospital (LUTH), part of the Laboratory Network of the Nigeria Centre for Disease Control (NCDC)	African Centre of Excellence for Genomics of Infectious Diseases (ACEGID), Redeemer's University, Ede, Osun State, Nigeria	Ajogbasile F.V.; Folarin O.A.; Ihekweazu C. Happi C.T.; Kayode A.; Oguzie J.; Oluniyi P.E.
EPI_ISL_413595	Centre for Infectious Diseases and Microbiology Laboratory Services	NSW Health Pathology - Institute of Clinical Pathology and Medical Research; Westmead Hospital; University of Sydney	Carter I; Chen SC; Eden J-S; Gall; Gray K; Holmes EC; Kok J and Dwyer DE for the 2019-nCoV Study Group*; Lam C; M; Maddocks S; O'Sullivan MV; Rahman H; Rockett R; Sintchenko V; Timms; V
EPI_ISL_1469655	Centro Municipal de Saúde de Rolante	Epiclin	Ana Paula Muterle; Carolina Comerlato; Eliana Márcia Da Ros Wendland; Fernando Hayashi Sant'Anna; Janira Prichula; Juliana Comerlato
EPI_ISL_2274037	Centro Nacional de Enfermidades Tropicales (CENETROP)	Laboratory of Respiratory Viruses and Measles, Oswaldo Cruz Institute, FIOCRUZ	Alice Sampaio Rocha; Ana Carolina Mendonca; Anna Carolina Paixao; Cinthia Avila; Elisa Cavalcante Pereira; Fernando Motta; Luciana Appolinario; Marilda Siqueira on behalf of the Fiocruz COVID-19 Genomic Surveillance Network; Paola Resende; Renata Serrano Lopes; Roxana Loayza; Taina Venas
EPI_ISL_1494674	Centro de Investigación en Ciencias de la Salud y Biomedicina, U.A.S.L.P	Centro de Investigación en Ciencias de la Salud y Biomedicina, U.A.S.L.P	MD PhD Sofía Bernal Silva
EPI_ISL_2442164, EPI_ISL_2442181, EPI_ISL_2442185	Centro de Investigação em Saúde de Manhiça (CISM) & ISGlobal, Institut de Salut Global de Barcelona	Instituto de Biomedicina de València - CSIC	Alfredo Mayor; Arsenia Massinga; Inacio Mandomando; Irving Cancino; Iñaki Comas; Manoli Torres Puente; Santiago Jiménez-Serrano
EPI_ISL_1396418	Centro de Tecnología en Salud Pública de la Universidad Nacional de Rosario	Laboratorio Mixto de Biotecnología Acuática (LMBA) on behalf of 'Proyecto Argentino Interinstitucional de genomica de SARS-CoV-2' (PAIS Consortium)	Adriana Giri; Agustina Cerri; Ana Cavatorta; Ana Paletta; Diego Chouhy; Elisa Bolatti; Elizabeth Tapia (argenTAG); Federico Remes Lenicov; Flavio Spetale; Gastón Viarengo; Ignacio García Labari; Javier Murillo; Joaquín Ezpeleta; Julian Acosta; Laura Angelone; Leandro Ciappina; María Re; Pablo Casal; Pilar Bulacio; Silvana Spinelli; Silvia Arranz; Sofía Lavista Llanos; Vanina Villanova; Victoria Posner
EPI_ISL_2798996, EPI_ISL_2799099	Centrālā Laboratorija, SIA	Riga East University Hospital, National Microbiology Reference Laboratory; Eurofins Genomics Europe Sequencing GmbH	Arzu Algulieva; Diāna Dušacka; Dārta Pūpola; Ilva Pole; Jana Osīte; Jevgenijs Bodrenko; Jūlija Čevere; Reinis Vangravs; Reinis Zeltmatis; Sergejs Ņikišins; Stella Lapiņa; Ģirts Šķenders
EPI_ISL_486416, EPI_ISL_534206	Centrālā laboratorija	Latvian Biomedical Research and Study Centre	Ivars Silamiķelis; Jana Osīte; Jānis Kloviņš; Jānis Pjalkovskis; Kaspars Megnis; Marta Priedīte; Monta Ustinova; Stella Lapiņa; Uga Dumpis; Vita Rovīte; Ņikita Zrelovs
EPI_ISL_2504134, EPI_ISL_2504141, EPI_ISL_2504142	Chantal Biya International Reference Centre (CIRCB)	International Centre for Genetic Engineering and Biotechnology (ICGEB) and ARGO Open Lab for Genome Sequencing	Alessandro Marcello; Alexis Ndjolo; Celine Nkenfou; Collins Chenwi; Danilo Licastro; Desire Takou; Emanuele Orsini; Grace Angong Belournou; Joseph Fokam; Ndine Fainguem; Simeone Dal Monego
EPI_ISL_1239292	Charlotte Maxeke Johannesburg Academic Hospital, National Health Laboratory Services, Gauteng, South Africa	National Institute for Communicable Diseases of the National Health Laboratory Service	Amoako DG; Bhiman JN; Ismail A; Mahlangu B; Mohale T; Ntuli N; Scheepers C
EPI_ISL_492028, EPI_ISL_1634462, EPI_ISL_2178951	Child Health Research Foundation	Child Health Research Foundation	Afroza Akter Tanni; CHRF Bangladesh Genomics Team; Maksuda Islam; Md Hafizur Rahman; Md Saiful Islam Sajib; Roly Malaker; Samir K Saha; Senjuti Saha; Syed Muktadir Al Sium
EPI_ISL_1970349	Chongqing International Travel Health Care Center	Chongqing Municipal Center for Disease Control and Prevention	Hua Ling; Mingyue Wang; Rong Rong; Sheng Ye; Wenge Tang; Xingdan Luo; Yanqing Peng; Yun Tang; Zhangping Tan; Zhaohui Deng , Shuang Chen; Zhen Yu
EPI_ISL_3118692	Chris Hani Baragwanath Academic Hospital POC laboratory	KRISP, KZn Research Innovation and Sequencing Platform	Brown MJ; Emmanuel SJ; Giandhari J; Lessells R; Omar S; Pillay S; Tegally H; Wadula J; Wilkinson E; Yajna R; de Oliveira T
EPI_ISL_2839458	City General Hospital "8 Septemvri"- Skopje	Research Center for Genetic Engineering and Biotechnology "Georgi D. Efremov" , Macedonian Academy of Sciences and Arts	Aleksandar J. Dimovski; Dijana Plasheska-Karanfilska; Gjorgji Bozinovski; Milena Jakimovska; Predrag Noveski
EPI_ISL_636981	City of Chimoio	KRISP, KZN Research Innovation and Sequencing Platform	Giandhari J; Ismael N; Nadia Sitoe; Nedio Mabunda; Paulo Arnaldo; Pillay S; Tegally H; Wilkinson E; de Oliveira T
EPI_ISL_1300645, EPI_ISL_1300658	Clinical Center, University of Sarajevo; Unit for Clinical Microbiology	Clinical Center, University of Sarajevo; Unit for Clinical Microbiology	Amela Dedeić-Ljubović; Edina Zahirović; Golubinka Boshevska; Irma Salimović-Bešić; Maja Kuzmanovska; Sandra Vegar-Zubović; Sebija Izetbegović; Suzana Arapčić
EPI_ISL_678259	Clinical Hospital - Shtip	Research Center for Genetic Engineering and Biotechnology "Georgi D. Efremov" , Macedonian Academy of Sciences and Arts	RCGEB - MASA
EPI_ISL_2692993	Clinical Microbiology Laboratory, Faculty of Medicine, Universitas Indonesia	Clinical Microbiology Laboratory, Faculty of Medicine, Universitas Indonesia	Fitrhiyah; Ibrahim F; Karuniawati A; Natasha A; Saharman YR; Sudarmono P.; Yasmon A
EPI_ISL_581704, EPI_ISL_830804	Clinical Virology	Clinical Bacteriology	Adrian Egli; Alexander Gensch; Alfredo Mari; Christian Nickel; Hans Hirsch; Hans Pargger; Helena MB Seth-Smith; Julia Bielicki; Karoline Leuzinger; Kirstine K. Soegaard; Madlen Stange; Manuel Battegay; Martin Siegemund; Michael Osthoff; Michael Schweitzer; Myrta Brunner; Rita Schneider-SliFemalea; Rita Schneider-Sliwa; Roland Bingisser; Sarah Tschudin-Sutter; Simon Fuchs; Stefano Bassetti; Tim Roloff
EPI_ISL_3276927, EPI_ISL_3276991	Clinical Virology Laboratory, Institute of Liver and Biliary Sciences	ILBS	Amit Pandey; Chhagan Bihari Sharma; Diptanu Paul; Ekta Gupta; Reshu Agarwal; Shiv Kumar Sarin; Varun Suroliya
EPI_ISL_447335	Clinical Virology Unit, Hadassah Hebrew University Medical Center	Stern Lab	Stern Lab
EPI_ISL_1935606, EPI_ISL_2492428	Cliniques universitaires Saint-Luc	UCLouvain/IREC/MBLG	Benoit Kabamba Mukadi; Eleonore Ngyuvula; Jean Ruelle
EPI_ISL_1234167	Colorado Department of Public Health and Environment	Colorado Department of Public Health and Environment	Diana Ir; Emily A. Travanty; Laura Bankers; Molly C. Hetherington-Rauth; Sarah Elizabeth Totten; Shannon Ely; Shannon R. Matzinger
EPI_ISL_681309, EPI_ISL_2715285, EPI_ISL_2715287, EPI_ISL_2715288, EPI_ISL_2932624, EPI_ISL_2932655, EPI_ISL_2932663			
see above	Communicable Disease Laboratory, Public Health Directorate	Communicable Disease Laboratory, Public Health Directorate	AlAbbas, Z.; AlHujairi, Z.; Almoamen, G.; Altaif, Z.; Alwasti, H.; Marhoon, A.
EPI_ISL_2375921	Concordia Clinic	NHLS/UCT	Arash Iranzadeh; Bruna Galvao; Carolyn Williamson; Deelan Doolabh; Diana Hardie; Innocent Mudau; Kruger Marais; Lynn Tyers; Marvin Hsiao; Stephen Korsman
EPI_ISL_3014108	Cotugno	TIGEM	Antonio Grimaldi Patrizia Annunziata Francesco Panariello Biancamaria Pierri Claudia Tiberio Teresa Giuliano Valentina Bouche Chiara Colantuono Maria Concetta Cuomo Denise Di Concilio Lucio Di Filippo Anna Manfredi Marcello Salvi Antonio Limone Luigi Atripaldi Pellegrino Cerino Andrea Ballabio Davide Cacchiarelli
EPI_ISL_1084757	Croatian Institute of Public Health	Croatian Institute of Public Health	Irena Tabain; Ivana Ferenčak
EPI_ISL_3105882	Cruz-Rabe Maternity and General Hospital	Research Institute for Tropical Medicine	Clyde Dapat; Deana Mae Ocampo; Emmanuel Kagning Tsinda; Francisco Gerardo Polotan; Hitoshi Oshitani; Inez Andrea Medado; Jefferson Earl Halog; Joana Ina Manalo; Lei Lanna Dancel; Ma Angelica Tujan; Mariko Saito-Obata; Mayuko Saito; Michiko Okamoto; Samantha Louise Bado
EPI_ISL_2153497	DR GEORGE MUKHARI LABORATORY	National Institute for Communicable Diseases of the National Health Laboratory Service	Amoako DG; Bhiman JN; Ismail A; Mahlangu B; Mohale T; Ntuli N; Scheepers C
EPI_ISL_2595726, EPI_ISL_2596341, EPI_ISL_2597312, EPI_ISL_2612300	DSMRC	DSMRC	Khine Zaw Oo; Ko Ko Lwin; Ko Ko Win; Kyee Myint; Nay Myo Aung; Pai Khant Kyaw; Phyo Kyaw Aung; Thet Wai Oo; Zaw Win Htun
EPI_ISL_1312896	DYOMEDEA LYON	CNR Virus des Infections Respiratoires - France SUD	Antonin Bal; Bruno Lina; Gregory Destras; Gwendolyne Burfin; Hadrien Regue; Laurence Josset; Martine Valette; Quentin Semanas
EPI_ISL_1457768	DYOMEDEA SAUVEGARDE LYON	CNR Virus des Infections Respiratoires - France SUD	Antonin Bal; Bruno Lina; Gregory Destras; Gwendolyne Burfin; Hadrien Regue; Laurence Josset; Martine Valette; Quentin Semanas
EPI_ISL_2372406, EPI_ISL_2648206, EPI_ISL_2648207, EPI_ISL_2648208, EPI_ISL_2648209, EPI_ISL_2648211, EPI_ISL_2648214, EPI_ISL_2648215, EPI_ISL_2648217, EPI_ISL_2648218, EPI_ISL_2648219, EPI_ISL_2648220			
see above	Debswana Orapa Mine Hospital Laboratory	Botswana Harvard HIV Reference Laboratory	Boitumelo Zuze; Botshelo Radibe; Dorcas Maruapula; Joseph Makhema; Keoratile Ntshambiwa; Kgomotso Moruisi; Koketso Maotwe; Legodile Kooepile; Lesedi Magama; Madisa Mine; Mosepele Mosepele; Mpo Molapisi; Ontlametse T. Bareng; Pamela Smith-Lawrence; Roger Shapiro; Shahin Lockman; Sikhulile Moyo; Simani Gaseitsiwe; Thongbotho Mphoyakgosi; Wonderful T. Choga
EPI_ISL_2432518, EPI_ISL_2432922, EPI_ISL_2432953	Demy Health	National Reference Laboratory, Nigeria Centre for Disease Control	Anthony Ahumibe; Catherine Okoi; Chimaobi Chukwu; Dr Chikwe Ihekweazu; Dr Ndodo Nnaemeka; Dr Omoare Adesuyi; Grace Esebanmen; Naidoo Dhamari; Nwando Mba; Olusola Anuoluwapo Akanbi
EPI_ISL_1340764	Departamento de Virologia, Laboratorio Central de Salud Pública,	Laboratory of Respiratory Viruses and Measles, Oswaldo Cruz Institute, FIOCRUZ	Alice Sampaio Rocha; Ana Carolina Mendonca; Anna Carolina Paixao; Cynthia Vazquez; Fernando Motta; Luciana Appolinario; Marilda Siqueira on behalf of the Fiocruz COVID-19 Genomic Surveillance Network; Paola Resende; Renata Serrano Lopes

	Avenida Venezuela y Teniente Escurra, Asunción, Paraguay		
EPI_ISL_1013600	Department for Molecular Diagnostics, Centre for Medical Microbiology, Institute of Public Health, Montenegro	Charité Universitätsmedizin Berlin, Institut für Virologie	Barbara Mühlemann; Christian Drosten; Danijela Vujošević; Julia Schneider; Julia Tesch; Jörn Beheim-Schwarzbach; Marija Govedarica; Talitha Veith; Terry Jones; Tobias Bleicker; Victor M Corman
EPI_ISL_420142	Department for Virology, Molecular Biology and Genome Research, R. G. Lugar Center for Public Health Research, National Center for Disease Control and Public Health (NCDC) of Georgia.	Department for Virology, Molecular Biology and Genome Research, R. G. Lugar Center for Public Health Research, National Center for Disease Control and Public Health (NCDC) of Georgia.	Adam Kotorashvili; Amiran Gamkrelidze.; Ana Papkiauri; Ann Machablishvili; Anna Kasradze; Davit Tsaguria; Ekaterine Khmaladze; Ekaterine Zangaladze; Ekaterine Zhghenti; Giorgi Tomashvili; Gvantsa Brachveli; Gvantsa Chanturia; Irma Burjanadze; Ketevan Sidamonidze; Khatuna Zakhashvili; Lela Sabadze; Lela Urushadze; Magda Dgebuadze; Maia Alkhazashvili; Mari Gavashelidze; Mariam Zakalashvili; Marine Murtskhvaladze; Meri Pantsulaia; Nino Berishvili; Paata Imnadze; Roena Sukhiashvili; Tamar Jashiashvili; Tata Imnadze; Tea Tevdoradze
EPI_ISL_1914665, EPI_ISL_1914783, EPI_ISL_3098702, EPI_ISL_3204239, EPI_ISL_3204245	Department for Virology, Molecular Biology and Genome Research, R. G. Lugar Center for Public Health Research, National Center for Disease Control and Public Health (NCDC) of Georgia.	Department for Virology, Molecular Biology and Genome Research, R. G. Lugar Center for Public Health Research, National Center for Disease Control and Public Health (NCDC) of Georgia.	Adam Kotorashvili; Amiran Gamkrelidze.; Ana Papkiauri; Ann Machablishvili; Anna Kasradze; Davit Tsaguria; Ekaterine Khmaladze; Ekaterine Zangaladze; Ekaterine Zhghenti; Giorgi Gogoladze; Giorgi Tomashvili; Gvantsa Brachveli; Gvantsa Chanturia; Irma Burjanadze; Ketevan Sidamonidze; Khatuna Zakhashvili; Lela Sabadze; Lela Urushadze; Magda Dgebuadze; Maia Alkhazashvili; Mari Gavashelidze; Mariam Zakalashvili; Marine Murtskhvaladze; Meri Pantsulaia; Nato Kotaria; Nino Berishvili; Paata Imnadze; Roena Sukhiashvili; Tamar Jashiashvili; Tata Imnadze; Tea Tevdoradze
EPI_ISL_2670493	Department of Bacteria, Parasites and Fungi, Statens Serum Institut, Copenhagen, Denmark	Statens Serum Institut Bioinformatics and Microbial Genomics	Danish Covid-19 Genome Consortium
EPI_ISL_515086	Department of Biochemistry, Cell and Molecular Biology	WACCBIP, University of Ghana	A.K.; Adu, B.; Amenga-Etego; Ampofo, W.; Amuzu; Anang; Arjarquah, A.; Asante, I.; Awandare; Bediako, Y.; Boatemaa, L.; Bonney, E.; Bonney, K.; C.M.; D.S.; Eshun, M.; G.A.; G.B.; J.K.; J.M.; Kotey, E.; Kumordjie, S.; Kyei; L.N.; Magnussen, V.; Morang'a; Mutungi; Ngoi; Quashie, P.; Tei-Maya, F.
EPI_ISL_884827	Department of Biochemistry, Cell and Molecular Biology, West African Centre for Cell Biology of Infectious Pathogens (WACCBIP), University of Ghana	Department of Biochemistry, Cell and Molecular Biology, West African Centre for Cell Biology of Infectious Pathogens (WACCBIP), University of Ghana	A.-K.; A.B.; Abass; Akoriyea; Amenga-Etego; Amoako, E.; Amuzu; Awandare; Bediako, Y.; Boakye; C.M.; D.S.; Diallo; G.A.; J.M.; Kibinge, N.; Kumi-Ansah, F.; L.N.; Magnussen, V.; Mohammed, A.; Morang'a; Ngoi; O.D.; Odoom, T.; Quashie, P.; S.K.; Tapela, K.; Tei-Maya, F.
EPI_ISL_540475	Department of Clinical Microbiology	GIGA Medical Genomics	Axelle Chaslain; Bouchra Boujemla; Cécile Meex; Céline Fombellida-Lopez; Keith Durkin; Maria Artesi; Marie-Pierre Hayette; Pierrette Melin; Raphaël Boreux; Sébastien Bontems; Vincent Bours
EPI_ISL_1870142	Department of Clinical Microbiology, Odense University Hospital, Odense, Denmark	Aalborg University	Danish Covid-19 Genome Consortium
EPI_ISL_2245688	Department of Experimental Modeling and Pathogenesis of Infectious Diseases	WHO National Influenza Centre Russian Federation	Alekseev A.Yu.; Andrey Komissarov; Anna Ivanova; Artem Fadeev; Chepurnov A.A.; Daria Danilenko; Dmitry Lioznov; Elena Nabieva; Georgii Bazykin; Kirill Varchenko; Kononova Yu.V.; Ksenia Safina; Kseniya Komissarova; Maria Pisareva; Maria Timofeeva; Mikhail Bakaev; Nikita Yolshin; Oula Masour; Shestopalov A.M.; Sobolev I.A.; Tamila Musaeva; Veronika Eder
EPI_ISL_1563631	Department of Genetic Engineering and Biotechnology, Shahjalal University of Science and Technology	Genomic Research Lab, BCSIR	Abu Sayeed Mohammad Mahmud; Ajit Ghosh; Barna Goswami; Eshrar Osman; G. M. Nurnabi Azad Jewel; Iffat Jahan; Md. Ahasan Habib; Md. Akkas Ali; Md. Asraful Jahan; Md. Fahmid Hossain Bhuiyan; Md. Hammadul Hoque; Md. Kamrul Islam; Md. Murshed Hasan Sarkar; Md. Nazmul Hasan; Md. Saddam Hossain; Md. Salim Khan; Md. Shamsul Haque Prodhan; Mohammad Mohi Uddin; Mohammad Samir Uzzaman; Shahina Akter; Tanjina Akhter Banu
EPI_ISL_1018303, EPI_ISL_1020208, EPI_ISL_2723768	Department of Health Technology and Informatics, The Hong Kong Polytechnic University	Department of Health Technology and Informatics, The Hong Kong Polytechnic University	Alan Ka-Lun Wu; Alex Yat-Man Ho; Barry Kin-Chung Wong; Chloe Toi-Mei Chan; David Ho-Keung Shum; Denise Sze-Hang Wong; Gilman Kit-Hang Siu; Hiu-Yin Lao; Hoi-Ching Jim; Ivan Tak-Fai Wong; Jake Siu-Lun Leung; Kam-Tong Yip; Kenneth Siu-Sing Leung; Kingsley King-Gee Tam; Kitty Sau-Chun Fung; Kristine Luk; Lam-Kwong Lee; Miranda Chong-Yee Yau; Sandy Ka-Yee Chau; Shea Ping Yip; Tak-Lun Que; Timothy Ting-Leung Ng; Wing Cheong Yam; Wing-Hei Lo; Wing-Kin To; Yvette Wai-Man Lai
EPI_ISL_1495326	Department of Laboratory Medicine, Division of Clinical Virology, University of Medicine, Vienna	Bergthaler laboratory, CeMM Research Center for Molecular Medicine of the Austrian Academy of Sciences	Andreas Bergthaler; Anna Schedl; Bekir Erguner; Benedikt Agerer; Christoph Bock; Fabian Amman; Jan Laine; Lukas Endler; Maelle Le Moing; Martin Senekowitsch; Michael Schuster; Petr Triska; Thomas Penz
EPI_ISL_538436, EPI_ISL_538456, EPI_ISL_538458	Department of Laboratory Medicine, Tan Tock Seng Hospital	Department of Laboratory Medicine, Tan Tock Seng Hospital	Barkham TMS; Chen YYC; Li C; Lim JX; Maurer-Stroh S; Nagarajan N; Sessions OM; Tang WY; Zair X
EPI_ISL_1192188	Department of Medical Microbiology, St. Olavs hospital	Norwegian Institute of Public Health, Department of Virology	Atiya R Ali; Debech Nadia; Engebretsen Serina Beate; Garcia Llorente Ignacio; Hilde Elshaug; Hilde Vollan; Kamilla Heddeland Instefjord; Karoline Bragstad; Kathrine Stene-Johansen; Marie Paulsen Madsen; Olav Hungnes; Pedersen Benedikte Nevjen; Rasmus Riis Kopperud
EPI_ISL_512844	Department of Medical Research	DMR_Myanmar	Aung Kyaw Kyaw; Aung Zaw Latt; Hlaing Myat Thu; Hnin Ohnmar Soe; Htin Lin; Kay Thi Aye; Lai Lai San; Myat Htut Nyunt; Nan Aye Thida Oo; Ni Ni Zaw; Phyu Win Ei; Su Mon Win; Theingi Win Myat; Wah Wah Aung; Yi Yi Kyaw; Zaw Than Htun
EPI_ISL_2547429	Department of Medical Virology, School of Medicine Ahvaz Jundishapur University of Medical sciences	Genetics Research Center, University of Social Welfare and Rehabilitation Sciences	Ahmad Tavakoli; Azarakhsh Azaran; Farid yousefi; Hossein Najmabadi.; Kimia Kahrizi; Maryam Beheshtian; Marzieh Mohseni; Mohammad Farahmand; Mohsen Savaie; Seyed Mohammad Jazayeri; Zohreh Fattahi
EPI_ISL_3066017	Department of Microbiology	Department of Microbiology	Paul K.S. Chan; Zigui Chen
EPI_ISL_1745697	Department of Microbiology, Faculty of Medicine and Dentistry, Palacky University and University Hospital Olomouc	Institute of Molecular and Translational Medicine / Laboratory of Experimental Medicine, Faculty of Medicine and Dentistry, Palacky University	Barbora Blumová; Hana Jaworek; Marián Hajdúch; Pavel Sauer; Rastislav Slavkovský; Tomáš Pospíšil; Vladimíra Koudeláková
EPI_ISL_2833646, EPI_ISL_2833648, EPI_ISL_2833655, EPI_ISL_2833665	Department of Microbiology, National Institute for Public Health of Kosova	Charité Universitätsmedizin Berlin, Institut für Virologie	Aferdita Hyseni; Barbara Mühlemann; Blendi Jerliu; Christian Drosten; Donjeta Hajdari; Julia Schneider; Jörn Beheim-Schwarzbach; Nazmi Mehmeti; Pranvera Abazi; Talitha Veith; Terry Jones; Victor M Corman; Xhevat Jakupi; Zana Deva
EPI_ISL_1164628, EPI_ISL_1164630, EPI_ISL_1164632, EPI_ISL_1164633, EPI_ISL_1164724	Department of Molecular Virology, Cyprus Institute of Neurology and Genetics	Department of Molecular Virology, Cyprus Institute of Neurology and Genetics	Anastasis Oulas; Andreas Hadjisavvas; Christina Christodoulou; Christina Tryfonos; Dana Koptides; Denise Alexandrou; George Krashias; George Spyrou; Jan Richter; Maria Loizidou; Mihalis Panayiotidis; Olga Kalakouta; Pavlos Fanis; Stavros Bashiardes
EPI_ISL_2622046	Department of Pathology	Centre for Research in Advanced Tropical Bioscience Universiti Malaysia Pahang, 26300 Gambang, Pahang, Malaysia	Hajar Fauzan Bin Ahmad; Norhidayah Binti Kamarudin; Ummu Afeera Zainulabid
EPI_ISL_439399	Department of Pathology, University of Cambridge	Wellcome Sanger Institute for the COVID-19 Genomics UK (COG-UK) consortium	Alex Alderton; Aminu S. Jahun; Anna Yakovleva; Charlotte J. Houldcroft; Cordelia Langford; David K. Jackson; Dominic Kwiatkowski; Ewan Harrison; Fahad A Khokhar; Grant Hall; Ian Goodfellow; Ian Johnston; John Sillitoe on behalf of the Wellcome Sanger Institute COVID-19 Surveillance Team (http://www.sanger.ac.uk/covid-team); Laura G Caller; Luke W Meredith; M. Estée Török; Martin D. Curran; Myra Hosmillo; Roberto Amato; Sarah L. Caddy; Sonia Goncalves; Theresa Feltwell; William L. Hamilton
EPI_ISL_1969990, EPI_ISL_2099884, EPI_ISL_3375697	Department of Public Health Bucharest	National Institute of Infectious Diseases-Prof. Dr. Matei Bals Molecular Diagnostics Laboratory	Andreea Tudor; Corina Casangiu; Dan Otelea; Leontina Banica; Marius Surleac; Ovidiu Vlaicu; Simona Paraschiv
EPI_ISL_2687992	Department of Public Health Constanta	National Institute of Infectious Diseases-Prof. Dr. Matei Bals Molecular Diagnostics Laboratory	Corina Casangiu; Dan Otelea; Leontina Banica; Marius Surleac; Petre Milu; Robert Hohan; Simona Paraschiv
EPI_ISL_1289875	Department of Public Health Microbiology Ljubljana, National Laboratory for Health, Environment and Food	Department of Public Health Microbiology Ljubljana, National Laboratory for Health, Environment and Food	José Gonçalves; Katarina Prosenc; Martin Bosilj; Metka Paragi; Tom Koritnik
EPI_ISL_582509	Department of Respiratory and other Viral Infections of L.V.Gromashevsky Institute of Epidemiology & Infectious Diseases NAMS of Ukraine	Department of Respiratory and other Viral Infections of L.V.Gromashevsky Institute of Epidemiology & Infectious Diseases NAMS of Ukraine, JSC "Farmak"	Alla Mironenko; Andriy Goy; Ihor Kravchuk; Larysa Radchenko; Ludmyla Bolotova; Nataliia Teteriuk
EPI_ISL_979973, EPI_ISL_1315427	Department of Respiratory and other Viral Infections of L.V.Gromashevsky Institute of Epidemiology & Infectious Diseases NAMS of Ukraine	Department of Respiratory and other Viral Infections of L.V.Gromashevsky Institute of Epidemiology & Infectious Diseases NAMS of Ukraine, JSC "Farmak"	Alla Mironenko; Andriy Goy; Ihor Kravchuk; Larysa Radchenko; Ludmyla Bolotova; Nataliia Teteriuk
EPI_ISL_654818	Department of Respiratory and other Viral Infections of L.V.Gromashevsky Institute of Epidemiology & Infectious Diseases NAMS of Ukraine	Department of Respiratory and other Viral Infections of L.V.Gromashevsky Institute of Epidemiology & Infectious Diseases NAMS of Ukraine, JSC "Farmak"	Alla Mironenko; Andriy Goy; Ihor Kravchuk; Larysa Radchenko; Ludmyla Bolotova; Nataliia Teteriuk
EPI_ISL_1385791, EPI_ISL_1969995, EPI_ISL_2434781, EPI_ISL_2438547, EPI_ISL_2894973	Department of Virology	Department of Virology	Aamer Ikram; Abdul Ahad; Austin Leach; Joel Montgomery; John Klena; Ketan Patel; Massab Umair; Melissa Mobley; Muhammad Ammar; Muhammad Salman; Nazish Badar; Qasim Ali; Sana Tamim; Shannon Whitmer; Syed Adnan Haider; Zaira Rehman

EPI_ISL_481681, EPI_ISL_862055, EPI_ISL_3014888, EPI_ISL_3015047	Department of Virology and Immunology, University of Helsinki and Helsinki University Hospital, Huslab Finland	Department of Virology, Faculty of Medicine, University of Helsinki, Helsinki, Finland	Essi Korhonen; Hanna Jarva; Hanna Liimatainen; Hannimari Kallio-Kokko; Harri Kangas; Hussein Alburkat; Jenni Virtanen; Maija Lappalainen; Maija Suvanto; Olli Vapalahti; Pekka Ellonen; Phuoc Truong; Ravi Kant; Sari Hannula; Satu Kurkela; Teemu Smura
EPI_ISL_855564, EPI_ISL_855571	Department of Virology, Principal Military Hospital of Instruction of Tunis	Bundeswehr Institute of Microbiology	Habiba Naija; Kilian Stoecker; Malena Bestehorn-Willmann; Markus H. Antwerpen; Mathias C. Walter; Roman Wölfel & Mohamed Ben Moussa; Simone Eckstein; Susann Handrick
EPI_ISL_1867529, EPI_ISL_2178060	Department of Virus and Microbiological Special Diagnostics, Statens Serum Institut, Copenhagen, Denmark	Aalborg University	Danish Covid-19 Genome Consortium
EPI_ISL_757470, EPI_ISL_757974	Department of Virus and Microbiological Special Diagnostics, Statens Serum Institut, Copenhagen, Denmark	Albertsen Lab, Department of Chemistry and Bioscience, Aalborg University, Denmark	Danish Covid-19 Genome Consortium
EPI_ISL_615676, EPI_ISL_618102, EPI_ISL_618354, EPI_ISL_618355, EPI_ISL_618356	Department of Virus and Microbiological Special Diagnostics, Statens Serum Institut, Denmark	Albertsen lab, Department of Chemistry and Bioscience, Aalborg University, Denmark	Danish Covid-19 Genome Consortia
EPI_ISL_2400054, EPI_ISL_2491413	Dept. of Medical Microbiology, Stavanger University Hospital, Helse Stavanger HF	Norwegian Institute of Public Health, Department of Virology	Atiya R Ali; Debech Nadia; Engebretsen Serina Beate; Garcia Llorente Ignacio; Hilde Elshaug; Hilde Vollan; Jon Bråte; Kamilla Heddeland Instefjord; Karoline Bragstad; Kathrine Stene-Johansen; Line Victoria Moen; Marie Paulsen Madsen; Olav Hungnes; Pedersen Benedikte Nevjen; Rasmus Riis Kopperud
EPI_ISL_2171041	Dhulikhel Hospital, Kathmandu University Hospital	Molecular and Genomics Research Lab, Dhulikhel Hospital, Kathmandu University Hospital	Dipesh Tamrakar; Manu Vanaerschot; Meghnath Dhimal; Navin Adhikari; Nishan Katuwal; Pradip Gyanwali; Rajeev Shrestha; Saroj Bhattarai; Surendra Kumar Madhup
EPI_ISL_2101017	Diagnostic Laboratory-Microbiology	Biomedical Laboratory-2	A. Dharshan De Silva; Dilini Nakkawita; Dumitha Govindapala; Harshi Abeygoonawardena; Himali S. Jayasinghearachchi; Pawani Gamage; Priyamali Jayasekera; Thamarasi Senaratne; Upeksha Kulasekera
EPI_ISL_1533077	Diagnostic and Research Center of Infectious Diseases, Medical Faculty, Andalas University	Diagnostic and Research Center of Infectious Diseases, Medical Faculty, Andalas University	Andani Eka Putra; Ayu Novita Trisnawati; Dede Rahman Agustian; Desmawati; Dessy Arisanty; Fauzul Azhim; Gestina Aliska; Ikhwan R. Sudji; Juane Plantika Menra; Linosefa; Mutia Lailani; Nia Ayuni Putri; Nita Afriani; SM Rezvi; Sekar Asri Tresnaningtyas; Siskalil Fahma; Syafrizayanti; Syandrez Prima Putra; Yolani Syaputri
EPI_ISL_2423458, EPI_ISL_2423485	Diagnostyka. Laboratoria Medyczne.	1. ViroGenetics - BSL3 Laboratory of Virology, Małopolska Centre of Biotechnology, Jagiellonian University; 2. Diagtron Laboratoria Lukasz Rabalski	Gromowski, T.; Kowalski, M.; Labaj; Maciej Kosinski; Mazur-Panasiuk, N.; Natalia Derewonko; P.P.; Pyrc, K.; Rabalski L.; Rogalska-Kupiec M.; Swadzba J.; Sylwia Januszczak; Szulc, P.; Wydmanski, W.
EPI_ISL_2230062	Dimedical	1. National Institute of Public Health - National Institute of Hygiene; 2. Eurofins Genomics Europe Sequencing GmbH	ECDC COVID-19 WGS support team; Eurofins Genomics Europe Sequencing Team; Gierczyńki Rafał; Sadkowska-Todys Małgorzata; Wołkowicz Tomasz; Zacharczuk Katarzyna
EPI_ISL_1007655, EPI_ISL_1936582, EPI_ISL_1936583, EPI_ISL_2161137, EPI_ISL_2361092, EPI_ISL_2361097, EPI_ISL_2361253, EPI_ISL_2710499			
see above	Division of Emerging Infectious Diseases, Bureau of Infectious Diseases Diagnosis Control, Korea Disease Control and Prevention Agency	Division of Emerging Infectious Diseases, Bureau of Infectious Diseases Diagnosis Control, Korea Disease Control and Prevention Agency	Ae Kyung Park; Chae Young Lee; Eun-Jin Kim; Heui Man Kim; Il-Hwan Kim; Jeong-Ah Kim; Jeong-Min Kim; Jin Sun No; Namjoo Lee; Sang Hee Woo
EPI_ISL_2695849, EPI_ISL_2695860, EPI_ISL_2695870, EPI_ISL_2695871, EPI_ISL_2695873, EPI_ISL_2695874	Division of Medical Virology, National Health Laboratory Service (NHLS), Tygerberg Hospital / Stellenbosch University	Division of Medical Virology, National Health Laboratory Service (NHLS), Tygerberg Hospital / Stellenbosch University	Bronwyn Kleinhans; Gert van Zyl; San Emmanual James; Susan Engelbrecht; Tania Stander; Tongai Maponga; Tulio de Oliveira; Wolfgang Preiser
EPI_ISL_1591434, EPI_ISL_3066391, EPI_ISL_3066514, EPI_ISL_3068096	Division of Medical Virology, National Health Laboratory Service (NHLS), Tygerberg Hospital / Stellenbosch University	Division of Medical Virology, Stellenbosch University and NHLS Tygerberg Hospital	Bronwyn Kleinhans; Gert van Zyl; Kayla Delaney; Menzi Nkosi; Susan Engelbrecht; Wolfgang Preiser
EPI_ISL_426187, EPI_ISL_497962	Division of Viral Diseases, Center for Laboratory Control of Infectious Diseases, Korea Centers for Diseases Control and Prevention	Division of Viral Diseases, Center for Laboratory Control of Infectious Diseases, Korea Centers for Diseases Control and Prevention	Heui Man Kim; Hye-Jun Jo; Jeong-Min Kim; Jun-Sub Kim; Junhyeong Jang; Mi-Seon Kim; Myung Guk Han; Namjoo Lee; Sang Hee Woo; Sehee Park; Yoon-Seok Chung
EPI_ISL_460681, EPI_ISL_523013, EPI_ISL_523755, EPI_ISL_801441, EPI_ISL_801442, EPI_ISL_2145550, EPI_ISL_2154193			
see above	Dutch COVID-19 response team	Erasmus Medical Center	Anne van der Linden; Annemiek van der Eijk; Aura Timen; Bas Oude Munnink; Claudia Schapendonk; Corien Swaan; Corine GeurtsvanKessel; David Nieuwenhuijse; Emmanuelle Munger; Irina Chestakova; Jeroen van Kampen; Jolanda Voermans; Madelief Mollers; Manon Haverkate; Marion Koopmans; Marjan Boter; Mark Pronk; Mart Stein; OH consortium; Pascal Lexmond; Reina Sikkema; Richard Molenkamp; Sandra Kengne Kamga Mobou; Stefan van Nieuwkoop; Theo Bestebroer; on behalf of the Dutch national COVID-19 respo; on behalf of the Dutch national COVID-19 response team.
EPI_ISL_547453, EPI_ISL_547478, EPI_ISL_943477, EPI_ISL_1014550, EPI_ISL_1232433, EPI_ISL_1595983, EPI_ISL_1704943, EPI_ISL_1705929, EPI_ISL_1706084, EPI_ISL_1792360, EPI_ISL_1792865, EPI_ISL_1792896, EPI_ISL_1962005, EPI_ISL_1962940, EPI_ISL_1962944, EPI_ISL_2093544, EPI_ISL_2093798, EPI_ISL_2094340, EPI_ISL_2094357, EPI_ISL_2094395, EPI_ISL_2094396, EPI_ISL_2302173, EPI_ISL_2302971, EPI_ISL_2303931, EPI_ISL_2303970, EPI_ISL_2303978, EPI_ISL_2405481, EPI_ISL_2405597, EPI_ISL_2405620, EPI_ISL_2476313, EPI_ISL_2609622, EPI_ISL_2610186			
see above	Dutch COVID-19 response team	National Institute for Public Health and the Environment (RIVM)	Adam Meijer; AnneMarie van den Brandt; Annelies Kroneman; Bas van der Veer; Chantal Reusken; Dennis Schmitz; Dirk Eggink; Eunice Then; Florian Zwagemaker; Harry Vennema; James Groot; Jeroen Cremer; Jolienke Hardeman; Karim Hajji; Kim Freriks; Linda van de Nes; Lisa Wijsman; Lynn Aarts; Melissa van Tuil; Robert Kohl; Ryanne Jaarsma; Sanne Bos; Sharon van den Brink; Sjoerd Kuiling; on behalf of the national COVID-19 response team
EPI_ISL_2621147	Dysselsdorp Clinic wc DDC	NHLS/UCT	Arash Iranzadeh; Bruna Galvao; Carolyn Williamson; Deelan Doolabh; Diana Hardie; Gert Marais; Innocent Mudau; Lynn Tyers; Marvin Hsiao; Stephen Korsman
EPI_ISL_2230399	Dział Diagnostyki Laboratoryjnej SPZOZ	1. National Institute of Public Health - National Institute of Hygiene; 2. Eurofins Genomics Europe Sequencing GmbH	ECDC COVID-19 WGS support team; Eurofins Genomics Europe Sequencing Team; Gierczyńki Rafał; Sadkowska-Todys Małgorzata; Wołkowicz Tomasz; Zacharczuk Katarzyna
EPI_ISL_512313	E. Gulbja Laboratorija	Latvian Biomedical Research and Study Centre	Dmitrijs Perminovs; Ivars Silamiķelis; Jānis Kloviņš; Kaspars Megnis; Mikus Gavars; Monta Ustinova; Uga Dumpis; Vita Rovīte; Ņikita Zrelovs
EPI_ISL_1312414	E. Gulbja laboratorija	Latvian Biomedical Research and Study Centre	Davids Fridmanis; Dmitrijs Perminovs; Guntars Zarins; Ivars Silamikelis; Janis Klovins; Janis Pjalkovskis; Jurijs Perevoscikovs; Kaspars Megnis; Laila Silamikele; Lauma Freimane; Laura Ansone; Liga Birzniece; Mikus Gavars; Monta Ustinova; Nikita Zrelovs; Uga Dumpis; Una Krumina; Vita Rovite
EPI_ISL_1696047	E.F.S B.F.C JEAN MINJOZ	Department of Virology, Henri Mondor University Hospital, Assistance Publique Hôpitaux de Paris, Université Paris-Est Créteil, INSERM U955	Alexandre Soulier; Christophe Rodriguez; Elisabeth Trawinski; Guillaume Gricourt; Jean-Michel Pawlotsky; Melissa N'Debi; Slim Fourati; Vanessa Demontant
EPI_ISL_2359398	EHNV	Laboratory of genomics and metagenomics	Claire Bertelli; Damien Jacot; Gilbert Greub; Sébastien Aeby; Trestan Pillonel
EPI_ISL_2370853	ETHNIKO KENTRO AIMODOSIAS E.KE.A.	Greek Genome Center, Biomedical Research Foundation of the Academy of Athens (BRFAA)	Dimitrios Thanos; Efthimia Petinaki; Emmanouil Athanasiadis; Giannis Vatsellas; Katerina Zoi; Kostas Stamoulis; Theodoros Loupis
EPI_ISL_2447785	East London Laboratory	National Institute for Communicable Diseases of the National Health Laboratory Service	Amoako DG; Bhiman JN; Ismail A; Mahlangu B; Mohale T; Ntuli N; Scheepers C
EPI_ISL_2479322, EPI_ISL_2940897, EPI_ISL_2941304	Edmonton Provincial Lab	Public Health Agency of Canada (PHAC) National Microbiology Laboratory	Buss; Croxen M; Deo A; Dieu P; E; Ferrato C; Gill K; Khan F; Koleva P; Li V; Lloyd C; Lynch T; Ma R; Murphy S; Pabbaraju K; Shokoples S; Thayer J; Tipples G; Whitehouse M; Wong A; Yu C; Zelyas N
EPI_ISL_2029260	Ekstralab Tuzla	Alea Genetic Centre	Adis Kandic; Dino Pecar; Enis Kandic; Lana Salihefendic; Nusret Butkovic; Rijad Konjhodzic
EPI_ISL_2086267	Enthabeni Lancet Laboratories	KRISP, KZN Research Innovation and Sequencing Platform	Emmanuel SJ; Giandhari J; Khan S; Lessells R; Mdlalose K; Naidoo Y; Pillay S; Ramphal U; Tegally H; Wilkinson E; York D; de Oliveira T
EPI_ISL_640064	Ethembeni Clinic [Prieska]	NHLS/UCT	Arash Iranzadeh; Bruna Galvao; Carolyn Williamson; Deelan Doolabh; Diana Hardie; Innocent Mudau; Kruger Marais; Lynn Tyers; Marvin Hsiao; Stephen Korsman
EPI_ISL_1897644, EPI_ISL_1897648, EPI_ISL_1897884, EPI_ISL_2241494, EPI_ISL_2241622, EPI_ISL_2241623	Ethiopian Biotechnology Institute (EBTI)	International Centre for Genetic Engineering and Biotechnology (ICGEB) and ARGO Open Lab for Genome Sequencing	Alessandro Marcello; Danilo Licastro; Emanuele Orsini; Getnet Hailu; Hailu Dadi; Kassahun Tesfaye; Keyru Tuki; Kominist Asmamaw; Molalegne Bitew; Simeone Dal Monego; Yakob Gebregziabher Tsegay
EPI_ISL_1712273	FSBI «NATIONAL MEDICAL RESEARCH CENTER FOR OBSTETRICS, GYNECOLOGY AND PERINATOLOGY NAMED AFTER ACADEMICIAN V.I.KULAKOV» MINISTRY OF HEALTHCARE OF THE RUSSIAN FEDERATION	Center for Precision Genome Editing and Genetic Technologies for Biomedicine, Pirogov Medical University, Moscow, Russian Federation	Anastasia Shut; Andrey Krivoy; Dmitriy Korostin; Margarita Korzhanova; Vera Belova; Yegor Botsmanov

EPI_ISL_1273098	Faculty of Medicine, Al-Quds University	Faculty of Medicine, Al-Quds University	Al-Jawabreh, A.; Dumaidi, K.; Ereqat, S.; Nasereddin, A.
EPI_ISL_2382403	FarmaLab Indoutama Halim	National Institute of Health Research and Development	Arie Ardiansyah Nugraha; Hana Apsari Pawestri; Hartanti Dian Ikawati; Kartika Dewi Puspa; Krisna Pangesti; Nelly Puspandari; Subangkit; Triyani Soekarso; Vivi Setiawaty
EPI_ISL_451966	Federal Budget Institution of Science, State Research Center for Applied Microbiology & Biotechnology	Federal Budget Institution of Science, State Research Center for Applied Microbiology & Biotechnology	Abaimova A; Bakhteeva I; Blagodatskikh S; Bogun A; Borzilov A; Chekan L; Chernysh S; Denisenko E; Dentovskaya S; Detushev K; Detusheva E; Dyatlov I; Firstova V; Frolov V; Fursov M; Fursova N; Galkina E; Gapelchenkova T; Goncharova J; Gorbatov A; Hlyntseva A; Ivanov S; Kalmantayev T; Kalmantayeva O; Kanashenko M; Kartsev N; Kartseva A; Khomyakov A; Khramov M; Kislichkina A; Kolchanova A; Koroleva-Ushakova A; Kosilova I; Krasilnikova E; Kuzin V; Kuzina E; Makarova M; Marin M; Novikova T; Platonov M; Podkopaev Y; Ryabko A; Shaikhutdinova R; Shemyakin I; Shishkina L; Silkina M; Sizova A; Skryabin Y; Slukin P; Slukina N; Solomentsev V; Solovieva A; Teymurazov M; Timofeev V; Titareva G; Trunyakova A; Tyurin E; Vagayskaya A; Zeninskaya N; Zhumakaev R
EPI_ISL_2508043	Fimlab Laboratoriot Oy Tampere	Expert Microbiology, National Institute for Health and Welfare	Carita Savolainen-Kopra; Erika Lindh; Haider al-Hello; Jani Halkilahti; Kirsi Liitsola; Niina Ikonen; Olli Vapalahti; Pekka Ellonen; Phuoc Truong; Päivi Laurila; Ravi Kant; Sari Hannula; Soile Blomqvist; Teemu Smura
EPI_ISL_2860230	First Aide Diagnostic Center	Philippine Genome Center	Alethea R. de Guzman; Anna Ong-Lim; Arianne A. Zamora; Benedict A. Maralit; Carlo M. Lapid; Celia Carlos; Devon Ray Pacial; Diomedes A. Carino; Edsel Maurice Salvaña; El King D. Morado; Elcid Aaron R. Pangilinan; Eva Maria Cutiongco-de la Paz; Francis A. Tablizo; Henrietta Marie Rodriguez; Jaime C. Montoya; Jan Michael C. Yap; Jarvin E. Nipales; Jo-Hannah S. Llames; John Q. Wong; Joshua Gregor A. Dizon; Juan Antonio R. Magalang; Karol Sophia Agape R. Padilla; Kenneth M. Kim; Kris P. Punayan; Krisitna Patriz Dela Cruz; Lindsay Claire D.L. Carandang; Ma. Exanil Plantig; Marc Edsel C. Ayes; Maria Rosario Singh-Vergeire and Cynthia P. Saloma; Maria Sofia L. Yangzon; Marielle M Gamboa; Marissa Alejandria; Nina Francesca Bustamante; Razel Nikka M. Hao; Renato Jacinto Q. Mantaring; Rianna Patricia S. Cruz; Sheila Mae M. Araiza; Yvonne Valerie Austria; Zipporah Mariebelle R. Enriquez; Zyrel V. Mollejon
EPI_ISL_2889849, EPI_ISL_2889850	Fondation Congolaise pour la recherche medicale (FCRM)	Fondation Congolaise pour la Recherche Médicale	Abel Lissom; Batchi-Bouyou Armel Landry; Francine Ntoumi; Jean Claude Djontu; Mfoutou Mapanguy Claujens Chastel; Thirumalaisamy P. Velavan
EPI_ISL_581487	Fondation Congolaise pour la recherche medicale (FCRM)	NGS Competence Center Tübingen, Institut für Medizinische Mikrobiologie und Hygiene, Universitätsklinikum Tübingen	Angel Angelov
EPI_ISL_3342566, EPI_ISL_3342568	Fondation Congolaise pour la recherche medicale (FCRM), Francine Ntoumi	Fondation Congolaise pour la Recherche Médicale	Batchi-Bouyou Armel Landry; Dr. Abel Lissom; Dr. Jean Claude Djontu; Mfoutou Mapanguy Claujens Chastel; Prof. Dr. Thirumalaisamy P. Velavan; Prof. Francine Ntoumi
EPI_ISL_1854772	Fondation Congolaise pour la recherche medicale (FCRM), Francine Ntoumi	Institute of Tropical Medicine	Mfoutou Mapanguy Claujeans Chastel and Batchi-Bouyou Armel Landry; Prof. Dr. Thirumalaisamy P. Velavan; Prof. Francine Ntoumi
EPI_ISL_912365	Fondation Congolaise pour la recherche medicale (FCRM), Francine Ntoumi	NGS Competence Center Tuebingen, Institut für Medizinische Mikrobiologie und Hygiene, Universitaetsklinikum Tübingen	Angel Angelov
EPI_ISL_2620946	Fondazione IRCCS Ca' Granda Ospedale Maggiore Policlinico	Fondazione IRCCS Ca' Granda Ospedale Maggiore Policlinico	Ferruccio Ceriotti; Sara Uceda Renteria
EPI_ISL_1469585	Fundação Hospitalar de Sapucaia do Sul	Epiclin	Ana Paula Muterle; Carolina Comerlato; Eliana Márcia Da Ros Wendland; Fernando Hayashi Sant'Anna; Janira Prichula; Juliana Comerlato
EPI_ISL_493369, EPI_ISL_1828957, EPI_ISL_1828958	Furst Medical Laboratory	Norwegian Institute of Public Health, Department of Virology	Atiya R Ali; Debech Nadia; Engebretsen Serina Beate; Garcia Llorente Ignacio; Hilde Elshaug; Hilde Vollan; Jon Bråte; Kamilla Heddeland Instefjord; Karoline Bragstad; Kathrine Stene-Johansen; Marie Paulsen Madsen; Olav Hungnes; Pedersen Benedikte Nevjen; Rasmus Riis Kopperud
EPI_ISL_1110194, EPI_ISL_1110195, EPI_ISL_1110196, EPI_ISL_1110197, EPI_ISL_1110198, EPI_ISL_1672700, EPI_ISL_1672744, EPI_ISL_1672745, EPI_ISL_1672746, EPI_ISL_1672747, EPI_ISL_1672748, EPI_ISL_1672749, EPI_ISL_1706618, EPI_ISL_1706658, EPI_ISL_1706661			
see above	G.H.E.F.Grand Hôpital EST Francilien	Department of Virology, Henri Mondor University Hospital, Assistance Publique Hôpitaux de Paris, Université Paris-Est Créteil, INSERM U955	Alexandre Soulier; Christophe Rodriguez; Elisabeth Trawinski; Guillaume Gricourt; Jean-Michel Pawlotsky; Melissa N'Debi; Slim Fourati; Vanessa Demontant
EPI_ISL_1672460, EPI_ISL_1672547, EPI_ISL_1755046, EPI_ISL_2179448, EPI_ISL_2259268	GH A.CHENEVIER-H.MONDOR	Department of Virology, Henri Mondor University Hospital, Assistance Publique Hôpitaux de Paris, Université Paris-Est Créteil, INSERM U955	Alexandre Soulier; Christophe Rodriguez; Elisabeth Trawinski; Guillaume Gricourt; Jean-Michel Pawlotsky; Melissa N'Debi; Slim Fourati; Vanessa Demontant
EPI_ISL_1672499	GH JOFFRE DUPUYTREN	Department of Virology, Henri Mondor University Hospital, Assistance Publique Hôpitaux de Paris, Université Paris-Est Créteil, INSERM U955	Alexandre Soulier; Christophe Rodriguez; Elisabeth Trawinski; Guillaume Gricourt; Jean-Michel Pawlotsky; Melissa N'Debi; Slim Fourati; Vanessa Demontant
EPI_ISL_2179373, EPI_ISL_2179568, EPI_ISL_2179569, EPI_ISL_2179576	GH de l'Est Francilien	Department of Virology, Henri Mondor University Hospital, Assistance Publique Hôpitaux de Paris, Université Paris-Est Créteil, INSERM U955	Alexandre Soulier; Christophe Rodriguez; Elisabeth Trawinski; Guillaume Gricourt; Jean-Michel Pawlotsky; Melissa N'Debi; Slim Fourati; Vanessa Demontant
EPI_ISL_2779303	GK Prison Busia (Matayo)Matayos Prison	USAMRD-A, Basic Science Laboratory	Alan Lemtudo; Beth Mutai; Brian Andika; Carol Kifude; Clement Masakwe; Eric Muthanje; Esther Omuseni; Faith Sigei; Gathii Kimita; George Awinda; John Waitumbi; Josphat Nyataya; Rachel Githii; Rehema Liyai; Stephen Ochola
EPI_ISL_2779388	GK Prison Kiambu	USAMRD-A, Basic Science Laboratory	Alan Lemtudo; Beth Mutai; Brian Andika; Carol Kifude; Clement Masakwe; Eric Muthanje; Esther Omuseni; Faith Sigei; Gathii Kimita; George Awinda; John Waitumbi; Josphat Nyataya; Rachel Githii; Rehema Liyai; Stephen Ochola
EPI_ISL_2779315	GK Prisons Dispensary Kapsabet	USAMRD-A, Basic Science Laboratory	Alan Lemtudo; Beth Mutai; Brian Andika; Carol Kifude; Clement Masakwe; Eric Muthanje; Esther Omuseni; Faith Sigei; Gathii Kimita; George Awinda; John Waitumbi; Josphat Nyataya; Rachel Githii; Rehema Liyai; Stephen Ochola
EPI_ISL_2547341	Garies PHC Clinic	National Health Laboratory Service/University of Cape Town (NHLS/UCT)	Arash Iranzadeh; Bruna Galvao; Carolyn Williamson; Deelan Doolabh; Diana Hardie; Gert Marais; Innocent Mudau; Lynn Tyers; Marvin Hsiao; Stephen Korsman
EPI_ISL_730570, EPI_ISL_984745, EPI_ISL_984749	Gazi University Faculty of Medicine, Medical Virology Laboratory	Gazi University Faculty of Medicine, Medical Virology Laboratory	Erdem Şahin; Gülendam Bozdayı; Hager Muftah; Işıl Fidan; Kayhan Çağlar; Murat Dizbay; Selin Yiğit; Shaknoza Sarzhanova; Özlem Güzel Tunçcan
EPI_ISL_2375920	Genadendal Clinic wc GDC	NHLS/UCT	Arash Iranzadeh; Bruna Galvao; Carolyn Williamson; Deelan Doolabh; Diana Hardie; Innocent Mudau; Kruger Marais; Lynn Tyers; Marvin Hsiao; Stephen Korsman
EPI_ISL_2681439	General Hospital "8th of September"	Laboratory of virology and molecular diagnostics, Institute of Public Health	Boshevska G; Janchevska E.; Kuzmanovska M
EPI_ISL_922076	General Hospital #Abdulah Nakas" Sarajevo	Alea Genetic Centre	Dino Pecar; Lana Salihefendic; Rijad Konjhodzic; Sead Jazic
EPI_ISL_677707, EPI_ISL_678252	General Hospital - Kumanovo	Research Center for Genetic Engineering and Biotechnology "Georgi D. Efremov" , Macedonian Academy of Sciences and Arts	RCGEB - MASA
EPI_ISL_1971036	General Hospital - Struga	Research Center for Genetic Engineering and Biotechnology "Georgi D. Efremov" , Macedonian Academy of Sciences and Arts	Aleksandar J. Dimovski; Dijana Plasheska-Karanfilska; Gjorgji Bozinovski; Milena Jakimovska; Predrag Noveski
EPI_ISL_406798	General Hospital of Central Theater Command of People's Liberation Army of China	BGI & Institute of Microbiology, Chinese Academy of Sciences & Shandong First Medical University & Shandong Academy of Medical Sciences & General Hospital of Central Theater Command of People's Liberation Army of China	Weifeng Shi and Zhenhong Hu; Weijun Chen; Yuhai Bi
EPI_ISL_1167794, EPI_ISL_1167905, EPI_ISL_2009586, EPI_ISL_2391269, EPI_ISL_2543479, EPI_ISL_2597257	Genetica Molecular and Subdepartamento de Virologia ISP Chile	Instituto de Salud Publica de Chile	Andres Castillo; Barbara Parra; Constanza Campano; Gisselle Barra; Jaime Lagos; Javier Tognarelli; Jorge Fernandez; Karen Orostica; Loredana Arata; Patricia Bustos; Rodrigo Fasce; Soledad Ulloa
EPI_ISL_735275, EPI_ISL_735312	Genomic Laboratory (GLAB) (Conjoint lab of Health Directorate of Istanbul and Istanbul Technical University)	Genomic Laboratory (GLAB), Istanbul Technical University	Arzu Irvem; Ayse Serra Ozel; Betsi Kose; Gizem Alkurt; Gizem Dinler Doganay; Ilker Karacan; Jale Yildiz; Levent Doganay; Mehtap Aydin; Nihat Bugra Agaoglu; Nilsun Altunal; Nisan Denizce Can; Ozlem Akgun Dogan; Payam Zolfagharian; Tugba Kizilboga Akgun; Yasemin Kendir Demirkol
EPI_ISL_812784, EPI_ISL_2566467, EPI_ISL_2566468, EPI_ISL_2566470, EPI_ISL_2566473, EPI_ISL_2566474, EPI_ISL_2566477, EPI_ISL_2566478, EPI_ISL_2566481, EPI_ISL_2566485, EPI_ISL_2566486, EPI_ISL_2566490, EPI_ISL_2566506, EPI_ISL_2566507, EPI_ISL_2566509, EPI_ISL_2566510, EPI_ISL_2566512, EPI_ISL_2566513, EPI_ISL_2566514			
see above	Genomics Program, Children Cancer Hospital	Genomics Program, Children Cancer Hospital	Abdelaziz, H.; Abdo, I.; Abouelnaga, S.; Almeldin, A.; Amer, K.; Bakry, U.; Diab, A.; El-Shaqnqery, H.; El-Zayat, M.; ElHaddad, A.; ElHalafawy, A.; Elnaqeeb, M.; Farawela, H.; Farawyla, H.; Gomaa, C.; Hadad, A.; Halafawy, A.; Hammad, M.; Hassan, R.; Hassan, W.; Hatem, A.; Hossam, M.; Hussein, S.; Ismail, J.; Jalal, D.; Magdeldin, S.; Mansour, T.; Monuir, G.; Saaid, M.; Said, D.; Salah, H.; Samir, O.; Sayed, A.; Shalaby, L.; Soliman, M.; Soliman, S.; Yahia, A.; shalaby, L.
EPI_ISL_2434161	Genomik Solidaritas Indonesia Laboratorium	Genomik Solidaritas Indonesia Laboratorium	Alfin Mohammad Abdillah; Anindya Pradipta Susanto; Annisa Muthiah Sukirman; Ariel Pradipta; Carissa Sintca Wijaya; Dhahlia Agustina Cahyono; Gracia Felias Enos Korompis; Meutia Ayuputeri Kumaheri; Normastuti Adhini Tantyo; Vania Gavrila Wikasa
EPI_ISL_2140696	George Hospital wc GRH	NHLS/UCT	Arash Iranzadeh; Bruna Galvao; Carolyn Williamson; Deelan Doolabh; Diana Hardie; Innocent Mudau; Kruger Marais; Lynn Tyers; Marvin Hsiao; Stephen Korsman
EPI_ISL_2340207, EPI_ISL_2340210	Germano de Sousa	Instituto Nacional de Saude (INSA)	Borges et al
EPI_ISL_1225490, EPI_ISL_1225510, EPI_ISL_1225513, EPI_ISL_1225563, EPI_ISL_1503116	Gorgas Memorial Laboratory of Health Studies	Gorgas Memorial Laboratory of Health Studies	Adriana Weeden; Alejandra Valoy; Alexander Martinez; Ambar Moreno; Anyuri Ortiz; Brechla Moreno; Castillo Jorge; Claudia Gonzalez; Daniel Castillo; Danilo Franco; Davis Beltran; Dimelza Arauz; Elimelec Valdespino; Franco Danilo; Gonzalez Claudia; Gretel Vasquez; Ilka Guerra; Isela Guerrero; Jessica Gondola; Jim Chang; Juan Miguel Pascale; Layda Abrego; Leyda Abrego; Lisseth Saenz; Lopez-Verges Sandra; Mabel Martinez-Montero; Maria Chen-German; Marlene Castillo; Marlenne Castillo; Martinez Alexander; Melissa Gaitan; Moreno Ambar; Moreno Brechla; Oris Chavarria; Ortiz Alma; Rita Corrales; Rita Rodriguez; Sandra Lopez-Verges; Yamilka Diaz; Yaneth Pitti; Zumara Chaverra
EPI_ISL_2376850	Greek Genome Center, Biomedical	Greek Genome Center, Biomedical Research Foundation of the	Dimitrios Thanos; Emmanouil Athanasiadis; Giannis Vatsellas; Katerina Zoi; Theodoros Loupis

	Research Foundation of the Academy of Athens (BRFAA)	Academy of Athens (BRFAA)	
EPI_ISL_2301819, EPI_ISL_2369746, EPI_ISL_2371337	Greek Genome Center, Biomedical Research Foundation of the Academy of Athens (BRFAA)	Greek Genome Center, Biomedical Research Foundation of the Academy of Athens (BRFAA)	Dimitrios Thanos; Emmanouil Athanasiadis; Giannis Vatsellas; Katerina Zoi; Theodoros Loupis
EPI_ISL_3207493	Groote Schuur Hospital wc GSH	NHLS/UCT	Arash Iranzadeh; Bruna Galvao; Carolyn Williamson; Deelan Doolabh; Diana Hardie; Gert Marais; Innocent Mudau; Lynn Tyers; Marvin Hsiao; Rageema Joseph; Sisonke; Stephen Korsman
EPI_ISL_699085	Group 42 (G42) Healthcare, Abu Dhabi, United Arab Emirates; Department of Health, The United Arab Emirates	G42 Healthcare	Ashish Koshy; Budoor Alqarni; Denghui Liu; Fang Chen; Hanif Khalak; Huanming Yang; Javier Quilez; Jian Wang; Junhua Li; Ke Liang; Long Lin; Mohammed Saifuddin Fasihuddin; Nan Qiao; Nawal Ahmed Mohamed Al Kaabi; Pauline Ogrodzki; Pei Wu; Peng Xiao; Pengjuan Liu; Rong Liu; Sally Mahmoud; Siyang Liu; Stephen S. Francis; Tao Ma; Vinay Kusuma; Walid Abbas Zaher; Weibin Liu; Wenjun He; Xavier Anton; Xin Jin; Xin Meng; Xinyu Huang; Xun Xu; Zhaorong Yuan
EPI_ISL_1696300, EPI_ISL_1696301	Groupe LCD	Department of Virology, Henri Mondor University Hospital, Assistance Publique Hôpitaux de Paris, Université Paris-Est Créteil, INSERM U955	Alexandre Soulier; Christophe Rodriguez; Elisabeth Trawinski; Guillaume Gricourt; Jean-Michel Pawlotsky; Melissa N'Debi; Slim Fourati; Vanessa Demontant
EPI_ISL_509701	Guatemala Ministry of Public Health	Pathogen Discovery, Respiratory Viruses Branch, Division of Viral Diseases, Centers for Disease Control and Prevention	Anna Uehara; Clinton Paden; Haibin Wang; Jing Zhang; Krista Queen; Suxiang Tong; Yan Li; Ying Tao
EPI_ISL_511406	H Braga	Instituto Nacional de Saude (INSA)	Borges et al
EPI_ISL_1853561	H Fernando Fonseca	Instituto Nacional de Saude (INSA) and Institute of Biomedicine (iBiMed), Universidade de Aveiro	Borges et al
EPI_ISL_1854262	H Forcas Armadas - Polo Lisboa	Instituto Nacional de Saude (INSA) and Institute of Biomedicine (iBiMed), Universidade de Aveiro	Borges et al
EPI_ISL_602380, EPI_ISL_733035, EPI_ISL_733046, EPI_ISL_1491600	HELIX LLC	WHO National Influenza Centre Russian Federation	Alexey Masharsky; Andrey Komissarov; Anna Ivanova; Artem Fadeev; Daria Danilenko; Dmitry Bazhenov; Dmitry Lioznov; Elena Nabieva; Georgii Bazykin; Ksenia Safina; Kseniya Komissarova; Maria Baturova; Maria Pisareva; Maria Timofeeva; Tamila Musaeva; Veronika Eder
EPI_ISL_1965466, EPI_ISL_1965470	HG Pharma GmbH	Bergthaler laboratory, CeMM Research Center for Molecular Medicine of the Austrian Academy of Sciences	Andreas Bergthaler; Anna Schedl; Bekir Erguner; Benedikt Agerer; Christoph Bock; Fabian Amman; Jan Laine; Lukas Endler; Maelle Le Moing; Martin Senekowitsch; Michael Schuster; Petr Triska; Thomas Penz
EPI_ISL_2484793	HOME QUARANTINE TASKFORCE	Hong Kong Department of Health	Alan K.L. Tsang; Dominic N.C. Tsang; Edman T.K. Lam; Gannon C.K. Mak; Ken H.L. Ng; Peter C.W. Yip; Peter K.C. Cheng; Rickjason C.W. Chan
EPI_ISL_1731504	HOPITAL DU MONT DORE	CHU Clermont-Ferrand, service de virologie	Bisseux Maxime; Combes Patricia; Henquell Cécile; Mirand Audrey
EPI_ISL_1239447	HOPITAL MARIN HENDAYE	CNR Virus des Infections Respiratoires - France SUD	Antonin Bal; Bruno Lina; Gregory Destras; Gwendolyne Burfin; Hadrien Regue; Laurence Josset; Martine Valette; Quentin Semanas
EPI_ISL_2209295	HOSP MUN DE MOGI DAS CRUZES PREF WALDEMAR COSTA FILHO	Instituto Butantan / Mendelics	Antonio Jorge Martins; Claudia Renata dos Santos Barros; David Schlesinger; Debora Botequio Moretti; Dimas Tadeu Covas; Elaine Cristina Marqueze; Elaine Vieira Santos; Evandra Strazza Rodrigues; Heidge Fukumasu; Jayme Augusto de Souza-Neto; José Salvatore Leister Patané; Luiz Alcantara; Luiz Lehmann Coutinho; Maria Carolina Elias; Maurício Lacerda Nogueira; Rafael dos Santos Bezerra; Raul Machado Neto; Rejane Maria Tommasini Grotto; Ricardo Haddad; Sandra Coccuzzo Sampaio Vessoni; Simone Kashima; Svetoslav Nanev Slavov; Vincent Louis Viala
EPI_ISL_1712401	HOSPITAL CIUDAD NEILY	Inciensa, Instituto Costarricense de Investigación y Enseñanza en Nutrición y Salud	Adriana Godínez; Claudio Soto-Garita; Estela Cordero; Francisco Duarte; Hebleen Porras; Joselyn Prado & Raúl Zeledón-Mayorga; José Luis Vargas; Mariela Gutiérrez; Melany Calderón
EPI_ISL_1067604	HOSPITAL SAN JUAN DE DIOS	Inciensa, Instituto Costarricense de Investigación y Enseñanza en Nutrición y Salud	Adriana Godínez; Claudio Soto-Garita; Estela Cordero; Francisco Duarte; Hebleen Porras; Melany Calderón & Daniel Cascante-Serrano
EPI_ISL_1510697	HOSPITAL UNIVERSITARIO INSULAR DE GRAN CANARIA	Instituto de Salud Carlos III	A. Monzón; CRISTOBAL; F. Casas; I. Jiménez; I.DEL ROSARIO QUINTANA; M. Sandonís; P. Zaballos; S. Cuesta; S. Iglesias-Caballero; S. Pozo; S. Varona; V. Camarero; Vázquez-Morón
EPI_ISL_794659	HOSPITAL UNIVERSITARIO SAN IGNACIO	Instituto Nacional de Salud - Dirección de Investigación en Salud Pública	Carlos Franco-Muñoz; Diego A. Álvarez-Díaz; Diego Andrés Prada; Gerardo Santamaría; Jonathan Reales; Julian Naizaque; Katherine Laiton-Donato; Magdalena Wiesner; Marcela Mercado-Reyes; Maria T. Herrera; Martha Lucia Ospina Martinez; Mauricio Pacheco-Montealegre; Paola Muñoz-Laiton; Sheryl Corchuelo
EPI_ISL_2193525	Hackensack Medical Center	New York Genome Center	Andre Corvelo; Barry Kreiswirth; David Perlin; Dayna M. Oschwald; Jose Mediavilla; Kaelea Composto; Kar Chow; Liang Chen; Marcus Cunningham; Michael Zody; Samantha Fennessey; Tom Maniatis
EPI_ISL_700473	Heideveld CDC wc HVP	NHLS/UCT	Arash Iranzadeh; Bruna Galvao; Carolyn Williamson; Deelan Doolabh; Diana Hardie; Innocent Mudau; Kruger Marais; Lynn Tyers; Marvin Hsiao; Stephen Korsman
EPI_ISL_960153	Heideveld Emergency Centre	National Health Laboratory Service/UCT	Arash Iranzadeh; Bruna Galvao; Carolyn Williamson; Deelan Doolabh; Diana Hardie; Innocent Mudau; Kruger Marais; Lynn Tyers; Marvin Hsiao; Stephen Korsman
EPI_ISL_906711, EPI_ISL_906752	Hematology Laboratory, Section of Molecular Diagnostics, University Clinical Centre, Medical University of Gdansk	Laboratory of Recombinant Vaccines	Adam Sodol; Aneta Szulc; Anna Piotrowska-Mietelska; Boguslaw Szewczyk; Ewa Milosz; Izabela Szczygielska; Krystyna Bienkowska-Szewczyk; Krzysztof Lewandowski; Lukasz Rabalski; Maciej Grzybek; Maciej Kosinski; Marlena Robakowska
EPI_ISL_2678300	HerpeZ	Institute of Tropical Medicine	Alimuddin Zumla; Edgar Simulundu; Franklyn Egbe Nkongho; John Tembo; Kangwa Mulonga; Kwitaka Maluzi; Le Thi Kieu Linh; Lloyd Mulenga; Matthew Bates; Moses Chilufya; Nathan Kapata; Prof. Dr. Thirumalaisamy P Velavan; Sivaramakrishna Rachakonda; Sombo Fwoloshi; Srinivas Reddy Pallerla; Victor Mukonka
EPI_ISL_770024	Hle - Asociacion Hogar De Ancianos Santiago Crespo Calvo	Inciensa, Instituto Costarricense de Investigación y Enseñanza en Nutrición y Salud	Adriana Godínez; Claudio Soto-Garita; Estela Cordero; Francisco Duarte; Hebleen Porras; Melany Calderón & Mariel López
EPI_ISL_2779330, EPI_ISL_2779346	Homabay County Referral Hospital	USAMRD-A, Basic Science Laboratory	Alan Lemtudo; Beth Mutai; Brian Andika; Carol Kifude; Clement Masakwe; Eric Muthanje; Esther Omuseni; Faith Sigei; Gathii Kimita; George Awinda; John Waitumbi; Josphat Nyataya; Rachel Githii; Rehema Liyai; Stephen Ochola
EPI_ISL_1196003, EPI_ISL_1196011	Homecare	National Institute for Communicable Diseases of the National Health Laboratory Service	Amoako DG; Bhiman JN; Ismail A; Mahlangu B; Maphalala GP; Mohale T; Ntuli N; Scheepers C
EPI_ISL_2547352	Hondeklipbaai Clinic	National Health Laboratory Service/University of Cape Town (NHLS/UCT)	Arash Iranzadeh; Bruna Galvao; Carolyn Williamson; Deelan Doolabh; Diana Hardie; Gert Marais; Innocent Mudau; Lynn Tyers; Marvin Hsiao; Stephen Korsman
EPI_ISL_1111061, EPI_ISL_1118883, EPI_ISL_1118898, EPI_ISL_1118899, EPI_ISL_1239371, EPI_ISL_1259284, EPI_ISL_1259293, EPI_ISL_1259299, EPI_ISL_1259300, EPI_ISL_1336334, EPI_ISL_2178465, EPI_ISL_2363514			
see above	Hopital	National Reference Center for Viruses of Respiratory Infections, Institut Pasteur, Paris	Angela Brisebarre; Camille Capel; Christophe Malabat; Combe Patrice; Corinne Maufrais; Damien Mornico; Ducancelle Alexandra; Ducancelle Alexandra; Etienne Simon-Lorière; Frédéric Lemoine; Louise Lefrançois; Luizy Nelly; Marion Barbet; Martres Pascale; Maud Vanpeene; Méline Bizard; Scanvic AgnèS; Sylvie Behillil; Sylvie van der Werf; Vincent Enouf
EPI_ISL_1257892	Hopital Laquintinie de Douala	Institut Pasteur de Dakar	Dia Ndongo; Diagne Moussa Moïse; Diallo Amadou; Diop Mamadou; Elisabeth Carniel; Faye Ousmane; Loucoubar Cheikh; Njouom Richard; Sall Amadou Alpha; Sankhe Safietou
EPI_ISL_1381145, EPI_ISL_1381819, EPI_ISL_1381820, EPI_ISL_1381826, EPI_ISL_1381827, EPI_ISL_1443884, EPI_ISL_1517032, EPI_ISL_1582704, EPI_ISL_1582708, EPI_ISL_1583043, EPI_ISL_1622958, EPI_ISL_1623017, EPI_ISL_2178413, EPI_ISL_2178501, EPI_ISL_2178502, EPI_ISL_2188319, EPI_ISL_2188321			
see above	Hopital	National Reference Center for Viruses of Respiratory Infections, Institut Pasteur, Paris	Adrien Pain; Amaury Vaysse; Angela Brisebarre; Bressollette CéLine; Camille Capel; Christophe Malabat; CléMence Guillaume; Corinne Maufrais; CéLine Bressollette; Eric Farfour; Etienne Simon-Lorière; Frédéric Lemoine; Gastli Nabil; Hecquet Denise; JéRome Guinard; Louise Lefrançois; LéA Pilorge; Marion Barbet; Maud Vanpeene; Méline Bizard; Nabil Gastli; Pierre Lechat; Sylvaine Bastian; Sylvie Behillil; Sylvie Van der Werf; Sylvie van der Werf; Thibault Guinoiseau; Vincent Enouf
EPI_ISL_2401806	Hospital Center Emile Mayrisch	Laboratoire national de sante, Microbiology, Microbial Genomics Platform	Anke Wienecke-Baldacchino; Catherine Ragimbeau; Cynthia Oxacelay; Fatu Djabi; Jessica Tapp; Lise Pignon; Raoul Salmon; Tamir Abdelrahman
EPI_ISL_1384133, EPI_ISL_1384141	Hospital Center Luxembourg	Laboratoire national de sante, Microbiology, Microbial Genomics Platform	Anke Wienecke-Baldacchino; Catherine Ragimbeau; Fatu Djabi; Jean-Hugues Francois; Jessica Tapp; Lise Pignon; Michel Kohnen; Raoul Salmon; Tamir Abdelrahman
EPI_ISL_2965569, EPI_ISL_2965583, EPI_ISL_2965585	Hospital Español	Centro de Innovación en Vigilancia Epidemiológica (CiVE), Institut Pasteur Montevideo, Uruguay	Alicia Costábile; Alvaro Fajardo; Ana Moller; Andrés Lizasoain; Belén González; Bernardina Rivera; Cecilia Alonso; Cecilia Salazar; Gonzalo Bello; Gonzalo Moratorio; Gregorio Iraola; Henry Albornoz; Ignacio Ferrés; Javier Hurtado; Juan Zanetti; Julio Medina; Luciana Griffero; Lucía Spangenberg; Ma Noel Bentancor; Ma Pía Techera; Mailen Arleo; Martina Alonso; Matías Maidana; Mauricio Méndez; Melissa Duquía; Mercedes Paz; Natalia Rego; Natalia Reyes; Nicolas Nin; Odhille Chappos; Paula Perbolianachis; Pilar Moreno; Rodney Colina; Rodrigo Arce; Tamara Fernández-Calero; Tania Possi; Veronica Noya; Viviana Bortagaray
EPI_ISL_1647957, EPI_ISL_2483973	Hospital General Universitario Gregorio Marañón	Hospital General Universitario Gregorio Marañón	Cristina Rodriguez-Grande; Darío García de Viedma; Julia Suárez; Laura Pérez-Lago; Marta Herranz Martin; Patricia Muñoz; Pedro Sola Campoy; Pilar Catalán; Sergio Buenestado Serrano; Victor Manuel de la Cueva
EPI_ISL_539496	Hospital Nostra Senyora de Meritxell	Instituto de Salud Carlos III	A. Monzón; F. Casas; F. Fernández; I; I. Jiménez; Iglesias-Caballero; M. Camarero; M. Cuesta; M. González-Esguevillas; M. Molinero Calamita; M. Zaballos; P. Jiménez; S. Juliá; S. Pozo; S. Varona
EPI_ISL_2516574	Hospital Universitari Bellvitge	Microbiology Department	Aida Gonzalez-Diaz; Carmen Ardanuy; Jordi Camara; Jordi Niubó; Laura Calatayud; M Angeles Domínguez; Miguel Fernandez-Huerta; Sara Marti
EPI_ISL_819339, EPI_ISL_2162246	Hospital Universitari Vall d'Hebron - Vall d'Hebron Institut de Recerca	Hospital Universitari Vall d'Hebron - Vall d'Hebron Institut de Recerca	Alejandra González-Sánchez; Andrés Antón; Ariadna Rando; Carla Castillo; Cristina Andrés; Damir Garcia-Cehic; Josep F Abril; Josep Quer; Juliana Esperalba; Maria Carmen Martin; Maria Gema Codina; Maria Piñana; Tomàs Pumarola
EPI_ISL_2223696, EPI_ISL_2225235	Houston Methodist Hospital	Houston Methodist Hospital	Ilya J. Finkelstein; James J. Davis; Jessica Cambric; Jimmy Gollihar; Kristina Reppond; Layne Pruitt; Madison N. Shyer; Marcus Nguyen; Matthew Ojeda Saavedra; Paul A. Christensen; Prasanti Yerramilli; Randall J. Olsen; Robert Olson; Ryan Gadd; S. Wesley Long; Sishir Subedi; and James M. Musser
EPI_ISL_645030	Human Genome Variation Research Group, Malopolska Centre of Biotechnology	Human Genome Variation Research Group, Malopolska Centre of Biotechnology	Botwina, P.; Branicki, W.; Dabrowska, A.; Foremny, J.; Gromowski, T.; Klajmon, A.; Kopera, K.; Kowalski, M.; Labaj; Marszalek, K.; Owczarek, K.; P.P.; Pisarek, A.; Pospiech, E.; Pyrc, K.; Sanak, M.; Swadzba, J.; Szczepanski, A.
EPI_ISL_526224, EPI_ISL_526225, EPI_ISL_1041199, EPI_ISL_1041201, EPI_ISL_1041211	Hungarian Defence Forces Military Medical Centre	National Laboratory of Virology, Szentágothai Research Centre	Balázs Somogyi; Bálint Eszenyi; Csaba Pereszlényi; Endre Gábor Tóth; Ferenc Jakab; Gábor Kemenesi; Ágnes Balázs-Nagy
EPI_ISL_1110181, EPI_ISL_1110207, EPI_ISL_1672676, EPI_ISL_1672679,	Hôpital Avicenne	Department of Virology, Henri Mondor University Hospital, Assistance Publique Hôpitaux de Paris, Université Paris-Est Créteil, INSERM U955	Alexandre Soulier; Christophe Rodriguez; Elisabeth Trawinski; Guillaume Gricourt; Jean-Michel Pawlotsky; Melissa N'Debi; Slim Fourati; Vanessa Demontant

EPI_ISL_1672704			
EPI_ISL_940160	Hôpital Bichat Claude Bernard, Laboratoire de Virologie	IAME UMR1137 Inserm, Université de Paris, Hôpital Bichat	Alexandre Storto; Amélie Recoing; Antoine Bridier-Nahmias; Benoit Visseaux; Charlotte Charpentier; Diane Descamps; Gilles Collin; Lena Daniel; Mélanie Bertine; Nadhira Houhou-Fidouh; Quentin Le Hingrat; Siham Hamri
EPI_ISL_1085610, EPI_ISL_1085617, EPI_ISL_1088552, EPI_ISL_1088554, EPI_ISL_1088565, EPI_ISL_1088566, EPI_ISL_1110156, EPI_ISL_1110157, EPI_ISL_1110173, EPI_ISL_1110178, EPI_ISL_1110203			
see above	Hôpital Henri Mondor	Department of Virology, Henri Mondor University Hospital, Assistance Publique Hôpitaux de Paris, Université Paris-Est Créteil, INSERM U955	Alexandre Soulier; Christophe Rodriguez; Elisabeth Trawinski; Guillaume Gricourt; Jean-Michel Pawlotsky; Melissa N'Debi; Slim Fourati; Vanessa Demontant
EPI_ISL_1672723, EPI_ISL_1672794	Hôpital Paul Brousse	Department of Virology, Henri Mondor University Hospital, Assistance Publique Hôpitaux de Paris, Université Paris-Est Créteil, INSERM U955	Alexandre Soulier; Christophe Rodriguez; Elisabeth Trawinski; Guillaume Gricourt; Jean-Michel Pawlotsky; Melissa N'Debi; Slim Fourati; Vanessa Demontant
EPI_ISL_1110170, EPI_ISL_1110210	Hôpital Pitié-Salpêtrière	Department of Virology, Henri Mondor University Hospital, Assistance Publique Hôpitaux de Paris, Université Paris-Est Créteil, INSERM U955	Alexandre Soulier; Christophe Rodriguez; Elisabeth Trawinski; Guillaume Gricourt; Jean-Michel Pawlotsky; Melissa N'Debi; Slim Fourati; Vanessa Demontant
EPI_ISL_1672452, EPI_ISL_1672453	Hôpitaux Universitaires de Strasbourg NHC	Department of Virology, Henri Mondor University Hospital, Assistance Publique Hôpitaux de Paris, Université Paris-Est Créteil, INSERM U955	Alexandre Soulier; Christophe Rodriguez; Elisabeth Trawinski; Guillaume Gricourt; Jean-Michel Pawlotsky; Melissa N'Debi; Slim Fourati; Vanessa Demontant
EPI_ISL_2359858	ICH-SION	Laboratory of genomics and metagenomics	Claire Bertelli; Damien Jacot; Gilbert Greub; Sébastien Aeby; Trestan Pillonel
EPI_ISL_1097023, EPI_ISL_1097024	IHU Mediterranee Infection	IHU Mediterranee Infection	Philippe Colson et al.
EPI_ISL_2444814, EPI_ISL_2444817, EPI_ISL_2444827, EPI_ISL_2444829	IICS-UNA	IICS-UNA	Adriana Valenzuela; Alejandra Rojas; Chyntia Diaz; Eva Nara; Fatima Cardozo; Florencia del Puerto; Joel Ortiz; Jonas Fernandez; Laura Franco; Laura Mendoza; Leticia Rojas; Magaly Martinez; Maria Eugenia Galeano.
EPI_ISL_1495760, EPI_ISL_1495761	ILV Kärnten	Bergthaler laboratory, CeMM Research Center for Molecular Medicine of the Austrian Academy of Sciences	Andreas Bergthaler; Anna Schedl; Bekir Erguner; Benedikt Agerer; Christoph Bock; Fabian Amman; Jan Laine; Lukas Endler; Maelle Le Moing; Martin Senekowitsch; Michael Schuster; Petr Triska; Thomas Penz
EPI_ISL_2628208	INHRR	Laboratorio de Virología Molecular	Carmen L Loureiro; CoViVen Group; Domingo J Garzaro; Esmeralda Vizzi; Flor H Pujol; Héctor R Rangel; José Luis Zambrano; Lieska Rodríguez; Mariana Hidalgo; Pierina D´Angelo; Rossana C Jaspe; Víctor Alarcón; Yoneira Sulbaran; Zoila Moros
EPI_ISL_2879781	INSACOG Surveillance	INSACOG at CSIR Institute of Genomics and Integrative Biology	INSACOG
EPI_ISL_2521611	INSACOG-Mizoram	National Institute of Biomedical Genomics – INSACOG	Arindam Maitra; Gracy Laldinmawii; N Senthil Kumar; Nidhan Kumar Biswas; Saumitra Das; Sreedhar Chinnaswamy; Swagnik Roy
EPI_ISL_2132720, EPI_ISL_2548435, EPI_ISL_2548441	INSACOG-WB	National Institute of Biomedical Genomics – INSACOG	Ajay Chakraborti; Arindam Maitra; Bhaswati Bandyopadhyay; Nidhan Kumar Biswas; Saumitra Das; Sreedhar Chinnaswamy; Tamal Ghosh
EPI_ISL_826823	INSPI-CRN DE INFLUENZA Y OTROS VIRUS RESPIRATORIOS	Instituto de Salud Publica de Chile	Alfredo Bruno; Andres Castillo; Barbara Parra; Domenica de Mora; Gisselle Barra; Jaime Lagos; Javier Tognarelli; Jimmy Garcez; Jorge Fernandez; Loredana Arata; Manuel Gonzalez; Martiza Olmedo; Michelle Paez; Patricia Bustos; Rodrigo Fasce; Solon Narvaez
EPI_ISL_2015111	IU-Cerrahpasa, Cerrahpasa School of Medicine, COVID-19 Lab	IU-Cerrahpasa, Cerrahpasa School of Medicine, COVID-19 Lab	Kenan Midilli; Mert Kuskucu; Yesim Tuyji Tok
EPI_ISL_2628303, EPI_ISL_2628304, EPI_ISL_2628306, EPI_ISL_3281603, EPI_ISL_3298689, EPI_ISL_3298766	IVIC	Laboratorio de Virología Molecular	Carmen L Loureiro; CoViMol Group; CoViVen Group; Domingo J Garzaro; Esmeralda Vizzi; Flor H Pujol; Héctor R Rangel; José Luis Zambrano; Lieska Rodríguez; Mariana Hidalgo; Pierina D´Angelo; Rossana C Jaspe; Víctor Alarcón; Yoneira Sulbaran; Zoila Moros
EPI_ISL_1086108, EPI_ISL_1509303	IZSM	TIGEM	Andrea Ballabio; Anna Manfredi; Antonio Grimaldi; Antonio Grimaldi Patrizia Annunziata Francesco Panariello Biancamaria Pierri Claudia Tiberio Valentina Bouche Chiara Colantuono Maria Concetta Cuomo Denise Di Concilio Lucio Di Filippo Anna Manfredi Marcello Salvi Antonio Limone Luigi Atripaldi Pellegrino Cerino Andrea Ballabio Davide Cacchiarelli; Antonio Limone; Biancamaria Pierri; Chiara Colantuono; Davide Cacchiarelli.; Denise Di Concilio; Francesco Panariello; Lucio Di Filippo; Marcello Salvi; Maria Concetta Cuomo; Patrizia Annunziata; Pellegrino Cerino; Valentina Bouche
EPI_ISL_2365910	Imeda Hospital	Imelda Hospital	Dagmar Obbels; Hanne Valgaeren; Johan Frans
EPI_ISL_2001064, EPI_ISL_2001068	Immunology, Noguchi Memorial Institute for Medical Research	Immunology, Noguchi Memorial Institute for Medical Research	A.W.; Adu, B.; Agbodzi, B.; Appiah-Kubi, J.; Asare; Assane, H.; Awunyo, S.; Bonney; Campbell, A.; Dagnran, A.; Dorkenoo, A.; Gevao, S.; Halatoko; Harding, D.; Issa, Z.; J.K.; K.M.; Kossi, K.; Kumordjie, S.; Lamboni, L.; Layibo, Y.; Maman, I.; Mohktar, Q.; Rogers, J.; Sadji; Salah, D.; Salou, M.; Wurie, I.; Y.A.
EPI_ISL_1517392	Inciensa, Instituto Costarricense de Investigación y Enseñanza en Nutrición y Salud	Inciensa, Instituto Costarricense de Investigación y Enseñanza en Nutrición y Salud	Cristian Peréz-Corrales; Valeria Peralta-Barquero & Patricia Salas
EPI_ISL_893214	Indiana Animal Disease Diagnostic Laboratory	Carpi Laboratory - Purdue University	Abebe A Fola; G Kenitra Hendrix; Giovanna Carpi; Ilinca I Ciubotariu; Jack Dorman; Lev Gorenstein; Rebecca P Wilkes
EPI_ISL_3275174, EPI_ISL_3275175, EPI_ISL_3275380, EPI_ISL_3275381, EPI_ISL_3275382, EPI_ISL_3275384, EPI_ISL_3275385, EPI_ISL_3275390			
see above	Indira Gandhi Memorial Hospital	Indira Gandhi Memorial Hospital	Dr. Milza Abdul Muhsin; Mr. Ibrahim Nishan Ahmed; Ms. Aishath Shuhudha; Ms. Aminath Nazfa; Ms. Fathimath Zimna
EPI_ISL_1502814	Infectious Diseases Department , Instituto Nacional de Ciencias Medicas y Nutrición	Instituto Nacional de Ciencias Medicas y Nutricion Infectious Diseases	Diana Paola Davalos Martinez; Fernando Arteaga Cabello; Fernando Ledesma Barrientos; Guillermo M. Ruiz-Palacios; Luis Alberto Garcia Andrade; Luz Elena Cervantes Villar; Miriam Arciniega Fuentes; Pilar Ramos Cervantes; Violeta Ibarra Gonzalez
EPI_ISL_2151337	Infectious Diseases, King Faisal Hospital Research Center	Infectious Diseases, King Faisal Hospital Research Center	Al-Qahtani A, A.; Alahideb B, M.; Alhamlan F, S.; Almaghrabi R, S.; Alsanea M, S.; Althawadi S, I.; Balavenkatesh Mani; M. and UdayaRaja G, K.; Mutabagani M, S.
EPI_ISL_1692506, EPI_ISL_2186300, EPI_ISL_2282386, EPI_ISL_2283188, EPI_ISL_2323864	Infinity Biologix	Centers for Disease Control and Prevention Division of Viral Diseases, Pathogen Discovery	Adrian Paskey; Benjamin Rambo-Martin; Chirayu Goswami; Christian Bixby; Christopher Gulvick; Clinton R. Paden; Dakota Howard; Darlene Wagner; Dhwani Batra; Duncan MacCannell; Jason Caravas; Jonathan Schultz; Kara Moser; Matthew Schmerer; Peter W. Cook; Robin Grimwood; Russ Hager; Scott Sammons; Shatavia Morrison; Yihe Wang; Yvette Unoarumhi
EPI_ISL_2281237, EPI_ISL_2281238, EPI_ISL_2281245	Insitut Pasteur Bangui	Institut Pasteur de Dakar	Amadou Alpha Sall; Amadou Diallo; Benjamin Selekon; Cheikh Loucoubar; Christian Malaka; Mamadou Diop; Marie-Astrid Vernet; Moussa Moïse Diagne; Ndack Ndiaye; Ndongo Dia; Ousmane Faye; Rivalyn Nakoune Yandoko; Safietou Sankhe; Sandra Garba Ouangole
EPI_ISL_1908147, EPI_ISL_1908148, EPI_ISL_1908155, EPI_ISL_1908158, EPI_ISL_1908735, EPI_ISL_1909099, EPI_ISL_1909241, EPI_ISL_1909243, EPI_ISL_1909244, EPI_ISL_1913077, EPI_ISL_1913165			
see above	Institut National d'Hygiène	Laboratoire de Biotechnologie	Abdelomunim Essabbar; Fatima El Falaki; Fatima ElFalaki; Hicham Oumzil; Lahcen Belyamani and Azeddine Ibrahimi; Mohamde Rhajaoui; Mohamed Rhajaoui; Mouna Ouadghiri; Saaïd Amzazi; Tarik Aanniz
EPI_ISL_1434443, EPI_ISL_1436817	Institut National d'Hygiène	Unité Mixte Internationale TransVIHMI (UMI 233 IRD – U1175 INSERM - Université de Montpellier) IRD (Institut de recherche pour le développement)	Abla A. KONOU; Adodo SADJI; Ahidjo AYOUBA; Akoélé SILIADIN; Alassane OURO-MEDELI; Amivi EHLAN; Améyo DORKENOO; Anoumou DAGNRA; Christelle BUTEL; Déléma MABA; Eric DELAPORTE; Issaka Maman; Kokou TEGUENI; Laetitia SERRANO; Martine PEETERS; Messanh DOUFFAN; Mireille PRINCE-DAVID; Mounerou SALOU; Sidonie A.M.KAGNISSODE; Sika DOSSIM; Wembo A. HALATOKO
EPI_ISL_2111158	Institut National d'Hygiène Consortium National de Veille Génomique - Maroc	Laboratoire de Biotechnologie	Abdelomunim Essabbar; Fatima El Falaki; Hicham Oumzil; Lahcen Belyamani and Azeddine Ibrahimi; Mohamed Rhajaoui; Mouna Ouadghiri; Saaïd Amzazi; Tarik Aanniz
EPI_ISL_1443002	Institut National d'hygiène	"Unité Mixte Internationale TransVIHMI (UMI 233 IRD – U1175 INSERM - Université de Montpellier) IRD (Institut de recherche pour le développement)"	Abla A. KONOU; Adodo SADJI; Ahidjo AYOUBA; Akoélé SILIADIN; Alassane OURO-MEDELI; Amivi EHLAN; Améyo DORKENOO; Anoumou DAGNRA; Christelle BUTEL; Déléma MABA; Eric DELAPORTE; Issaka Maman; Kokou TEGUENI; Laetitia SERRANO; Martine PEETERS; Messanh DOUFFAN; Mireille PRINCE-DAVID; Mounerou SALOU; Sidonie A.M.KAGNISSODE; Sika DOSSIM; Wembo A. HALATOKO
EPI_ISL_1303031, EPI_ISL_2278010	Institut National d’Hygiène (INH)	Unité Mixte Internationale TransVIHMI (UMI 233 IRD – U1175 INSERM - Université de Montpellier)IRD (Institut de recherche pour le développement)	Abla A. KONOU; Adodo SADJI; Ahidjo AYOUBA; Akoélé SILIADIN; Alassane OURO-MEDELI; Amivi EHLAN; Améyo DORKENOO; Anoumou DAGNRA; Christelle BUTEL; Déléma MABA; Eric DELAPORTE; Issaka Maman; Kokou TEGUENI; Laetitia SERRANO; Martine PEETERS; Messanh DOUFFAN; Mireille PRINCE-DAVID; Mounerou SALOU; Sidonie A.M.KAGNISSODE; Sika DOSSIM; Wembo A. HALATOKO
EPI_ISL_418206, EPI_ISL_418207, EPI_ISL_418212, EPI_ISL_418216, EPI_ISL_420078, EPI_ISL_477154, EPI_ISL_482878			
see above	Institut Pasteur Dakar	Institut Pasteur de Dakar	Amadou Alpha Sall; Amadou Alpha Sall.; Amadou Alpha sall; Mamadou Diop; Mamadou Malado Jallow; Mamadou malado Jallow; Marie Henriette Dior Ndione; Moussa Moise Diagne; Ndongo Dia; Ousmane Faye; Safietou Sankhe
EPI_ISL_498239	Institut Pasteur de Dakar	Institut Pasteur de Dakar	Amadou Alpha Sall.; Mamadou Diop; Mamadou Malado Jallow; Marie Henriette Dior Ndione; Moussa Moise Diagne; Ndongo Dia; Ousmane Faye; Safietou Sankhe Mbengue
EPI_ISL_1013236, EPI_ISL_1013425	Institut Pasteur de Guadeloupe	National Reference Center for Viruses of Respiratory Infections, Institut Pasteur, Paris	Angela Brisebarre; Camille Capel; Etienne Simon-Lorière; Marion Barbet; Maud Vanpeene; Méline Bizard; Sylvie Behillil; Sylvie van der Werf; Talarmin Antoine; Vincent Enouf
EPI_ISL_2245780, EPI_ISL_2245783, EPI_ISL_2245787, EPI_ISL_2245796, EPI_ISL_2245801, EPI_ISL_2245805, EPI_ISL_2245818, EPI_ISL_2245827, EPI_ISL_2245864, EPI_ISL_2245903, EPI_ISL_2245923, EPI_ISL_2245924, EPI_ISL_2245946, EPI_ISL_2245964, EPI_ISL_2245969, EPI_ISL_2245970, EPI_ISL_2245974			
see above	Institut Pasteur de Guinée	Institut Pateur de Dakar	Dia Ndongo; Diagne Moussa Moïse; Diallo Amadou; Diop Mamadou; Faye Ousmane; Grayo Solene; Loucoubar Cheikh; Maimouna Mbanne; Mbengue Safietou Sankhe; Mohamed Kane; Ndiaye Ndack; Sall Amadou Alpha; Tordo Noel
EPI_ISL_613429	Institut Pasteur de la Guadeloupe	Institut Pasteur de la Guadeloupe	Angela Brisebarre; Antoine Talarmin; Camille Capel; Etienne Simon-Lorière; Marion Barbet; Maud Vanpeene; Méline Bizard; Stéphanie Guyomard; Sylvie Behillil; Sylvie van der Werf; Sébastien Breurec; Vincent Enouf
EPI_ISL_2966236	Institut Pasteur du Maroc	Functional Genomic Plateform/CNRST	Alaoui Sanaa-amine; Chouati Taha; EL FAHIME Elmostafa; Elannaz Hicham; Ennibi Khalid; Ghamaz Hamza; Hemlali Mouhsine; MERABET MOUAD; Maaroufi Abderrahmane; Melloul Marouane; NOURLIL Jalal; Touil Nadia; Youbi Mohammed
EPI_ISL_2110643	Institut Pasteur du Maroc	Functional Genomics platform/CNRST	Abdelkrim Meziane Bellefquih; Alaoui Sanaa-amine; Chouati Taha; EL FAHIME Elmostafa; Elannaz Hicham; Ennibi Khalid; Hemlali Mouhsine; MERABET MOUAD; Maaroufi Abderrahmane; Melloul Marouane; NOURLIL Jalal; Touil Nadia; Youbi Mohammed

EPI_ISL_459972	Institut Pasteur du Maroc	Institut Pasteur du Maroc	Abdellah Faouzi; Anass Abbad; Anderrahmane Maaroufi; Angela Brisebarre; Camille Capel; Etienne Simon-Lorière; Jalal Nourlil; Latifa Anga; Marion Barbet; Maud Vanpeene; Mjid Elouалid; Méline Bizard; Sylvie Behillil; Sylvie van der Werf; Vincent Enouf
EPI_ISL_475820	Institut für Virologie am Department für Hygiene, Mikrobiologie und Public Health	Bergthaler laboratory, CeMM Research Center for Molecular Medicine of the Austrian Academy of Sciences	Alexander Lercher; Alexandra Popa; Andreas Bergthaler; Benedikt Agerer; Christoph Bock; Daniela Schmid; Dorothee von Laer; Elisabeth Puchhammer-Stoeckl; Franz Allerberger; Gregor Hörmann; Guenter Weiss; Henrique Colaco; Jakob-Wendelin Genger; Jan Laine; Judith Aberle; Kinga Rigler-Hohenwarter; Lukas Endler; Manfred Nairz; Mark Smyth; Martin Senekowitsch; Michael Schuster; Peter Hufnagl; Rainer Gattringer; Stephan Aberle; Thomas Penz; Wegene Borena
EPI_ISL_955163	Institute for Biocides and Medical Ecology	Institute of microbiology and Immunology, Faculty of Medicine, University of Belgrade	Jankovic, M.; Jovanovic, T.; Knezevic, A.; Milicevic, O.; Sekler, M.; Tesovic, B.; Vidanovic, D.
EPI_ISL_1633465, EPI_ISL_1633466, EPI_ISL_1633467	Institute for Health Research, Epidemiological Surveillance and Training (IRESSEF)	Abbott	Adbou Padane; Ambroise Ahouidi; Aminata Dia; Aminata Mboup; Ana Olivo; Anna julienne selbe Ndiaye; Barbara Harris; Cyrille Diedhiou; Gavin Cloherty; Mary Rodgers; Moustapha Mbow; Nafissatou Leye; Ndeye Diabou Diagne; Papa Alassane Diaw; Souleymane Mboup; Todd Meyer
EPI_ISL_1827860, EPI_ISL_1827865, EPI_ISL_1827867, EPI_ISL_1827877, EPI_ISL_1827932, EPI_ISL_1827941, EPI_ISL_1827949			
see above	Institute for Health Research, Epidemiological Surveillance and Training (IRESSEF)	Abbott Laboratories	Adbou Padane; Ambroise Ahouidi; Aminata Dia; Aminata Mboup; Ana Olivo; Anna julienne selbe Ndiaye; Barbara Harris; Cyrille Diedhiou; Gavin Cloherty; Mary Rodgers; Moustapha Mbow; Nafissatou Leye; Ndeye Diabou Diagne; Papa Alassane Diaw; Souleymane Mboup; Todd Meyer
EPI_ISL_1495277, EPI_ISL_1495278, EPI_ISL_1495280, EPI_ISL_1495283, EPI_ISL_1495356	Institute for Laboratory Diagnostics and Microbiology, Klinikum Klagenfurt am Wörthersee	Bergthaler laboratory, CeMM Research Center for Molecular Medicine of the Austrian Academy of Sciences	Andreas Bergthaler; Anna Schedl; Bekir Erguner; Benedikt Agerer; Christoph Bock; Fabian Amman; Jan Laine; Lukas Endler; Maelle Le Moing; Martin Senekowitsch; Michael Schuster; Petr Triska; Thomas Penz
EPI_ISL_490101, EPI_ISL_644672, EPI_ISL_718275, EPI_ISL_2091000, EPI_ISL_2625633	Institute for Medical Research, Infectious Disease Research Centre, National Institutes of Health, Ministry of Health Malaysia	Institute for Medical Research, Infectious Disease Research Centre, National Institutes of Health, Ministry of Health Malaysia	Azizan MA; Kalyanasundram J; Kamel K; Mohd Zawawi Z; Mohd-Zawawi Z; Ramly N; Robert F; Suppiah J; Thayan R
EPI_ISL_2104730, EPI_ISL_2678097	Institute for Urban Disease Control and Prevention	COVID-19 Network Investigations (CONI) Alliance	Amornmas Kongklieng; Angkana Huang; Anthony R. Jones; Arporn Wangwiwatsin; Bhakbhoom Panthan; Chonticha Klungtong; Duangkamon Loesbanluechai; Ekawat Pasomsub; Elizabeth Batty; Insee Sensorn; Janjira Thaipadungpanit; Kamolthip Atsawawaranunt; Khajohn Joonlasak; Kingkan Rakmanee; Krittikorn Kumpornsin; Namfon Kotanan; Prayuth Kaewmalang; Pukkaporn Parnwijitkul; Stefan Fernandez; Thanat Chookajorn; Theerarat Kochakarn; Treewat Watthanachockchai; Vichan Pawun; Wasun Chantratita; Wudtichai Manasatienkij
EPI_ISL_1055791, EPI_ISL_1056970, EPI_ISL_1057041	Institute of Biocides and Medical Ecology, Belgarde, Serbia	Virology Department Institute of Microbiology and Immunology Faculty of Medicine University of Belgrade	Abazovic Dzihan; Banko Ana; Despot Dragana; Loncar Ana; Milicevic Ognjen; Miljanovic Danijela
EPI_ISL_600562	Institute of Epidemiology Disease Control And Research	Institute for Developing Science and Health Initiatives	Firadausi Qadri; Lauren Cowley; Md. Mahfuz-Al-mamun; Mokibul Hassan Afrad; Sadia Isfat Ara Rahman; Tahmina Shirin
EPI_ISL_2600368, EPI_ISL_2600369	Institute of Epidemiology, Disease Control and Research (IEDCR)	IEDCR-ideSHi-icddr,b	Firdausi Qadri; Hassan Afrad; Manjur Hossain Khan; Sadia Rahman; Tahmina Shirin
EPI_ISL_1599187, EPI_ISL_1915438, EPI_ISL_1938476	Institute of Epidemiology, Disease Control and Research (IEDCR)	Institute for Developing Science and Health Initiatives (ideSHi)	Fidausi Qadri; Hassan Afrad; Sadia Rahman; Tahmina Shirin
EPI_ISL_463091	Institute of Life Sciences, Bhubaneswar	Immunogenomics lab, Institute of Life Sciences, Bhubaneswar	Ajay Parida; Arup Ghosh; Atimukta Jha; DBT's PAN-INDIA 1000 SARS-CoV2 RNA genome sequencing consortium; Eshna Laha; Ghulam Hussain Syed; ILS COVID-19 TEAM; Kautilya Kumar Jena; Manasi Priyadarshini; Neha Singh; Orissa COVID-19 Study Group; Punit Prasad; Rajeeb Swain; Rupesh Dash; Saiket De; Sandhya Suranjika; Shanti Senapati; Shuchi Smita; Soma Chattopadhyay; Sunil Raghav; Swati Madhulika; Tushar K. Beuria; Viplov K. Biswas
EPI_ISL_1063496, EPI_ISL_1063521, EPI_ISL_1063522, EPI_ISL_1063996, EPI_ISL_1118863, EPI_ISL_1118864, EPI_ISL_1181840, EPI_ISL_1181852, EPI_ISL_1181856, EPI_ISL_1181859, EPI_ISL_1181860, EPI_ISL_1181861, EPI_ISL_1181862, EPI_ISL_1250645, EPI_ISL_1250657, EPI_ISL_1250658, EPI_ISL_1250661, EPI_ISL_1250667, EPI_ISL_1250677, EPI_ISL_1250678, EPI_ISL_1250681, EPI_ISL_1273386, EPI_ISL_1273388, EPI_ISL_1406287, EPI_ISL_1406290, EPI_ISL_1495066, EPI_ISL_1509922, EPI_ISL_1657058, EPI_ISL_1668564, EPI_ISL_1668566, EPI_ISL_1668567, EPI_ISL_1668569, EPI_ISL_1668571, EPI_ISL_1675247, EPI_ISL_1675248, EPI_ISL_1675250, EPI_ISL_1964287, EPI_ISL_1973575, EPI_ISL_2955269			
see above	Institute of Microbiology and Immunology, Faculty of Medicine, University of Ljubljana	Institute of Microbiology and Immunology, Faculty of Medicine, University of Ljubljana	Alen Suljič; Andraž Celar; Andrej Celar; Dominika Šturm; Doroteja Vlaj; Mario Poljak; Matic Brvar; Miša Korva; Patricija Pozvek; Samo Zakotnik; Tatjana Avšič – Županc; Tomaž Mark Zorec; Špela Pleh
EPI_ISL_417482, EPI_ISL_516650, EPI_ISL_697783, EPI_ISL_697786, EPI_ISL_728202, EPI_ISL_1896696, EPI_ISL_2004106, EPI_ISL_2086712, EPI_ISL_2228102, EPI_ISL_2484966, EPI_ISL_2534327			
see above	Institute of Microbiology, Universidad San Francisco de Quito	Institute of Microbiology, Universidad San Francisco de Quito	Belén Prado-Vivar; Bernardo Darquea; Bernardo Gutiérrez; Ernesto Pazos; Fernada Zurita; Fernanda Zurita; Gabriel Trueba; Guzmán Bernabéu Lorenzo; Jonathan Araujo; Jorge Huertas; Juan José Guadalupe; Manuel Jibaja; Mayra Beltrán; Melva Morales; Michelle Grunauer; Milton Tobar; Monica Becerra-Wong; Patricio Reyes; Patricio Rojas-Silva; Paúl Cárdenas; Prado-Vivar; Rommel Guevara; Sully Márquez; Tania Guayasamin; Tanya Guayasamin; Verónica Barragán
EPI_ISL_1443642	Institute of Microbiology, Universidad San Francisco de Quito	Omics Sciences Laboratory	; Andrea Cunguan; Belén Prado-Vivar; Bernardo Gutiérrez; Darlyn Amaya; Dayron Brossad; Derly Andrade Molina; Emily Sulay Saltos Montalvo; Fernanda Zurita; Gabriel Morey León; Gabriel Trueba; Juan Carlos Fernández Cadena; Juan José Guadalupe; Katheryn Sacheri Viteri; Michelle Grunauer; Monica Becerra-Wong; Nabih Dahik; Patricio Rojas-Silva; Paula Juliana Gavilanes Jarrín; Paúl Cárdenas; Rubén Armas González; Sully Márquez; Verónica Barragán
EPI_ISL_403931	Institute of Pathogen Biology, Chinese Academy of Medical Sciences & Peking Union Medical College	Institute of Pathogen Biology, Chinese Academy of Medical Sciences & Peking Union Medical College	Chao Wu; Jianwei Wang; Lili Ren; Qi Jin; Yiwei Liu; Zhiqiang Wu; Zichun Xiang
EPI_ISL_2657582	Institute of Public Health Šibenik-Knin County	Croatian Institute of Public Health	Irena Tabain; Ivana Ferenčak
EPI_ISL_577740, EPI_ISL_1547856, EPI_ISL_2351249	Institute of Virology, Biomedical Research Center of the Slovak Academy of Sciences, Bratislava	Faculty of Natural Sciences, Comenius University, Bratislava	Boris Klempa; Brona Brejova; Broňa Brejová; Dominika Fričová; Edita Staroňová; Elena Tichá; Jozef Nosek; Juraj Kopacek; Juraj Kopáček; Kristina Borsova; Kristína Boršová; Lubomira Lukacikova; Martina Lickova; Martina Ličková; Martina Nebohacova; Martina Neboháčová; Monika Slavikova; Monika Sláviková; Sabina Fumacova Havlikova; Sabina Fumačová Havlíková; Tomas Vinar; Tomáš Vinař; Viktoria Cabanova; Viktoria Hodorova; Viktória Hodorová; Viktória Čabanová; Ľubomíra Lukáčiková
EPI_ISL_3200834, EPI_ISL_3200835, EPI_ISL_3200837, EPI_ISL_3200841	Institute of Virology, Medical Center, University of Freiburg, Freiburg, Germany	Institute of Virology, Clinial Virus Genomics, Medical Center, University of Freiburg, Freiburg, Germany	Hajo Grundmann; Jonas Fuchs; Lena Jaki; Lisa Kern; Marcus Panning; Sandra Reuter
EPI_ISL_1654823, EPI_ISL_3163943, EPI_ISL_3163954	Institute of Virology, Vaccines and Sera "Torlak"	Institute of microbiology and Immunology, Faculty of Medicine, University of Belgrade	Banko A.; Cupic M.; Jankovic, M.; Jovanovic, T.; Knezevic, A.; Lazarevic I.; Milicevic, O.; Miljanovic D.; Sekler, M.; Tesovic, B.; Vidanovic, D.
EPI_ISL_860633	Instituto Adolfo Lutz - Regional de Sorocaba	Instituto Adolfo Lutz, Interdiciplinary Procedures Center, Strategic Laboratory	Claudia Regina Gonçalves; Claudio Tavares Sacchi; Erica Valessa Ramos Gomes; Karoline Rodrigues Campos
EPI_ISL_2344260	Instituto Butantan	Instituto de Medicina Tropical de Sao Paulo	Brazil-UK Centre for Arbovirus Discovery Diagnosis Genomics and Epidemiology (CADDE) Genomic Network - Instituto de Medicina Tropical
EPI_ISL_2839267	Instituto Nacional de Cancerología	Centro de Investigación en Enfermedades Infecciosas (CIENI), Instituto Nacional de Enfermedades Respiratorias (INER)	Alejandra Garcia-Gasca; Alejandra Hernández-Terán; Alejandro Sanchez-Flores; Alfredo Herrera-Estrella; Alicia Ocaña-Mondragón; Andreu Comas-García; Angel Gustavo Salas-Lais; Antonio Loza Román; Bernardo Martínez-Miguel; Blanca Taboada; Brenda Irasema Maldonado-Meza; Bruno Gomez-Gil; Carla Ivón Herrera-Najera; Carlos F. Arias; Celia Boukadida; Clara Esperanza Santacruz-Tinoco; Concepción Grajales-Muñiz; Consorcio Mexicano de Vigilancia Genómica (CoViGen-Mex). Authors (in alphabetical order): Julio Elias Alvarado-Yaah; Cristóbal Cháidez-Quiróz; Célida Duque Molina; Célida Martínez- Rodríguez; Daniel Fregoso-Rueda; Daniel Lira Morales; Eduardo Becerril-Vargas; Fernando Fontove-Herrera; Fidencio Mejía-Nepomuceno; Francisco Pulido; Gloria Elena Espinosa-Ayala; Gloria María Molina-Salinas; Gloria Vazquez; Hector Esteban Paz-Juárez; Hector Montoya-Fuentes; Helen Haydee Fernanda Ramírez-Plascencia; Irvin González-López; Jean Pierre González; Joel Armando Vázquez-Pérez.; Jorge Salas-Hernández; José Antonio Enciso-Moreno; José Arturo Martínez-Orozco; José Esteban Muñoz-Medina; José de Jesús Nuñez-Contreras; Juan Bautista Chale-Dzul; Julissa Enciso-Ibarra; Kathia Elizabeth Tapia-Diaz; Luis Alberto Ochoa-Carrera; Margarita Matías-Florentino; Mario Mújica-Sánchez; Marissa Pérez-García; María Guadalupe Santiago-Mauricio; María Guadalupe de Jesús Mireles-Rivera; Nelly Sélem-Mojica; Pavel Isa; Ricardo Ciria Merce; Ricardo Grande; Rosa María Gutierrez Rios; Santiago Ávila-Ríos; Selene Zárate; Susana Lopez; Victor Eduardo Garcia-Arias; Víctor Hugo Borja-Aburto
EPI_ISL_1301725, EPI_ISL_1302152, EPI_ISL_1302291	Instituto Nacional de Enfermedades Respiratorias (INER)	Instituto de Biotecnología de la UNAM	Alejandra Hernández-Terán; Alejandro Sanchez-Flores; Alma Rincón-Rubio; Andrea Santos Coy-Arechavaleta; Authors from IBT; Blanca Taboada; Celia Boukadida; Clara Esperanza Santacruz-Tinoco; Edgar Mendieta-Condado; Eduardo Becerril-Vargas; Fidencio Mejía-Nepomuceno; Francisco Pulido; Gisela Barrera-Badillo; Gloria Vazquez; Hector Esteban Paz-Juárez; IMSS; InDRE and INER (in alphabetical order): Carlos F. Arias; Irma Lopez-Martinez; Jerome Jean Verleyen; Joel Armando Vázquez-Pérez; Jorge Salas-Hernández; José Arturo Martínez-Orozco; José Ernesto Ramírez-González; José Esteban Muñoz-Medina; Larissa Fernandes-Matano; Lucia Hernandez-Rivas; Luis Alberto Ochoa-Carrera; Margarita Matías-Florentino; Mario Mújica-Sánchez; Natividad Cruz-Ortiz; Pavel Isa; Ricardo Grande; Santiago Ávila-Ríos; Tatiana Nunez-Garcia; Teresita Rojas-Mendoza
EPI_ISL_2492560, EPI_ISL_2492604, EPI_ISL_2492656, EPI_ISL_2492728, EPI_ISL_2492786, EPI_ISL_2492788, EPI_ISL_2492791, EPI_ISL_2492797, EPI_ISL_2492818, EPI_ISL_2492837, EPI_ISL_2492847, EPI_ISL_2492915, EPI_ISL_2617133, EPI_ISL_2617181, EPI_ISL_2617182, EPI_ISL_2617183, EPI_ISL_2685971, EPI_ISL_2685982, EPI_ISL_2685983, EPI_ISL_2841655			
see above	Instituto Nacional de Investigação em Saúde	CERI, Centre for Epidemic Response and Innvoation, Stellenbosch University and KRISP, KZN Research Innovation and Sequencing Platform, UKZN.	Afonso P; David K; Emmanuel SJ; Freitas RH; Giandhari J; Inglês L; Lutucuta S; Miranda J; Morais J; Mufinda M; Naidoo Y; Neto Z; Paulo A Carralero RR Paixão JP; Pereira A; Pillay S; Tegally H; Wilkinson E; de Oliveira T
EPI_ISL_1347897, EPI_ISL_1545286, EPI_ISL_1545330, EPI_ISL_1545369, EPI_ISL_2494774, EPI_ISL_2494868, EPI_ISL_2494878, EPI_ISL_2494885, EPI_ISL_2494900, EPI_ISL_2494944, EPI_ISL_2495023, EPI_ISL_2495048, EPI_ISL_2609536, EPI_ISL_2609539, EPI_ISL_2609561, EPI_ISL_2609562			
see above	Instituto Nacional de Investigação em Saúde	KRISP, KZN Research Innovation and Sequencing Platform	Afonso P; David K; Emmanuel SJ; Freitas RH; Giandhari J; Inglês L; Lutucuta S; Miranda J; Morais J; Mufinda M; Naidoo Y; Neto Z; Paulo A Carralero RR Paixão JP; Pereira A; Pillay S; Tegally H; Wilkinson E; de Oliveira T
EPI_ISL_791084	Instituto Nacional de Salud - Unidad de Secuenciación y Análisis Genómico	Instituto Nacional de Salud - Dirección de Investigación en Salud Pública	Carlos Franco-Muñoz; Diego A. Álvarez-Díaz; Diego Andrés Prada; Gerardo Santamaría; Jonathan Reales; Julian Naizaque; Katherine Laiton-Donato; Magdalena Wiesner; Marcela Mercado-Reyes; Maria T. Herrera; Martha Lucia Ospina Martinez; Mauricio Pacheco-Montealegre; Paola Muñoz-Laiton; Sheryl Corchuelo
EPI_ISL_941986	Instituto Nacional de Salud, Bogotá, Colombia	Centro de Investigaciones en Microbiología y Biotecnología-UR (CIMBIUR), Facultad de Ciencias Naturales, Universidad del Rosario, Bogotá, Colombia Instituto Nacional de Salud, Bogotá, Colombia Icahn School of Medicine at Mount Sinai, New York, USA	Adriana van de Guchte; Alberto Paniz-Mondolfi; Ana S. Gonzalez-Reiche; Carolina Flórez; Carolina Hernández; Emilia Mia Sordillo; Hala Alejel Alshammary; Harm van Bakel; Jayeeta Dutta; Juan David Ramírez; Luz Helena Patiño; Marina Muñoz; Matthew M. Hernandez; Nathalia Ballesteros; Sergio Gomez; Viviana Simon; Zenab Khan
EPI_ISL_498170	Instituto Nacional de Salud, Bogotá, Colombia	Instituto Nacional de Salud, Bogotá, Colombia	Astrid C. Flórez; Carlos Andrés Durán; Carlos Franco-Muñoz; Carolina Ferro; Christian Julian Villabona-Arenas; Diana Marcela Walteros-Acero; Diego A. Álvarez-Díaz; Diego Andrés Prada; Franklin Prieto; Jonathan Reales; Jose A. Usme-Ciro; Katherine Laiton-Donato; Liz Villabona-Arenas; Marcela Mercado-Reyes; Martha Lucia Ospina Martinez; Nicolas D. Franco-Sierra; Sussy Echeverria; Zulma M. Cucunubá
EPI_ISL_2617100, EPI_ISL_2617116, EPI_ISL_2617124, EPI_ISL_2617149, EPI_ISL_2617168, EPI_ISL_2688576, EPI_ISL_2688577, EPI_ISL_2688578			
see above	Instituto Nacional de Saude (INS), Mozambique	CERI, Centre for Epidemic Response and Innvoation, Stellenbosch University and KRISP, KZN Research Innovation	Emmanuel S; Giandhari J; Nadia Sitoe; Naidoo Yeshnee; Nalia Ismael; Nedio Mabunda; Paulo Arnaldo; Pillay S; Tegally H; Tshabuila Derek; Wilkinson E; Yajna Ramphal; de Oliveira T

		and Sequencing Platform, UKZN.	
EPI_ISL_887500, EPI_ISL_887502, EPI_ISL_1132826, EPI_ISL_1132834, EPI_ISL_2396897, EPI_ISL_2396914, EPI_ISL_2396919, EPI_ISL_2396926, EPI_ISL_2396931, EPI_ISL_2396934			
see above	Instituto Nacional de Saude (INS), Mozambique	KRISP, KZN Research Innovation and Sequencing Platform	Emmanuel S; Giandhari J; Nadia Sitoe; Nalia Ismael; Nedio Mabunda; Paulo Arnaldo; Pillay S; Tegally H; Wilkinson E; de Oliveira T
EPI_ISL_913940, EPI_ISL_1054995	Instituto de Diagnostico y Referencia Epidemiologicos INDRE_RNLSP	Instituto de Diagnostico y Referencia Epidemiologicos (INDRE)	Abril Rodriguez-Maldonado; Adnan Araiza-Rodriguez; Claudia Wong-Arambula; David Fragoso-Fonseca; Ernesto Ramirez-Gonzalez.; Fabiola Garces-Ayala; Gisela Barrera-Badillo; Irma Lopez-Martinez; Lucia Hernandez-Rivas; Mayra Jimenez-Morales; Nancy Munoz-Hernandez; Natividad Cruz-Ortiz; Sergio Rangel-Guerrero; Tatiana Nunez-Garcia
EPI_ISL_1301519	Instituto de Diagnostico y Referencia Epidemiologicos InDRE_RNLSP	Instituto de Biotecnología de la UNAM	Alejandra Hernández-Terán; Alejandro Sanchez-Flores; Alma Rincón-Rubio; Andrea Santos Coy-Arechavaleta; Authors from IBT; Blanca Taboada; Celia Boukadida; Clara Esperanza Santacruz-Tinoco; Edgar Mendieta-Condado; Eduardo Becerril-Vargas; Fidencio Mejía-Nepomuceno; Francisco Pulido; Gisela Barrera-Badillo; Gloria Vazquez; Hector Esteban Paz-Juárez; IMSS; InDRE and INER (in alphabetical order): Carlos F. Arias; Irma Lopez-Martinez; Jerome Jean Verleyen; Joel Armando Vázquez-Pérez; Jorge Salas-Hernández; José Arturo Martínez-Orozco; José Ernesto Ramírez-González; José Esteban Muñoz-Medina; Larissa Fernandes-Matano; Lucia Hernandez-Rivas; Luis Alberto Ochoa-Carrera; Margarita Matías-Florentino; Mario Mújica-Sánchez; Natividad Cruz-Ortiz; Pavel Isa; Ricardo Grande; Santiago Ávila-Ríos; Tatiana Nunez-Garcia; Teresita Rojas-Mendoza
EPI_ISL_2776175, EPI_ISL_2776177, EPI_ISL_2776197	Instituto de Medicina Tropical & Salud Global (IMTSAG)	Grubaugh Lab - Yale School of Public Health	Alejandaro Vallejo Degaudenzi; Anderson Brito; Annie Watkins; Chaney Kalinich; Chantal Vogels; Elisa Contreras; Esperanza Mendoza; Isabel Ott; Jessica Rothman; Joseph Fauver; Kendall Billig; Mallery Breban; Mary Petrone; Nathan Grubaugh; Robert Paulino-Ramirez; Tara Alpert; Tobias Koch; Victor Virgilio Calderon
EPI_ISL_1378833, EPI_ISL_3104763	Instituto de Medicina Tropical & Salud Global Universidad Iberoamericana	Grubaugh Lab - Yale School of Public Health	Alejandaro Vallejo Degaudenzi; Anderson Brito; Andrea Firpo; Annie Watkins; Chaney Kalinich; Chantal Vogels; David Rivera Aponte; Elisa Contreras; Ernesto Vazquez; Esperanza Mendoza; Fabiola Cruz-Lopez; Fabiola Fontanet; Isabel Ott; Isabell Ott; Jessica Rothman; Joseph Fauver; Karolane Gonzalez; Kendall Billig; Mallery Breban; Mary Petrone; Natalie Machargo; Nathan Grubaugh; Patricia Serrano; Robert Paulino-Ramirez; Tania Mitwalli; Tara Alpert; Tobias Koch; Vianca Aponte; Victor Virgilio Calderon
EPI_ISL_1627995	Instytut Onkologii	1. National Institute of Public Health - National Institute of Hygiene; 2. Eurofins Genomics Europe Sequencing GmbH	ECDC COVID-19 WGS support team; Eurofins Genomics Europe Sequencing Team; Gierczyńki Rafał; Sadkowska-Todys Małgorzata; Wołkowicz Tomasz; Zacharczuk Katarzyna
EPI_ISL_1652056, EPI_ISL_1652061, EPI_ISL_1652066	Integrated Biorepository of H3Africa Uganda – IBRH3AU	Molecular Biology Laboratory	Ashaba Fred Katabazi; Bernard Ssentalo Bagaya; David Patrick Kateete; Edgar Kigozi; Emmanuel Nasinghe; Eric Katagirya; Gerald Mboowa; Lwanga Newton; Misaki Wayengera; Moses Joloba; Moses Luutu; Nsubuga Gideon; Rogers Kamulegeya; Samuel Kirimunda; Sarah Stanley; Savannah Mwesigwa
EPI_ISL_1910387, EPI_ISL_1910391, EPI_ISL_2620888, EPI_ISL_2620889, EPI_ISL_2887849, EPI_ISL_2887851, EPI_ISL_2887852, EPI_ISL_2887857, EPI_ISL_3154879, EPI_ISL_3154885, EPI_ISL_3154890, EPI_ISL_3154891, EPI_ISL_3154892, EPI_ISL_3154897, EPI_ISL_3154898, EPI_ISL_3154899			
see above	Iressef Genomics lab	IRESSEF	Abdou PADANE; Ambroise AHOUIDI; Aminata DIA; Aminata MBOUP; Astou Gaye GAYE; Barada CISSE; Birahim Piere NDIAYE; Diabou Diagne; Gora LO; Khadim GUEYE; Moustapha MBOW; Nafisatou LEYE; Ndeye Coumba Toure KANE; Papa Alassane DIAW; Samba Ndiour; Seni Ndiaye; Souleymane MBOUP; Yacine DIA
EPI_ISL_483708, EPI_ISL_514293, EPI_ISL_514305, EPI_ISL_514308, EPI_ISL_516900, EPI_ISL_649094	Israel Central Virology laboratory	Israel Central Virology laboratory	Efrat Dahan Bucris; Ella Mendelson; Michal Mandelboim; Neta Zuckerman; Oran Erster
EPI_ISL_2183422, EPI_ISL_2183433, EPI_ISL_2636159	Israel Central Virology laboratory	Israel National Consortium for SARS-CoV-2 sequencing	Dana Bar-Ilan; Efrat Dahan Bucris; Efrat Glick-Saar; Ella Mendelson; Gideon Rechavi; Michal Mandelboim; Miranda Geva; Neta Zuckerman; Netanel Abu; Omri Nayshool; Oran Erster; Orna Mor
EPI_ISL_3275790	JIPMER Puducherry	inStem NCBS – INSACOG	Uma Ramakrishnan Dasaradhi Palakodeti Aswin SaiNarain
EPI_ISL_779272, EPI_ISL_2811827	Jamil-ur-Rahman Center for Genome Research, Dr. Panjwani Center for Molecular Medicine and Drug Research	Jamil-ur-Rahman Center for Genome Research, Dr. Panjwani Center for Molecular Medicine and Drug Research	Ain, Nu.; Ansari, S.; B.T.; I.A.; Iqbal, W.; Irfan, M.; Jahan, S.; Javed; Kakar, N.; Khan; Khan, I.; Khan, S.; Nisa, Z.; Nisa, Zu.; Rashid, M.; Rehman, Z.; Sarwar, B.; Shakeel, M.; Siddiqi, S.; Zehra, M.
EPI_ISL_3031422, EPI_ISL_3031423, EPI_ISL_3031425, EPI_ISL_3031426, EPI_ISL_3031427, EPI_ISL_3031430, EPI_ISL_3031431			
see above	Jaramogi Oginga Odinga Teaching and Referral Hospital	USAMRD-A, Basic Science Laboratory	Alan Lemtudo; Beth Mutai; Brian Andika; Carol Kifude; Clement Masakwe; Eric Muthanje; Esther Omuseni; Faith Sigei; Gathii Kimita; George Awinda; John Waitumbi; Josphat Nyataya; Rachel Githii; Rehema Liyai; Stephen Ochola
EPI_ISL_949093	Jessa	Jessa	Jessa_cmdLab
EPI_ISL_2932611, EPI_ISL_2932618	Jordan University of Science Technology/ Irbid filed Hospital/ MOH	Princess Haya Biotechnology Center/ Jordan University of Science Technology	Areej Alquran; Emad Abu Yaqeen; Hazem Haddad; Maha Karam; Saied Jaradat; Shereen Issa; Suha Hasan
EPI_ISL_3275167	K. Himmafushi	Indira Gandhi Memorial Hospital	Dr. Milza Abdul Muhsin; Mr. Ibrahim Nishan Ahmed; Ms. Aishath Shuhudha; Ms. Aminath Nazfa; Ms. Fathimath Zimna
EPI_ISL_3275170	K. Maafushi	Indira Gandhi Memorial Hospital	Dr. Milza Abdul Muhsin; Mr. Ibrahim Nishan Ahmed; Ms. Aishath Shuhudha; Ms. Aminath Nazfa; Ms. Fathimath Zimna
EPI_ISL_2779335	KCRH KEMRI-DEID Surveillance Site	USAMRD-A, Basic Science Laboratory	Alan Lemtudo; Beth Mutai; Brian Andika; Carol Kifude; Clement Masakwe; Eric Muthanje; Esther Omuseni; Faith Sigei; Gathii Kimita; George Awinda; John Waitumbi; Josphat Nyataya; Rachel Githii; Rehema Liyai; Stephen Ochola
EPI_ISL_2779331, EPI_ISL_2779466, EPI_ISL_2779546, EPI_ISL_2779547, EPI_ISL_3031382, EPI_ISL_3031383, EPI_ISL_3031386, EPI_ISL_3031387, EPI_ISL_3031401, EPI_ISL_3031407, EPI_ISL_3031409, EPI_ISL_3031410, EPI_ISL_3031416			
see above	KCRH KEMRI/DEID Surveillance Site	USAMRD-A, Basic Science Laboratory	Alan Lemtudo; Beth Mutai; Brian Andika; Carol Kifude; Clement Masakwe; Eric Muthanje; Esther Omuseni; Faith Sigei; Gathii Kimita; George Awinda; John Waitumbi; Josphat Nyataya; Rachel Githii; Rehema Liyai; Stephen Ochola
EPI_ISL_2348627, EPI_ISL_2348630	KEMRI	KEMRI & ICGEB	Alessandro Marcello; Emanuele Orsini; Eric A Lelo; Janet Majanja; Samwel L Symekher; Silvanos M Opanda; Wadegu Meshack; Wallace D Bulimo
EPI_ISL_2602986, EPI_ISL_2602987, EPI_ISL_2602988, EPI_ISL_2602990, EPI_ISL_2602991, EPI_ISL_2602992	KEMRI Center for Biotechnology Research and Development	KEMRI-Wellcome Trust Research Programme,Kilifi	; Githinji G.; Matoke D.; Mburu M.W.; Mohamed K.S.; Onyango C.; de Laurent Z.
EPI_ISL_2602784, EPI_ISL_2602785, EPI_ISL_2602795, EPI_ISL_2602799, EPI_ISL_2602807, EPI_ISL_2602810, EPI_ISL_2602820, EPI_ISL_2602825, EPI_ISL_2602831, EPI_ISL_2602874, EPI_ISL_2602887, EPI_ISL_2602894, EPI_ISL_2602905, EPI_ISL_2602910, EPI_ISL_2602911, EPI_ISL_2602915, EPI_ISL_2602916, EPI_ISL_2602920, EPI_ISL_2602923, EPI_ISL_2602926, EPI_ISL_2602930, EPI_ISL_2602933, EPI_ISL_2602936, EPI_ISL_2602939, EPI_ISL_2602940, EPI_ISL_2602942, EPI_ISL_2602945			
see above	KEMRI-CGHR	KEMRI-Wellcome Trust Research Programme,Kilifi	; Githinji G.; Matoke D.; Mburu M.W.; Mohamed K.S.; Onyango C.; de Laurent Z.
EPI_ISL_1440087, EPI_ISL_1440090, EPI_ISL_1440096, EPI_ISL_1440118, EPI_ISL_1440172, EPI_ISL_1440284, EPI_ISL_1610420, EPI_ISL_1610444, EPI_ISL_2602494, EPI_ISL_2602544, EPI_ISL_2602546, EPI_ISL_2602547, EPI_ISL_2602548, EPI_ISL_2602549, EPI_ISL_2602550, EPI_ISL_2602551, EPI_ISL_2602557, EPI_ISL_2602565, EPI_ISL_2602570, EPI_ISL_2602571, EPI_ISL_2602577, EPI_ISL_2602579, EPI_ISL_2602580, EPI_ISL_2602583, EPI_ISL_2602585, EPI_ISL_2602589, EPI_ISL_2602590, EPI_ISL_2602595, EPI_ISL_2602597, EPI_ISL_2602599, EPI_ISL_2602600, EPI_ISL_2602601, EPI_ISL_2602604, EPI_ISL_2602606, EPI_ISL_2602608, EPI_ISL_2602610, EPI_ISL_2602615, EPI_ISL_2602622, EPI_ISL_2602623, EPI_ISL_2602625, EPI_ISL_2602626, EPI_ISL_2602627, EPI_ISL_2602628, EPI_ISL_2602634, EPI_ISL_2602635, EPI_ISL_2602637, EPI_ISL_2602639, EPI_ISL_2602650, EPI_ISL_2602652, EPI_ISL_2602653, EPI_ISL_2602654, EPI_ISL_2602660, EPI_ISL_2602661, EPI_ISL_2602663, EPI_ISL_2602664, EPI_ISL_2602665, EPI_ISL_2602666, EPI_ISL_2602667, EPI_ISL_2602668, EPI_ISL_2602670, EPI_ISL_2602671, EPI_ISL_2602676, EPI_ISL_2602677, EPI_ISL_2602678, EPI_ISL_2602680, EPI_ISL_2602681, EPI_ISL_2602684, EPI_ISL_2602685, EPI_ISL_2602686, EPI_ISL_2602687, EPI_ISL_2602688, EPI_ISL_2602689, EPI_ISL_2602690, EPI_ISL_2602693, EPI_ISL_2602694, EPI_ISL_2602695, EPI_ISL_2602696, EPI_ISL_2602697, EPI_ISL_2602698, EPI_ISL_2602699, EPI_ISL_2602700, EPI_ISL_2602706, EPI_ISL_2602707, EPI_ISL_2602714, EPI_ISL_2602719, EPI_ISL_2602724, EPI_ISL_2602730, EPI_ISL_2602746, EPI_ISL_2602749, EPI_ISL_2602753, EPI_ISL_2602755, EPI_ISL_2602761, EPI_ISL_2602994, EPI_ISL_2602996, EPI_ISL_2603010, EPI_ISL_2603036, EPI_ISL_2603038, EPI_ISL_2603039, EPI_ISL_2603040, EPI_ISL_2603041, EPI_ISL_2603043, EPI_ISL_2603044, EPI_ISL_2603045, EPI_ISL_2603047, EPI_ISL_2603048, EPI_ISL_2603049, EPI_ISL_2603050, EPI_ISL_2603051, EPI_ISL_2603052, EPI_ISL_2603053, EPI_ISL_2603054, EPI_ISL_2603055, EPI_ISL_2603060, EPI_ISL_2603063, EPI_ISL_2603065, EPI_ISL_2603066, EPI_ISL_2603067, EPI_ISL_2603068, EPI_ISL_2603069, EPI_ISL_2603070, EPI_ISL_2603072, EPI_ISL_2603074, EPI_ISL_2603075, EPI_ISL_2603078, EPI_ISL_2603079, EPI_ISL_2603082, EPI_ISL_2603085, EPI_ISL_2603086, EPI_ISL_2603088, EPI_ISL_2603089, EPI_ISL_2603090, EPI_ISL_2603091, EPI_ISL_2603093, EPI_ISL_2603095, EPI_ISL_2603096, EPI_ISL_2603098, EPI_ISL_2603100, EPI_ISL_2603103, EPI_ISL_2603104, EPI_ISL_2603105, EPI_ISL_2603106, EPI_ISL_2603108, EPI_ISL_2603111, EPI_ISL_2603113, EPI_ISL_2603115, EPI_ISL_2603118, EPI_ISL_2603119, EPI_ISL_2603120, EPI_ISL_2603121, EPI_ISL_3049405, EPI_ISL_3049442, EPI_ISL_3049488, EPI_ISL_3049489, EPI_ISL_3049497, EPI_ISL_3049498, EPI_ISL_3049501, EPI_ISL_3049569, EPI_ISL_3049580, EPI_ISL_3049583, EPI_ISL_3049593, EPI_ISL_3049594, EPI_ISL_3049599, EPI_ISL_3049603, EPI_ISL_3049614, EPI_ISL_3049616, EPI_ISL_3049620, EPI_ISL_3049623, EPI_ISL_3049636, EPI_ISL_3049655, EPI_ISL_3049708, EPI_ISL_3049715, EPI_ISL_3049731, EPI_ISL_3049734			
see above	KEMRI-Wellcome Trust Research Programme,Kilifi	KEMRI-Wellcome Trust Research Programme,Kilifi	; Githinji G.; Matoke D.; Mburu M.W.; Mohamed K.S.; Onyango C.; Thiongo K.; de Laurent Z.; deLaurent Z.
EPI_ISL_568705, EPI_ISL_568801, EPI_ISL_568803, EPI_ISL_568815, EPI_ISL_806574, EPI_ISL_806575, EPI_ISL_806611, EPI_ISL_806644, EPI_ISL_806665, EPI_ISL_806667, EPI_ISL_806670, EPI_ISL_806701, EPI_ISL_806707, EPI_ISL_806709, EPI_ISL_855535, EPI_ISL_855544, EPI_ISL_968810, EPI_ISL_968882, EPI_ISL_968884, EPI_ISL_968897, EPI_ISL_968911, EPI_ISL_968913, EPI_ISL_968955, EPI_ISL_968998, EPI_ISL_969001, EPI_ISL_969018, EPI_ISL_969074, EPI_ISL_1039228			
see above	KEMRI-Wellcome Trust Research Programme/KEMRI-CGMR-C Kilifi	KEMRI-Wellcome Trust Research Programme/KEMRI-CGMR-C Kilifi	Githinji et al; Githinji et al 2020
EPI_ISL_2779322	KEMRI/DEID Surveillance Site (Isebani Sub-County Hospital)	USAMRD-A, Basic Science Laboratory	Alan Lemtudo; Beth Mutai; Brian Andika; Carol Kifude; Clement Masakwe; Eric Muthanje; Esther Omuseni; Faith Sigei; Gathii Kimita; George Awinda; John Waitumbi; Josphat Nyataya; Rachel Githii; Rehema Liyai; Stephen Ochola
EPI_ISL_3031403, EPI_ISL_3031404, EPI_ISL_3031405	KEMRI/DEID Surveillance Site (Kombewa Sub County Hospital)	USAMRD-A, Basic Science Laboratory	Alan Lemtudo; Beth Mutai; Brian Andika; Carol Kifude; Clement Masakwe; Eric Muthanje; Esther Omuseni; Faith Sigei; Gathii Kimita; George Awinda; John Waitumbi; Josphat Nyataya; Rachel Githii; Rehema Liyai; Stephen Ochola
EPI_ISL_2779374, EPI_ISL_2779375	KEMRI/DEID Surveillance Site (Lamu County Hospital)	USAMRD-A, Basic Science Laboratory	Alan Lemtudo; Beth Mutai; Brian Andika; Carol Kifude; Clement Masakwe; Eric Muthanje; Esther Omuseni; Faith Sigei; Gathii Kimita; George Awinda; John Waitumbi; Josphat Nyataya; Rachel Githii; Rehema Liyai; Stephen Ochola
EPI_ISL_2779376	KEMRI/DEID Surveillance Site (Mtongwe Navy Base Hospital)	USAMRD-A, Basic Science Laboratory	Alan Lemtudo; Beth Mutai; Brian Andika; Carol Kifude; Clement Masakwe; Eric Muthanje; Esther Omuseni; Faith Sigei; Gathii Kimita; George Awinda; John Waitumbi; Josphat Nyataya; Rachel Githii; Rehema Liyai; Stephen Ochola
EPI_ISL_2382192, EPI_ISL_2382203, EPI_ISL_2382205, EPI_ISL_2382206, EPI_ISL_2382208, EPI_ISL_2382209, EPI_ISL_2382211, EPI_ISL_2382212, EPI_ISL_2382215, EPI_ISL_2382223, EPI_ISL_2382699, EPI_ISL_2382700, EPI_ISL_2382701, EPI_ISL_2382709, EPI_ISL_2382712, EPI_ISL_2382717, EPI_ISL_2382718, EPI_ISL_2582843, EPI_ISL_2582848, EPI_ISL_2582868, EPI_ISL_2582872, EPI_ISL_2662665, EPI_ISL_2662668, EPI_ISL_2662670, EPI_ISL_2662671, EPI_ISL_2662672, EPI_ISL_2662673, EPI_ISL_2662680, EPI_ISL_2662683, EPI_ISL_2662691, EPI_ISL_2662694, EPI_ISL_2662695, EPI_ISL_2662696, EPI_ISL_2662700, EPI_ISL_2662704, EPI_ISL_3411584, EPI_ISL_3411765, EPI_ISL_3411767, EPI_ISL_3411773			
see above	KIMBERLEY LABORATORY	National Institute for Communicable Diseases of the National Health Laboratory Service	Amoako DG; Bhiman JN; Everatt J; Ismail A; Mahlangu B; Mnguni A; Mohale T; Ntuli N; Scheepers C
EPI_ISL_3237256, EPI_ISL_3237257, EPI_ISL_3342422, EPI_ISL_3411812	KOPANONG LABORATORY	National Institute for Communicable Diseases of the National Health Laboratory Service	Amoako DG; Bhiman JN; Everatt J; Ismail A; Mahlangu B; Mnguni A; Mohale T; Ntuli N; Scheepers C
EPI_ISL_455629	KRISP, KZN Research Innovation and Sequencing Platform	KRISP, KZN Research Innovation and Sequencing Platform	Chimukangara B; Deforche K; Giandhari J; Lessells R; Pillay S; Tegally H; Wilkinson E; de Oliveira T

EPI_ISL_1094192, EPI_ISL_1312230, EPI_ISL_1322650, EPI_ISL_1382686, EPI_ISL_2403956, EPI_ISL_2403997, EPI_ISL_2404564, EPI_ISL_2404565, EPI_ISL_2404566, EPI_ISL_2404567, EPI_ISL_2404568, EPI_ISL_2404569, EPI_ISL_2404570, EPI_ISL_2425245, EPI_ISL_2832699			
see above	KU Leuven, Rega Institute, Clinical and Epidemiological Virology	KU Leuven, Rega Institute, Clinical and Epidemiological Virology	Bert Vanmechelen; Joan Marti-Carerras; Piet Maes; Tony Wawina-Bokalanga
EPI_ISL_1039041	Kamchatka Regional Children's Infectious Diseases Hospital	WHO National Influenza Centre Russian Federation	Andrey Komissarov; Anna Ivanova; Artem Fadeev; Daria Danilenko; Dmitry Bazhenov; Dmitry Lioznov; Elena Nabieva; Georgii Bazykin; Ksenia Safina; Kseniya Komissarova; Maria Pisareva; Maria Timofeeva; Tamila Musaeva; Veronika Eder
EPI_ISL_1313387	Karolinska University Hospital	Karolinska University Hospital	Annelie Bjerkner; Isak Sylvin; Jan Albert; Karolina Ininbergs; Lina Guerra Blomqvist; Lynda Eneh; Martin Ekman; Martina Wahlund; Robert Dyrdak; Sandra Broddesson; Tanja Normark; Tobias Allander; Valtteri Wirta; Zhibing Yun
EPI_ISL_2648231, EPI_ISL_2648232, EPI_ISL_2648235, EPI_ISL_2648239, EPI_ISL_2648240	Kasane Primary Hospital Laboratory	Botswana Harvard HIV Reference Laboratory	Agnes Karutwaeng; Boitumelo Zuze; Botshelo Radibe; Dorcas Maruapula; Joseph Makhema; Keoratile Ntshambiwa; Legodile Kooepile; Madisa Mine; Mosepele Mosepele; Ontlametse T. Bareng; Roger Shapiro; Rose Munyere; Shahin Lockman; Sikhulile Moyo; Simani Gaseitsiwe; Thongbotho Mphoyakgosi; Wonderful T. Choga
EPI_ISL_512811	Kenema Government Hospital, Ministry of Health and Sanitation	Kenema Government Hospital, Ministry of Health and Sanitation	Andersen, K.; Garry, R.; Goba, A.; Grant, D.; Happi, C.; Jalloh, S.; Mehta, S.; Momoh, M.; Olawoye, I.; Oluniyi, P.; Park, D.; Sandi, J.; Siddle, K.; Tomkins-Tinch, C.
EPI_ISL_1239251, EPI_ISL_1239488	Kimberley Hospital, National Health Laboratory Services, Northern Cape, South Africa	National Institute for Communicable Diseases of the National Health Laboratory Service	Amoako DG; Bhiman JN; Ismail A; Mahlangu B; Mohale T; Ntuli N; Scheepers C
EPI_ISL_489996	King Fahad Medical City	King Fahad Medical City	Alghoraibi, M.; Alosaimi, B.; Enani, M.; Naeem, A.
EPI_ISL_3236930, EPI_ISL_3237049	King Faisal Specialist Hospital & Research Centre	King Faisal Specialist Hospital & Research Centre	Abeer N Alshukairi; Ashraf Dada; Mohamad K Al Hroub; Rayan Bawayan; Waseem A Al Mousa
EPI_ISL_3031398	King'ong'o Dispensary	USAMRD-A, Basic Science Laboratory	Alan Lemtudo; Beth Mutai; Brian Andika; Carol Kifude; Clement Masakwe; Eric Muthanje; Esther Omuseni; Faith Sigei; Gathii Kimita; George Awinda; John Waitumbi; Josphat Nyataya; Rachel Githii; Rehema Liyai; Stephen Ochola
EPI_ISL_2860281	Klinik Primera Clinica	Biosafety Level-3 Laboratory, Indonesian Institute of Sciences (LIPI)	Andri Wardiana; Anik B. Dharmayanthi; Idris; Indriawati; Listiana Oktavia; Masrukhin; Ratih A. Ningrum; Yuliawati
EPI_ISL_2693464	Klinika za infektivne bolesti "Dr. Fran Mihaljević"	Hrvatski zavod za javno zdravstvo	Irena Tabain; Ivana Ferenčak
EPI_ISL_1583298	Klinikum Wels-Grieskirchen	Bergthaler laboratory, CeMM Research Center for Molecular Medicine of the Austrian Academy of Sciences	Andreas Bergthaler; Anna Schedl; Bekir Erguner; Benedikt Agerer; Christoph Bock; Fabian Amman; Jan Laine; Lukas Endler; Maelle Le Moing; Martin Senekowitsch; Michael Schuster; Petr Triska; Thomas Penz
EPI_ISL_1280682	Klinikum der Stadt Ludwigshafen - Institut für Labordiagnostik Hygiene und Transfusionsmedizin	Robert Koch Institute	
EPI_ISL_1300334	Klinisk mikrobiologi	The Public Health Agency of Sweden	Anna Risberg; Anna-Malin Linde; Carlo Berg; Karin Tegmark-Wisell; Maria Lind Karlberg; Mattias Haukland; Mia Brytting; Noura Walai; Oskar Karlsson Lindsjo; Petra Edquist; Petra Holmstrom; Reza Advani; Samuel Ohman; Sofia Stamouli
EPI_ISL_2649991	Kudat Hospital	Institute for Medical Research, Infectious Disease Research Centre, National Institutes of Health, Ministry of Health Malaysia	Azizan MA; Kamel K; Mohd Zawawi Z; Ramly N; Robert F; Suppiah J; Thayan R
EPI_ISL_515182	Kumasi Centre for Collaborative Research in Tropical Medicine, Kumasi.	Institute of Virology, Charité – Universitätsmedizin Berlin	Augustina Sylverken; Christian Drosten; Eric Adu; Jesse Addo Asamoah; Julia Schneider; Jörn Beheim-Schwarzbach; Michael Owusu; Philip El-Duah; Richard Phillips.; Richmond Gorman; Richmond Yeboah; Sherihane Aryeetey; Victor Max Corman
EPI_ISL_2547368	Kuruman Hospital	National Health Laboratory Service/University of Cape Town (NHLS/UCT)	Arash Iranzadeh; Bruna Galvao; Carolyn Williamson; Deelan Doolabh; Diana Hardie; Gert Marais; Innocent Mudau; Lynn Tyers; Marvin Hsiao; Stephen Korsman
EPI_ISL_2779525	LAB-BMC	USAMRD-A, Basic Science Laboratory	Alan Lemtudo; Beth Mutai; Brian Andika; Carol Kifude; Clement Masakwe; Eric Muthanje; Esther Omuseni; Faith Sigei; Gathii Kimita; George Awinda; John Waitumbi; Josphat Nyataya; Rachel Githii; Rehema Liyai; Stephen Ochola
EPI_ISL_1313043	LABORATOIRE ALPIGENE	CNR Virus des Infections Respiratoires - France SUD	Antonin Bal; Bruno Lina; Gregory Destras; Gwendolyne Burfin; Hadrien Regue; Laurence Josset; Martine Valette; Quentin Semanas
EPI_ISL_2629297	LABORATOIRE L'ABO+ LV3	CNR Virus des Infections Respiratoires - France SUD	Antonin Bal; Bruno Lina; Gregory Destras; Gwendolyne Burfin; Hadrien Regue; Laurence Josset; Martine Valette; Quentin Semanas
EPI_ISL_2657871	LABORATORIO CLINICA IMBANACO	Instituto Nacional de Salud- Dirección de Investigación en Salud Pública	Carlos Franco-Muñoz; Carmen Osorio; Diana Malo; Diego A. Álvarez-Díaz; Diego Andrés Prada; Gerardo Santamaría; Hector Alejandro Ruiz-Moreno; Jhonnatan Reales-González; Jorge Rivera; Juan Camilo Martinez; Julian Naizaque; Katherine Laiton-Donato; Lisseth Pardo; Magdalena Wiesner; Marcela Mercado-Reyes; Maria T. Herrera-Sepúlveda; Marta Lopez Blanco; Martha Lucia Ospina Martinez; Paola Rojas; Sergio Gomez; Sheryll Corchuelo; Ángela Alarcon Cruz
EPI_ISL_2272979	LABORATORIO CLINICO LABIN	Inciensa, Instituto Costarricense de Investigación y Enseñanza en Nutrición y Salud	Adriana Godínez; Claudio Soto-Garita; Estela Cordero; Francisco Duarte; Hebleen Porras; Joselyn Prado & Pei Ling Chan Ma; José Luis Vargas; Mariela Gutiérrez; Melany Calderón
EPI_ISL_1403498	LBA Casteljaloux	Cerba Lab	Benazra M; Haïm-Boukobza S; Lecorche E; Olivi M; Roquebert B; Trombert-Paolantoni S; Zimmer S
EPI_ISL_1253552, EPI_ISL_1253553, EPI_ISL_1253554, EPI_ISL_1287741, EPI_ISL_1287742, EPI_ISL_1287743, EPI_ISL_1287744, EPI_ISL_1287745, EPI_ISL_1287746, EPI_ISL_1287747, EPI_ISL_1287749, EPI_ISL_1287750, EPI_ISL_1287751, EPI_ISL_1312994, EPI_ISL_1313000, EPI_ISL_1313001, EPI_ISL_1314035, EPI_ISL_1314039, EPI_ISL_1935386			
see above	LBM UNIBIO VALENTIN	CNR Virus des Infections Respiratoires - France SUD	Antonin Bal; Bruno Lina; Gregory Destras; Gwendolyne Burfin; Hadrien Regue; Laurence Josset; Martine Valette; Quentin Semanas
EPI_ISL_2674296	LDSP CALDAS	Instituto Nacional de Salud- Dirección de Investigación en Salud Pública	Carlos Franco-Muñoz; Carmen Osorio; Diana Malo; Diego A. Álvarez-Díaz; Diego Andrés Prada; Gerardo Santamaría; Hector Alejandro Ruiz-Moreno; Jhonnatan Reales-González; Jorge Rivera; Juan Camilo Martinez; Julian Naizaque; Katherine Laiton-Donato; Lisseth Pardo; Magdalena Wiesner; Marcela Mercado-Reyes; Maria T. Herrera-Sepúlveda; Marta Lopez Blanco; Martha Lucia Ospina Martinez; Paola Rojas; Sergio Gomez; Sheryll Corchuelo; Ángela Alarcon Cruz
EPI_ISL_2533816	LESP Campeche	Instituto de Diagnostico y Referencia Epidemiologicos (INDRE)	Abril Rodriguez-Maldonado; Ariadna Medina-Benitez; Claudia Wong-Arambula; Ernesto Ramirez-Gonzalez.; Gisela Barrera-Badillo; Irma Lopez-Martinez; Joaquin Quiroz-Mercado; Lucia Hernandez-Rivas; Natividad Cruz-Ortiz; Sergio Rangel-Guerrero; Tatiana Nunez-Garcia; Vanessa Rivero-Arredondo
EPI_ISL_1516769	LESP Nuevo Leon	Instituto de Diagnostico y Referencia Epidemiologicos (INDRE)	Abril Rodriguez-Maldonado; Ariadna Medina-Benitez; Claudia Wong-Arambula; Ernesto Ramirez-Gonzalez.; Gisela Barrera-Badillo; Irma Lopez-Martinez; Joaquin Quiroz-Mercado; Lucia Hernandez-Rivas; Natividad Cruz-Ortiz; Sergio Rangel-Guerrero; Tatiana Nunez-Garcia; Vanessa Rivero-Arredondo
EPI_ISL_2246840	LESP Tamaulipas	Instituto de Diagnostico y Referencia Epidemiologicos (INDRE)	Abril Rodriguez-Maldonado; Ariadna Medina-Benitez; Claudia Wong-Arambula; Ernesto Ramirez-Gonzalez.; Gisela Barrera-Badillo; Irma Lopez-Martinez; Joaquin Quiroz-Mercado; Lucia Hernandez-Rivas; Natividad Cruz-Ortiz; Sergio Rangel-Guerrero; Tatiana Nunez-Garcia; Vanessa Rivero-Arredondo
EPI_ISL_482738	LNR National Reference Laboratory, Mohammed VI University of Health Sciences	Medical Biotechnology Laboratory, Rabat Medical and Pharmacy School, Mohammed The Vth University in Rabat	Chakib NEJJARI; Houda BENRAHMA; Idrissa DIAWARA; Imane SMYEJ; Jalil El ATAR; Jalila RAHOUI; Lahcen BELYAMANI and Azeddine IBRAHIMI; Laila SBABOU; Loubna ALLAM; M.W. CHEMAO-ELFIHRI; Meriem LAAMARTI; Mouna OUADGHIRI; Rachid EL JAOUDI; Rachid MENTAG; Rokia LAAMARTI; Saaid AMZAZI; Souad KARTTI
EPI_ISL_1313015	LX BIO	CNR Virus des Infections Respiratoires - France SUD	Antonin Bal; Bruno Lina; Gregory Destras; Gwendolyne Burfin; Hadrien Regue; Laurence Josset; Martine Valette; Quentin Semanas
EPI_ISL_2617531	Lab Genomik Solidaritas Indonesia	National Institute of Health Research and Development	Arie Ardiansyah Nugraha; Hana Apsari Pawestri; Hartanti Dian Ikawati; Kartika Dewi Puspa; Krisna Pangesti; Meutia Kumaheri; Nelly Puspandari; Subangkit; Triyani Soekarso; Vivi Setiawaty
EPI_ISL_1273195	Lab voor klinische biologie	Lab voor klinische biologie	Bruno Verhasselt; Hannelore Hamerlinck; Marija Janevska
EPI_ISL_548119, EPI_ISL_579114, EPI_ISL_579425, EPI_ISL_877221, EPI_ISL_877226, EPI_ISL_1621314, EPI_ISL_1904849, EPI_ISL_1904853, EPI_ISL_2406490			
see above	LabPLUS	Institute of Environmental Science and Research (ESR)	Anja Werno; Antje van der Linden; Arlo Upton; Chris Mansell; David Hammer; Dragana Drinkovic; Erasmus Smit; Gary McAuliffe; Hana Sofia Andersson; Hermes Perez; James Ussher; Jill Sherwood; Jing Wang; Joep de Ligt; Josh Freeman; Julia Howard; Juliet Elvy; Lauren Jelly; Mary DeAlmeida; Matt Blakiston; Matt Storey; Matthew Rogers; Max Bloomfield; Michael Addidle; Michelle Balm; Muhammad Faisal; Nikki Freed; Olin Silander; Olivia Stroeven; Rachel Boyle; Sally Roberts; SallyAnn Harbison; Sarah Jefferies; Sharmini Muttaiyah; Susan Morpeth; Susan Taylor; Timothy Blackmore; Vani Sathyendran; Veronica Playle; Virginia Hope; Xiaoyun Ren
EPI_ISL_2854726	Labkesda Kota Tangerang	National Institute of Health Research and Development	; Arie Ardiansyah Nugraha; Hana Apsari Pawestri; Hartanti Dian Ikawati; Kartika Dewi Puspa; Krisna Pangesti; Nelly Puspandari; Subangkit; Triyani Soekarso; Vivi Setiawaty
EPI_ISL_1118902, EPI_ISL_1118903, EPI_ISL_1118904, EPI_ISL_1118905, EPI_ISL_1118906, EPI_ISL_1118907, EPI_ISL_1118908, EPI_ISL_1118909, EPI_ISL_1219949, EPI_ISL_1219950, EPI_ISL_1259298, EPI_ISL_1259301, EPI_ISL_1259302, EPI_ISL_1259303, EPI_ISL_1336331, EPI_ISL_1336332, EPI_ISL_1336333, EPI_ISL_1443910, EPI_ISL_1623013, EPI_ISL_1739306, EPI_ISL_1739316, EPI_ISL_2029888, EPI_ISL_2029936, EPI_ISL_2178452, EPI_ISL_2178453, EPI_ISL_2178454, EPI_ISL_2178455, EPI_ISL_2178456, EPI_ISL_2178457, EPI_ISL_2178458, EPI_ISL_2178459, EPI_ISL_2178460, EPI_ISL_2178461, EPI_ISL_2178462, EPI_ISL_2178463, EPI_ISL_2259091, EPI_ISL_2293623, EPI_ISL_2709261			
see above	Labo Analyses Med	National Reference Center for Viruses of Respiratory Infections, Institut Pasteur, Paris	Amaury Vaysse; Angela Brisebarre; Bonnet Cyrile; Brieuc Gestin; Camille Capel; Christophe Malabat; Coignard Catherine; Corinne Maufrais; Damien Mornico; Ducancelle Alexandra; Durivault JéRôMe; Ebel Anne; Etienne Simon-Lorière; Fabienne Artur; Frédéric Lemoine; Holstein Anne; Jonas Amzalag; Le Berre David; Le Vicky; Louise Lefrançois; Mallet C; Marion Barbet; Maud Vanpeene; Melanie Caron; Méline Bizard; OphéLie Said-Delattre; Patricia Tamby; Pierre Lechat; Sylvie Behillil; Sylvie Van der Werf; Sylvie van der Werf; Vincent Enouf
EPI_ISL_1840660, EPI_ISL_1885872, EPI_ISL_1885882, EPI_ISL_1885939, EPI_ISL_1905080, EPI_ISL_1905093, EPI_ISL_1905100, EPI_ISL_1905104, EPI_ISL_1905107, EPI_ISL_1905115, EPI_ISL_1905116, EPI_ISL_1982504, EPI_ISL_1986473			
see above	Labor Berlin Charité Vivantes GmbH / Institut für Virologie	Charité Universitätsmedizin Berlin, Institut für Virologie/Labor Berlin	Barbara Mühlemann; Christian Drosten; Christine Stephan; Peter Menzel; Rolf Schwarzer; Terry Jones; Victor M Corman
EPI_ISL_2384799	Labor Dr. Fenner und Kollegen	Heinrich Pette Institute, Leibniz Institute for Experimental Virology	Adam Grundhoff; Alexis Robitaille; Johannes Knobloch; Martin Aepfelbacher; Nicole Fischer; Thomas Günther
EPI_ISL_2125112	Labor Prof. Dr. G. Enders MVZ GbR	Robert Koch Institute	
EPI_ISL_1502112, EPI_ISL_1508849, EPI_ISL_1508890, EPI_ISL_1508894, EPI_ISL_1508916, EPI_ISL_1508949, EPI_ISL_1508959, EPI_ISL_1508990, EPI_ISL_1509102, EPI_ISL_1524717, EPI_ISL_1575442, EPI_ISL_1575443, EPI_ISL_1575444, EPI_ISL_1575479, EPI_ISL_1586897, EPI_ISL_1586899, EPI_ISL_1586900, EPI_ISL_1586901, EPI_ISL_1675094, EPI_ISL_1675141, EPI_ISL_1810924, EPI_ISL_1810927			
see above	Laboratoire Biolim/FSS/UL	Unité Mixte Internationale TransVIHMI (UMI 233 IRD – U1175 INSERM - Université de Montpellier) IRD (Institut de recherche pour le développement)	Abla A. KONOU; Adodo SADJI; Ahidjo AYOUBA; Akoélé SILIADIN; Alassane OURO-MEDELI; Amivi EHLAN; Améyo DORKENOO; Anoumou DAGNRA; Christelle BUTEL; Déléma MABA; Eric DELAPORTE; Issaka Maman; Kokou TEGUENI; Koku AGBODEKA; Laetitia SERRANO; Martine PEETERS; Messanh DOUFFAN; Mireille PRINCE-DAVID; Mounerou SALOU; Sidonie A.M.KAGNISSODE; Sika DOSSIM; Syntiche DEVATCHAGNI Adodo SADJI; Syntyche DEVATCHAGNI; Wembo A. HALATOKO
EPI_ISL_1754272	Laboratoire CBM 25 TERRE ROUGE	Department of Virology, Henri Mondor University Hospital, Assistance Publique Hôpitaux de Paris, Université Paris-Est Créteil, INSERM U955	Alexandre Soulier; Christophe Rodriguez; Elisabeth Trawinski; Guillaume Gricourt; Jean-Michel Pawlotsky; Melissa N'Debi; Slim Fourati; Vanessa Demontant
EPI_ISL_1905079, EPI_ISL_2318037	Laboratoire Central de Virologie	Laboratoire de Biotechnologie	Abdelomunim Essabbar; Amal Zouaki; Ghizlane EL Amin; Hakima Kabbaj; Lahcen Belyamani and Azeddine Ibrahimi; Mouna Ouadghiri; Myriam Seffar; Naima El Hafidi; Saaïd Amzazi; Tarik Aanniz
EPI_ISL_2978186, EPI_ISL_2978188, EPI_ISL_2978190, EPI_ISL_2978194, EPI_ISL_2978196, EPI_ISL_2978197, EPI_ISL_2978199, EPI_ISL_2978206, EPI_ISL_2978212, EPI_ISL_2978214, EPI_ISL_2978217, EPI_ISL_2978221, EPI_ISL_2978223, EPI_ISL_2978224, EPI_ISL_2978225, EPI_ISL_2978226, EPI_ISL_2978228, EPI_ISL_2978236			
see above	Laboratoire National de Santé Publique, Brazzaville, République du	Pathogen Genomics Lab, National Institute for Biomedical Research (INRB)	Amuri Aziza; Andrew Rambaut; Catherine Pratt; Eddy Kinganda-Lusamaki; Edith Nkwembe; Emmanuel Lokilo Lofiko; Fabien Roch Niama; Francisca Muyembe Mawete; Gabriel Kabamba; Ian Goodfellow; Jean Claude Makangara; Jean-Jacques Muyembe Tamfum; Josh Quick; Matthias Pauthner; Michael Wiley; Nick Loman; Placide Mbala-Kingebeni; Raphael Lumembe; Steve Ahuka-Mundeke; Trevor Bedford

	Congo		
EPI_ISL_2686246	Laboratoire National de Référence pour les Fièvres Hémorragiques Virales, Centre Muraz	Centre Muraz	Ange Badjo; Arsène Zongo; Essia Belarbi; Fabian Leendertz; Grit Schubert; Jasmin Schlotterbeck; Saïdou Ouedraogo; Soumeya Ouangraoua; Thérèse Kagone
EPI_ISL_1972308	Laboratoire National de Santé Publique du Cameroun	Pathogen Genomics Lab, National Institute for Biomedical Research (INRB)	Amuri Aziza; Andrew Rambaut; Catherine Pratt; Eddy Kinganda-Lusamaki; Edith Nkwembe; Emmanuel Lokilo Lofiko; Francisca Muyembe Mawete; Gabriel Kabamba; Ian Goodfellow; Jean Claude Makangara; Jean-Jacques Muyembe Tamfum; Josh Quick; Marie Claire Okomo; Matthias Pauthner; Michael Wiley; Nick Loman; Placide Mbala-Kingebeni; Raphael Lumembe; Steve Ahuka-Mundeke; Trevor Bedford
EPI_ISL_2157559, EPI_ISL_2157562, EPI_ISL_2157568, EPI_ISL_2492505, EPI_ISL_2492530	Laboratoire National de Santé Publique – LNSP (HAITI - LNSP)	Laboratory of Respiratory Viruses and Measles, Oswaldo Cruz Institute, FIOCRUZ	Alice Sampaio Rocha; Ana Carolina Mendonca; Anna Carolina Paixao; Elisa Cavalcante Pereira; Fernando Motta; Ito Journel; Jaques Boncy; Luciana Appolinario; Marilda Siqueira on behalf of the Fiocruz COVID-19 Genomic Surveillance Network; Paola Resende; Patrick Delly; Renata Serrano Lopes; Taina Venas
EPI_ISL_3360861	Laboratoire Professeur Daniel GAHOUMA (LPDG)	Centre de Recherches Médicales de Lambaréné (CERMEL)	Ayong More; Bertrand Lell; Davy Leger Mouangala; Elvyre Mbongo-Nkama; Emilio Skarwan; Georgelin Nguema Ondo; Guy Stéphane Padzys; Gédéon Prince Manouana; Jean Bernard Lekana-Douki; Joël-Fleury Djoba Siawaya and Ayola Akim Adegnika; Kevine Zang Ella; Ludovic Mewono; Moustapha Nzamba Maloum; Noé Patrick Mbondoukwe; Rodrigue Bikangui; Rodrigue Mintsa Nguema; Sam O'neilla Oye Bingono; Samira Zoa Assoumou; Sandrine Zeh Nfor; Srinivas reddy Pallerla; Steffen Borrmann; Thirumalaisamy P. Velavan
EPI_ISL_1760554	Laboratoire Professeur Daniel GAHOUMA (LPDG)	Centre de recherches médicales de Lambaréné (CERMEL)	Ayola A. Adegnika; Ayong Moure; Bertrand Lell; Bénédicte Ndeboko; Emilio Skarwan; Georgelin Nguema Ondo; Gédéon P. Manouana; Haruka Abe; Jiro Yasuda; Joel Fleury Djoba Siawaya; Rodrigue Bikangui; Rotimi Myrabelle Avome Houechenou; Samira Zoa-Assoumou; Yuri Ushijima
EPI_ISL_1103576, EPI_ISL_1116470, EPI_ISL_1159697, EPI_ISL_1159698, EPI_ISL_1810938, EPI_ISL_1810947, EPI_ISL_1904692, EPI_ISL_1904846, EPI_ISL_1904876, EPI_ISL_1904886, EPI_ISL_1904887			
see above	Laboratoire central de Virologie	Laboratoire de Biotechnologie	Abdelmunim Essabbar; Amal Zouaki; Ghizlane EL Amin; Hakima Kabbaj; Lahcen Belyamani and Azeddine Ibrahimi; Mouna Ouadghiri; Myriam Seffar; Saaïd Amzazi; Tarik Aanniz
EPI_ISL_2227197, EPI_ISL_2227198, EPI_ISL_2227199, EPI_ISL_2227200, EPI_ISL_2227201, EPI_ISL_2227202, EPI_ISL_2227203			
see above	Laboratoire d'analyse biomédicale du camp Ouezzin Coulibaly	Centre Muraz	Abdoul-Salam Ouedraogo; Amariane Koné; Armel Poda; Arsène Zongo; Essia Belarbi; Fabian Leendertz; Grit Schubert; Halidou Tinto; Soumeya Ouangraoua; Thérèse Kagoné; Yacouba Sawadogo; Zekiba Tarnagda
EPI_ISL_660446, EPI_ISL_660448, EPI_ISL_660450, EPI_ISL_660451, EPI_ISL_660452, EPI_ISL_660481, EPI_ISL_660493, EPI_ISL_660503, EPI_ISL_660509, EPI_ISL_2142708, EPI_ISL_2142743			
see above	Laboratoire de Microbiologie CHU Souro Sanou	Centre Muraz	Abdoul-Salam Ouedraogo; Amariane Koné; Armel Poda; Arsène Zongo; Essia Belarbi; Fabian Leendertz; Grit Schubert; Halidou Tinto; Lassana Sangaré; Soumeya Ouangraoua; Thérèse Kagoné; Yacouba Sawadogo; Zekiba Tarnagda
EPI_ISL_2289102, EPI_ISL_2289104, EPI_ISL_2289107, EPI_ISL_2289121, EPI_ISL_2289123	Laboratoire de Microbiologie CHU Souro Sanou	Laboratoire bacteriologie virologie CHUSS	Abdoul-Salam Ouedraogo; Abdoulie Kanteh; Abdul Sesay; Annette Erhart; Armel Poda; François Kiemdé; Halidou Tinto; Mariama Kujabi; Yacouba Sawadogo
EPI_ISL_1116464, EPI_ISL_1116467, EPI_ISL_1116468, EPI_ISL_1118675	Laboratoire de Microbiologie- CHU Habib Bourguiba – Sfax	Laboratoire des Procédés de Criblage Moléculaire et Cellulaire- Centre de Biotechnologie de Sfax	A. and Masmoudi, S.; Abdelmoulah, F.; Abid, N.; Ajili, F.; Aouni, M.; Ben Ayed, I.; Bensaid, M.; Chtourou, A.; Elargoubi, A.; Fki-berrajah, L.; Gaaloul, I.; Gargouri, S.; Hammami, A.; Kamoun, S.; Karray Hakim, H.; Kharat, N.; Mastouri, M.; Mhalla, S.; Nabli, A.; Rebai; Smeti, I.; Souissi, A.; Stambouli, N.; Turki, M.
EPI_ISL_1241662, EPI_ISL_1403098, EPI_ISL_3143818	Laboratoire de santé publique du Québec	Laboratoire de santé publique du Québec	Guillaume Bourque; Ioannis Ragoussis; Jesse Shapiro; Mark Lathrop and Michel Roger on behalf of the CoVSeQ research group; Mark Lathrop and Michel Roger on behalf of the CoVSeQ research group (http://covseq.ca/researchgroup); Sandrine Moreira
EPI_ISL_1207287, EPI_ISL_1208160, EPI_ISL_1208398, EPI_ISL_1208399	Laboratoire de virologie clinique - Institut Pasteur de Tunis	1-Laboratory of Microbiology, National Reference Lab, Charles Nicolle Hospital; 2-University of Tunis ElManar, Faculty of Medicine of Tunis, LR99ES09, Tunis, Tunisia	Alia BenKahla; Anissa Chouikha; Fares Wasfi; Henda Triki; Ilhem Boutiba-Ben Boubaker.; Imen Kacem; Ines Mdini; Jalila Ben Khelil; Maher Kharrat; Manel Ben Sassi; Mariem Gdoura; Mouna Ben Sassi; Mouna Safer; Nissaf Ben Alaya; Riadh Daghfous; Riadh Gouider; Roua Ben Othman; Salma Abid; Sameh Trabelsi; Sana Ferjani; Sara Chammam; Sondes Haddad
EPI_ISL_476825	Laboratoire des Fièvres Hémorragiques Virales du Benin	Charité-Universitätsmedizin Berlin	Anges; Drexler; Jan Felix; Moreira-Soto Andres; Sander Anna-Lena; Yadouleton
EPI_ISL_2932537, EPI_ISL_2932558, EPI_ISL_2932562, EPI_ISL_2958658, EPI_ISL_2958659, EPI_ISL_2958660, EPI_ISL_2958661, EPI_ISL_2958663, EPI_ISL_2958665, EPI_ISL_2958666, EPI_ISL_2958667, EPI_ISL_2958668, EPI_ISL_2958669, EPI_ISL_2958670, EPI_ISL_2958671			
see above	Laboratoire des Fièvres Hémorragiques Virales du Benin	Institut für Virologie - Institute of Virology - Charite	Andres Moreira-Soto; Anges Yadouleton; Anna-Lena Sander; Benjamin Hounkpatin and Jan Felix Drexler; Benjamin Hounkpatin and Jan Felix Drexler; Carine Tchibozo; Christian Drosten; Dossou Ange; Eclou Sedjro; Edmilson F de Oliveira Filho; Gildas Hounkanrin; Hinson Fidelia; Keke K. René; Mamoudou Harouna Djingarey; Melchior A. Joël Aïssi; Michael Nagel; Olfert Landt; Praise Adewumi; Salifou Sourakatou; Victor Max Corman; Wendy Karen Jo; Yvette Badou
EPI_ISL_1383629, EPI_ISL_1383630, EPI_ISL_1918209	Laboratoire national de sante, Microbiology, Virology	Laboratoire national de sante, Microbiology, Microbial Genomics Platform	Anke Wienecke-Baldacchino; Catherine Ragimbeau; Fatu Djabi; Jessica Tapp; Lise Pignon; Raoul Salmon; Tamir Abdelrahman; Trung Nguyen Nguyen
EPI_ISL_740028, EPI_ISL_744958, EPI_ISL_745024	Laboratoire national de santé, Microbiology, Virology	Laboratoire national de santé, Microbiology, Microbial Genomics Platform	Anke Wienecke-Baldacchino; Catherine Ragimbeau; Fatu Djabi; Jessica Tapp; Lise Pignon; Raoul Salmon; Tamir Abdelrahman
EPI_ISL_2400636	Laboratoires d'analyses medicales - Ketterthill	Laboratoire national de sante, Microbiology, Microbial Genomics Platform	Anke Wienecke-Baldacchino; Caroline Scheiber; Catherine Ragimbeau; Fatu Djabi; Jessica Tapp; Lise Pignon; Raoul Salmon; Serge Vedy; Tamir Abdelrahman
EPI_ISL_1719751	Laboratori Clinic Territorial de Girona	Can Ruti SARS-CoV-2 Sequencing Hub (HUGTiP/IrsiCaixa/IGTP)	Alba Sanchez; Anna Not; Antoni E Bordoy; Bonaventura Clotet; Cristina Casan; Cristina Esteban; Francesc Catala-Moll; Gemma Clara; Ignacio Blanco; Marc Noguera-Julian; Maria Casadella; Mariona Parera; Mercedes Guerrero; Montserrat Gimenez; Pere-Joan Cardona; Pilar Armengol; Roger Paredes; Veronica Saludes; and Elisa Martro on behalf of the Can Ruti SARS-CoV-2 Sequencing Hub.
EPI_ISL_1831644, EPI_ISL_1892959, EPI_ISL_2000095, EPI_ISL_2000110	Laboratori Clínic Territorial de Girona	Can Ruti SARS-CoV-2 Sequencing Hub (HUGTiP/IrsiCaixa/IGTP)	Alba Sánchez; Anna Not; Antoni E Bordoy; Bonaventura Clotet; Cristina Casañ; Cristina Esteban; Francesc Catala-Moll; Gemma Clara; Ignacio Blanco; Marc Noguera-Julian; Maria Casadellà; Mariona Parera; Mercedes Guerrero; Montserrat Giménez; Pere-Joan Cardona; Pilar Armengol; Roger Paredes; Verónica Saludes; and Elisa Martró on behalf of the Can Ruti SARS-CoV-2 Sequencing Hub.
EPI_ISL_981033	Laboratorio Central Sección Virología. Hospital Regional Artemides Zatti (Viedma, Rio Negro); Laboratorio MICROBIOM (General Roca, Río Negro); Hospital Zonal Dr. Ramón Carrillo (San Carlos De Bariloche, Río Negro)	Laboratorio Central Mg. Luis Alfredo Pianciola on behalf of 'Proyecto Argentino Interinstitucional de genomica de SARS-CoV-2' (PAIS Consortium)	Antonela De Fino; C Pintos; C Ziehm; Darío Fabián di Prátula; J Ousset; L Pianciola.; Liliana Fonseca; M Fernandez; M Mazzeo; M Nabaes; Marcela Nóbile; María Laura Álvarez; Patricia Valeria Blanco; Silvana Cecchi; Yesica Espasandin
EPI_ISL_2681213	Laboratorio Central de Epidemiología (LCE)	Instituto de Biotecnología de la UNAM	; Alejandra García-Gasca; Alejandra Hernández-Terán; Alejandro Sánchez-Flores; Alfredo Herrera-Estrella; Alicia Ocaña-Mondragón; Andreu Comas-García; Angel Gustavo Salas-Lais; Antonio Loza Román; Bernardo Martínez-Miguel; Blanca Taboada; Brenda Irasema Maldonado-Meza; Bruno Gómez-Gil; Carla Ivón Herrera-Najera; Carlos F. Arias; Celia Boukadida; Clara Esperanza Santacruz-Tinoco; Concepción Grajales-Muñiz; Consorcio Mexicano de Vigilancia Genómica (CoViGen-Mex). Authors (in alphabetical order): Julio Elias Alvarado-Yaah; Cristóbal Cháidez-Quiróz; Célida Duque Molina; Célida Martínez- Rodríguez; Daniel Fregoso-Rueda; Daniel Lira Morales; Eduardo Becerril-Vargas; Fernando Fontove-Herrera; Fidencio Mejía-Nepomuceno; Francisco Pulido; Gloria Elena Espinosa-Ayala; Gloria María Molina-Salinas; Gloria Vazquez; Hector Esteban Paz-Juárez; Hector Montoya-Fuentes; Helen Haydee Fernanda Ramírez-Plascencia; Irvin González-López; Jean Pierre González; Jesús Hernández; Joel Armando Vázquez-Pérez.; Jorge Salas-Hernández; José Antonio Enciso-Moreno; José Arturo Martínez-Orozco; José Esteban Muñoz-Medina; José de Jesús Nuñez-Contreras; Juan Bautista Chale-Dzul; Julissa Enciso-Ibarra; Luis Alberto Ochoa-Carrera; Margarita Matías-Florentino; Mario Mújica-Sánchez; Marissa Perez-Garcia; María Guadalupe Santiago-Mauricio; María Guadalupe de Jesús Mireles-Rivera; Nelly Sélem-Mojica; Pavel Isa; Ricardo Ciria Merce; Ricardo Grande; Rosa María Gutiérrez Rios; Santiago Ávila-Ríos; Selene Zárate; Susana Lopez; Verónica Mata-Haro; Victor Eduardo García-Arias; Víctor Hugo Borja-Aburto
EPI_ISL_1492647	Laboratorio Central de Salud Publica de Paraguay	Laboratorio Central de Salud Publica de Paraguay	Andrea Gómez de la Fuente; Cynthia Vázquez; Flavia Aburjaile; Juan Torales; Luiz Carlos Junior Alcantara; Marta Giovanetti; María José Ortega; María Liz Gamarra; Shirley Villalba; Talita Adelino; Vagner Fonseca
EPI_ISL_2660689	Laboratorio Central de Saude Publica do Estado de Sergipe (LACEN/SE)	Laboratory of Respiratory Viruses and Measles, Oswaldo Cruz Institute, FIOCRUZ	Alice Sampaio Rocha; Ana Carolina Mendonca; Anna Carolina Paixao; Cliomar Alves dos Santos; Elisa Cavalcante Pereira; Fernando Motta; Luciana Appolinario; Marilda Siqueira on behalf of the Fiocruz COVID-19 Genomic Surveillance Network; Paola Resende; Renata Serrano Lopes; Tainá Moreira Martins Venas
EPI_ISL_792541, EPI_ISL_2940267	Laboratorio Central, Ministerio de Salud Córdoba	Instituto de Patologia Vegetal (CIAP-INTA) on behalf of 'Proyecto Argentino Interinstitucional de genomica de SARS-CoV-2' (PAIS Consortium)	Barbas, G.; Castro, G.; Debat, HJ.; FD; Fernández; M; M.B.; MB; Marquez, N.; Pisano; Re; Re, V.; V
EPI_ISL_750163	Laboratorio DILAVE/MGAP-INIA-UdelaR -Tacuarembó	Institut Pasteur de Montevideo	Ana Carolina Mendonça; Andrés Lizasoain; Camila Simoes; Cecilia Alonso; Cecilia Salazar; Daiana Mir; Fernando López-Tort; Fernando Motta; Gonzalo Bello; Ighor Arantes; Ignacio Ferrés; Jose Sotelo; Leticia Maya; Letícia Garay Martins; Luciana Appolinario; Lucía Spangenberg; Mailen Arleo; Mariana Brandes; Marilda Mendonça Siqueira; Marilda Tereza Mar da Rosa; María José Benitez-Galeano; Martín Graña; Matías Castells; Matías Victoria; Matías Salvo; Natalia Rego; Natalia Reyes; Pablo Smircich; Paola Cristina Resende; Rodney Colina; Tamara Fernandez-Calero; Tania Possi; Tatiana Schäffer Gregianini; Verónica Noya; Yasser Vega
EPI_ISL_1525249, EPI_ISL_1647346, EPI_ISL_1654000, EPI_ISL_1706550, EPI_ISL_1821763, EPI_ISL_2107000	Laboratorio HUB -Azienda Ospedaliero Universitaria - AOU – Cagliari	Laboratorio SPOKE Biologia Molecolare -Azienda Ospedaliero Universitaria - AOU – Cagliari	Alessandra Scano; Ferdinando Coghe; Germano Orrù; Miriam Loddo; Riccardo Cappai; Sara Fais; Valentina Medda
EPI_ISL_2244910, EPI_ISL_2244911, EPI_ISL_2244912	Laboratorio HUB -Azienda Ospedaliero Universitaria - AOU – Cagliari, Italy	Department of Infectious Diseases, Istituto Superiore di Sanità	Alessandra Lo Presti; Angela Di Martino; Ferdinando Coghe; Germano Orrù; Manuela Marra; Marco Crescenzi; Maria Carollo; Paola Stefanelli; Riccardo Cappai; Sara Fais; Stefano Fiore
EPI_ISL_3333121	Laboratorio Nacional de Salud	Asociación de Salud Integral/Clínica Familiar "Luis Ángel García"	Ana S. Gonzalez-Reiche; Claudia Rangel; Danicela Mercado; Eduardo Arathoon; Hilda Ruiz; Luis Aguirre; Luis Rivas; Narda Medina; Oscar Bonilla; Osmar Gamboa
EPI_ISL_2825088, EPI_ISL_2825105, EPI_ISL_2827828	Laboratorio Nacional de Salud	Laboratorio Nacional de Salud	Gabriela García; Linda Mendoza

EPI_ISL_837589, EPI_ISL_837595	Laboratorio Nacional de Salud	Laboratory of Respiratory Viruses and Measles, Oswaldo Cruz Institute, FIOCRUZ	Ana Carolina Mendonca; Anna Carolina Paixao; Cesar Roberto Conde Pereira; Claudia Estrada; Fernando Motta; Luciana Appolinario; Marilda Siqueira on behalf of the Fiocruz COVID-19 Genomic Surveillance Network; Paola Resende
EPI_ISL_3045410	Laboratorio Nacional de Salud Pública Dr. Defilló - LNSPDD	Laboratory of Respiratory Viruses and Measles, Oswaldo Cruz Institute, FIOCRUZ	Alice Sampaio Rocha; Ana Carolina Mendonca; Anna Carolina Paixao; Elisa Cavalcante Pereira; Fernando Motta; Grey Benoit Vasquez; Isaac Miguel Sanchez; Ivonne Imbert; Lucia de la Cruz; Luciana Appolinario; Marilda Siqueira on behalf of the Fiocruz COVID-19 Genomic Surveillance Network; Nurys de Castro; Paola Resende; Renata Serrano Lopes; Ronald Skewes; Taina Venas
EPI_ISL_3275179, EPI_ISL_3275192, EPI_ISL_3275198, EPI_ISL_3275200, EPI_ISL_3275218, EPI_ISL_3275234, EPI_ISL_3275248			
see above	Laboratorio Nacional de Salud, Ministerio de Salud Publica y Asistencia Social	Genomics and Proteomics Departament, Gorgas Memorial Institute For Health Studies	Alexander A Martinez; Ambar Moreno; Claudia Estrada; Claudia Gonzalez V; César Roberto Conde Pereira; Jessica Gondola; Leyda Abrego; Marlene Castillo; Oris Chavarria
EPI_ISL_2648258, EPI_ISL_2650524, EPI_ISL_2650533	Laboratorio Nacional de Vigilancia de la Salud – Sección de Virologia	Genomics and Proteomics Departament, Gorgas Memorial Institute For Health Studies	Alexander Martinez; Ambar Moreno; Claudia Díaz; Claudia Gonzalez; Elda Martínez; Jessica Gondola; Leyda Abrego; Marlene Castillo; Mitzi Castro; Oris Chavarria; Sandra Paola Paz; Sofia Carolina Alvarado
EPI_ISL_1789698	Laboratorio PGM	Laboratorio de Infectología Molecular, Departamento de Bioquímica y Medicina Molecular,Facultad de Medicina - Universidad Autónoma de Nuevo León	Ana M. Rivas-Estilla; Daniel Arellanos-Soto; Eduardo Garza-de-la-Peña; Gabriela Elizondo; Javier Ramos-Jimenez; Kame A. Galán-Huerta; María F. Herrera-Saldivar; Natalia Martínez-Acuña; Sonia A. Lozano-Sepúlveda
EPI_ISL_2462063	Laboratorio de Biologia Molecular, Hospital San Pedro Claver	Microbiologia Molecular, Instituto SELADIS, Universidad Mayor de San Andrés	Aneth Vasquez Michel; Carmen Delgado Barrera; Oscar M. Rollano-Peñaloza; Sandra Miranda Sardon
EPI_ISL_2600378	Laboratorio de Biologia Molecular, Hospital San Pedro Claver	Molecular Genetics Laboratory, Instituto de Investigaciones Químicas, Universidad Mayor de San Andrés	Aneth Vasquez Michel; Carmen Delgado Barrera; Oscar M. Rollano-Peñaloza; Sandra Miranda Sardon
EPI_ISL_2427736, EPI_ISL_2427766	Laboratorio de Biología Molecular Médica Uruguaya	Departments of Pathology and Medicine, New York University School of Medicine	Adriana Heguy; Cecilia Sorhouet; Christian Marier; Dacia Dimartino; Gonzalo Manrique; Maria Cristina Mogdasy; Maria Noel Zubillaga; Maria Victoria Elizondo; Paul Zappile
EPI_ISL_953404, EPI_ISL_953408, EPI_ISL_953420, EPI_ISL_953425	Laboratorio de Investigaciones de Baney	"Swiss Tropical and Public Health Institute"	"Carlos Cortes; Bonifacio Manguire Nlavo; Claudia Daubenberger; Diosdado Odjama Nseng Ada; Elizabeth Nyakarungu; Guillermo Garcia; Maximilian Mpina; Mitoha Ondo O Ayekaba; Philip Wonder Phiri"; Salome Hosch; Tobias Schindler
EPI_ISL_1700675, EPI_ISL_1700679, EPI_ISL_2002671	Laboratorio de Investigaciones de Baney	Swiss Tropical and Public Health Institute	Bonifacio Manguire Nlavo; Carlos Cortes; Claudia Daubenberger; Diosdado Odjama Nseng Ada; Elizabeth Nyakarungu; Guillermo Garcia; Maximilian Mpina; Mitoha Ondo O Ayekaba; Philip Wonder Phiri; Philipp Wagner; Salome Hosch; Tobias Schindler; Yahya Maidane
EPI_ISL_648311, EPI_ISL_648326, EPI_ISL_648336, EPI_ISL_648339, EPI_ISL_648367, EPI_ISL_648374, EPI_ISL_648378			
see above	Laboratorio de Investigaciones de Baney	University Hospital Basel, Clinical Bacteriology	Adrian Egli; Alfredo Mari; Bonifacio Manguire Nlavo; Carlos Cortes; Claudia Daubenberger; Diosdado Odjama Nseng Ada; Elizabeth Nyakarungu; Guillermo Garcia; Helena Seth-Smith; Madlen Stange; Maximilian Mpina; Mitoha Ondo O Ayekaba; Philip Wonder Phiri; Salome Hosch; Tim Roloff; Tobias Schindler
EPI_ISL_1092325, EPI_ISL_1137488, EPI_ISL_1532225	Laboratorio de Referencia Nacional de Virus Respiratorio. Instituto Nacional de Salud Perú	Laboratorio de Referencia Nacional de Biotecnología y Biología Molecular. Instituto Nacional de Salud Perú	Carlos Padilla Rojas; Henri Bailon Calderon; Johanna Balbuena Torrez; Karolyn Vega Chozo; Luis Barcena; Marco Galarza Perez; Maribel Huaringa Nuñez; Nancy Rojas Serrano; Omar Caceres Rey; Priscila Lope Pari
EPI_ISL_1111112, EPI_ISL_1111114, EPI_ISL_1111223, EPI_ISL_1111249, EPI_ISL_1111266	Laboratorio de Referencia Nacional de Virus Respiratorio. Instituto Nacional de Salud Perú	Laboratorio de Referencia Nacional de Enteropatógenos. Instituto Nacional de Salud del Perú	Fiorella Orellana Peralta; Iris Silva Molina; Junior Caro Castro; Ronnie Gavilan Chavez; Veronica Hurtado Vela; Willi Quino Sifuentes
EPI_ISL_540959	Laboratorio de Referencia Nacional de Virus Respiratorios, Instituto Nacional de Salud Peru	Laboratorio de Genómica Microbiana, Universidad Peruana Cayetano Heredia	Alejandra Dávila-Barclay; Brenda Ayzanoa; Camila Castillo-Vilcahuaman; Guillermo Salvatierra; Janet Huancachoque; Luis González; Maribel Huaringa; Pablo Tsukayama; Pedro E. Romero; Pool Marcos
EPI_ISL_3401456	Laboratorio de Referencia Nacional de Virus Respiratorios. Centro Nacional de Salud Publica. Instituto Nacional de Salud Peru.	Laboratorio de Referencia Nacional de Virus Respiratorios. Centro Nacional de Salud Publica. Instituto Nacional de Salud Peru.	Carlos Padilla Rojas; Henri Bailon Calderon; Iris Silva Molina; Joseph Huayra Niquen; Lely Solari Zerpa; Luis Barcena Flores; Marco Galarza Perez; Nancy Rojas Serrano; Nieves Sevilla Castañeda; Omar Caceres Rey; Orson Mestanza Millones; Princesa Medrano Alhuay; Priscila Lope Pari; Sandra Morales Ruiz; Sara Gordillo Vilchez; Steve Acedo Lazo; Veronica Hurtado Vela; Victor Jimenez Vasquez; Wendy Lizarraga Olivares
EPI_ISL_2536804, EPI_ISL_2921475, EPI_ISL_3376410	Laboratorio de Referencial Nacional de Virus Respiratorios	Laboratorio de Referencial Nacional de Virus Respiratorios	Carlos Padilla Rojas; Henri Bailon Calderon; Iris Silva Molina; Joseph Huayra Niquen; Lely Solari Zerpa; Luis Barcena Flores; Marco Galarza Perez; Nancy Rojas Serrano; Omar Caceres Rey; Orson Mestanza Millones; Priscila Lope Pari; Sandra Morales Ruiz; Steve Acedo Lazo; Veronica Hurtado Vela
EPI_ISL_3275292, EPI_ISL_3275295	Laboratorio de Vigilancia en Salud Pública el Salvador	Genomics and Proteomics Departament, Gorgas Memorial Institute For Health Studies	Alexander Martinez; Ambar Moreno; Claudia Díaz; Claudia Gonzalez; Denis G Jovel A; Gustavo M Ramirez; Jessica Gondola; Leyda Abrego; Marlene Castillo; Oris Chavarria; Ruth C Vasquez C; Sandra Paola Paz
EPI_ISL_1396198	Laboratorio de Virología del Hospital de Niños Dr. Ricardo Gutierrez	Área de Secuenciación del Laboratorio de Virología del Hospital de Niños Dr. Ricardo Gutierrez on behalf of 'Proyecto Argentino Interinstitucional de genomica de SARS-CoV-2' (PAIS Consortium)	A; Acevedo; Acuña; Alexay; Alvarez Lopez; Barreda Frank; C; D; E; G; Goya; Grandis; Jacques; LE; Labarta; Lusso; M; ME; MI; Medina; Mistchenko; N; Nabaes Jodar; Natale; O; S; Streitenberger; Thomas; Valinotto; Viegas, M.; Villegas
EPI_ISL_1662550	Laboratorio di Microbiologia	Laboratorio di Microbiologia	Martinetti Lucchini Gladys; Valeria Spina
EPI_ISL_754910	Laboratory Diagnostics and Clinical Immunology of Developmental Age, Medical University of Warsaw	genXone SA, Research & Development Laboratory; The Faculty of Mathematics, Informatics and Mechanics of the University of Warsaw	Anna Gambin; Grzegorz Nowicki; Jakub Grabowski; Maciej Sykulski; Michał Kaszuba; Monika Mańkowska-Woźniak; Natalia Drwęska-Matelska; Urszula Demkow; Łukasz Krych
EPI_ISL_956331	Laboratory Medicine	Department of Laboratory Medicine, Lin-Kou Chang Gung Memorial Hospital, Taoyuan, Taiwan	Cheng-Hsun Chiu; Cheng-Ta Yang; Chung-Guei Huang; Guang-Wu Chen; Kuo-Chien Tsao; Kuo-Ming Lee; Mei-Jen Hsiao; Peng-Nien Huang; Po-Wei Huang; Shin-Ru Shih; Shu-Li Yang; Yi-Chun Liu; Yu-Nong Gong
EPI_ISL_2467938	Laboratory for HIV and opportunistic infections diagnosis The Republican Research and Practical Center for Epidemiology and Microbiology (RRPCEM)	Laboratory for HIV and opportunistic infections diagnosis The Republican Research and Practical Center for Epidemiology and Microbiology (RRPCEM	Alina Drozd; Artur Akhremchuk; Elena Gasich; Hanna Gudel; Katsiaryna Belyakova; Kirill Bulda; Leonid Valentovich; Nastassia Kabankova
EPI_ISL_1138899	Laboratory for HIV and opportunistic infections diagnosis The Republican Research and Practical Center for Epidemiology and Microbiology (RRPCEM)	Laboratory for HIV and opportunistic infections diagnosis The Republican Research and Practical Center for Epidemiology and Microbiology (RRPCEM)	Anatoly Krasko; Artur Akhremchuk; Elena Gasich; Kirill Bulda; Leonid Valentovich; Vladimir Gorbunov
EPI_ISL_2233677, EPI_ISL_2233685	Laboratory of Clinical Virology	Greek Genome Center, Biomedical Research Foundation of the Academy of Athens (BRFAA)	Dimitrios Thanos; Emmanouil Athanasiadis; George Sourvinos; Giannis Vatsellas; Katerina Zoi; Theodoros Loupis
EPI_ISL_1138532	Laboratory of Communicable Diseases	1. Laboratory of Communicable Diseases (Estonia); 2. Eurofins Genomics Europe Sequencing GmbH	Liidia Dotsenko
EPI_ISL_735392	Laboratory of Genetics and Personalized Medicine, Zan Mitrev Clinic	Laboratory of Genetics and Personalized Medicine, Zan Mitrev Clinic	2020; Kungulovski et al.
EPI_ISL_2301696, EPI_ISL_2301733	Laboratory of Immunohematology, Division of Hematology	Greek Genome Center, Biomedical Research Foundation of the Academy of Athens (BRFAA)	Athanasia Mouzaki; Dimitrios Thanos; Emmanouil Athanasiadis; Giannis Vatsellas; Katerina Zoi; Theodoros Loupis
EPI_ISL_434466	Laboratory of Microbiology, Medical School, National and Kapodistrian University of Athens	Laboratory of Biology, Department of Medicine, Democritus University of Thrace	Bampali, M.; Dovrolis, N.; Froukala, E.; Gatzidou, E.; Kassela K.; N. and Karakasiliotis, I.; Spanakis; Stavropoulou, A.; Tsakris, A.; Veletza, S.
EPI_ISL_2896981	Laboratory of Microbiology, National Reference Lab, Charles Nicolle Hospital; 2-University of Tunis ElManar, Faculty of Medicine of Tunis, LR99ES09, Tunis, Tunisia	1-Clinical and Experimental Pharmacology Lab, LR16SP02, National Center of Pharmacovigilance, University of Tunis El Manar, Tunis, Tunisia. 2-Neurodegenerative diseases and psychiatric troubles, LR18SP03, Razi Hospital, University of Tunis El Manar, Tunis, Tunisia. 3- Ministry of Health, National Observatory of New and Emerging Diseases, 1006, Tunis, Tunisia	Alia Benkahla; Emna Gaies; Ilhem Boutiba-Ben Boubaker.; Imen Kacem; Imen Mkada; Ines Mdini; Jalila Ben Khelil; Maher Kharrat; Mouna Ben Sassi; Mouna Safer; Nissaf Ben Alaya; Riadh Daghfous; Riadh Gouider; Roua Ben Othman; Salma Abid; Sameh Trabelsi; Sana Ferjani; Sarra Chammam
EPI_ISL_803120, EPI_ISL_2153433, EPI_ISL_2154331	Laboratory of Microbiology, National Reference Lab, Charles Nicolle Hospital; 2-University of Tunis ElManar, Faculty of Medicine of Tunis, LR99ES09, Tunis, Tunisia	Clinical and Experimental Pharmacology Lab, LR16SP02, National Center of Pharmacovigilance, University of Tunis El Manar, Tunis, Tunisia. 2-Neurodegenerative diseases and psychiatric troubles, LR18SP03, Razi Hospital, University of Tunis El Manar, Tunis, Tunisia. 3- Ministry of Health, National Observatory of New and Emerging Diseases, 1006, Tunis, Tunisia	Ahmed Fakhfakh; Alia BenKahla; Gaies Emna; Habiba Ben Romdhane; Hanen El Jebari; Ilhem Boutiba-Ben Boubaker; Ilhem Boutiba-Ben Boubaker.; Imen Kacem; Imen Mdini; Jalila Ben Khelil; Maher Kharrat; Manel Ben Sassi; Mouna Ben Sassi; Mouna Safer; Nissaf Ben Alaya; Raja Mahfoudh; Riadh Daghfous; Riadh Gouider; Riadh Gouider.; Roua Ben Othman; Salma Abid; Sameh Trabelsi; Sana Ferjani; Sarra Chamman; Souissi Amira; Taha Maatoug; Zaineb Hamzaoui

EPI_ISL_3343854, EPI_ISL_3343967, EPI_ISL_3344066	Laboratory of Molecular Biology and Cancer Immunology	Quadram Institute Bioscience	Abdul K. Sessay; Alexander J. Trotter; Andrew J. Page; Bassam Badran; Georgi Merhi; Hala Abou Naja; Hamad Hasan; Jad Koweyes; Janine M. Wilkinson; Kate A. Makin; Leonardo de Oliveira Martins; Mark Pallen; Mark Webber; Martin Lott; Matthew W. Felgate; Mona Al Buaini; Nabil-Fareed Alikhan; Nada Ghosn; Orla J. Jupp; Rachael Stanley; Rose K. Davidson; Sarah Gardener; Sima Tokajian; Sophie J. Prosolek; Tatiana Tohmeh; Thanh Le-Viet
EPI_ISL_876028	Laboratory of Molecular Biology, Diagnostyka sp. z o.o.	genXone SA, Research & Development Laboratory	Grzegorz Nowicki; Jakub Grabowski; Maciej Sykulski; Michał Kaszuba; Monika Mańkowska-Woźniak; Natalia Drwęska-Matelska; Łukasz Krych
EPI_ISL_801607	Laboratory of Molecular Virology, Pontificia Universidad Católica de Chile	MSHS Pathogen Surveillance Program	Adolfo Garcia-Sastre; Adriana van De Guchte; Ajay Obla; Ana Maria Contreras; Ana S. Gonzalez-Reiche; Bremy Alburquerque; Carlos Palma; Constanza Maldonado; Edward C. Holmes; Eileen Serrano; Erick Salinas; Hala Alshammary; Harm van Bakel; Jayeeta Dutta; Jorge Levican; Juan Soto; Leonardo I. Almonacid; M. Belen Leyton; Marcela Ferres; Matthew M. Hernandez; Melissa Smith; Rafael A. Medina.; Robert Sebra; Shwetha Hara Sridhar; Tamara García-Salum; Viviana Simon; Ying-Chih Wang; Zenab Khan
EPI_ISL_984651	Laboratory of Virology of Federal Budget Health Care Institution Center of Hygiene and Epidemiology in Arkhangelsk region	Group of Genomics and Postgenomic Technologies of Central Research Institute of Epidemiology	Akimkin VG; Bulanenko VP; Kaptelova VV; Kondrasheva LV; Korneenko EV; Ponomareva YA; Saenko SS; Samoilov AE; Semyina LV; Shishko LA; Speranskaya AS; Tivanova EV; Valdokhina AV
EPI_ISL_1341503	Laboratory of Virology, National center of expertise	RSE "National Center of Expertise" and RSE "National center for Biotechnology"	Abdaliyev Askar; Amirgazin Asylulan; Balykbaev Kanat; Kamalova Dinara; Ramankulov Erlan; Sharipova Saule; Shevtsov Alexandr; Tungushbayev Talgat
EPI_ISL_1191740	Laboratory of virology and molecular diagnostics	Laboratory of virology and molecular diagnostics	Boshevska Golubinka; Janchevska Elizabeta; Kuzmanovska Maja
EPI_ISL_1669992, EPI_ISL_2392161, EPI_ISL_2987557, EPI_ISL_2987571	Laboratory of virology and molecular diagnostics, Institute of Public Health	Laboratory of virology and molecular diagnostics, Institute of Public Health	Boshevska G; Janchevska E.; Kuzmanovska M
EPI_ISL_1448018	Laboratory of virology, National center of expertise	RSE "National Center for Biotechnology" and RSE "National Center of Expertise"	Abdaliyev Askar; Amirgazin Asylulan; Balykbaev Kanat; Kamalova Dinara; Ramankulov Yerlan; Sharipova Saule; Shevtsov Alexandr; Tungushbayev Talgat
EPI_ISL_2150989, EPI_ISL_2158599	Laboratório de Biologia Molecular Jean Piaget	MRCG at LSHTM, Genomics lab	Abdoulie Kanteh; Abdul Karim Sesay; Adul Candé; Aicha Balde; Aladje Balde; Bakary Sanyang; Bubacar Delgado Pinto Embaló; Dabiri Damilari; Edmira Maria da Costa; Erica Luis Maria Magalhães; Faatu Cassama; Mariama Kujabi; Milanca Agostinho Cá; Paulina Joãozinho da Costa Jarra Manneh; Rei José Pereira; Rui Inndi; Sainabou Laye Ndure; Simão Tchuda Bióté
EPI_ISL_1152514	Labormedizin Darmstadt	Robert Koch Institute	
EPI_ISL_1973556, EPI_ISL_2484343, EPI_ISL_2484440, EPI_ISL_2484541, EPI_ISL_2484554, EPI_ISL_2484555, EPI_ISL_2484579, EPI_ISL_2484580, EPI_ISL_2484581, EPI_ISL_2484583, EPI_ISL_2484607, EPI_ISL_2768045, EPI_ISL_3344073			
see above	Labormedizinisches Zentrum Dr Risch	Clinical Bacteriology	Adrian Egli; Alfredo Mari; Fanny Wegner; Hans Hirsch; Helena MB Seth-Smith; Julia Bielicki; Karoline Leuzinger; Lorenz Risch; Madlen Stange; Manuel Battegay; Martin Risch; Nadia Wohlwend; Tim Roloff
EPI_ISL_660136	Lancet	National Health Laboratory Service (NHLS), Tygerberg	Bronwyn Kleinhans; Davis M-A; Draper C; Eduan Wilkindon; Gert van Zyl; Houriiyah Tegally; Hsiao M; Kayla Delaney; Siegfried N; Susan Engelbrecht; Tulio de Oliveira; Williamson C; Wolfgang Preiser
EPI_ISL_3074056	Lancet Laboratory	National Institute for Communicable Diseases of the National Health Laboratory Service	Amoako DG; Bhiman JN; Everatt J; Ismail A; Mahlangu B; Mnguni A; Mohale T; Ntuli N; Scheepers C
EPI_ISL_738321, EPI_ISL_738323	Landstuhl Regional Medical Center	United States Air Force School of Aerospace Medicine	Amanda Javorina; Anthony Fries; Clarise Starr; Cole Anderson; Elizabeth Macias; Fritz Castillo; Jennifer Meyer; Sarah Purves; William Gruner
EPI_ISL_2779489, EPI_ISL_2779490	Lanet hospital	USAMRD-A, Basic Science Laboratory	Alan Lemtudo; Beth Mutai; Brian Andika; Carol Kifude; Clement Masakwe; Eric Muthanje; Esther Omuseni; Faith Sigei; Gathii Kimita; George Awinda; John Waitumbi; Josphat Nyataya; Rachel Githii; Rehema Liyai; Stephen Ochola
EPI_ISL_2443545, EPI_ISL_2863898	Lboratorio Central de Saude Publica do Estado do Parana (LACEN/PR)	Laboratory of Respiratory Viruses and Measles, Oswaldo Cruz Institute, FIOCRUZ	Agatha Cristinne Prudencio Soares; Alice Sampaio Rocha; Ana Carolina Mendonca; Anna Carolina Paixao; Elisa Cavalcante Pereira; Fernando Motta; Ighor Leonardo Arantes Gomes; Irina Riediger; Luciana Appolinario; Marilda Siqueira on behalf of the Fiocruz COVID-19 Genomic Surveillance Network; Paola Resende; Renata Serrano Lopes; Taina Venas
EPI_ISL_2138822, EPI_ISL_2138848	Life Sciences Center, Vilnius University	Institute of Biotechnology, Life Sciences Center, Vilnius University	Albertas Timinskas; Alma Gedvilaite; Danguole Ziogiene; Emilija Vasiliunaite; Milda Norkiene
EPI_ISL_1018852, EPI_ISL_1244963, EPI_ISL_1246214, EPI_ISL_1275591, EPI_ISL_1333887, EPI_ISL_1374137, EPI_ISL_1410265, EPI_ISL_1451288, EPI_ISL_1451346, EPI_ISL_1473594, EPI_ISL_1473658, EPI_ISL_1483822, EPI_ISL_1831400, EPI_ISL_1831515			
see above	Lighthouse Lab in Cambridge	Wellcome Sanger Institute for the COVID-19 Genomics UK (COG-UK) Consortium	Cordelia Langford; David K. Jackson; Dominic Kwiatkowski; Ewan Harrison; Ian Johnston; Jeffrey Barrett; John Sillitoe on behalf of the Wellcome Sanger Institute COVID-19 Surveillance Team; Rob Howes; Roberto Amato; Sonia Goncalves; The Lighthouse Lab in Cambridge and Alex Alderton
EPI_ISL_1173975, EPI_ISL_1333084, EPI_ISL_1673188, EPI_ISL_1698571, EPI_ISL_1698756	Lighthouse Lab in Glasgow	Wellcome Sanger Institute for the COVID-19 Genomics UK (COG-UK) Consortium	Anna Dominiczak and Alex Alderton; Carol Clugston; Cordelia Langford; David Gray; David K. Jackson; Dominic Kwiatkowski; Ewan Harrison; Harper VanSteenhouse; Ian Johnston; Jeffrey Barrett; John Sillitoe on behalf of the Wellcome Sanger Institute COVID-19 Surveillance Team; Roberto Amato; Sonia Goncalves; Yumi Kasai
EPI_ISL_530530, EPI_ISL_533385	Lighthouse Lab in Glasgow	Wellcome Sanger Institute for the COVID-19 Genomics UK (COG-UK) consortium	Anna Dominiczak and Alex Alderton; Carol Clugston; Cordelia Langford; David Gray; David K. Jackson; Dominic Kwiatkowski; Ewan Harrison; Harper VanSteenhouse; Ian Johnston; John Sillitoe; Roberto Amato; Sonia Goncalves; Yumi Kasai
EPI_ISL_1790998, EPI_ISL_2317665, EPI_ISL_2435364	Lighthouse Lab in Milton Keynes	Wellcome Sanger Institute for the COVID-19 Genomics UK (COG-UK) Consortium	Cordelia Langford; David K. Jackson; Dominic Kwiatkowski; Ewan Harrison; Ian Johnston; Jeffrey Barrett; John Sillitoe on behalf of the Wellcome Sanger Institute COVID-19 Surveillance Team; Roberto Amato; Sonia Goncalves; The Lighthouse Lab in Milton Keynes and Alex Alderton
EPI_ISL_2129363	Limbach - MVZ Humangenetik Ulm	Robert Koch Institute	
EPI_ISL_568978, EPI_ISL_804502, EPI_ISL_900119, EPI_ISL_900181, EPI_ISL_1201045, EPI_ISL_1201046	MEPHI, Aix Marseille University	MEPHI, Aix Marseille University	Anthony LEVASSEUR
EPI_ISL_2895751	MIDDELBURG LABORATORY	National Institute for Communicable Diseases of the National Health Laboratory Service	Amoako DG; Bhiman JN; Everatt J; Ismail A; Mahlangu B; Mnguni A; Mohale T; Ntuli N; Scheepers C
EPI_ISL_2556117, EPI_ISL_2557251	MOH - Jaber Al-Ahmad Hospital (Innovation Research Laboratory)	MOH - Jaber Al-Ahmad Hospital (Innovation Research Laboratory)	Mohammad Alghounaim; Salman Al-Sabah
EPI_ISL_2779443	MOH-K	USAMRD-A, Basic Science Laboratory	Alan Lemtudo; Beth Mutai; Brian Andika; Carol Kifude; Clement Masakwe; Eric Muthanje; Esther Omuseni; Faith Sigei; Gathii Kimita; George Awinda; John Waitumbi; Josphat Nyataya; Rachel Githii; Rehema Liyai; Stephen Ochola
EPI_ISL_2346387, EPI_ISL_2346419, EPI_ISL_2346425, EPI_ISL_2346426, EPI_ISL_2346428, EPI_ISL_2346429, EPI_ISL_2346432, EPI_ISL_2346433, EPI_ISL_2346434, EPI_ISL_2346435			
see above	MRC/UVRI & LSHTM Uganda Research Unit, Central Public Health Laboratories	MRC/UVRI & LSHTM Uganda Research Unit, Central Public Health Laboratories	Dan Lule Bugembe; Isaac Sseeewanyana; Matthew Cotten; My V.T. Phan; Patrick Semanda; Pontiano Kaleebu; Susan Nabadda
EPI_ISL_1970565, EPI_ISL_1970566, EPI_ISL_1970567, EPI_ISL_1970568	MRC/UVRI & LSHTM Uganda Research Unit	MRC/UVRI & LSHTM Uganda Research Unit	Dan Lule Bugembe; Isaac Sseeewanyana; Matthew Cotten; My V.T. Phan; Patrick Semanda; Pontiano Kaleebu; Susan Nabadda
EPI_ISL_1469325, EPI_ISL_1469397, EPI_ISL_1469416	MRC/UVRI & LSHTM Uganda Research Unit	Where sequence data have been generated and submitted to GISAID	Dan Lule Bugembe; Isaac Sseeewanyana; Matthew Cotten; My V.T. Phan; Patrick Semanda; Pontiano Kaleebu; Susan Nabadda
EPI_ISL_3149400, EPI_ISL_3149401, EPI_ISL_3149402, EPI_ISL_3149403	MRC/UVRI & LSHTM Uganda Research Unit , Rakai Health Sciences Program	MRC/UVRI & LSHTM Uganda Research Unit , Rakai Health Sciences Program	; Charles Ssuuna; Matthew Cotten Dan Lule Bugembe; My V.T. Phan; Pontiano Kaleebu; Ronald Moses Galiwango; Steven J Reynolds
EPI_ISL_2690447, EPI_ISL_2690448, EPI_ISL_2690449, EPI_ISL_2690450, EPI_ISL_2690451, EPI_ISL_2690456, EPI_ISL_2690458, EPI_ISL_2690459, EPI_ISL_2690461, EPI_ISL_2690462			
see above	MRC/UVRI & LSHTM Uganda Research Unit, Central Public Health Laboratories, Rakai Health Sciences Program	MRC/UVRI & LSHTM Uganda Research Unit, Central Public Health Laboratories	Dan Lule Bugembe; Hellen Nansumba; Isaac Sseeewanyana; Matthew Cotten; My V.T. Phan; Patrick Semanda; Pontiano Kaleebu; Susan Nabadda
EPI_ISL_2690465, EPI_ISL_2690466, EPI_ISL_2690467, EPI_ISL_2690469, EPI_ISL_2690470, EPI_ISL_2690471, EPI_ISL_2690472, EPI_ISL_2690473, EPI_ISL_2690474, EPI_ISL_2690475, EPI_ISL_2690476			
see above	MRC/UVRI & LSHTM Uganda Research Unit, Central Public Health Laboratories, Rakai Health Sciences Program	MRC/UVRI & LSHTM Uganda Research Unit, Rakai Health Sciences Program	Charles Ssuuna; Dan Lule Bugembe; Matthew Cotten; My V.T. Phan; Pontiano Kaleebu; Ronald Moses Galiwango; Steven J Reynolds
EPI_ISL_3149350	MRC/UVRI & LSHTM Uganda Research Unit, Central Public Health Laboratories	MRC/UVRI & LSHTM Uganda Research Unit, Central Public Health Laboratories	Dan Lule Bugembe; Hellen Nansumba; Isaac Sseeewanyana; Matthew Cotten; My V.T. Phan; Patrick Semanda; Pontiano Kaleebu; Susan Nabadda
EPI_ISL_3152083,	MRCG	LBV Le Dantec	; Abdoulie Kante; Abdul Karim Cesay; Adjiratou Aissatou BA; Aminata Sileymane Thiam; Anna julienne Selbe NDiaye; Assane Dieng; Awa Ba-Diallo; Dianke Samaté; Gora Lo; Halimatou Diop Ndiaye; Jarra Manneh; Khadim Gueye; Makhtar Camara; Mbengué Fall; Moustapha Sakho;

EPI_ISL_3152106, EPI_ISL_3152108			Oumy DIOP; Pasacaline Manga; Pauline Yacine Sene; Sada Diallo; Serigne Saliou Niane; ousseynou Gueye
EPI_ISL_428857, EPI_ISL_471158, EPI_ISL_471160, EPI_ISL_471168, EPI_ISL_561009, EPI_ISL_561024, EPI_ISL_561147, EPI_ISL_561213, EPI_ISL_810982, EPI_ISL_810986, EPI_ISL_811037, EPI_ISL_1216112, EPI_ISL_1234531, EPI_ISL_2142748, EPI_ISL_2820697, EPI_ISL_2941556, EPI_ISL_2941560, EPI_ISL_2958626, EPI_ISL_2958629, EPI_ISL_2958631, EPI_ISL_2958632, EPI_ISL_2958636, EPI_ISL_2958637, EPI_ISL_2958638, EPI_ISL_2958644, EPI_ISL_2958645, EPI_ISL_2958646, EPI_ISL_2958647, EPI_ISL_2958648, EPI_ISL_2958649, EPI_ISL_2958653, EPI_ISL_3150934			
see above	MRCG at LSHTM Genomics lab	MRCG at LSHTM Genomics lab	Abdoulie Kanteh; Abdul Karim sesay; Bakary Sanyang; Dabiri Damilari; Damiri Damilari; Jarra Manneh; Mariama Kujabi; Sainabou laye Ndure; Sesay et al
EPI_ISL_414476	MSHS Clinical Microbiology Laboratories	MSHS Pathogen Surveillance Program	Adolfo Garcia-Sarstre; Alberto Paniz-mondolfi; Ana Sylvia Gonzalez-Reiche; Emilia Sordillo; Florian Krammer; Gopi Patel; Harm van Bakel; Jose Polanco; Judith Aberg; Lisa Miorin; Matthew Hernandez; Melissa Gitman; Melissa Smith; Nancy Francoeur; Randy Albrecht; Robert Sebra; Shelcie Fabre; Viviana Simon; Wen-chun Liu; Zenab Khan
EPI_ISL_1936102, EPI_ISL_1936104, EPI_ISL_1936107, EPI_ISL_1936108, EPI_ISL_1936109, EPI_ISL_1936111, EPI_ISL_1936112, EPI_ISL_1936113, EPI_ISL_1936115, EPI_ISL_1936116, EPI_ISL_1936117, EPI_ISL_1936124, EPI_ISL_1936127, EPI_ISL_1936134, EPI_ISL_1936137, EPI_ISL_1936139, EPI_ISL_1936140, EPI_ISL_1936145, EPI_ISL_1936240, EPI_ISL_1936241, EPI_ISL_1936242, EPI_ISL_1936243, EPI_ISL_1936245, EPI_ISL_1936246, EPI_ISL_1936248, EPI_ISL_1936249, EPI_ISL_1936250, EPI_ISL_1936257, EPI_ISL_1936261, EPI_ISL_1936262, EPI_ISL_1936264, EPI_ISL_1936265, EPI_ISL_1936271, EPI_ISL_1936272, EPI_ISL_1936298, EPI_ISL_1969080, EPI_ISL_1969081			
see above	Main Chemical Laboratories Egypt Army	Main Chemical Laboratories Egypt Army	Abdullah Salama; AbedElrahman Zekri; Ahmed Gad; Bassem Elharty; Laila Elsawy; Mervat Hassan; Mohamed Seadawy; Mohamed Shamel; Mostfa Elhoseiny; Mostfa Yakout; Ola Elroby; Sabah Ahmed; Sherine Helmy
EPI_ISL_2779292	Malaba border point	USAMRD-A, Basic Science Laboratory	Alan Lemtudo; Beth Mutai; Brian Andika; Carol Kifude; Clement Masakwe; Eric Muthanje; Esther Omuseni; Faith Sigei; Gathii Kimita; George Awinda; John Waitumbi; Josphat Nyataya; Rachel Githii; Rehema Liyai; Stephen Ochola
EPI_ISL_1827704	Manzana	National Institute for Communicable Diseases of the National Health Laboratory Service	Amoako DG; Bhiman JN; Ismail A; Mahlangu B; Maphalala GP; Mohale T; Ntuli N; Scheepers C
EPI_ISL_1363775	Maryland Genomics, Institute for Genome Sciences, University of Maryland School of Medicine	Maryland Genomics, Institute for Genome Sciences, University of Maryland School of Medicine	Aditya; Claire M; Fraser; Holly; Humphrys; Jacques; Kranthi; Lisa D; Luke J; Mehta; Mike; Ott; Ravel; Roussey; Sadzewicz; Sandra; Tallon; Vavikolanu
EPI_ISL_1910608, EPI_ISL_1913025	Mashrek Medical Diagnostic Center	Microbial Pathogenomics Lab - LAU	Georgi Merhi; Jad Koweyes; Sima Tokajian; Tamara Salloum
EPI_ISL_1196007	Mbabane Gov Hospital	National Institute for Communicable Diseases of the National Health Laboratory Service	Amoako DG; Bhiman JN; Ismail A; Mahlangu B; Maphalala GP; Mohale T; Ntuli N; Scheepers C
EPI_ISL_482761, EPI_ISL_483035, EPI_ISL_483037, EPI_ISL_1109484, EPI_ISL_1165080	Medical Ain Shams Research Institute (MASRI), Ain Shams University	Medical Ain Shams Research Institute (MASRI), Ain Shams University	Ahmad Moustafa; Ashraf Omar; Aya Mohamed; Fatma Ebied; Ghada Ismael; Hagar Elshora; Hala Hafez; Hesham Elghazaly; Hoda Ezz Elarab; Iman Foda; Mahmoud Elmeitini; Manal Hamdy Elsaid; Mohamed Elhadidi; Osama Mansour; Osama Mansour.; Reham Kassab; Reham Mamdouh; Samia Abdou Girgis; Sara Elnakeep; Sara Hassan Agwa; Shaimaa Moustafa; Shimaa Moustafa
EPI_ISL_2107509, EPI_ISL_2107524	Medical Laboratory Sciences, Arab American University	Medical Laboratory Sciences, Arab American University	Al-Jawabreh, A.; Al-Jawabreh, H.; Dumaidi, K.; Ereqat, S.; Nasereddin, A.
EPI_ISL_1156171	Medizinisches Labor Wahl Lüdenscheid	Robert Koch Institute	
EPI_ISL_480338	Microbial Genomics Laboratory, Institut Pasteur de Montevideo	Microbial Genomics Laboratory, Institut Pasteur de Montevideo	Cecilia Salazar; Gonzalo Moratorio; Gregorio Iraola; Ignacio Ferrés; Marianoel Pereira; Pilar Moreno
EPI_ISL_562386	Microbiological Diagnostic Unit - Public Health Laboratory (MDU-PHL)	MDU-PHL	Sait, M.; Schultz M. B.; Seemann, T.; Sherry, N.
EPI_ISL_2136858, EPI_ISL_2281323	Microbiology Department, Laboratori Clínic Metropolitana Nord. Hospital Universitari Germans Trias i Pujol	Can Ruti SARS-CoV-2 Sequencing Hub (HUGTiP/IrsiCaixa/IGTP)	Alba Sánchez; Anna Not; Antoni E Bordoy; Bonaventura Clotet; Cristina Casañ; Cristina Esteban; Francesc Catala-Moll; Gemma Clara; Ignacio Blanco; Marc Noguera-Julian; Maria Casadellà; Mariona Parera; Mercedes Guerrero; Montserrat Giménez; Pere-Joan Cardona; Pilar Armengol; Roger Paredes; Verónica Saludes; and Elisa Martró on behalf of the Can Ruti SARS-CoV-2 Sequencing Hub.
EPI_ISL_1347071, EPI_ISL_1386061, EPI_ISL_1583545, EPI_ISL_1636446	Microbiology Department, Laboratori Clínic Metropolitana Nord. Hospital Universitari Germans Trias i Pujol.	Can Ruti SARS-CoV-2 Sequencing Hub (HUGTiP/IrsiCaixa/IGTP)	Alba Sánchez; Anna Not; Antoni E Bordoy; Bonaventura Clotet; Cristina Casañ; Cristina Esteban; Francesc Catala-Moll; Gemma Clara; Ignacio Blanco; Marc Noguera-Julian; Maria Casadellà; Mariona Parera; Mercedes Guerrero; Montserrat Giménez; Pere-Joan Cardona; Pilar Armengol; Roger Paredes; Verónica Saludes; and Elisa Martró on behalf of the Can Ruti SARS-CoV-2 Sequencing Hub.
EPI_ISL_1495974	Microbiology Department, University Hospital Donostia	Microbiology Department, University Hospital Donostia	Cilla G.; Gomez M; Marimon JM; Montes M; Piñeiro L; Sorarrain A
EPI_ISL_2688489	Microbiology Laboratory, Attikon University Hospital, Athens	Central National Laboratory ,Public Health Organization	N.Siafakas; S.Pournaras et al
EPI_ISL_1064028	Microbiology and Virology Unit, Azienda Ospedale Padova, Padova, Italy	Department of Molecular Medicine, Computational Medicine Group, Univeresity of Padova, Padova, Italy	Andrea Crisanti; Andrea Spitaleri; Claudia Del Vecchio; Daniela Maria Cirillo; Dejan Lazarevic; Elisa Franchin; Enrico Lavezzo; Fabio Simeoni; Federico Bianca; Francesca Saluzzo; Francesco Onelia; Giovanni Lorenzin; Giovanni Tonon; Laura Manuto; Marco Grazioli; Stefano Toppo
EPI_ISL_2348487	Middle East Institute of Health University Hospital	Microbial Pathogenomics Lab - LAU	Edmond Abboud; Georgi Merhi; Jad Koweyes; Sima Tokajian
EPI_ISL_548103, EPI_ISL_548121, EPI_ISL_2406492	Middlemore Hospital	Institute of Environmental Science and Research (ESR)	Anja Werno; Antje van der Linden; Arlo Upton; Chris Mansell; David Hammer; Dragana Drinkovic; Erasmus Smit; Gary McAuliffe; Hana Sofia Andersson; Hermes Perez; James Ussher; Jill Sherwood; Jing Wang; Joep de Ligt; Josh Freeman; Julia Howard; Juliet Elvy; Lauren Jelly; Mary DeAlmeida; Matt Blakiston; Matt Storey; Matthew Rogers; Max Bloomfield; Michael Addidle; Michelle Balm; Muhammad Faisal; Nikki Freed; Olin Silander; Olivia Stroeven; Rachel Boyle; Sally Roberts; SallyAnn Harbison; Sarah Jefferies; Sharmini Muttaiyah; Susan Morpeth; Susan Taylor; Timothy Blackmore; Vani Sathyendran; Veronica Playle; Virginia Hope; Xiaoyun Ren
EPI_ISL_906049, EPI_ISL_940711, EPI_ISL_1063988, EPI_ISL_1063989, EPI_ISL_1170076, EPI_ISL_2158074	Ministry of Health Turkey	Ministry of Health Turkey	Ayse Basak Altas; Ayşe Başak Altaş; Fatma Bayrakdar; Gulay Korukluoglu; Gülay Korukluoğlu; Suleyman Yalcin; Süleyman Yalcin; Yasemin Cosgun; Yasemin Coşgun
EPI_ISL_1713274, EPI_ISL_1713276, EPI_ISL_1713637, EPI_ISL_1713938, EPI_ISL_2274319, EPI_ISL_2408295, EPI_ISL_2843089, EPI_ISL_2843112			
see above	Ministry of Public Health / Hamad Medical Corporation	Biomedical Research Center (BRC), Qatar University / Qatar Genome Project (QGP)	Asmaa A. Al-Thani. MOPH and HMC: Abdullatif Al-Khal; Asmaa A. Al-Thani. MOPH and HMC: Abdullatif Al-Khal; BRC: Fatiha M. Benslimane; Chadi Saad; Dana Al-Batesh; Dina Elgakhlab QGP: Fatima H. Al-Kuwari; Dina Elgakhlab QGP: Fatima H. Al-Kuwari; Einas A. E. Al-Kuwari; Hadi M. Yassine; Hamad E. Al-Romaihi; Hamda Alromaihi; Heba A. Al-Khatib; Mashael A. Al-Bader; Mohammed Al-Thani; Muna A. S. Al-Maslamani; Oal Al-Jamal; Peter V. Coyle; Reham A. El-Kahlout. QBB: Tasneem Al-Hamad; Reham A. El-Kahlout. QBB: Tasneem Al-Hamad; Roberto Bertollini; Salih Al-Marri
EPI_ISL_1714023, EPI_ISL_1714185, EPI_ISL_1714436, EPI_ISL_1714461, EPI_ISL_1714618	Ministry of Public Health / Hamad Medical Corporation	Weill Cornell Medical College - Qatar (WCM-Q), Genomics Core Laboratory / Qatar Genome Project (QGP)	Chadi Saad MOPH and HMC: Abdullatif Al-Khal; Dina Elgakhlab; Einas A. E. Al-Kuwari; Hamad E. Al-Romaihi; Hamda Alromaihi; Joel A Malek. QGP: Fatima H. Al-Kuwari; Laith Abu-Raddad; Mashael A. Al-Bader; Meryem Bensaad; Mohammed Al-Thani; Muna A. S. Al-Maslamani; Peter V. Coyle; Reham A. El-Kahlout. QBB: Tasneem Al-Hamad; Roberto Bertollini; Salih Al-Marri; Shameem Younuskunju; WCMQ: Ayeda A. Ahmed; Yasmin Mohamoud
EPI_ISL_2140698, EPI_ISL_2375989	Mitchells Plain Hospital wc MPH	NHLS/UCT	Arash Iranzadeh; Bruna Galvao; Carolyn Williamson; Deelan Doolabh; Diana Hardie; Innocent Mudau; Kruger Marais; Lynn Tyers; Marvin Hsiao; Stephen Korsman
EPI_ISL_463740	Mohammed Bin Rashid University of Medicine and Health Sciences	Al Jalila Genomics Center	Abdulmajeed Alkhaja; Abiola Catherine Senok; Ahmad Abou Tayoun; Alawi Alsheikh-Ali; Divinlal Harilal; Hamda Khansaheb; Hanan Al Suwaidi; Mohammed Uddin; Norbert Nowotny; Qutayba Hamid; Rabih Halwani; Rifat Hamoudi; Rupa Murthy Varghese; Sathishkumar Ramaswamy; Tom Loney; Zulfa Omar Deesi
EPI_ISL_904015	Molecular Biology and Virology lab, Faculty of Veterinary Medicine, Jordan University of Science and Technology	Molecular Biology and Virology lab, Faculty of Veterinary Medicine, Jordan University of Science and Technology	Dr.Mahmoud Hamad Gazo; Dr.Moh''D Borhan Al-Zghoul; Dr.Mustafa Ababneh; Dr.Saied Jaradat; Ghaya Abdellatif Alwahdanee; Hazem Haddad; Mohammad Hussien Alboom; Suhaila Ibrahim khalil
EPI_ISL_421573	Molecular Diagnostic Services	KRISP, KZN Research Innovation and Sequencing Platform	Chimukangara B; Deforche K; Giandhari J; Lessells R; Ngcapu S; Pillay S; Samsunder N; Tegally H; Wilkinson E; de Oliveira T
EPI_ISL_2097215, EPI_ISL_2101358, EPI_ISL_2105875, EPI_ISL_2399457, EPI_ISL_2399546	Molecular Diagnostics Pathology Department Mater Dei Hospital Malta	Molecular Diagnostics Pathology Department Mater Dei Hospital Malta	C Cilia; G Zahra; L Grech; M Briffa; R Borg
EPI_ISL_802549, EPI_ISL_802550	Molecular Microbiology and Food Research Laboratory (MMFRLAB) - Universidad San Sebastián	Facultad de Ciencias de la Vida, UNAB	Claudio Meneses; César Echeverría; Dayán Sanhueza; Eduardo Castro; Jorge Olivares; Macarena Bastías; Sebastián Wolter; Waldo Díaz
EPI_ISL_895745, EPI_ISL_895767, EPI_ISL_895834	Molecular biology division, Institute of Clinical Biochemistry and Diagnostics, Charles University, Faculty of Medicine in Hradec Králové and University Hospital Hradec Králové	Molecular biology division, Institute of Clinical Biochemistry and Diagnostics, Charles University, Faculty of Medicine in Hradec Králové and University Hospital Hradec Králové	Helena Kovaříková; Ivana Baranová; Jitka Novotná; Kateřina Hrochová; Kateřina Pehlíková; Petr Brož; Tereza Baťková; Vladimír Palička. Cooperation project with BioVendor-R&D and bioinformatics company BIOXSYS s r.o.
EPI_ISL_2448657, EPI_ISL_2626244, EPI_ISL_3101330	Molecular diagnostic laboratory of Federal Budget Institution of Science "Central Research Institute of Epidemiology" of The Federal Service on Customers' Rights Protection and Human Well-being Surveillance	Group of Genomics and Postgenomic Technologies of Central Research Institute of Epidemiology	Akimkin V.G.; Berlina Y.Y.; Bulanenko V.P.; Cherkashina A.S.; Dohoyan A.Y.; Golubeva A.G.; Kaptelova V.V.; Kondrasheva L.Y.; Korneenko E.V.; Nadtoka M.I.; Saenko S.S.; Samoilov A.E.; Shipulina O.Y.; Sinitsyn S.O.; Smirnova Y.S.; Solovieva E.D.; Speranskaya A.S.; Tivanova E.V.; Valdohina A.V.; Zotova M.I.; Zuev S.N.

EPI_ISL_1365023, EPI_ISL_1365027, EPI_ISL_1365030, EPI_ISL_1663659, EPI_ISL_1663663, EPI_ISL_1663669, EPI_ISL_1663676			
see above	Molecular diagnostic unit for viral haemorrhagic fevers and emerging viruses, Bouaké CHU Laboratory	Molecular diagnostic unit for viral haemorrhagic fevers and emerging viruses, Bouaké CHU Laboratory	Adjaratou Traoré; Bamba Fatoumata Touré; Chantal Akoua-Koffi; Coulibaly Mbegnan; Diané Bamourou; Essia Belarbi; Etilé Anoh; Fabian Leendertz; Grit Schubert; Kra Ouffoué; Monemo Pacome; Oby Wayoro; Safiatou Karidioula; Soundélé Maïté
EPI_ISL_614350, EPI_ISL_614379, EPI_ISL_614380, EPI_ISL_681841, EPI_ISL_682058, EPI_ISL_1662584	Molecular diagnostic unit for viral haemorrhagic fevers and emerging viruses, Bouaké CHU Laboratory	Project group Epidemology of Highly Pathogenic Microorganisms, Robert Koch-Institute	Adjaratou Traoré; Bamba Fatoumata Touré; Chantal Akoua-Koffi; Coulibaly Mbegnan; Diané Bamourou; Essia Belarbi; Etilé Anoh; Fabian Leendertz; Grit Schubert; Kra Ouffoué; Monemo Pacome; Safiatou Karidioula; Soundélé Maïté
EPI_ISL_1867965, EPI_ISL_1871906, EPI_ISL_1878641	Molekylær Medicinsk Afdeling, Aarhus University Hospital, Aarhus, Denmark	Aalborg University	Danish Covid-19 Genome Consortium
EPI_ISL_889357, EPI_ISL_889361	Motol University Hospital	Institute of Applied Biotechnologies a.s.	Kateřina Kvapilová; Martin Kašný; Ondřej Brzoň; Pavel Dřevínek; Petr Klempt; Petr Kvapil
EPI_ISL_3031397	Mount Kenya Hospital	USAMRD-A, Basic Science Laboratory	Alan Lemtudo; Beth Mutai; Brian Andika; Carol Kifude; Clement Masakwe; Eric Muthanje; Esther Omuseni; Faith Sigei; Gathii Kimita; George Awinda; John Waitumbi; Josphat Nyataya; Rachel Githii; Rehema Liyai; Stephen Ochola
EPI_ISL_855001	NGS Lab, DNA SOLUTION LTD.	NGS Lab, DNA SOLUTION LTD.	Chowdhury, M.; H.U.; Haider; Hasan; Hosen; K.N.; Khaleque, A.; Khan; Khan, M.; M.B.; M.F.A.; M.H.; M.I.; Rabbi; Rahman, M.; Razu; Sufian, A.
EPI_ISL_2770480	NHLS Charlotte Maxeke Johannesburg Academic Hospital and the University of the Witwatersrand	KRISP, KZN Research Innovation and Sequencing Platform	Bulelani Manene; Florette Treurnicht; Giandhari Jennifer; Kathleen Subramoney; Naidoo Yeshnee; Pillay Sureshnee; San James; Tegally Houriiyah; Tshabuila Derek; Wilkinson Eduan; Yajna Ramphal; de Oliveira Tulio
EPI_ISL_912537, EPI_ISL_2162305, EPI_ISL_2162311, EPI_ISL_2162315, EPI_ISL_2162319, EPI_ISL_2162329, EPI_ISL_2162336, EPI_ISL_2162340, EPI_ISL_2162347, EPI_ISL_2162351, EPI_ISL_2162352, EPI_ISL_2162354, EPI_ISL_2162357, EPI_ISL_2162361, EPI_ISL_2162364, EPI_ISL_2162365, EPI_ISL_2162366, EPI_ISL_2162369, EPI_ISL_2162372, EPI_ISL_2162374, EPI_ISL_2162375, EPI_ISL_2162376, EPI_ISL_2162379, EPI_ISL_2162382			
see above	NHLS Universitas Academic	UFS Virology	D Goedhals; Emmanuel Ogunbayo; MM Nyaga; MT Mogotsi; P Nthiga; PA Bester; T de Oliveira
EPI_ISL_467439, EPI_ISL_602772, EPI_ISL_736970	NHLS-IALCH	KRISP, KZN Research Innovation and Sequencing Platform	Chimukangara B; ChimukangaraB; Giandhari J; Khan S; Lessells R; Mdlalose K; Pillay S; Tegally H; Wilkinson E; York D; de Oliveira T
EPI_ISL_2494465, EPI_ISL_2494476, EPI_ISL_2494502, EPI_ISL_2494505, EPI_ISL_2494507, EPI_ISL_2494510, EPI_ISL_2494514, EPI_ISL_2494515, EPI_ISL_2494516, EPI_ISL_2494517, EPI_ISL_2494541, EPI_ISL_2494542, EPI_ISL_2494621, EPI_ISL_2494624, EPI_ISL_2494625			
see above	NHLS_VIRO	KRISP, KZn Research Innovation and Sequencing Platform	Baillie Vicky; Giandhari Jennifer; Madhi Shabir; Naidoo Yeshnee; Pillay Sureshnee; San James; Tegally Houriiyah; Tshabuila Derek; Wilkinson Eduan; de Oliveira Tulio; du Plessis Jeanine
EPI_ISL_2693824, EPI_ISL_2693831, EPI_ISL_2693881, EPI_ISL_2693883, EPI_ISL_2693895, EPI_ISL_2693896, EPI_ISL_2693898, EPI_ISL_2893259, EPI_ISL_2893263, EPI_ISL_2893265, EPI_ISL_2893271, EPI_ISL_2893282, EPI_ISL_2893285, EPI_ISL_2893287, EPI_ISL_2893288, EPI_ISL_2893293, EPI_ISL_2893298, EPI_ISL_3275356, EPI_ISL_3275368			
see above	NHLS_VIRO Eastern Cape	KRISP, KZN Research Innovation and Sequencing Platform	Emmanuel SJ; Giandhari J; Giandhari Jennifer; Khan S; Laguda-Akingba O; Lessells R; Mdlalose K; Naidoo Yeshnee; Pillay S; Pillay Sureshnee; San James; Tegally H; Tegally Houriiyah; Tshabuila Derek; Wilkinson E; Wilkinson Eduan; Yajna Ramphal; York D; de Oliveira T; de Oliveira Tulio
EPI_ISL_2727180, EPI_ISL_2727185, EPI_ISL_2727186, EPI_ISL_2727194, EPI_ISL_2727196, EPI_ISL_2727197, EPI_ISL_2727198, EPI_ISL_2727206, EPI_ISL_2727216			
see above	NHLS_VIRO Eastern Cape	KRISP, KZn Research Innovation and Sequencing Platform	Emmanuel SJ; Giandhari J; Khan S; Laguda-Akingba O; Lessells R; Mdlalose K; Pillay S; Tegally H; Wilkinson E; York D; de Oliveira T
EPI_ISL_418241, EPI_ISL_420037, EPI_ISL_766863, EPI_ISL_766864, EPI_ISL_766871, EPI_ISL_766873, EPI_ISL_766874, EPI_ISL_766875			
see above	NIC Viral Respiratory Unit - Institut Pasteur of Algeria	National Reference Center for Viruses of Respiratory Infections, Institut Pasteur, Paris	Angela Brisebarre; Etienne Simon-Lorière; Fawzi Derrar; Flora Donati; Marion Barbet; Maud Vanpeene; Mélanie Albert; Méline Bizard; Sylvie Behillil; Sylvie van der Werf; Vincent Enouf
EPI_ISL_3161806, EPI_ISL_3161807, EPI_ISL_3161808	NIC, Viral Respiratory Unit	Virology Departement	Aicha Bensalem; Aissam Hachid; Alia Grad; Amel Benyahia; Fawzi Derrar; Fayez Khardine; Fetouma Doudou; Mohamed Amine Beloufa; Nardjes Hihi
EPI_ISL_454548, EPI_ISL_454561	NIV Influenza	NIV Influenza	Potdar V
EPI_ISL_422403	NMIMR, Department of Virology	WACCBIP, University of Ghana	Abraham Kwabena Anang; Augustina Arjarquah; Bright Adu; Collins M. Morang'a; Dominic S. Y. Amuzu; Erasmus Kotey; Evelyn Bonney; Fred Tei-Maya; George B. Kyei; Gordon A. Awandare; Ivy Asante; Joyce M. Ngoi; Kofi Bonney; Linda Boatemaa; Miriam Eshun; Peter Quashie; Selassie Kumordjie; Vanessa Magnussen; William Ampofo
EPI_ISL_1587554, EPI_ISL_1587569, EPI_ISL_1587570	NOVABIO BERGERAC	CNR Virus des Infections Respiratoires - France SUD	Antonin Bal; Bruno Lina; Gregory Destras; Gwendolyne Burfin; Hadrien Regue; Laurence Josset; Martine Valette; Quentin Semanas
EPI_ISL_1707512, EPI_ISL_1707513, EPI_ISL_1707514, EPI_ISL_1707515, EPI_ISL_1707517	NOVABIO DORDOGNE	CNR Virus des Infections Respiratoires - France SUD	Antonin Bal; Bruno Lina; Gregory Destras; Gwendolyne Burfin; Hadrien Regue; Laurence Josset; Martine Valette; Quentin Semanas
EPI_ISL_1253574, EPI_ISL_1253575, EPI_ISL_1253576	NOVELAB Ingels Vignon	CNR Virus des Infections Respiratoires - France SUD	Antonin Bal; Bruno Lina; Gregory Destras; Gwendolyne Burfin; Hadrien Regue; Laurence Josset; Martine Valette; Quentin Semanas
EPI_ISL_2162093	NS-QEII Health Sciences Centre	National Microbiology Laboratory (NML)	Anna Majer; Anneliese Landgraff; CanCOGeN's metadata curation team; Dan Gaston; Darian Hole; Elsie Grudeski; Gary Van Domselaar; Grace Seo; Janice Pettipas; Jason LeBlanc; Jennifer Tanner; Kirsten Biggar; Madison Chapel; Morag Graham; Natalie Knox; Nathalie Bastien; Philip Mabon; Public Health Agency of Canada CanCOGeN team; Rhiannon Huzarewich; Russell Mandes; Shari Tyson; Timothy Booth; Todd Hatchette; Yan Li
EPI_ISL_444638	NYU Langone Health	Departments of Pathology and Medicine, New York University School of Medicine	Adriana Heguy; Alireza Khodadadi-Jamayran; Amy Rapkiewicz; Andre M. Ribeiro-dos-Santos; Andrew Lytle; Antonio Serrano; Brendan Belovarac; Christian Marier; Dacia Dimartino; Emily Guzman; Emily Huang; Gael Westby; George Jour; Guomiao Shen; Iman Osman; Jared Pinnell; John Cadley; John Chen; Lawrence H. Lin; Ludovic Boytard; Margaret Black; Maria Aguero-Rosenfeld; Marie Samanovic-Golden; Mark J. Mulligan; Matija Snuderl; Matthew T. Maurano; Megan Hogan; Nick Vulpescu; Paolo Cotzia; Paul Zappile; Peter Meyn; Raquel Ordonez Ciriza; Raven Luther; Sitharam Ramaswami; Tatyana Gindin; Theodore Vougiouklakis; Vanessa Raabe; Xiaojun Feng; Yutong Zhang
EPI_ISL_2800233	Nacionālais medicīnas serviss - laboratorija, SIA	Riga East University Hospital, National Microbiology Reference Laboratory; Eurofins Genomics Europe Sequencing GmbH	Arzu Algulieva; Diāna Dušacka; Dārta Pūpola; Ilva Pole; Inita Balta; Jevgenijs Bodrenko; Jūlija Čevere; Natalja Mikena; Reinis Vangravs; Reinis Zeltmatis; Sergejs Ņikišins; Ģirts Šķenders
EPI_ISL_2674519	Nastavni zavod za javno zdravstvo Primorsko- Goranske Županije	Hrvatski zavod za javno zdravstvo	Irena Tabain; Ivana Ferenčak
EPI_ISL_2894986, EPI_ISL_2894995, EPI_ISL_2894996	National Agency for Public Health, Republic of Moldova	Charité Universitätsmedizin Berlin, Institut für Virologie	Ala Halacu; Barbara Mühlemann; Christian Drosten; Julia Schneider; Julia Tesch; Jörn Beheim-Schwarzbach; Mariana Apostol; Talitha Veith; Terry Jones; Tobias Bleicker; Victor M Corman
EPI_ISL_1805717, EPI_ISL_1805741	National Center for Communicable Diseases (NCCD) National Influenza Center	National Center for Communicable Diseases (NCCD) National Influenza Center	Ankhbayar S; Battur L; Bayasgalan N; Darmaa B; Hideka M; Khishigmunkh Ch; Mina N; Naranzul Ts; Nymadawa P; Seiichiro F; Shinji W; Tsogzolmaa G
EPI_ISL_1854607, EPI_ISL_1854627, EPI_ISL_1854638	National Center of Disease Control and Prevention of the Republic of Armenia	UW Virology Lab	Alexander Greninger; Anahit Hovhannisyan; Andranik Chavushyan; Anna Khazaryan; Arsen Arakelyan; Diana Avetyan; Gisane Khachatyan; Hong Xie; Hovsep Ghazaryan; Keith R. Jerome; Lasata Shrestha; Lyudmila Niazyan; Maria Nikoghosyan; Meei-Li Huang; Michelle Lin; Nelli Muradyan; Pavitra Roychoudhury; Roksana Zakharyan; Shah Mohamed Bakhash; Siras Hakobyan; Tamara Sirunyan
EPI_ISL_2081896, EPI_ISL_2081933, EPI_ISL_2081937, EPI_ISL_2081938, EPI_ISL_2180426, EPI_ISL_2379231, EPI_ISL_2841982, EPI_ISL_2841983			
see above	National Center of Infectious and Parasitic Diseases	National Center of Infectious and Parasitic Diseases	Alexiev; Alexiev et al; Dimitrova; Dobrinov; Donchev; Grigorova I.; Grigorova L.; Hristova; Ivanov; Kantardjiev; Korsun; Philipova; Stoitsova; Stoykov; Trifonova
EPI_ISL_2458825, EPI_ISL_2461515, EPI_ISL_2461706, EPI_ISL_2556860	National Centre for Disease Control (NCDC) Biotechnology Division, Delhi	NCDC Delhi, Biotechnology Division INSACOG	Hema Gogia; Hemlata Lall; Kalaiarasan Ponnusamy; Mahesh S Dhar; Manoj K Singh; Meena Datta; Partha Rakshit; Preeti Madan; Priyanka Singh; Radhakrishnan V. S; Robin Marwal; Sandhya Kabra; Sujeet K Singh; Uma Sharma
EPI_ISL_2492631, EPI_ISL_2492664, EPI_ISL_2492734, EPI_ISL_2493030, EPI_ISL_2493031, EPI_ISL_2493032, EPI_ISL_2493034, EPI_ISL_2493035, EPI_ISL_2493068, EPI_ISL_2493069			
see above	National HIV Reference Laboratory, Ministry of Health, Public Health Institute of Malawi	CERI, Centre for Epidemic Response and Innvoation, Stellenbosch University and KRISP, KZN Research Innovation and Sequencing Platform, UKZN.	Auld A; Chilima B; Chiwaula M; Emmanuel SJ; Giandhari J; Kaba M; Kampira E; Kasambara W; Kim L; Lessells R; Maida A; Mvula B; Mwangomba W; Naidoo Y; Panja L; Pillay S; Tegally H; Wadonda N; Wilkinson E; de Oliveira T
EPI_ISL_1407101, EPI_ISL_1407121, EPI_ISL_1407217, EPI_ISL_2494782, EPI_ISL_2494822, EPI_ISL_2494830, EPI_ISL_2494899, EPI_ISL_2494924, EPI_ISL_2494937, EPI_ISL_2494960, EPI_ISL_2494966, EPI_ISL_2494972, EPI_ISL_2609589, EPI_ISL_2609596			
see above	National HIV Reference Laboratory, Ministry of Health, Public Health Institute of Malawi	KRISP, KZN Research Innovation and Sequencing Platform	Auld A; Chilima B; Chiwaula M; Emmanuel SJ; Giandhari J; Kaba M; Kampira E; Kasambara W; Kim L; Lessells R; Maida A; Mvula B; Mwangomba W; Naidoo Y; Panja L; Pillay S; Tegally H; Wadonda N; Wilkinson E; de Oliveira T
EPI_ISL_1677720	National Health Laboratory	Botswana Institute for Technology Research and Innovation	Dineo Emang Tshiamo. Tefelo Thela; Gape Nyepetsi; Kefentse Arnold Tumedi; Madisa Mine; Maitshwarelo Ignatius Matsheka; Malebogo Kebabonye; Thongbotho Mphoyakgosi
EPI_ISL_560386	National Health Laboratory	Botswana Institute for Technology Research and innovation	Dineo Emang Tshiamo. Gape Nyepetsi; Kefentse Arnold Tumedi; Madisa Mine; Maitshwarelo Ignatius Matsheka; Thongbotho Mphoyakgosi
EPI_ISL_622987	National Health Laboratory Service	National Institute for Communicable Diseases of the National Health Laboratory Service	Allam M; Bhiman JN; Ismail A; Khumalo Z; Kwenda S; Mnyameni F; Mohale T; Mtshali P; Subramoney K
EPI_ISL_1550938,	National Health Laboratory Service,	KRISP, KZN Research Innovation and Sequencing Platform	Emmanuel SJ; Giandhari J; Khan S; Lessells R; Mdlalose K; Naidoo Y; Pillay S; Ramphal U; Tegally H; Wilkinson E; York D; de Oliveira T

EPI_ISL_2086237	South Africa		
EPI_ISL_1371929, EPI_ISL_2617186, EPI_ISL_2617188, EPI_ISL_2617189, EPI_ISL_2617191, EPI_ISL_2617204, EPI_ISL_2617205, EPI_ISL_2617208, EPI_ISL_2617210, EPI_ISL_2617213, EPI_ISL_2617216, EPI_ISL_2688535, EPI_ISL_2688544, EPI_ISL_2688550, EPI_ISL_2688552, EPI_ISL_2688553, EPI_ISL_2688571			
see above	National Health Laboratory Service, South Africa	KRISP, KZn Research Innovation and Sequencing Platform	Emmanuel SJ; Giandhari J; Giandhari Jennifer; Khan S; Lessells R; Maslo C; Mdlalose K; Naidoo Yeshnee; Pillay S; Pillay Sureshnee; San James; Sitharam L; Tegally H; Tegally Houriiyah; Tshabuila Derek; Wilkinson E; Wilkinson Eduan; Yajna Ramphal; York D; de Oliveira T; de Oliveira Tulio
EPI_ISL_3252976, EPI_ISL_3252978	National Health Laboratory, Ministry Of Health And Sports, The Republic Of The Union Of Myanmar	Pathogen Genomics Center, National Institute of Infectious Diseases	Eh Htoo Pe; Htay Htay Tin; Kentaro Itokawa; Makoto Kuroda; Masanori Hashino; Reiko Saito; Rina Tanaka; Tsuyoshi Sekizuka; Wint Wint Phyu
EPI_ISL_456600	National Health Laboratory, Timor-Leste	Microbiological Diagnostic Unit Public Health Laboratory, The Peter Doherty Institute for Infection and Immunity	Antonia da Costa, E.; Baird, R.; Barreto, I.; Caly, L.; Canisia, D.; Dakh, F.; Dolores de Jesus da Costa, M.; Douglas, N.; Francis, J.; Freeman, K.; Jayanti Pereira Tilman, A.; Marr, I.; Sait, M.; Salles de Sousa, A.; Schultz, M.; Seemann, T.; Sherry, N.; Soares da Silva, E.; Wapling, J.; Ximenes, J.
EPI_ISL_770471, EPI_ISL_770474	National Health laboratory	Botswana Institute for Technology Research and Innovation	Dineo Emang Tshiamo. Gape Nyepetsi; Kefentse Arnold Tumedi; Madisa Mine; Maitshwarelo Ignatius Matsheka; Malebogo Kebabonye; Thongbotho Mphoyakgosi
EPI_ISL_2455494, EPI_ISL_2455497, EPI_ISL_2455585	National Hospital for Tropical Diseases	Oxford University Clinical Research Unit, Hanoi, Vietnam	H.Rogier van Doorn on behalf of the OUCRU COVID-19 research group; Le Van Duyet; Nguyen Thi Hong Thuong; Nguyen Thi Kim Chi; Nguyen Thi Tam; Nguyen Thu Trang; Pham Ngoc Thach; Phan Manh Cuong; Thomas Kesteman; Van Dinh Trang
EPI_ISL_511898	National Hospital of Tropical Diseases	Oxford University Clinical Research Unit, Hanoi, Vietnam	H. Rogier van Doorn; Le Nguyen Minh Hoa; Nguyen Thi Hong Thuong; Nguyen Thi Ngoc Diep; Nguyen Thi Tam; Nguyen Thu Trang; Pham Ngoc Thach; Van Dinh Trang; Vu Thi Ngoc Bich; on behalf of the OUCRU COVID-19 research group
EPI_ISL_2695785	National Influenza Center (NIC). Virology department. Institut National d'Hygiène.	National Influenza Center (NIC). Virology department. Institut National d'Hygiène.	Abderrahmane BIMOUHEN; Hassan IHAZMAD; Hicham OUMZIL; Samira BENKERROUM; Zakia REGRAGUI; fatima EL FALAKI and Mohamed RHAJAOUI
EPI_ISL_862077, EPI_ISL_862078	National Influenza Center, Virology Department	National Influenza Center	A Nejati; F Ajaminejad and T Mokhtari Azad; J Yavarian; K Sadeghi; N Ghavvami; NZ Shafiei Jandaghi; V Salimi
EPI_ISL_2285856, EPI_ISL_2285858, EPI_ISL_2285859, EPI_ISL_2285860, EPI_ISL_2285865, EPI_ISL_2285866, EPI_ISL_2535784, EPI_ISL_2535825, EPI_ISL_2873861, EPI_ISL_2873862, EPI_ISL_2873869, EPI_ISL_2873870, EPI_ISL_2873872			
see above	National Influenza Centre	National Influenza Centre	; Benjamiin B. Lindsey; Benjamin H. Foulkes; Dennis Laryea; Ernest Asiedu; Franklin Asiedu-Bekoe; Gordon Awandare; Ivy A. Asante; Joseph Oliver-Commey; Joyce Ngoi; Linda Boatemaa; Lorreta Kwasah; Mathew D. Parker; Michael Marks; Mildred Adusei-Poku; Sharon Hsu; Thushan I de Silva; William K. Ampofo
EPI_ISL_476838	National Influenza Centre for Northern Greece	National Influenza Centre for Northern Greece	Maria Christoforidi
EPI_ISL_410301	National Influenza Centre, National Public Health Laboratory, Kathmandu, Nepal	The University of Hong Kong	Alfonso J. Rodriguez-Morales; Anup Bastola; Basu Dev Pandey; Bibek Kumar Lal; Daniel Chu; Haogao Gu; Leo Poon; Malik Peiris; Ranjit Sah; Runa Jha
EPI_ISL_402125	National Institute for Communicable Disease Control and Prevention (ICDC) Chinese Center for Disease Control and Prevention (China CDC)	National Institute for Communicable Disease Control and Prevention (ICDC) Chinese Center for Disease Control and Prevention (China CDC)	Chen; Dai; F.-H.; Hu, Y.; J.-H.; J.-J.; J.-L. and Zhu; Liu, Y.; Pei; Q.-M.; She; Song; T.-Y.; Tao; Tian; Wang; Wang, W.; Wu, F.; Xu, L.; Y.-L.; Y.-M.; Y.-Y.; Y.-Z.; Yu, B.; Z.-G.; Z.-W.; Zhang; Zhao, S.; Zheng
EPI_ISL_430297, EPI_ISL_450298, EPI_ISL_450301, EPI_ISL_490310, EPI_ISL_504192, EPI_ISL_504237, EPI_ISL_515138, EPI_ISL_2433736, EPI_ISL_2433815, EPI_ISL_2662715			
see above	National Institute for Communicable Diseases of the National Health Laboratory Service	National Institute for Communicable Diseases of the National Health Laboratory Service	A; Allam M; Amoako DG; Bhiman JN; Everatt J; Ismail A; Khumalo Z; Kwenda S; Mahlangu B; Mnguni A; Mnyameni F; Mohale T; Mtshali P; Ntuli N; Scheepers C; Subramoney K; van Heusden P; von Gottberg
EPI_ISL_1914915, EPI_ISL_1914931, EPI_ISL_1914936	National Institute for Communicable Diseases,National Health Laboratory Services, Gauteng, South Africa	National Institute for Communicable Diseases of the National Health Laboratory Service	Amoako DG; Bhiman JN; Ismail A; Mahlangu B; Mohale T; Ntuli N; Scheepers C
EPI_ISL_498693, EPI_ISL_591270, EPI_ISL_850947, EPI_ISL_2170894	National Institute for Viral Disease Control and Prevention, China CDC	National Institute for Viral Disease Control and Prevention, China CDC	; 10; 4&; 7; Baoying Huang3; Cao Chen; Cao Chen3&; Dayan Wang; Dayan Wang3; Dongyan Wang3; Fengqin Li6; George F.Gao; George Fu Gao1; Haibo Sun5; Hong Wang; Hong Wang3; Huilai Ma; Huilai Ma1&; Ji Wang; Ji Wang3&; Jian Cai1; Jianqun Zhang2&; Jianxing Yu1; Jingdong Song; Jun Han; Jun Meng2; Kai Nie; Li Bai6; Li Zhao3; Liang Wang1; LingLing Mao; Lingling Mao5; Mingchun Luan2; Naiying Mao3; Ning Li6; Peihua Niu3; Qian Yang3; Ruqin Gao; Shaofeng Jiang9; Shihong Yang2; Shiwen Wang; Shuangli Zhu3; Tao Ma1; Tianjiao Ji3; Wei Yao2*; Weimin Zhou; WenQing Yao , Wenbo Xu; Wenbo Xu; Wenbo Xu3*; Wenjie Tan; Wenjie Tan3; Wenqing Yao5*; Xiang Ren1; Xiang Zhao; Xiang Zhao3; Yan Zhang3; Yang Song; Yang Song3&; Yanhai Wang; Yanhai Wang3; Yao Meng; Yecheng Yao11; Yenan Feng; Ying Qin1&; Yingwei Sun5; Yong Zhang; Yong Zhang3; Yong Zhang , Bo Zhijian , Jianqun Zhang; Yuchao Wu; Yunting Xia8; Zhaoguo Wang; Zhen Zhu3; Zhijian Bo2; Zhixiao Chen; Zhongjie Li1; Zijian Feng1*
EPI_ISL_2107094, EPI_ISL_2107095, EPI_ISL_2107097, EPI_ISL_2107101	National Institute of Health (NIH) - Federal Government of Somalia	African Centre of Excellence for Genomics of Infectious Diseases (ACEGID), Redeemer's University	A.T.; Abechi; Ajogbasile; Akano; C.A.; C.T.; Eromon; F.V.; Folarin, O.; Happi; I.B.; J.N.; J.U.; K.O.; Kayode; Nosamiefan, I.; Oguzie; Olawoye; Olumade; Oluniyi; P.E.; P.S.; T.J.; Ugwu; Uwanibe
EPI_ISL_833130	National Institute of Health Research and Development	National Institute of Health Research and Development	A; AA; Adam; Agustiningsih; F; Febriyani; Febriyanti; HA; HD; Hariastuti; Herman; Herna; IL; Ikawati; Indalao; J; K; KD; KNA; Kipuw; Kurniawati; Muna; Mursinah; N; NI; NK; NL; Nikmah; Nugraha; Pangesti; Pawestri; Puspa; Puspandari; R; Ramadhany; Rizki; Rukminiati; Setiawaty; Soekarso; Subangkit; Susanti; Susilarini; T; UA; V; Wibowo; Y
EPI_ISL_1279947, EPI_ISL_2099853	National Institute of Infectious Diseases-Prof. Dr. Matei Bals Molecular Diagnostics Laboratory	National Institute of Infectious Diseases-Prof. Dr. Matei Bals Molecular Diagnostics Laboratory	Andreea Tudor; Corina Casangiu; Dan Otelea; Leontina Banica; Marius Surleac; Ovidiu Vlaicu; Petre Milu; Simona Paraschiv
EPI_ISL_943561	National Institute of Laboratory Medicine and Referral Center	Genomic Research Lab, BCSIR	A. K. M. Shamsuzzaman; Abu Sayeed Mohammad Mahmud; Arifa Akram; Asish Kumar Ghosh; Barna Goswami; Eshrar Osman; Iffat Jahan; Mahmuda Yeasmin; Md. Ahashan Habib; Md. Maruf Ahmed Molla; Md. Murshed Hasan Sarkar; Md. Saddam Hossain; Md. Salim Khan; Mohammad Samir Uzzaman; Shahina Akter; Tanjina Akhtar Banu; Tasnim Nafisa
EPI_ISL_2323222, EPI_ISL_2380410	National Institute of Public Health	Charles University, Faculty of Science, BIOCEV, OMICS Genomics	Blanka Hamplová; Ingrid Poláková; Jana Šmahelová; Jiří Novák; Magdalena Jančářová; Ruth Tachezy; Sebastian Cristian Treitli; Vladimír Hampl; Zoltán Füssy; Štěpánka Hrdá
EPI_ISL_1261163, EPI_ISL_1261260	National Institute of Public Health	National Reference Laboratory for Influenza and Respiratory Viruses CZE	Alexander Nagy; Dusan Trnka; Helena Jirincova; Jaromira Vecerova; Timotej Suri
EPI_ISL_2562079	National Institute of Public Health	State Veterinary Institute Prague	A; D; H; J; Jirincova; Nagy; Suri; T; Trnka; Vecerova
EPI_ISL_1659614	National Institute of Public Health - National Institute of Hygiene	1. Academic Center for Pathomorphological and Genetic-Molecular Diagnostics ltd, Bialystok, Poland 2. National Institute of Public Health - National Institute of Hygiene, Warsaw, Poland	Anetta Sulewska; Jacek Nikliński; Janusz Dzięcioł; Joanna Kiśluk; Katarzyna Zacharczuk; Konrad Raczkowski; Magdalena Nowakowska; Małgorzata Sadkowska-Todys; Piotr Karabowicz; Piotr Majewski; Przemysław Biecek. Joanna Reszeć; Radosław Charkiewicz; Tomasz Wołkowicz
EPI_ISL_2928004, EPI_ISL_2928005, EPI_ISL_2928006, EPI_ISL_2928007, EPI_ISL_2928008, EPI_ISL_2928009, EPI_ISL_2928010, EPI_ISL_2928011, EPI_ISL_2928012			
see above	National Institute of Public Health, Bujumbura,Burundi, MRC/UVRI & LSHTM Uganda Research Unit	MRC/UVRI & LSHTM Uganda Research Unit, National Institute of Public Health	Alexis Niyomwungere; Anatole Nkeshimana; Dan Lule Bugembe; Deogratius Ssemwanga; Jerome Nkurunziza; Léopold Ouedraogo; Matthew Cotten; My V.T. Phan; Pontiano Kaleebu
EPI_ISL_431833	National Institutes of Health, University of the Philippines Manila	Philippine Genome Center, University of the Philippines System	Alessandra C. Sanchez; Benedict A. Maralit; Bernard Demot; Carlo M. Lapid; Christina Tan; El King D. Morado; Eva Maria Cutiongco-de la Paz; Francis A. Tablizo; Haifa L.Gaza; Jan Michael C. Yap; Jarvin E. Nipales; Jo-Hannah S. Llames; John Mark Velasco; Joshua Gregor A. Dizon; Joy Ann Petronio-Santos; Julius Aaron Mejia; Kris P. Punayan; Kristianne Arielle D. Gabriel; Maribell Dollete; Marissa M. Alejandria; Raul V. Destura; Shana F. Genavia; Shebna Rose D. Fabilloren; Shiela Mae M. Araiza; Sonia Salamat; and Cynthia P. Saloma
EPI_ISL_1335479	National Laboratory for Health, Environment and Food, OMM, Celje	CISLD (Clinical Institute of Special Laboratory Diagnostics), University Children's Hospital, University Medical Center Ljubljana	Ana Grom; Barbara Jenko Bizjan; Jernej Kovač; Katarina Kozmos; Marko Pokorn; Maruša Debeljak; Robert Šket; Tadej Battelino; Tine Tesovnik
EPI_ISL_2001884, EPI_ISL_2273281	National Laboratory for Health, Environment and Food, OMM, Celje	NLZOH (National Laboratory for Health, Environment and Food) / CISLD (Clinical Institute of Special Laboratory Diagnostics), University Children's Hospital, University Medical Center Ljubljana	Aleksander Kocuvan; Aleksander Mahnic; Alenka Štorman; Ana Grom; Barbara Jenko Bizjan; Daša Kavka / Jernej Kovač; Kaja Tominc; Katarina Kozmos; Maja Rupnik; Marko Pokorn; Maruša Debeljak; Mateja Borinc; Maša Jarčič; Nika Gobec; Robert Šket; Sandra Janezic; Tadej Battelino; Tine Tesovnik; Tjasa Žohar Čretnik
EPI_ISL_2162562, EPI_ISL_2789193, EPI_ISL_3316531	National Laboratory for Health, Environment and Food, OMM, Maribor	NLZOH (National Laboratory for Health, Environment and Food) / CISLD (Clinical Institute of Special Laboratory Diagnostics), University Children's Hospital, University Medical Center Ljubljana	Aleksander Kocuvan; Aleksander Mahnic; Alenka Štorman; Ana Grom; Andrej Golle / Jernej Kovač; Barbara Jenko Bizjan; Kaja Tominc; Katarina Kozmos; Maja Rupnik; Marko Pokorn; Maruša Debeljak; Maša Jarčič; Mojca Cimerman; Nika Gobec; Nika Volmajer; Robert Šket; Sandra Janezic; Tadej Battelino; Tine Tesovnik; Tjasa Žohar Čretnik; Tjaša Žohar Čretnik
EPI_ISL_512639	National Laboratory for Influenza/Virology reference laboratory, Public Health Center of the Ministry of Health of Ukraine	Respiratory Virus Unit, Microbiology Services Colindale, Public Health England	Dr. Iryna Demchyshyna; PHE Covid Sequencing Team
EPI_ISL_647971, EPI_ISL_647980, EPI_ISL_1191826, EPI_ISL_1191831, EPI_ISL_1191833, EPI_ISL_1191834	National Microbiology Reference Laboratory	Quadram Institute Bioscience	Agnes Juru; Alexander Goredema; Ana-Victoria Gutierrez; Andrew J. Page; Andrew Tarupiwa; Barbra Murwira; Beuty Makamure; Charles Nyagupe; David Baker; Faustinos T Takawira; Gaetan Thilliez; Gemma Kay; Gibson Mhlanga; Hlanai Gumbo; Isaac Phiri; Justin O'Grady; Kenneth K Maeka; Leonardo de Oliveira Martins; Muchaneta Mugabe; Portia Manangazira; Raiva Simbi; Robert Kingsley; Sekesai Zinyowera; Tapfumanei Mashe; Tatenda Takawira; Thanh Le Viet
EPI_ISL_2492565, EPI_ISL_2492708, EPI_ISL_2492913	National Microbiology Reference Laboratory, Ministry of Health, Harare, Zimbabwe	CERI, Centre for Epidemic Response and Innvoation, Stellenbosch University and KRISP, KZN Research Innovation and Sequencing Platform, UKZN.	Agnes Juru; Air Comodor Dr J. Chimedza; Charles Nyagupe; Dr Raiva Simbi; Emmanuel SJ; Giandhari J; Hlanai Gumbo; Kenneth Maeka; Naidoo Y; Pillay S; Tapfumanei Mashe; Tatenda Takawira; Tegally H; Wilkinson E; de Oliveira T
EPI_ISL_1095620	National Public Health Center, COVID	National Public Health Center, National Biosafety Laboratory	Bernadett Pályi; Dániel Déri; Judit Henczkó; Norbert Solymosi; Nóra Magyar; Zoltán Kis

	Laboratory		
EPI_ISL_2479957, EPI_ISL_2479975, EPI_ISL_2479985	National Public Health Laboartory	CSIR-Institute of Genomics and Integrative Biology	Jyoti Acharya; Lokbandhu Chaudhary; Priya Jha; Runa Jha; Suni Dangol
EPI_ISL_3235116, EPI_ISL_3235120, EPI_ISL_3235155	National Public Health Laboratory	Laboratory of Respiratory Viruses and Measles, Oswaldo Cruz Institute, FIOCRUZ	Alice Sampaio Rocha; Ana Carolina Mendonca; Anna Carolina Paixao; Elisa Calvalcante Pereira; Fernando Motta; Luciana Appolinario; Marilda Siqueira on behalf of the Fiocruz COVID-19 Genomic Surveillance Network; Michelle Hamilton; Paola Resende; Renata Serrano Lopes; Taina Venas
EPI_ISL_845546, EPI_ISL_845548, EPI_ISL_845549, EPI_ISL_845550, EPI_ISL_845551	National Public Health Laboratory, Cameroon	African Centre of Excellence for Genomics of Infectious Diseases (ACEGID), Redeemer's University	Oluniyi P.E. et al
EPI_ISL_2450770	National Public Health Laboratory, Ministry of Health, Ministry of Health, Republic of South Sudan	South Sudan Ministry of Health, WHO South Sudan, MRC/UVRI & LSHTM Uganda Research Unit	Abe G. Abias; Dan Lule Bugembe; Dennis Kenyi Lodiongo; James Ayei; John Rumunu; Joseph Francis Wamala; Juma John HM; Lul Lojok Deng; Matthew Cotten; My V.T. Phan; Pontiano Kaleebu; Richard Lino Loro Lako; Sudhir Bunga
EPI_ISL_536435, EPI_ISL_626631, EPI_ISL_825065, EPI_ISL_1098834, EPI_ISL_1719887, EPI_ISL_2349711, EPI_ISL_2508703, EPI_ISL_2508995, EPI_ISL_2509024			
see above	National Public Health Laboratory, National Centre for Infectious Diseases	National Public Health Laboratory, National Centre for Infectious Diseases	Cui L; Grace Jie Yin Ngan; Lin Cui; Lin RTP; Mak TM; Octavia S; Raymond Tzer Pin Lin; Royce Ang; Sophie Octavia; Tze Minn Mak; Zhenyang Zhou; Zhou Z
EPI_ISL_2674076, EPI_ISL_2674077	National Public Health Laboratory/Lumbini Provincial Hospital COVID 19 PCR Lab	National Public Health Laboratory/CSIR-Institute of Genomics and Integrative Biology	National Public Health Laboratory Team /Lumbini Provincial Hospital COVID 19 PCR Lab Team; National Public Health Laboratory Team /Lumbini Provincial Hospital COVID 19 PCR Lab Team
EPI_ISL_2674112	National Public Health Laboratory/Nepal Medical College & Teaching Hospital	National Public Health Laboratory/CSIR-Institute of Genomics and Integrative Biology	National Public Health Laboratory Team/Nepal Medical College & Teaching Hospital COVID 19 PCR Team
EPI_ISL_2095982	National Public Health Surveillance Laboratory	National Public Health Surveillance Laboratory	Ana Steponkiene; Danas Baksa; Jelena Razmuk; Lukas Vasionis; Lukas Zemaitis; Migle Gabrielaite; Svajune Muralyte
EPI_ISL_1273399, EPI_ISL_1273406, EPI_ISL_1273407, EPI_ISL_1273408	National Reference Laboratory - Ministry of Health Maseru Lesotho	National Institute for Communicable Diseases of the National Health Laboratory Service	Amoako DG; Banda R; Bhiman JN; Gorova V; Ismail A; Mahlangu B; Mathabo M; Mohale T; Mooko M; Ntuli N; Scheepers C
EPI_ISL_1073135	National Reference Laboratory, Nigeria Centre for Disease Control.	National Reference Laboratory, Nigeria Centre for Disease Control, Gaduwa,Abuja, Nigeria	Anthony Ahumibe; Chimaobi Chukwu; Dr Chikwe Ihekweazu; Dr Ndodo Nnaemeka; Dr Omoare Adesuyi; Esebanmen Grace; Naidoo Dhamari; Nwando Mba; Olusola Anuoluwapo Akanbi
EPI_ISL_962878	National Virology Reference Laboratory	National Public Health Laboratory, National Centre for Infectious Diseases	Lin Cui; Raymond Tzer Pin Lin; Taib Surita; Tze Minn Mak; Zaini Zainun; Zhenyang Zhou
EPI_ISL_671343	National Virus Reference Laboratory	Irish Coronavirus Sequencing Consortium - Teagasc Moorepark	Calm Walsh; Genuity Ireland
EPI_ISL_501261, EPI_ISL_528457, EPI_ISL_578311, EPI_ISL_959873, EPI_ISL_1118873, EPI_ISL_1181883, EPI_ISL_1499737, EPI_ISL_1696521, EPI_ISL_1785368, EPI_ISL_1791226, EPI_ISL_1891394, EPI_ISL_2240830, EPI_ISL_2612906			
see above	National Virus Reference Laboratory	National Virus Reference Laboratory	Calum Walsh; Charlene Bennet; Charlene Bennett; Cillian F De Gascun; Fiona Crispie; Gabriel Gonzalez; Guerrino Macori; Jonathan Dean; Matthew McCabe; Michael Carr; Paul Cotter; Seamus Fanning; Suzie Coughlan; Zoe Yandle
EPI_ISL_770475	National health Laboratory	Botswana Institute for Technology Research and Innovation	Dineo Emang Tshiamo. Gape Nyepetsi; Kefentse Arnold Tumedi; Madisa Mine; Maitshwarelo Ignatius Matsheka; Malebogo Kebabonye; Thongbotho Mphoyakgosi
EPI_ISL_2533882, EPI_ISL_2858619	Naval Medical Research Unit No. 3	Naval Medical Research Center Biological Defense Research Directorate	Andrea E. Luquette; Andrew J. Bennett; Bishwo N. Adhikari; Catherine E. Arnold; Chaselynn M. Watters; Emily K. Stefanov; Francisco Malagon; Kyle A. Long; Logan J. Voegtly; Luis A. Estrella; Michael V. Deschenes; Regina Z. Cer; Stephen M. Eggan; and Kimberly A. Bishop-Lilly
EPI_ISL_2501094	Netcare St Augustines Hospital	KRISP, KZN Research Innovation and Sequencing Platform	Emmanuel SJ; Giandhari J; Khan S; Lessells R; Mdlalose K; Naidoo Y; Pillay S; Ramphal U; Sisonke Team; Tegally H; Wilkinson E; York D; de Oliveira T
EPI_ISL_1250370	Netcare/AMPATH	KRISP, KZN Research Innovation and Sequencing Platform	Emmanuel SJ; Giandhari J; Khan S; Lessells R; Maslo C; Mdlalose K; Pillay S; Sitharam L; Tegally H; Wilkinson E; York D; de Oliveira T
EPI_ISL_696489	New Horizon Clinic wc NZC & NHLS/UCT	KRISP, KZN Research Innovation and Sequencing Platform	Arash Iranzadeh; Bruna Galvao; Carolyn Williamson; Deelan Doolabh; Diana Hardie; Emanuel James San; Houriiyah Tegally; Innocent Mudau; Jennifer Giandhari; Kruger Marais; Lynn Tyers; Marvin Hsiao; Stephen Korsman; Sureshnee Pillay; Tulio de Oliveira
EPI_ISL_825714, EPI_ISL_1073139, EPI_ISL_1381815	Nigeria Centre For Disease Control	National reference Laboratory, NCDC, Gaduwa, Abuja	Anthony Ahumibe; Catherine Okoi; Chimaobi Chukwu; Dr Chikwe Ihekweazu; Dr Ndodo Nnaemeka; Dr Omoare Adesuyi; Esebanmen Grace; Grace Esebanmen; Naidoo Dhamari; Nwando Mba; Olusola Anuoluwapo Akanbi
EPI_ISL_455426	Nigeria Centre for Disease Control	African Centre of Excellence for Genomics of Infectious Diseases (ACEGID), Redeemer's University, Ede, Osun State, Nigeria	Ajogbasile F.V.; Folarin O.A.; Happi C.T.; Ihekweazu C.; Kayode A.; Oguzie J.; Olawoye I.; Olumade T.; Oluniyi P.E.; Uwanibe J.
EPI_ISL_2565890, EPI_ISL_2565891, EPI_ISL_2565893, EPI_ISL_2565894, EPI_ISL_2565897	Nigeria Centre for Disease Control (NCDC)	Africa Centre for Excellence for Genomics of Infectious Diseases (ACEGID), Redeemer's University	A.T.; Abechi; Ajogbasile; Akano; C.A.; C.T.; Eromon; F.V.; Folarin, O.; Happi; I.B.; J.N.; J.U.; K.O.; Kayode; Nosamiefan, I.; Oguzie; Olawoye; Olumade; Oluniyi; P.E.; P.S.; T.J.; Ugwu; Uwanibe
EPI_ISL_1970548, EPI_ISL_1970551	Nigeria Centre for Disease Control (NCDC)	African Centre for Excellence for Genomics of Infectious Diseases (ACEGID), Redeemer's University	A.T.; Abechi; Ajogbasile; Akano; C.A.; C.T.; Eromon; F.V.; Folarin, O.; Happi; I.B.; J.N.; J.U.; K.O.; Kayode; Oguzie; Olawoye; Olumade; Oluniyi; P.E.; P.S.; T.J.; Ugwu; Uwanibe
EPI_ISL_872601, EPI_ISL_872610, EPI_ISL_906302, EPI_ISL_941281, EPI_ISL_941284, EPI_ISL_1242025, EPI_ISL_1242026			
see above	Nigeria Centre for Disease Control (NCDC)	African Centre of Excellence for Genomics of Infectious Diseases (ACEGID), Redeemer's University	Oluniyi P.E. et al
EPI_ISL_455412, EPI_ISL_487106, EPI_ISL_487111, EPI_ISL_527875, EPI_ISL_527876, EPI_ISL_527888, EPI_ISL_527889, EPI_ISL_527891, EPI_ISL_527892, EPI_ISL_729920, EPI_ISL_729921, EPI_ISL_729923, EPI_ISL_729925, EPI_ISL_729937, EPI_ISL_729944, EPI_ISL_729960, EPI_ISL_729966, EPI_ISL_729967, EPI_ISL_729971, EPI_ISL_729979, EPI_ISL_729986, EPI_ISL_730042, EPI_ISL_730043			
see above	Nigeria Centre for Disease Control (NCDC)	African Centre of Excellence for Genomics of Infectious Diseases (ACEGID), Redeemer's University, Ede, Osun State, Nigeria	Ajogbasile F.V.; Folarin O.A.; Happi C.T.; Ihekweazu C.; Kayode A.; Oguzie J.; Olawoye I.; Olumade T.; Oluniyi P.E.; Oluniyi P.E. et al; Uwanibe J.
EPI_ISL_2240754, EPI_ISL_2240771	Nigeria Centre for Disease Control (NCDC)	African Centre of Excellence for Genomics of Infectious Diseases (ACEGID), Redeemer's University	A.T.; Abechi; Ajogbasile; Akano; C.A.; C.T.; Eromon; F.V.; Folarin, O.; Happi; I.B.; J.N.; J.U.; K.O.; Kayode; Nosamiefan, I.; Oguzie; Olawoye; Olumade; Oluniyi; P.E.; P.S.; T.J.; Ugwu; Uwanibe
EPI_ISL_1235667	Nigerian Centre for Disease Control (NCDC)	African Centre of Excellence for Genomics of Infectious Diseases (ACEGID), Redeemer's University	I.B.; Olawoye; et.al
EPI_ISL_1715386	Nigerian Centre for Disease Control (NCDC)	African Centre of Excellence for Genomics of Infectious Diseases, Redeemer's University	I.B. et al; Olawoye
EPI_ISL_2361906, EPI_ISL_2376389	Noguchi Memorial Institute for Medical Research, University of Ghana, Legon, Ghana	Institute of Tropical Medicine, Universitätsklinikum Tübingen, Germany	Abraham Kwabena Anang; Bright Adu; Dorothy Yeboah-Manu; Hilda Opoku Frempong; John Kofi Odoom; Joseph Humphrey Kofi Bonney; Joyce Appiah-Kubi; Keren Okyerebea Attiku; Le Thi Kieu Linh; Quaneeta Mohktar; Sivaramakrishna Rachakonda; Srinivas-reddy Pallerla; Thirumalaisamy P Velavan
EPI_ISL_2333185	Norwegian Institute of Public Health, Department of Virology	Norwegian Institute of Public Health, Department of Virology	Atiya R Ali; Debech Nadia; Engebretsen Serina Beate; Garcia Llorente Ignacio; Hilde Elshaug; Hilde Vollan; Jon Bråte; Kamilla Heddeland Instefjord; Karoline Bragstad; Kathrine Stene-Johansen; Line Victoria Moen; Marie Paulsen Madsen; Olav Hungnes; Pedersen Benedikte Nevjen; Rasmus Riis Kopperud
EPI_ISL_934974	Novabio	CNR Virus des Infections Respiratoires - France SUD	Antonin Bal; Bruno Lina; Gregory Destras; Gwendolyne Burfin; Hadrien Règue; Laurence Josset; Martine Valette; Quentin Semanas
EPI_ISL_735437, EPI_ISL_735446	Nucleic Acid Testing - Rwanda National Reference Laboratory	GIGA Medical Genomics	Bouchra Boujemla; Esperence Umumararungu; Jacob Souopgui; Keith Durkin; Léon Mutesa; Maria Artesi; Marie-Pierre Hayette; Patrick Tuyisenge; Robert Rutayisire; Sabin Nsanzimana; Swaibu Gatare; Sébastien Bontems; Vincent Bours; Yvan Butera
EPI_ISL_925878, EPI_ISL_960230, EPI_ISL_960288, EPI_ISL_960300, EPI_ISL_960302, EPI_ISL_1020288, EPI_ISL_2362524, EPI_ISL_2362525, EPI_ISL_2362526, EPI_ISL_2365914, EPI_ISL_2521987, EPI_ISL_2521988, EPI_ISL_2521989, EPI_ISL_2521992, EPI_ISL_2521993, EPI_ISL_2521994, EPI_ISL_2521995, EPI_ISL_2521996, EPI_ISL_2521997, EPI_ISL_2522090, EPI_ISL_2522096, EPI_ISL_2828496			
see above	Nucleic Acid Testing, National Reference Laboratory	GIGA Medical Genomics	Bouchra Boujemla; Corinne Fasquelle; Esperence Umumararungu; Jacob Souopgui; Keith Durkin; Léon Mutesa; Maria Artesi; Marie-Pierre Hayette; Nathalie Renotte; Patrick Tuyisenge; Reuben Sindayiheba; Robert Rutayisire; Sabin Nsanzimana; Swaibu Gatare; Sébastien Bontems; Vincent Bours; Yvan Butera
EPI_ISL_2779342	Nyamira County Referral Hopital	USAMRD-A, Basic Science Laboratory	Alan Lemtudo; Beth Mutai; Brian Andika; Carol Kifude; Clement Masakwe; Eric Muthanje; Esther Omuseni; Faith Sigei; Gathii Kimita; George Awinda; John Waitumbi; Josphat Nyataya; Rachel Githii; Rehema Liyai; Stephen Ochola
EPI_ISL_2372356, EPI_ISL_2372363	Nyangabwe Hospital HIV Reference Laboratory	Botswana Harvard HIV Reference Laboratory	Boitumelo Zuze; Botshelo Radibe; Dorcas Maruapula; Joseph Makhema; Keoratile Ntshambiwa; Kgomotso Moruisi; Legodile Kooepile; Madisa Mine; Mosepele Mosepele; Ontlametse T. Bareng; Pamela Smith-Lawrence; Roger Shapiro; Shahin Lockman; Sikhulile Moyo; Simani Gaseitsiwe; Thongbotho Mphoyakgosi; Wonderful T. Choga
EPI_ISL_1549134, EPI_ISL_1549135, EPI_ISL_1549136, EPI_ISL_1549137	OLVZ Aalst	OLVZ Aalst	Astrid Holderbeke
EPI_ISL_2372266	OUCRU	OUCRU	Guy Thwaites; Huynh Trung Trieu; Lam Minh Yen; Le Manh Hung; Le Nguyen Truc Nhu; Le Thi Thu Huong; Le Van Tan; Nghiem My Ngoc; Ngo Ngoc Quang Minh; Nguyen Thanh Dung; Nguyen Thanh Phong; Nguyen Thanh Truong; Nguyen Thi Han Ny; Nguyen Thi thu Hong; Nguyen To Anh; Nguyen Tri Dung; Nguyen Van Vinh Chau; Tran Nguyen Hoang Tu; Tran Tan Thanh
EPI_ISL_2406461	Oddar Meanchey Rapid Response	Virology Unit, Institut Pasteur du Cambodge	Cecile Troupin; Chau Darapheak; Chin Savuth; Erik A Karlsson; Jurre Y Siegers; Kraing Sidonn; Leakhena Pum; Ly Sovann; Veasna Duong; Yi Sengdoeurn

	Team		
EPI_ISL_2547351	Okiep CHC	National Health Laboratory Service/University of Cape Town (NHLS/UCT)	Arash Iranzadeh; Bruna Galvao; Carolyn Williamson; Deelan Doolabh; Diana Hardie; Gert Marais; Innocent Mudau; Lynn Tyers; Marvin Hsiao; Stephen Korsman
EPI_ISL_491993, EPI_ISL_492016, EPI_ISL_492020	Oman-NIC	Department of Microbiology and Immunology-SQUH	Abdulla Balkhair; Ahlam Al-Amri; Aisha Al-Amri; Aisha Al-Busaidi; Amina Al Jardani; Fahad Zadjali; Fatma BaAlawi; Hamida AL Barwani; Hanan Al-kindi; Intisar Al-Shukri; Khulood Al-Mammary; Mohammed Al-Tobi; Samiha Al Kharusi; Samira Al-Maruqi; Zeyana AL-Dahmani
EPI_ISL_457706	Oman-NIC	Oman-NIC	Abdulla Balkhair; Ahlam Al-Amri; Aisha Al-Amri; Aisha Al-Busaidi; Amina Al Jardani; Fahad Zadjali; Fatma BaAlawi; Hamida AL Barwani; Hanan Al-kindi; Intisar Al-Shukri; Khulood Al-Mammary; Mohammed Al-Tobi; Samiha Al Kharusi; Samira Al-Maruqi; Zeyana AL-Dahmani
EPI_ISL_2921200, EPI_ISL_2921201, EPI_ISL_2925612, EPI_ISL_2925623, EPI_ISL_2925625	Oman-NIC	Oman-National Influenza Center	Aisha Al-Busaidi; Amina Al Jardani; Bilal Hussain.; Fahad Al Balushi; Hanan Al Kindi; Intisar Al-Shukri; Intisar Al-Shukri.; Laila Al Balushi; Samiha Al Kharusi
EPI_ISL_525429, EPI_ISL_1532301	Oman-National Influenza Center	Biotechnology & OMICs Laboratory	Abdul Latif Khan; Adil Al-Wahaibi; Adil Khan; Ahmed Al Harrasi; Ahmed Al-Harrasi; Ahmed Al-Rawahi; Ahmed N Al-Rawahi; Aisha Al-Amri; Amal Al-Maani; Amina Al-Jardani; Amina Al-Jardani.; Bilal Hussain; Hanan Al-Kindi; Intisar Al-Shukri; Sajjad Asaf; Samiha Al-Kharusi; Samira Al-Mahruqi; Samiya Al-Zadjali; Saqib Bilal; Seif Al-Abri
EPI_ISL_766569	Oman-National Influenza Center	Oman-National Influenza Center	Aisha Al-Busaidi; Amina Al-Jardani; Hamida Al-Barwani; Hanan Al-Kindi; Intisar Al-Shukri; Laila Al-Balushi; Samiha Al-Kharusi; Samira Al-Mahruqi
EPI_ISL_1647382	Orebro University Hospital	Orebro University Hospital	Sundqvist M et al
EPI_ISL_708172	Oslo University Hospital, Department of Medical Microbiology	Norwegian Institute of Public Health, Department of Virology	Hilde Elshaug; Hilde Vollan; Kamilla Heddeland Instefjord; Karoline Bragstad; Kathrine Stene-Johansen; Marie Paulsen Madsen; Olav Hungnes; Rasmus Riis Kopperud
EPI_ISL_2661879	Oswaldo Cruz Institute, FIOCRUZ/CE	Analytical Competence Molecular Epidemiology Lab/ACME, Oswaldo Cruz Foundation, Ceara (FIOCRUZ CE)	Alice Sampaio Rocha; Ana Carolina Mendonca; Anna Carolina Paixao; Elisa Cavalcante Pereira; Fabio Miyajima; Fernando Motta; Luciana Appolinario; Marilda Siqueira on behalf of the Fiocruz COVID-19 Genomic Surveillance Network; Paola Resende; Renata Serrano Lopes; Taina Venas
EPI_ISL_3031381, EPI_ISL_3031391, EPI_ISL_3031396, EPI_ISL_3031399	Othaya Sub-County Hospital	USAMRD-A, Basic Science Laboratory	Alan Lemtudo; Beth Mutai; Brian Andika; Carol Kifude; Clement Masakwe; Eric Muthanje; Esther Omuseni; Faith Sigei; Gathii Kimita; George Awinda; John Waitumbi; Josphat Nyataya; Rachel Githii; Rehema Liyai; Stephen Ochola
EPI_ISL_2695585	PATHCARE LABORATORY	National Institute for Communicable Diseases of the National Health Laboratory Service	Amoako DG; Bhiman JN; Everatt J; Ismail A; Mahlangu B; Mnguni A; Mohale T; Ntuli N; Scheepers C
EPI_ISL_2695543, EPI_ISL_2695556	POLOKWANE MANKWENG HOSPITAL	National Institute for Communicable Diseases of the National Health Laboratory Service	Amoako DG; Bhiman JN; Everatt J; Ismail A; Mahlangu B; Mnguni A; Mohale T; Ntuli N; Scheepers C
EPI_ISL_2493252	PRONTO ATENDIMENTO DE VARGEM GRANDE PAULISTA	Instituto Butantan	Antonio Jorge Martins; Claudia Renata dos Santos Barros; David Schlesinger; Debora Botequio Moretti; Dimas Tadeu Covas; Elaine Cristina Marqueze; Elaine Vieira Santos; Evandra Strazza Rodrigues; Heidge Fukumasu; Jayme Augusto de Souza-Neto; José Salvatore Leister Patané; Luiz Alcantara; Luiz Lehmann Coutinho; Maria Carolina Elias; Maurício Lacerda Nogueira; Rafael dos Santos Bezerra; Raul Machado Neto; Rejane Maria Tommasini Grotto; Ricardo Haddad; Sandra Coccuzzo Sampaio Vessoni; Simone Kashima; Svetoslav Nanev Slavov; Vincent Louis Viala
EPI_ISL_2241612, EPI_ISL_2241613, EPI_ISL_2504094, EPI_ISL_2504099, EPI_ISL_2504101, EPI_ISL_2504102, EPI_ISL_2504103, EPI_ISL_2504104, EPI_ISL_2504105, EPI_ISL_2504106, EPI_ISL_2504107, EPI_ISL_2504108, EPI_ISL_2504110, EPI_ISL_2504112, EPI_ISL_2504114, EPI_ISL_2504115, EPI_ISL_2504116, EPI_ISL_2504117, EPI_ISL_2509333, EPI_ISL_2509334			
see above	Palapye Primary Hospital Laboratory	Botswana Harvard HIV Reference Laboratory	Boitumelo Zuze; Botshelo Radibe; David Lawrence; Dorcas Maruapula; Joseph Makhema; Keoratile Ntshambiwa; Kwana Lechiile; Legodile Kooepile; Letsibogo Gaoraelwe; Madisa Mine; Modisa Motswaledi; Mosepele Mosepele; Ontlametse T. Bareng; Roger Shapiro; Shahin Lockman; Sikhulile Moyo; Sikhulile Wonderful T. Choga; Simani Gaseitsiwe; Thela Tefelo; Thongbotho Mphoyakgosi; Wonderful T. Choga
EPI_ISL_596561, EPI_ISL_596567	Palestinian Ministry of Health	Molecular Genetics Lab	Damien Richard; Dana Najjar; Francois Balloux; Hisham Darwish; Husam Sallam; Issa Shtayeh; Lucy van Dorp; Mahmoud Ruzayqat; Nouar Qutob; Osama Najjar; Zaidoun Salah
EPI_ISL_2035941, EPI_ISL_2035942, EPI_ISL_2035945, EPI_ISL_2035946, EPI_ISL_2035947, EPI_ISL_2035988	Pasteur Institute - Laboratory of Clinical Virology	Pasteur Institute - Laboratory of Clinical Virology	Anissa Chouikha; Henda Triki; Kais Ghedira; Mariem Gdoura; Sondos Haddad; Wasfi Fares
EPI_ISL_596777, EPI_ISL_794715, EPI_ISL_1017678	PathWest Laboratory Medicine WA	PathWest Laboratory Medicine WA Microbial Surveillance Unit	PathWest Laboratory Medicine WA Microbial Surveillance Unit
EPI_ISL_3207498	Pathcare lab	NHLS/UCT	Arash Iranzadeh; Bruna Galvao; Carolyn Williamson; Deelan Doolabh; Diana Hardie; Gert Marais; Innocent Mudau; Lynn Tyers; Marvin Hsiao; Rageema Joseph; Sisonke; Stephen Korsman
EPI_ISL_2013036	Pathcare-Vermaak Centurion	National Institute for Communicable Diseases of the National Health Laboratory Service	Amoako DG; Bhiman JN; Glass A; Gottberg A; Mahlangu B; Mohale T; Ntuli N; Oliveira TD; Scheepers C; Tegally H; Viana R
EPI_ISL_1483230	Pathogen Discovery, Respiratory Viruses Branch, Division of Viral Diseases, Centers for Disease Control and Prevention	Pathogen Discovery, Respiratory Viruses Branch, Division of Viral Diseases, Centers for Disease Control and Prevention	Anna Kelleher; Anna Uehara; Brian Lynch; Clinton R. Paden; Haibin Wang; Han Jia Justin Ng; Jing Zhang; Krista Queen; Peter Cook; Suxiang Tong; Yan Li; Ying Tao
EPI_ISL_684112, EPI_ISL_768646, EPI_ISL_895590	Pathogen Genomics Center, National Institute of Infectious Diseases	Pathogen Genomics Center, National Institute of Infectious Diseases	Kentaro Itokawa; Makoto Kuroda; Masanori Hashino; Rina Tanaka; Tsuyoshi Sekizuka
EPI_ISL_513172, EPI_ISL_677947, EPI_ISL_678170, EPI_ISL_751220	Pathogen Genomics Lab King Abdullah University of Science and Technology(KAUST)	Pathogen Genomics Lab King Abdullah University of Science and Technology(KAUST)	Abbas Al Mutair; Abdulaziz Alahmadi; Afrah Alsomali; Amanda Ooi; Amit Kumar Subudhi; Anwar Hashem; Arnab Pain; Asim Khogeer; Awad Al-Omari; Fadwa Alofi; Fathia Ben Rached; Jumana Taha; Kahled Algithami; Luke Esau; Muhammad Shuaib; Naif Almontashiri; Nashwa Al-khotani; Olga Douvropoulou; Raeece Naeem; Rahul P Salunke; Raushan Nugmanova; Samer Salih; Sara Mfarrej; Sharif Hala
EPI_ISL_2859182	Philippine General Hospital (PGH)	Philippine Genome Center	Alethea R. de Guzman; Anna Ong-Lim; Arianne A. Zamora; Benedict A. Maralit; Carlo M. Lapid; Celia Carlos; Devon Ray Pacial; Diomedes A. Carino; Edsel Maurice Salvaña; El King D. Morado; Elcid Aaron R. Pangilinan; Eva Maria Cutiongco-de la Paz; Francis A. Tablizo; Henrietta Marie Rodriguez; Jaime C. Montoya; Jan Michael C. Yap; Jarvin E. Nipales; Jo-Hannah S. Llames; John Q. Wong; Joshua Gregor A. Dizon; Juan Antonio R. Magalang; Karol Sophia Agape R. Padilla; Kenneth M. Kim; Kris P. Punayan; Krisitna Patriz Dela Cruz; Lindsay Claire D.L. Carandang; Ma. Exanil Plantig; Marc Edsel C. Ayes; Maria Rosario Singh-Vergeire and Cynthia P. Saloma; Maria Sofia L. Yangzon; Marielle M Gamboa; Marissa Alejandria; Nina Francesca Bustamante; Razel Nikka M. Hao; Renato Jacinto Q. Mantaring; Rianna Patricia S. Cruz; Sheila Mae M. Araiza; Yvonne Valerie Austria; Zipporah Mariebelle R. Enriquez; Zyrel V. Mollejon
EPI_ISL_2153969	Philippine Genome Center - Biobank (Region IV-A)	Philippine Genome Center	Alethea R. de Guzman; Anna Ong-Lim; Arianne A. Zamora; Asia Louisa U. Chong; Benedict A. Maralit; Candice Francheska B. Tambaoan; Carlo M. Lapid; Celia Carlos; Devon Ray Pacial; Edsel Maurice Salvaña; El King D. Morado; Eva Maria Cutiongco-de la Paz; Francis A. Tablizo; Irish Coleen A. Asin; Jaime C. Montoya; Jan Michael C. Yap; Jo-Hannah S. Llames; John Q. Wong; Joshua Gregor A. Dizon; Juan Antonio R. Magalang; Karol Sophia Agape R. Padilla; Kenneth M. Kim; Kris P. Punayan; Marc Edsel C. Ayes; Marc Jerrone R. Castro; Maria Rosario Singh-Vergeire and Cynthia P. Saloma; Maria Sofia L. Yangzon; Marissa Alejandria; Razel Nikka M. Hao; Rianna Patricia S. Cruz; Sheila Mae M. Araiza
EPI_ISL_2860018	Philippine Red Cross - Clark Molecular Laboratory	Philippine Genome Center	Alethea R. de Guzman; Anna Ong-Lim; Arianne A. Zamora; Benedict A. Maralit; Carlo M. Lapid; Celia Carlos; Devon Ray Pacial; Diomedes A. Carino; Edsel Maurice Salvaña; El King D. Morado; Elcid Aaron R. Pangilinan; Eva Maria Cutiongco-de la Paz; Francis A. Tablizo; Henrietta Marie Rodriguez; Jaime C. Montoya; Jan Michael C. Yap; Jarvin E. Nipales; Jo-Hannah S. Llames; John Q. Wong; Joshua Gregor A. Dizon; Juan Antonio R. Magalang; Karol Sophia Agape R. Padilla; Kenneth M. Kim; Kris P. Punayan; Krisitna Patriz Dela Cruz; Lindsay Claire D.L. Carandang; Ma. Exanil Plantig; Marc Edsel C. Ayes; Maria Rosario Singh-Vergeire and Cynthia P. Saloma; Maria Sofia L. Yangzon; Marielle M Gamboa; Marissa Alejandria; Nina Francesca Bustamante; Razel Nikka M. Hao; Renato Jacinto Q. Mantaring; Rianna Patricia S. Cruz; Sheila Mae M. Araiza; Yvonne Valerie Austria; Zipporah Mariebelle R. Enriquez; Zyrel V. Mollejon
EPI_ISL_2802119	Phutanang Clinic	NHLS/UCT	Arash Iranzadeh; Bruna Galvao; Carolyn Williamson; Deelan Doolabh; Diana Hardie; Gert Marais; Innocent Mudau; Lynn Tyers; Marvin Hsiao; Stephen Korsman
EPI_ISL_2422181	Platform BIS UZA/UAntwerpen	Labo Klinische Biologie, UZA	Basil Britto Xavier; Christine Lammens; Herman Goossens; Jasmine Coppens; Marie Le Mercier; Veerle Matheeussen
EPI_ISL_1120736	Platform BIS UZA/UAntwerpen	UAntwerp, Laboratory of Medical Microbiology	Basil Britto Xavier; Christine Lammens; Herman Goossens; Jasmine Coppens; Marie Le Mercier; Veerle Matheeussen
EPI_ISL_2447813	Port Elizabeth Laboratory	National Institute for Communicable Diseases of the National Health Laboratory Service	Amoako DG; Bhiman JN; Ismail A; Mahlangu B; Mohale T; Ntuli N; Scheepers C
EPI_ISL_712071	Port Elizabeth Provincial Hospital, National Health Laboratory Services, Eastern Cape, South Africa	National Institute for Communicable Diseases of the National Health Laboratory Service	Allam M; Bhiman JN; Ismail A; Mahlangu B; Mohale T; Ntuli N
EPI_ISL_2779317, EPI_ISL_2779424, EPI_ISL_3031388, EPI_ISL_3031389, EPI_ISL_3031390, EPI_ISL_3031419, EPI_ISL_3031420			
see above	Port Health Clinic, Busia border point	USAMRD-A, Basic Science Laboratory	Alan Lemtudo; Beth Mutai; Brian Andika; Carol Kifude; Clement Masakwe; Eric Muthanje; Esther Omuseni; Faith Sigei; Gathii Kimita; George Awinda; John Waitumbi; Josphat Nyataya; Rachel Githii; Rehema Liyai; Stephen Ochola
EPI_ISL_2779318, EPI_ISL_2779494, EPI_ISL_2779501	Port Health Clinic, Malaba border point	USAMRD-A, Basic Science Laboratory	Alan Lemtudo; Beth Mutai; Brian Andika; Carol Kifude; Clement Masakwe; Eric Muthanje; Esther Omuseni; Faith Sigei; Gathii Kimita; George Awinda; John Waitumbi; Josphat Nyataya; Rachel Githii; Rehema Liyai; Stephen Ochola
EPI_ISL_2790405	Primary Health Care Prijedor	Public Health Institute of Republic of Srpska	Branka Culibrk; Dijana Vukajlovic; Milica Celic; Pava Dimitrijevic; Stanka Tomic; Tatjana Markovic; Zeljka Sumic
EPI_ISL_1647744, EPI_ISL_1749617, EPI_ISL_2657889	Public Health Authority of the Slovak Republic	Laboratory of Genomics and Bioinformatics, Comenius University Science Park	Anna Gičová; Diana Rusňáková; Jakub Styk; Jaroslav Budiš; Miroslav Böhmer; Tatiana Sedláčková; Tomáš Szemes
EPI_ISL_2397276	Public Health Authority of the Slovak Republic	Public Health Authority of the Slovak Republic	Anna Gičová; Barbora Kotvasová; Elena Tichá; Lucia Ševčíková; Miroslav Böhmer; Pavol Mišenko; Terézia Vrabľová; Tomáš Szemes
EPI_ISL_2860640	Public Health Reference Laboratory	Erasmus Medical Center	Anne van der Linden; Annemiek van der Eijk; Bas Oude Munnink; Corine GeurtsvanKessel; David Nieuwenhuijse; Emmanuelle Munger; Irina Chestakova; Marion Koopmans; Marjan Boter; Omar Elahmer-Abdulla Bashein- Rahma Algheriani-Ahlam Alarif; Reina Sikkema; Richard Molenkamp; on behalf of the Dutch national COVID-19 response team.
EPI_ISL_513313	Public Health, United States Air Force	Public Health, United States Air Force School of Aerospace	A.C.; A.K.; A.W.; B.C.; C.R.; Chapleau; Connors; E.A.; Fries; J.R.; Javorina; Lambert; Macias; Meyer; Purves; R.R. and Starr; S.M.

	School of Aerospace Medicine	Medicine	
EPI_ISL_493545, EPI_ISL_493546	Quadram Institute Bioscience	COVID-19 Genomics UK (COG-UK) Consortium	Alexander J Trotter; Alison E. Mather; Alp Aydin; Ana P. Tedim; Anastasia Kolyva; Andrew Bell; Andrew J. Page; Claire Stuart; Dave J. Baker; Gemma L. Kay; John Wain; Justin O'Grady; Leonardo de Oliveira Martins; Lizzie Meadows; Maria Diaz; Mark Webber; Muhammed Yasir; Nabil-Fareed Alikhan; Ngozi Elumogo; Nicholas M. Thomson; Rachael Stanley; Rachel Gilroy; Reenesh Prakash; Samir Dervisevic; Samuel Bloomfield; Steven Rudder; Thanh Le-Viet
EPI_ISL_1300532, EPI_ISL_1306135	Queensland Health Forensic and Scientific Services	Queensland Health Forensic and Scientific Services	Son Nguyen
EPI_ISL_2531934	RIIP	National Reference Center for Viruses of Respiratory Infections, Institut Pasteur, Paris	Angela Brisebarre; Camille Capel; Christophe Malabat; Corinne Maufrais; Etienne Simon-Lorière; Frédéric Lemoine; Louise Lefrançois; Marion Barbet; Maud Vanpeene; Méline Bizard; StéPhanie Guyomard-Rabenirina; Sylvie Behillil; Sylvie Van der Werf; Vincent Enouf
EPI_ISL_2086961, EPI_ISL_2273062, EPI_ISL_2273063, EPI_ISL_2273067, EPI_ISL_2273068, EPI_ISL_2273075, EPI_ISL_2273079, EPI_ISL_2273080, EPI_ISL_2273081, EPI_ISL_2273085, EPI_ISL_2273089, EPI_ISL_2454661, EPI_ISL_2454662, EPI_ISL_2454667, EPI_ISL_2454668, EPI_ISL_2454671, EPI_ISL_2454677, EPI_ISL_2454681, EPI_ISL_2454683, EPI_ISL_2582830, EPI_ISL_2695609, EPI_ISL_2695612, EPI_ISL_2695615, EPI_ISL_2695616, EPI_ISL_2695621, EPI_ISL_2695623, EPI_ISL_2695625, EPI_ISL_2695754, EPI_ISL_2695755, EPI_ISL_2695756, EPI_ISL_2695763, EPI_ISL_2695764, EPI_ISL_2695767, EPI_ISL_2695770, EPI_ISL_2695771			
see above	ROB FERREIRA LABORATORY	National Institute for Communicable Diseases of the National Health Laboratory Service	Amoako DG; Bhiman JN; Everatt J; Ismail A; Mahlangu B; Mnguni A; Mohale T; Ntuli N; Scheepers C
EPI_ISL_763064	RS Kasih Ibu, Surakarta	Universitas Sebelas Maret (UNS); Rumah Sakit UNS (RS-UNS); National Institute of Health Research and Development, Indonesian Ministry of Health.	Afif A Ghufron; Arie A Nugraha; Betty Suryawati; Dewi Puspa; Hana A Pawestri; Hartanti Dian Ika; Hartono; Kartika; Maryani; Revi G H Novika; Vivi Setiawaty.; Yulia Sari
EPI_ISL_2262254	RS Umum Kartika Pulomas	National Institute of Health Research and Development	Arie Ardiansyah Nugraha; Hana Apsari Pawestri; Hartanti Dian Ikawati; Kartika Dewi Puspa; Krisna Pangesti; Nelly Puspandari; Subangkit; Triyani Soekarso; Vivi Setiawaty
EPI_ISL_454512, EPI_ISL_454520	RSE "National Center for Biotechnology"	RSE "National Center for Biotechnology"	Akbota Rakhmetova; Alexandr Shevtsov; Askar Abdaliyev; Asylulan Amirgazin; Ilyas Akhmetollayev; Ruslan Kalendar; Viktoriya Lutsay; Yerlan Ramankulov; Zabira Aushakhmetova
EPI_ISL_637110, EPI_ISL_637113	Rafik Hariri University Hospital	Microbial Pathogenomics Lab	Georgi Merhi; Rita Feghali; Sima Tokajian; Tamara Salloum
EPI_ISL_450512, EPI_ISL_450515	Rafik Hariri University Hospital	Rafik Hariri University Hospital	Rita Feghali
EPI_ISL_2348635, EPI_ISL_2348646	Rakai Health Sciences Program	MRC/UVRI & LSHTM Uganda Research Unit	Charles Ssuuna; Dan Lule Bugembe; Matthew Cotten; My V.T. Phan; Pontiano Kaleebu; Ronald Moses Galiwango; Steven J Reynolds
EPI_ISL_447029	Ramathibodi Hospital	COVID-19 Network Investigations (CONI) Alliance	Angkana Huang; Anthony R. Jones; Arporn Wangwiwatsin; Bhakbhoom Panthan; Chonticha Klungtong; Ekawat Pasomsub; Elizabeth Batty; Insee Sensorn; Janjira Thaipadungpanit; Khajohn Joonsalak; Kingkan Rakmanee; Krittikorn Kumpornsin; Namfon Kotanan; Stefan Fernandez; Thanat Chookajorn; Theerarat Kochakarn; Treewat Watthanachockchai; Wasun Chantratita; Wudtichai Manasatienkij
EPI_ISL_1740690	Randox Laboratories	Wellcome Sanger Institute for the COVID-19 Genomics UK (COG-UK) Consortium	Cordelia Langford; David K. Jackson; Dominic Kwiatkowski; Ewan Harrison; Ian Johnston; Jeffrey Barrett; John Sillitoe on behalf of the Wellcome Sanger Institute COVID-19 Surveillance Team; Randox Laboratories and Alex Alderton; Roberto Amato; Sonia Goncalves
EPI_ISL_2157357	Red - Regional de Vigilancia Genómica del COVID-19	Laboratory of Respiratory Viruses and Measles, Oswaldo Cruz Institute, FIOCRUZ	Alice Sampaio Rocha; Ana Carolina Mendonca; Anna Carolina Paixao; Claudia Díaz; Eilisa Cavalcante Pereira; Fernando Motta; Luciana Appolinario; Marilda Siqueira on behalf of the Fiocruz COVID-19 Genomic Surveillance Network; Mitzi Castro; Paola Resende; Renata Serrano Lopes; Sandra Paola Paz; Taina Venas
EPI_ISL_1415392, EPI_ISL_1415415	Reference Laboratory of the Ministry of Health	Laboratory of Respiratory Viruses and Measles, Oswaldo Cruz Institute, FIOCRUZ	Alice Sampaio Rocha; Ana Carolina Mendonca; Anna Carolina Paixao; Fernando Motta; Indira Martins; Jessica Edwards; Luciana Appolinario; Marilda Siqueira on behalf of the Fiocruz COVID-19 Genomic Surveillance Network; Paola Resende; Renata Serrano Lopes
EPI_ISL_2982858, EPI_ISL_2982863	Reference Laboratory of the Ministry of Health Royal Victoria Gardens	Laboratory of Respiratory Viruses and Measles, Oswaldo Cruz Institute, FIOCRUZ	Agatha Cristinne Prudencio; Alice Sampaio Rocha; Ana Carolina Mendonca; Anna Carolina Paixao; Elisa Cavalcante Pereira; Fernando Motta; Ighor Leonardo Arantes Gomes; Indira Martins; Jessica Edwards; Luciana Appolinario; Marilda Siqueira on behalf of the Fiocruz COVID-19 Genomic Surveillance Network; Paola Resende; Renata Serrano Lopes; Taina Venas
EPI_ISL_768617	Regional Medical Sciences Center 5 Samut Songkhram	National Institute of Health, Department of Medical Sciences, Ministry of Public Health, Thailand	; Natchaya Khiadsang; Pakorn Piromtong; Pilailuk Okada; Ratana Tacharoenmuang; Siripaporn Phuygun; Sittiporn Parnmen; Sunthareeya Waicharoen; Thanutsapa Thanadachakul; Warawan Wongboot; sirikanda wimol
EPI_ISL_708808	Regional medical sciences center 6 chonburi	National Institute of Health, Department of Medical Sciences, Ministry of Public Health, Thailand	Malinee Chittaganpitch; Pakorn Piromtong; Pilailuk Okada; Siripaporn Phuygun; Sittiporn Parnmen; Sunthareeya Waicharoen; Thanutsapa Thanadachakul; Warawan Wongboot
EPI_ISL_2796509	Research Center Clinical virology, Tehran University of Medical Sciences	Genetics Research Center, University of Social Welfare and Rehabilitation Sciences	Ali Jafarpour; Alireza Abdollahi; Fateme Tavangar; Hossein Najmabadi.; Kimia Kahrizi; Marzieh Mohseni; Seyed Mohammad Jazayeri; Zohreh Fattahi
EPI_ISL_2544703	Research Center for Emerging Viral Infections, Chang Gung University, Taiwan	Research Center for Emerging Viral Infections, Chang Gung University, Taiwan	Carol Wang; Chung-Guei Huang; Hsiao-Chen Tu; Hui-Ying Weng; Hung-Yu Shu; Jason Su; Jora Lin; Kuo-Ming Lee; Po-Wei Huang; Pocky Lai; Shih-Feng Tsai; Shin-Ru Shih; Shu-Li Yang; Tsu-Lan Wu; Yu-Nong Gong; Yung-Feng Lin
EPI_ISL_2742228	Respiratory Virus Unit, Microbiology Services Colindale, Public Health England	COVID-19 Genomics UK (COG-UK) Consortium	PHE Covid Sequencing Team
EPI_ISL_769875	Respiratory Virus Unit, National Infection Service, Public Health England	COVID-19 Genomics UK (COG-UK) Consortium	PHE Covid Sequencing Team
EPI_ISL_906851	Respiratory Viruses Branch, Centers for Disease Control and Prevention	Respiratory Viruses Branch, Centers for Disease Control and Prevention	C.R.; Cook, P.; Li, Y.; Paden; Queen, K.; Tao, Y.; Tong, S.; Uehara, A.; Wang, H.; Zhang, J.
EPI_ISL_1239490	Rob Ferreira Hospital, National Health Laboratory Services, Mpumalanga, South Africa	National Institute for Communicable Diseases of the National Health Laboratory Service	Amoako DG; Bhiman JN; Ismail A; Mahlangu B; Mohale T; Ntuli N; Scheepers C
EPI_ISL_2447838, EPI_ISL_2447870, EPI_ISL_2447871	Rob Ferreira Laboratory	National Institute for Communicable Diseases of the National Health Laboratory Service	Amoako DG; Bhiman JN; Ismail A; Mahlangu B; Mohale T; Ntuli N; Scheepers C
EPI_ISL_2250216	Royal Darwin Hospital Pathology	Microbiological Diagnostic Unit Public Health Laboratory (MDU-PHL)	Caly L.; Druce J.; M.L.; Meumann, E.; N.L.; Sait; Seemann T.; Sherry
EPI_ISL_1063900, EPI_ISL_1063901, EPI_ISL_1063915	Rwanda National Reference Laboratory	Rwanda National Reference Laboratory	Enatha Mukantwari; UMuringa Jeanne d'Arc; Umuringa Jeanne d'Arc
EPI_ISL_1603794	SARS-CoV-2 Sequencing Castilla y Leon-Spain Consortium	SARS-CoV-2 Sequencing Castilla y Leon-Spain Consortium	Antonio Orduña-Domingo; Carlos Fuster Foz; Carmen Aldea-Mansilla; Carmen Gimeno Crespo; David Abad; Gregoria Megías Lobón; Jose María Eiros Bouza; Laura Sánchez de Prada; M. Isabel Fernandez-Natal; Marta Dominguez-Gil; Marta Hernandez; María Antonia García Castro; Mª Fe Brezmes-Valdivieso; Noelia Arenal Andrés; Silvia Rojo; Sonsoles Garcinuño Pérez
EPI_ISL_1046778, EPI_ISL_2131701, EPI_ISL_2131743, EPI_ISL_2131776, EPI_ISL_2337068	SARS-CoV-2 testing team, National Institute of Infectious Diseases	Pathogen Genomics Center, National Institute of Infectious Diseases	Chang-Kweng Lim; Eri Nakayama; Hazuka Y Furihata; Kentaro Itokawa; Makoto Kuroda; Masanori Hashino; Masumichi Saito; Naomi Nojiri; Nozomu Hanaoka; Rina Tanaka; Sana Uchikoba; Shigeru Tajima; Tsuguto Fujimoto; Tsuyoshi Sekizuka
EPI_ISL_2934570	SECP MHU	The Institute of Molecular Biology and Genetics of NASU	M.Tukalo et al.
EPI_ISL_1171547	SYNLAB	GIGA Medical Genomics	Bouchra Boujemla; Cécile Meex; Keith Durkin; Maria Artesi; Marie-Pierre Hayette; Nathalie Renotte; Pierrette Melin; Raphaël Boreux; Sébastien Bontems; Vincent Bours
EPI_ISL_2642768, EPI_ISL_2643648	SYNLAB Eesti OÜ	Department of Microbiology, Institute of Biomedicine and Translational Medicine, University of Tartu	Aare Abroi; Andrio Lahesaare; Arina Shablinskaja; Dagmar Hoidmets; Ene-Ly Jõgeda; Eveli Kallas; Heiki Niglas; Irja Lutsar; Kai Truusalu; Kaisa Truus; Katrin Kaarna; Kristi Huik; Liidia Dotsenko; Lili Azin Milani; Mari-Anne Härma; Mats Hansen; Meri Pauskar; Olga Sadikova; Paul Naaber; Radko Avi; Taavi Päll; Tuuli Reisberg; Ulvi Gerst Talas
EPI_ISL_1217446, EPI_ISL_1281143, EPI_ISL_1437373	SYNLAB MVZ Heidelberg	Robert Koch Institute	
EPI_ISL_1567985	SYNLAB MVZ Leinfelden-Echterdingen	Robert Koch Institute	
EPI_ISL_2267650	SYNLAB MVZ Leverkusen	Robert Koch Institute	
EPI_ISL_586568	Saikrishna Hospital,Mehsana	Gujarat Biotechnology Research Centre	A M Kadri; Afzal Ansari; Apurvasinh Puvar; Chaitanya Joshi; Dinesh Kumar; Harsh Bakshi; Harshadbhai Parmar; Janvi Raval; Komal Patel; Labdhi Pandya; Madhvi Joshi; Maharshi Pandya; Monika Gandhi; Nidhi Patel; Nikha Trivedi; Nitin Savaliya; Pinal Trivedi; R D Dixit; Raghawendra Kumar; Zarna Patel; Zuber Saiyed
EPI_ISL_480072, EPI_ISL_691527	Sakai City Institute of Public Health	Pathogen Genomics Center, National Institute of Infectious Diseases	Hajime Kamiya; Kentaro Itokawa; Makoto Kuroda; Masanori Hashino; Motoi Suzuki; Rina Tanaka; Tatsuya Miyoshi; Tsuyoshi Sekizuka
EPI_ISL_1824065	San Gallicano Dermatological Institute I.F.O.	INMI Lazzaro Spallanzani IRCCS	A Massacci; B Bartolini; E Giombini; E Sperandio; F Santini; G Bonfiglio; GF Inger; M Pallocca
EPI_ISL_479800	Sapporo City Institute of Public Health	Pathogen Genomics Center, National Institute of Infectious Diseases	Asami Ohnishi; Hajime Kamiya; Kentaro Itokawa; Makoto Kuroda; Masanori Hashino; Motoi Suzuki; Rina Tanaka; Tsuyoshi Sekizuka
EPI_ISL_661185, EPI_ISL_676601	Scientific Veterinary Institute Novi Sad	Veterinary Specialized Institute "Kraljevo", Serbia	Afonso, C.; Banovic Djeri, B.; Jankovic, M.; Jovanovic, T.; Knezevic, A.; Petrovic, T.; Sekler, M.; Tesovic, B.; Vidanovic, D.; Volkening, J.
EPI_ISL_696515	Sedgefield Clinic wc SGE & NHLS/UCT	KRISP, KZN Research Innovation and Sequencing Platform	Arash Iranzadeh; Bruna Galvao; Carolyn Williamson; Deelan Doolabh; Diana Hardie; Emanuel James San; Houriiyah Tegally; Innocent Mudau; Jennifer Giandhari; Kruger Marais; Lynn Tyers; Marvin Hsiao; Stephen Korsman; Sureshnee Pillay; Tulio de Oliveira
EPI_ISL_849366, EPI_ISL_2135170, EPI_ISL_2140067, EPI_ISL_2140071,	Servicio Virosis Respiratorias-Departamento Virología-INEI	Instituto Nacional Enfermedades Infecciosas C.G.Malbran	Avaro M.; Baumeister E.; Benedetti E.; Campos J.; Cisterna D.; Dattero ME; Lorenzo F.; Molina V.; Perandones C.; Poklepovich T.; Pontoriero A.; Russo M.; Tuduri E.

EPI_ISL_2158745			
EPI_ISL_691645	Servicio de Microbiología, Hospital Universitario Son Espases	SeqCOVID-SPAIN consortium/IBV(CSIC)	Antonio Oliver and SeqCOVID-SPAIN consortium; Carla López-Causapé; Jordi Reina
EPI_ISL_419687	Servicio de Microbiología. Consorcio Hospital General Universitario de Valencia	Sequencing and Bioinformatics Service and Molecular Epidemiology Research Group. FISABIO-Public Health	Concepcion Gimeno; Fernando Gonzalez-Candelas; Giuseppe D'Auria; Griselda De Marco; Maria Alma Bracho; Maria Dolores Ocete; Neris Garcia-Gonzalez
EPI_ISL_500322, EPI_ISL_509615	Servicio de Microbiología. Hospital Universitario Donostia. OSI Donostialdea. Área de Enfermedades Infecciosas, Grupo de Infección Respiratoria y Resistencia Antimicrobiana. Instituto de Investigación Sanitaria Biodonostia	SeqCOVID-SPAIN consortium/IBV(CSIC)	Gustavo Cilla; Jose Maria Marimón and SeqCOVID-SPAIN consortium; Luis Piñeiro; Milagrosa Montes
EPI_ISL_2567026	Shamir Medical Center (Asaf Harofe)	Shamir Medical Center (Asaf Harofe)	Abu Hamad Ramzia; Adina Bar Chaim; Anna Vishnevsky; Chen Weiner; Nir Rainy; Patricia Benveniste-Lekovitz; Reut Sorek Abramovich; Yevgeni Yegorov
EPI_ISL_416385	Shanghai Public Health Clinical Center, Shanghai Medical College, Fudan University	National Research Center for Translational Medicine (Shanghai), Ruijin Hospital affiliated to Shanghai Jiao Tong University School of Medicine & Shanghai Public Health Clinical Center	Gang Lu; Hongzhou Lu; Saijuan Chen; Shengyue Wang; Xiaonan Zhang; Yun Ling; Yun Tan
EPI_ISL_582125	Sheikh Khalifa Medical City	Molecular Surveillance lab Sheikh Khalifa Medical City	Amirtharaj Francis; Hala Imambaccus; Hiba Saud; Sahar Almarzooqi; Sajeed Abdul; Stefan Weber
EPI_ISL_2086224, EPI_ISL_2086227	ShipMed Inc	KRISP, KZN Research Innovation and Sequencing Platform	Emmanuel SJ; Giandhari J; Lessells R; Naidoo Y; Pillay S; Ramphal U; Tegally H; Wilkinson E; de Oliveira T
EPI_ISL_500570	Singapore General Hospital	Department of Microbiology	Chenhao Li; Karrie Ko; Kern Rei Chng; Kian Sing Chan; Kun Lee Lim; Lynette Oon; Niranjan Nagarajan; Nurdyana Abdul Rahman
EPI_ISL_1284814	Sonic - MVZ Medizinisches Labor Bremen GmbH	Robert Koch Institute	
EPI_ISL_667804, EPI_ISL_1904462	South Eastern Area Laboratory Services (SEALS)	NSW Health Pathology - Institute of Clinical Pathology and Medical Research; Westmead Hospital; University of Sydney	CIDM-PH et al.
EPI_ISL_2928014, EPI_ISL_2928016, EPI_ISL_2928017, EPI_ISL_2928018, EPI_ISL_2928020, EPI_ISL_2928021, EPI_ISL_2928025, EPI_ISL_2928026, EPI_ISL_2928027			
see above	South Sudan Ministry of Health, WHO South Sudan, MRC/UVRI & LSHTM Uganda Research Unit	MRC/UVRI & LSHTM Uganda Research Unit, South Sudan Ministry of Health, WHO South Sudan	Abe G. Abias; Dan Lule Bugembe; Dennis Kenyi Lodiongo; James Ayei; John Rumunu; Joseph Francis Wamala; Juma John HM; Lul Lojok Deng; Matthew Cotten; My V.T. Phan; Pontiano Kaleebu; Richard Lino Loro Lako; Sudhir Bunga
EPI_ISL_3127444, EPI_ISL_3127445	State Key Laboratory for Diagnosis and Treatment of Infectious Diseases, National Clinical Research Center for Infectious Diseases, First Affiliated Hospital, Zhejiang University School of Medicine, Hangzhou, China. 310003	State Key Laboratory for Diagnosis and Treatment of Infectious Diseases, National Clinical Research Center for Infectious Diseases, First Affiliated Hospital, Zhejiang University School of Medicine, Hangzhou, China. 310003	Changzhong Jin; Danrong Shi; Fumin Liu; Haibo Wu; Hangping Yao; Keda Chen; Keda Chen , Zhigang Wu; Lanjuan Li; Linfang Cheng; Min Zheng; Nanping Wu; Shibo Li; Tianhao Weng; Tianhao Weng , Danrong Shi , Linfang Cheng; Xiangyun Lu; Zhigang Wu
EPI_ISL_2779639	State Key Laboratory of Emerging Infectious Diseases, The University of Hong Kong	The University of Hong Kong	Huachen Zhu; Lifeng Li; Yi Guan; Yiu Man Cheung
EPI_ISL_2716628	State Key Laboratory of Pathogen and Biosecurity, Beijing Institute of Microbiology and Epidemiology	State Key Laboratory of Pathogen and Biosecurity, Beijing Institute of Microbiology and Epidemiology	Campbell, A.; Campbell, J.; Gevao; Harding, D.; Lin, L.; Liu, Y.; P.F.; P.S.; Rogers, J.; Sahr; Wurie, I.; Zhang, J.
EPI_ISL_2375923	Stellenbosch Hospital wc STB	NHLS/UCT	Arash Iranzadeh; Bruna Galvao; Carolyn Williamson; Deelan Doolabh; Diana Hardie; Innocent Mudau; Kruger Marais; Lynn Tyers; Marvin Hsiao; Stephen Korsman
EPI_ISL_2032727, EPI_ISL_2278852, EPI_ISL_2418316, EPI_ISL_2418755, EPI_ISL_2419788	Swedish national genomic surveillance program of SARS-CoV-2	The Public Health Agency of Sweden	Alma Brolund; Maria Lind Karlberg; Maximilian Riess; Swedish national genomic surveillance program of SARS-CoV-2
EPI_ISL_2462426, EPI_ISL_2462429	Sydney South West Pathology Service (SSWPS) - Royal Prince Alfred Hospital - NSW Health Pathology	NSW Health Pathology - Institute of Clinical Pathology and Medical Research; Westmead Hospital; University of Sydney	CIDM-PH et al.
EPI_ISL_1159230	Synergy Laboratories	Synergy Laboratories	Megan Cornwell
EPI_ISL_2545909, EPI_ISL_2547038, EPI_ISL_2788800, EPI_ISL_2788837	Synlab Eesti OÜ	1. Laboratory of Communicable Diseases (Estonia); 2. Eurofins Genomics Europe Sequencing GmbH	Liidia Dotsenko et al.
EPI_ISL_1787536	Synlab Haut de France	UMR 8199/1283 EGID	Derhourhi Mehdi
EPI_ISL_2662581, EPI_ISL_2662589, EPI_ISL_2709945, EPI_ISL_2709948, EPI_ISL_2709956, EPI_ISL_2709990, EPI_ISL_2827054, EPI_ISL_2827152			
see above	TAMBO MEMORIAL LABORATORY	National Institute for Communicable Diseases of the National Health Laboratory Service	Amoako DG; Bhiman JN; Everatt J; Ismail A; Mahlangu B; Mnguni A; Mohale T; Ntuli N; Scheepers C
EPI_ISL_1196006	TLC Clinic	National Institute for Communicable Diseases of the National Health Laboratory Service	Amoako DG; Bhiman JN; Ismail A; Mahlangu B; Maphalala GP; Mohale T; Ntuli N; Scheepers C
EPI_ISL_2693000, EPI_ISL_2693004, EPI_ISL_2693006	TSGH-CP molecular lab	TSGH-CP molecular lab	Cherng-Lih Perng; Chien-Wen Chen; Chih-Kai Chang; Feng-Yee Chang; Hsing-Yi Chung; Hung-Sheng Shang; Jung-Chung Lin; Kuo-Ming Yeh; Kuo-Sheng Hung; Ming-Jr JIAN; Sheng-Kang Chiu; Shih-Hung Tsai; Tien-Yao Chang
EPI_ISL_3105872	Taguig City Health Office	Research Institute for Tropical Medicine	Clyde Dapat; Deana Mae Ocampo; Emmanuel Kagning Tsinda; Francisco Gerardo Polotan; Hitoshi Oshitani; Inez Andrea Medado; Jefferson Earl Halog; Joana Ina Manalo; Lei Lanna Dancel; Ma Angelica Tujan; Mariko Saito-Obata; Mayuko Saito; Michiko Okamoto; Samantha Louise Bado
EPI_ISL_1388124	Team W	University Hospital Basel, Clinical Bacteriology	Adrian Egli; Alfredo Mari; Hans Hirsch; Helena MB Seth-Smith; Julia Bielicki; Karoline Leuzinger; Madlen Stange; Manuel Battegay; Simon Fuchs; Tim Roloff
EPI_ISL_966940	Technical Support Units for Scientific Research (UATRS), National Centre for Scientific and Technical Research (CNRST)	Technical Support Units for Scientific Research (UATRS), National Centre for Scientific and Technical Research (CNRST)	Alaoui; Elalaoui; Elannaz, H.; Elouanass, M.; Ennibi; H. and El Fahime, E.; Hemlali, M.; Lahlou; M.A.; Melloul, M.; Rfaki, A.; S.A.; Touil, N.; a.I.
EPI_ISL_2611673, EPI_ISL_2931340	Temporary Specimen Collection Centre at the AsiaWorld-Expo	Hong Kong Department of Health	Alan K.L. Tsang; Dominic N.C. Tsang; Edman T.K. Lam; Ken H.L. Ng; Peter C.W. Yip; Rickjason C.W. Chan
EPI_ISL_1969991	Thai Red Cross Emerging Infectious Diseases Health Science Centre, Chulalongkorn Hospital, Faculty of Medicine, Chulalongkorn University	Thai Red Cross Emerging Infectious Diseases Center and Faculty of Medicine, Chulalongkorn University	Anthony R. Jones; Chonticha Klungthong; Khajohn Joonlasak; Opass Putcharoen; Piyawan Chinnawirotpisan; Rome Buathong; Sopon Iamsirithaworn; Stefan Fernandez; Supaporn Wacharapluesadee; Wichai Thanasopon; Wudtichai Manasatienkij
EPI_ISL_756357, EPI_ISL_1490226, EPI_ISL_2230689, EPI_ISL_2230694, EPI_ISL_2230696, EPI_ISL_2478949, EPI_ISL_2478964, EPI_ISL_2545191, EPI_ISL_2545194			
see above	The Caribbean Public Health Agency	Carrington Lab, Department of PreClinical Sciences, Faculty of Medical Sciences, The University of the West Indies	Adesh Ramsubhag; Anushka Ramjag; Arianne Brown-Jordan; Avery Hinds; Chinna Chinnadurai; Christine V. F. Carrington; Christopher Oura; Gabriel Escobar; Jaya Jayaraman; Jerome Foster; Karla Georges; Kenneth George; Marsha Ivey; Naresh Nandram; Narine Singh; Nikita S. D. Sahadeo; Nuno Faria; Oliver Pybus; Rahul Naidu; Rajini Haraksingh; Rhonda Sealey-Thomas; Risha Singh; Roshan Parasram; Sarah Hill; Sharon Belmar-George; Shawn Charles; Stanley Giddings; SueMin Nathaniel; Vernie Ramkissoon
EPI_ISL_2621679, EPI_ISL_2626658, EPI_ISL_2626661	The Caribbean Public Health Agency	Carrington Lab, Department of Preclinical Sciences, Faculty of Medical Sciences, The University of the West Indies	Anushka Ramjag; Arianne Brown-Jordan; Avery Hinds; Christine V. F. Carrington; Christopher Oura; Gabriel Escobar; Karla Georges; Kenneth George; Naresh Nandram; Nikita S. D. Sahadeo; Nuno Faria; Oliver Pybus; Risha Singh; Sarah Hill; Sharon Belmar-George; SueMin Nathaniel; Vernie Ramkissoon
EPI_ISL_2649754, EPI_ISL_2649790, EPI_ISL_2658357, EPI_ISL_2658366, EPI_ISL_2678129, EPI_ISL_2678165, EPI_ISL_2678168, EPI_ISL_2678169, EPI_ISL_2678171, EPI_ISL_2716569, EPI_ISL_2716570, EPI_ISL_2756572, EPI_ISL_2756582, EPI_ISL_2955590, EPI_ISL_2967981, EPI_ISL_2967984			
see above	The Caribbean Public Health Agency	Carrington Lab, Department of Preclinical Sciences, Faculty of Medical Sciences, The University of the West Indies, St Augustine Campus	Anushka Ramjag; Arianne Brown-Jordan; Avery Hinds; Christine V. F. Carrington; Christopher Oura; Gabriel Escobar; Hazel Laws; Jacquiline Bisesor-McKenzie; Karla Georges; Kenneth George; Naresh Nandram; Nikita S. D. Sahadeo; Nuno Faria; Oliver Pybus; Rhonda Sealey-Thomas; Risha Singh; Roshan Parasram; Sarah Hill; Sharon Belmar-George; Simone Keizer-Beache; SueMin Nathaniel; Vernie Ramkissoon
EPI_ISL_1838080	The National Centre for Cell Science	CSIR-Centre for Cellular and Molecular Biology-INSACOG	Ajay Pillai; Amareshwar Vodapalli; Ara Sreenivas; Archana Bharadwaj Siva; B Himasri; Blessy B John; Dhiraj Paul; Divya Tej Sowpati; INSACOG Consortium team; Karthik Bharadwaj Tallapaka; Lamuk Zaveri; Manoj Kumar Bhat; Mitali Inamdar; Mohak P Gujare; Onkar Kulkarni; Payel Mukherjee; Rakesh K Mishra; Sharath Chandra Thota; Shivang P. Bhanushali; Shreekant Verma; Sofia Banu; Sonal Manik Chavan; Tulasi Nagabandi; Valli Nagalakshmi Undamatla; Viswagithe S L; Yogesh Shouche
EPI_ISL_577628, EPI_ISL_577633, EPI_ISL_693682	The National Institute of Public Health	State Veterinary Institute Prague	A; D; H; J; Jirincova; L; Nagy; Novakova; Trnka; Vecerova

EPI_ISL_424583, EPI_ISL_828618	The National University Hospital of Iceland	deCODE genetics	Agnar Helgason; Alma Moller; Arna B Agustsdottir; Arnaldur Gylfason; Asgeir Sigurdsson; Aslaug Jonasdottir; Berglind Eiriksdottir; Bjarni Thorbjornsson; Brynjar O Jensson; Daniel F Gudbjartsson; Droplaug N Magnusdottir; Elisabet E Gardarsdottir; Emil A Thorarensen; Gardar Sveinbjornsson; Gisli Masson; Gudmundur Georgsson; Gudmundur L Norddahl; Gudrun Sigmundsdottir; Hakon Jonsson; Hannes Eggertsson; Hilma Holm; Ingileif Jonsdottir; Jona Saemundsdottir; Kamilla S Josefsdottir; Kari Stefansson; Karl G Kristinsson; Kjartan R Gudmundsson; Kristin E Sveinsdottir; Kristin E Sveinsdottir; Louise le Roux; Maney Sveinsdottir; Olafia S Gretarsdottir; Olafia S Gretarsdottir; Olafur T Magnusson; Pall Melsted; Patrick Sulem; Run Fridriksdottir; Solvi Rognvaldsson; Thora R Gunnarsdottir; Thordur Kristjansson; Thorolfur Gudnason; Unnur Thorsteinsdottir
EPI_ISL_754229	The Republican Research and Practical Center for Epidemiology and Microbiology (RRPCEM)	WHO National Influenza Centre Russian Federation	Anatoly Krasko; Andrey Komissarov; Anna Ivanova; Artem Fadeev; Daria Danilenko; Dmitry Bazhenov; Dmitry Lioznov; Elena Gasich; Elena Nabieva; Georgii Bazykin; Kirill Bulda; Ksenia Safina; Kseniya Komissarova
EPI_ISL_906060	Tilia Laboratories s.r.o.	Tilia Laboratories s.r.o.	MD; PhD.; Sona Pekova
EPI_ISL_2779295	Trans Nzoia hospital	USAMRD-A, Basic Science Laboratory	Alan Lemtudo; Beth Mutai; Brian Andika; Carol Kifude; Clement Masakwe; Eric Muthanje; Esther Omuseni; Faith Sigei; Gathii Kimita; George Awinda; John Waitumbi; Josphat Nyataya; Rachel Githii; Rehema Liyai; Stephen Ochola
EPI_ISL_756363, EPI_ISL_2230685	Trinidad Public Health Laboratory	Carrington Lab, Department of PreClinical Sciences, Faculty of Medical Sciences, The University of the West Indies	Adesh Ramsubhag; Arianne Brown-Jordan; Avery Hinds; Chinna Chinnadurai; Christine V. F. Carrington; Christopher Oura; Gabriel Escobar; Jaya Jayaraman; Jerome Foster; Karla Georges; Marsha Ivey; Naresh Nandram; Nikita S. D. Sahadeo; Nuno Faria; Oliver Pybus; Rahul Naidu; Rajini Haraksingh; Risha Singh; Roshan Parasram; Sarah Hill; Stanley Giddings; SueMin Nathaniel; Vernie Ramkissoon
EPI_ISL_960152	Tshwaragano Hospital	National Health Laboratory Service/UCT	Arash Iranzadeh; Bruna Galvao; Carolyn Williamson; Deelan Doolabh; Diana Hardie; Innocent Mudau; Kruger Marais; Lynn Tyers; Marvin Hsiao; Stephen Korsman
EPI_ISL_2802112	Tygerdal	NHLS/UCT	Arash Iranzadeh; Bruna Galvao; Carolyn Williamson; Deelan Doolabh; Diana Hardie; Gert Marais; Innocent Mudau; Lynn Tyers; Marvin Hsiao; Stephen Korsman
EPI_ISL_3214676	U.O. Microbiologia Laboratorio Unico Centro Servizi - AUSL della Romagna	U.O. Microbiologia, Laboratorio Unico Centro Servizi - AUSL della Romagna	Giorgio Dirani
EPI_ISL_2694568	UAB "Baltic Medics"	National Public Health Surveillance Laboratory	Ana Steponkiene; Danas Baksa; Jelena Razmuk; Lukas Vasionis; Lukas Zemaitis; Migle Gabrielaite; Svajune Muralyte
EPI_ISL_2625891	UAB Diagnostikos laboratorija	Institute of Biotechnology, Life Sciences Center, Vilnius University	Emilija Vasiliunaite__ Milda Norkiene__ Danguole Ziogiene__ Albertas Timinskas__ Alma Gedvilaite
EPI_ISL_2812359, EPI_ISL_2812363, EPI_ISL_2812364, EPI_ISL_2812367, EPI_ISL_2812373, EPI_ISL_2812374, EPI_ISL_2812384, EPI_ISL_2812389, EPI_ISL_2812401, EPI_ISL_2812405, EPI_ISL_2812413, EPI_ISL_2812427, EPI_ISL_2812428, EPI_ISL_2812429, EPI_ISL_2812431, EPI_ISL_2812436, EPI_ISL_2841639, EPI_ISL_2841640			
see above	UFS Virology	KRISP, KZn Research Innovation and Sequencing Platform	Giandhari Jennifer; Naidoo Yeshnee; Pillay Sureshnee; San James; Tegally Houriiyah; Tshabuila Derek; Wilkinson Eduan; Yajna Ramphal; de Oliveira Tulio
EPI_ISL_628760, EPI_ISL_861459	UHAS COVID-19 Lab	UHAS COVID-19 Lab	John O. Gyapong and the UHAS COVID-19 Lab Team; Jones Gyamfi; Kwabena O. Duedu; Reuben Ayivor-Djanie
EPI_ISL_733234	UMMC-Health	WHO National Influenza Centre Russian Federation	Andrey Komissarov; Anna Ivanova; Artem Fadeev; Daria Danilenko; Dmitry Bazhenov; Dmitry Lioznov; Elena Nabieva; Georgii Bazykin; Ksenia Safina; Kseniya Komissarova; Tatiana Platonova
EPI_ISL_2131453	UMR190-Unité des virus emergents	UMR190-Unité des virus emergents	cecile Baronti
EPI_ISL_1290838, EPI_ISL_1312895	UNIBIO	CNR Virus des Infections Respiratoires - France SUD	Antonin Bal; Bruno Lina; Gregory Destras; Gwendolyne Burfin; Hadrien Regue; Laurence Josset; Martine Valette; Quentin Semanas
EPI_ISL_1707511	UNIBIO ROMANS GAMBETTA	CNR Virus des Infections Respiratoires - France SUD	Antonin Bal; Bruno Lina; Gregory Destras; Gwendolyne Burfin; Hadrien Regue; Laurence Josset; Martine Valette; Quentin Semanas
EPI_ISL_2661466, EPI_ISL_3375856, EPI_ISL_3375859	US Air Force School of Aerospace Medicine	US Air Force School of Aerospace Medicine	Amanda Javorina; Anthony Fries; Carol Garrett; Clarise Starr; Elizabeth Macias; Jennifer Meyer; Sarah Purves; William Gruner
EPI_ISL_2617858	USC Clinical Lab	Los Angeles County Public Health Laboratories	P. Hemarajata et al.
EPI_ISL_734782, EPI_ISL_735094	UZ Leuven, National Reference Laboratory for Coronaviruses, Laboratory Medicine, Leuven, Belgium	KU Leuven, Rega Institute, Clinical and Epidemiological Virology	Bert Vanmechelen; Joan Marti-Carerras; Piet Maes; Tony Wawina-Bokalanga
EPI_ISL_737931, EPI_ISL_737953	Uganda Central Public Health Lab and Uganda Virus Research Institute	MRC/UVRI & LSHTM Uganda Research Unit	Dan Lule Bugembe; Matthew Cotten; My V.T. Phan; Pontiano Kaleebu et al.
EPI_ISL_451198	Uganda Virus Research Institute	MRC/UVRI & LSHTM Uganda Research Unit	Beatrice Dhaala; Dan Lule Bugembe; Deogratius Ssemwanga; Henry Kyobe; Henry Mwebesa; Jane Aceng; John Kayiwa; Jonas Lexow; Julius Lutwama; Matthew Cotten; My V.T Phan; Phionah Tushabe; Pontiano Kaleebu; Stephen Balinandi
EPI_ISL_615094	Umea klinisk mikrobiologi	The Public Health Agency of Sweden	Anna Risberg; Anna-Malin Linde; Karin Tegmark-Wisell; Maria Lind Karlberg; Mattias Haukland; Mia Brytting; Olov Svartstrom; Oskar Karlsson Lindsjo; Petra Edquist; Reza Advani; Sandra Broddesson
EPI_ISL_3023848	Universidad Autonoma de Yucatan	New York Genome Center	Amy Baldwin; Andre Corvelo; Aviles-Gomez E; Ayora-Talavera G; Chan-Gasca M; Dayna M. Oschwald; Flores-Quintal F; Granja-Perez P; Lopez-Coral L.; Michael Zody; Samantha Fennessey; Tom Maniatis; Villanueva-Jorge S; Yam-Pool E
EPI_ISL_523812	Universidad Iberoamericana, Instituto de Medicina Tropical & Salud Global	International Centre for Genetic Engineering and Biotechnology (ICGEB) and ARGO Open Lab Platform	Alejandro Vallejo Degaudenzi; Danilo Licastro; Eileen Riego; Leandro Tapia; Robert Paulino-Ramirez; Simeone Dal Monego; Sreejith Rajasekharan and Alessandro Marcello.; Victor Virgilio Calderon
EPI_ISL_812967	University Clinical Research Center, University of Sciences	University Clinical Research Center, University of Sciences	A.A.; Bane, S.; Dao, S.; Diakite, M.; Diarra, B.; Doumbia, S.; Guindo, I.; Iknane; Kone, A.
EPI_ISL_998080	University College London, Great Ormond Street Hospital for Children NHS Foundation Trust, Imperial College Healthcare NHS Trust	COVID-19 Genomics UK (COG-UK) Consortium	Alison Holmes; Charlotte Williams; Helena Tutill; Jacqueline Findlay; James Price; Judith Breuer; Julianne Brown; Kathryn Harris; Leysa Forrest; Mark Kristiansen; Paola Niola; Paola Resende Silva; Patricia Dyal; Paul Randell; Rachel Williams; Samuel Weeks; Sergi Castellano; Sunando Roy; Tony Brooks; Yasmin Panchbhaya
EPI_ISL_710570	University Hospital Dubrava	Ruđer Boškovic Institute; Forensic Science Centre Ivan Vučetić; University of Zagreb Faculty of Science	Ana Livun; Antonela Blažeković; Boris Maček; Danilo Licastro; Dunja Glavaš; Fran Borovečki; Fuad Ćosović; Gordana Maravić Vlahoviček; Ivan Šamija; Ivana Ćelap; Jasna Kašman; Josipa Skelin; Katarina Marija Tupek; Kristian Vlahoviček; Kristina Gotovac Jereić; Lidija Cvetko-Krajinović; Lucija Basić; Lucija Markulin; Maja Kuzman; Marina Korolija; Mario Štefanović; Mirjana Domazet-Lošo; Paula Štancl; Petra Vrabec; Robert Beližić; Rosa Karlić; Sanja Tadinac; Senčica Pejša; Tomislav Domazet-Lošo; Valentina Đumlijan-Combaj; Vjekoslav Tomaić; Vladimir Krajinović; Željka Mačak Šafranko
EPI_ISL_2790388	University Hospital Foca	Public Health Institute of Republic of Srpska	Branka Culibrk; Dijana Vukajlovic; Milica Celic; Pava Dimitrijevic; Stanka Tomic; Tatjana Markovic; Zeljka Sumic
EPI_ISL_1533411, EPI_ISL_1811189	University Hospitals of Geneva, Laboratory of Virology	HUG, Laboratory of Virology and the Health2030 Genome Center	Ana Rita Goncalves; Deborah Penet; Emmanouil Dermitzakis; Henri Pegeot; Ioannis Xenarios; Keith Harshman; Laurent Kaiser; Lorenzo Cerutti; Melyssa Elies; Samuel Cordey
EPI_ISL_960093	University Medical Center Hamburg Eppendorf	Heinrich Pette Institute, Leibniz Institute for Experimental Virology	Adam Grundhoff; Alexis Robitaille; Johannes Knobloch; Martin Aepfelbacher; Nicole Fischer; Thomas Günther
EPI_ISL_2864613	University of Health and Allied Sciences (UHAS) COVID-19 Testing and Research Centre	University of Health and Allied Sciences (UHAS) COVID-19 Testing and Research Centre	John O. Gyapong and the UHAS COVID-19 Lab Team; Jones Gyamfi; Kwabena O. Duedu; Reuben Ayivor-Djanie
EPI_ISL_2242506, EPI_ISL_2242809, EPI_ISL_2243020, EPI_ISL_2243223	University of Maiduguri Teaching Hospital	International Centre for Genetic Engineering and Biotechnology (ICGEB) and ARGO Open Lab for Genome Sequencing	Alessandro Marcello; Bamidele S Oderinde; Danilo Licastro; Emanuele Orsini; Galadima Gadzama; Marycelin M Baba; Monilade Akinola; Simeone Dal Monego; Zara Wuduri
EPI_ISL_955142, EPI_ISL_955144	University of Sarajevo, Veterinary Faculty, Laboratory for Molecular Diagnostic and Research Laboratory	University of Sarajevo, Veterinary Faculty, Laboratory for Molecular Diagnostic and Research Laboratory	Alić-Šeho A.; Goletić T.; Goletić Š.; Hodžić A.; Jažić A.; Nicević M.; Softić A.; Terzić I.; Terzić I. Jažić A.; Šabić E.
EPI_ISL_485393	University of Ulsan College of Medicine and Asan Medical Center	University of Ulsan College of Medicine and Asan Medical Center	Heungsup Sung; Jaewoong Lee; Jina Lee; Jiwon Jung; Jongsik Chun; Kihyun Lee; Kuenyoul Park; Kyu-Hwa Hur; Mauricio Chalita; Mi-Na Kim; Seok-Hwan Yoon; Sung-Han Kim; and Hae Kyung Lee
EPI_ISL_977294, EPI_ISL_977337, EPI_ISL_977351, EPI_ISL_977372, EPI_ISL_2803448, EPI_ISL_2803514, EPI_ISL_2803517, EPI_ISL_2803518, EPI_ISL_2803521, EPI_ISL_2803564, EPI_ISL_2803583, EPI_ISL_2803584, EPI_ISL_2803607, EPI_ISL_2803610, EPI_ISL_2803629, EPI_ISL_2803630, EPI_ISL_2803631, EPI_ISL_2803632, EPI_ISL_2803635, EPI_ISL_2803636, EPI_ISL_2803638, EPI_ISL_2803639, EPI_ISL_2803645, EPI_ISL_2803646, EPI_ISL_2803666, EPI_ISL_2803673, EPI_ISL_2803677, EPI_ISL_2803678, EPI_ISL_2803681, EPI_ISL_2803682, EPI_ISL_2803683, EPI_ISL_2803695, EPI_ISL_2803701, EPI_ISL_2803711, EPI_ISL_2803718, EPI_ISL_2803720, EPI_ISL_2803724, EPI_ISL_2803728, EPI_ISL_2803729, EPI_ISL_2803730, EPI_ISL_2803731, EPI_ISL_2803743			
see above	University of Zambia, School of Veterinary Medicine	UNZAVET and PATH	Daniel Bridges; Mulenga Mwenda-Chimfwembe; Ngonda Saasa; ZNPHI and ZGSC
EPI_ISL_1348629, EPI_ISL_1348645	Universitätsklinikum Heidelberg	Robert Koch Institute	
EPI_ISL_2840601	Urban Institute for Disease Prevention and Control	National Institute of Health, Department of Medical Sciences, Ministry of Public Health, Thailand	; Natchaya Khiadsang; Nuttida Thongpramul; Pakorn Piromtong; Pilailuk Okada; Ratana Tacharoenmuang; Siripaporn Phuygun; Sittiporn Parnmen; Sunthareeya Waicharoen; Thanutsapa Thanadachakul; Warawan Wongboot; sirikanda wimol
EPI_ISL_2382664, EPI_ISL_2382671	VERMAAK	National Institute for Communicable Diseases of the National Health Laboratory Service	Amoako DG; Bhiman JN; Ismail A; Mahlangu B; Mohale T; Ntuli N; Scheepers C
EPI_ISL_2360464, EPI_ISL_2360535, EPI_ISL_2360595, EPI_ISL_2360775, EPI_ISL_2360900, EPI_ISL_2360925, EPI_ISL_2494585			
see above	Vaccines and Infectious Diseases Analytics Research Unit (VIDA)	KRISP, KZn Research Innovation and Sequencing Platform	Baillie Vicky; Giandhari Jennifer; Madhi Shabir; Naidoo Yeshnee; Pillay Sureshnee; San James; Tegally Houriiyah; Tshabuila Derek; Wilkinson Eduan; de Oliveira Tulio; du Plessis Jeanine
EPI_ISL_1132714, EPI_ISL_1132715	Vaccines and Infectious Diseases Analytics Research Unit (VIDA)	KRISP, KZn Research Innovation and Sequencing Platform	Baillie Vicky; Giandhari Jennifer; Madhi Shabir; Naidoo Yeshnee; Pillay Sureshnee; Tegally Houriiyah; de Oliveira Tulio; du Plessis Jeanine
EPI_ISL_2140695, EPI_ISL_2375990, EPI_ISL_2375993, EPI_ISL_2621061	Victoria Hospital wc VHW	NHLS/UCT	Arash Iranzadeh; Bruna Galvao; Carolyn Williamson; Deelan Doolabh; Diana Hardie; Gert Marais; Innocent Mudau; Kruger Marais; Lynn Tyers; Marvin Hsiao; Stephen Korsman
EPI_ISL_419735	Victorian Infectious Diseases Reference Laboratory (VIDRL)	Victorian Infectious Diseases Reference Laboratory and Microbiological Diagnostic Unit Public Health Laboratory, Doherty Institute	Caly L.; Druce J.; Sait, M.; Schultz M.; Seemann T.; Sherry, N.

EPI_ISL_2779343	Vihiga County referral hospital	USAMRD-A, Basic Science Laboratory	Alan Lemtudo; Beth Mutai; Brian Andika; Carol Kifude; Clement Masakwe; Eric Muthanje; Esther Omuseni; Faith Sigei; Gathii Kimita; George Awinda; John Waitumbi; Josphat Nyataya; Rachel Githii; Rehema Liyai; Stephen Ochola
EPI_ISL_2802125	Villiersdorp Clinic wc VDP	NHLS/UCT	Arash Iranzadeh; Bruna Galvao; Carolyn Williamson; Deelan Doolabh; Diana Hardie; Gert Marais; Innocent Mudau; Lynn Tyers; Marvin Hsiao; Stephen Korsman
EPI_ISL_2226604	Vilniaus universitetas	Institute of Biotechnology, Life Sciences Center, Vilnius University	Albertas Timinskas; Alma Gedvilaite; Danguole Ziogiene; Emilija Vasiliunaite; Milda Norkiene
EPI_ISL_560405	Vilnius University Hospital Santaros Klinikos, Vilnius University	Institute of Biotechnology, Life Sciences Center, Vilnius University and Thermo Fisher Scientific	Albertas Timinskas; Alma Gedvilaite; Aurelija Zvirbliene; Daniel Naumovas; Justinas Slikas; Laimonas Griskevicius; Ligita Jancioriene; Mindaugas Paulauskas
EPI_ISL_914884, EPI_ISL_914886	Vilnius university hospital Santaros Klinikos, Center of Laboratory Medicine	Vilnius University Hospital Santaros Klinikos	Daniel Naumovas; Dovilė Ežerskytė; Ingrida Olendraite; Justinas Šlikas; Rimvydas Norvilas
EPI_ISL_1005231	Vilnius university hospital Santaros Klinikos, Center of Laboratory Medicine	Vilnius university hospital Santaros Klinikos, Center of Laboratory Medicine	Daniel Naumovas; Dovile Ezerskyte; Gytis Dudas; Ingrida Olendraite; Justinas Slikas; Laimonas Griskevicius; Rimvydas Norvilas
EPI_ISL_1130199, EPI_ISL_1130202, EPI_ISL_1130204, EPI_ISL_1195116, EPI_ISL_1259431, EPI_ISL_1259432, EPI_ISL_2019505, EPI_ISL_2375017, EPI_ISL_2375062			
see above	Viollier AG	Department of Biosystems Science and Engineering, ETH Zürich	Andrea Patrignani; Andreia Cabral de Gouvea; Catharine Aquino; Chaoran Chen; Christian Beisel; Christiane Beckmann; Christoph Noppen; David Dreifuss; Deborah Penet; Doris Popovic; Elodie Burcklen; Emmanouil Dermitzakis; Griffin White; Henri Pegeot; Ina Nissen; Ioannis Xenarios; Ivan Topolsky; Jay Tracy; Katharina Jahn; Keith Harshman; Lara Fuhrmann; Laura Neff; Lennart Opitz; Lorenzo Cerutti; Maria Domenica Moccia; Maurice Redondo; Mirjam Feldkamp; Natascha Santacroce; Niko Beerenwinkel; Noemie Santamaria de Souza; Olivier Kobel; Philipp Jablonski; Ralph Schlapbach; Rebecca Denes; Sarah Nadeau; Simon Grüter; Sophie Seidel; Tanja Stadler; Timothy Sykes
EPI_ISL_420840, EPI_ISL_447242, EPI_ISL_513614, EPI_ISL_527524, EPI_ISL_527547, EPI_ISL_527548, EPI_ISL_527553, EPI_ISL_1785567, EPI_ISL_2135841, EPI_ISL_2135843, EPI_ISL_2135844, EPI_ISL_2135845, EPI_ISL_2968537, EPI_ISL_2968539, EPI_ISL_2968551, EPI_ISL_2968556, EPI_ISL_2968689, EPI_ISL_2968690, EPI_ISL_2968691, EPI_ISL_2968701, EPI_ISL_2968706, EPI_ISL_2968711, EPI_ISL_2968714, EPI_ISL_2968715, EPI_ISL_2968718, EPI_ISL_2968721, EPI_ISL_2968723, EPI_ISL_2968726, EPI_ISL_2968728, EPI_ISL_2968730, EPI_ISL_2982501, EPI_ISL_2982505, EPI_ISL_3133659			
see above	Viral Respiratory Lab, National Institute for Biomedical Research (INRB)	Pathogen Sequencing Lab, National Institute for Biomedical Research (INRB)	Allison Black; Amuri Aziza; Andrew Rambaut; Catherine Pratt; Eddy Kinganda-Lusamaki; Edith Nkwembe; Emmanuel Lokilo Lofiko; Francisca Muyembe Mawete; Gabriel Kabamba; Ian Goodfellow; James Hadfield; Jean Claude Makangara; Jean-Jacques Muyembe Tamfum; Josh Quick; Kristian Andersen; Matthias Pauthner; Michael Wiley; Nick Loman; Placide Mbala-Kingebeni; Raphaël Lumembe; Steve Ahuka-Mundeke; Trevor Bedford
EPI_ISL_2499907, EPI_ISL_2499912, EPI_ISL_2549198, EPI_ISL_2549199, EPI_ISL_2657327, EPI_ISL_2657330, EPI_ISL_2657332, EPI_ISL_2657333, EPI_ISL_2657334, EPI_ISL_2657335, EPI_ISL_2657336, EPI_ISL_2657337, EPI_ISL_2657338, EPI_ISL_2657339, EPI_ISL_2657341, EPI_ISL_2657343, EPI_ISL_2657344, EPI_ISL_2657345, EPI_ISL_2657346, EPI_ISL_2657347, EPI_ISL_2657351, EPI_ISL_2657353, EPI_ISL_2657354, EPI_ISL_2657355			
see above	Virology Department, Central Health Laboratory	The Francis Crick Institute	Bahadoor BS; Crawford M; Daniels RS; Goldstone R; Harvey R; Manraj SS; Nicod J; Patel H; Ramuth M; Sonoo J
EPI_ISL_1191600, EPI_ISL_1827669, EPI_ISL_1827690, EPI_ISL_1827692	Virology Department, Victoria Hospital, Plaine-Wilhems, Mauritius	National Institute for Communicable Diseases of the National Health Laboratory Service	Allam M; Amoako DG; Baboo SB; Bhiman JN; Ismail A; Mahlangu B; Manraj SS; Mohale T; Ntuli N; Ramuth M; Scheepers C; Sonoo J
EPI_ISL_3231385, EPI_ISL_3231386, EPI_ISL_3231387, EPI_ISL_3231388, EPI_ISL_3231389, EPI_ISL_3231390, EPI_ISL_3231391, EPI_ISL_3231392, EPI_ISL_3231393, EPI_ISL_3231396, EPI_ISL_3231397, EPI_ISL_3231399, EPI_ISL_3231400, EPI_ISL_3231401, EPI_ISL_3231402, EPI_ISL_3231403			
see above	Virology Department,Central Health Laboratory	UMR PIMIT	Bahadoor BS; David Wilkinson; Manraj SS; Patrick Mavingui; Ramuth M; Sonoo J
EPI_ISL_2955336	Virology Laboratories	Erasmus Medical Center	Abd Moniem Ain shoka; Amel Nagiub; Anne van der Linden; Annemiek van der Eijk; Bas Oude Munnink; Corine GeurtsvanKessel; Dalia Ramadan; David Nieuwenhuijse; Emmanuelle Munger; Galal Mahmoud; Irina Chestakova; Marion Koopmans; Marjan Boter; Mohamed Hassany; Mohamed K. Khalifa; Nancy El Guindy; Ramy Galal; Reina Sikkema; Richard Molenkamp; Salma Sayed; Shymaa A. Showky; Wael H. Roshdy; on behalf of the Dutch national COVID-19 response team.
EPI_ISL_2960150	Virology Laboratories	Erasmus Medical Center Department of Virology	Abd Moniem Ain shoka; Amel Nagiub; Anne van der Linden; Annemiek van der Eijk; Bas Oude Munnink; Corine GeurtsvanKessel; Dalia Ramadan; David Nieuwenhuijse; Emmanuelle Munger; Galal Mahmoud; Irina Chestakova; Marion Koopmans; Marjan Boter; Mohamed Hassany; Mohamed K. Khalifa; Nancy El Guindy; Ramy Galal; Reina Sikkema; Richard Molenkamp; Salma Sayed; Shymaa A. Showky; Wael H. Roshdy; on behalf of the Dutch national COVID-19 response team.
EPI_ISL_1123282, EPI_ISL_1711646	Virology Laboratory of Praia	Institut Pasteur de Dakar	Dia Ndongo; Diagne Moussa Moïse; Diallo Amadou; Diop Mamadou; Faye Ousmane; Kevin Sanders; Loucoubar Cheikh; Mbengue Safietou Sankhe; Ndiaye Ndack; Sall Amadou Alpha; Sanders Kevin; Sankhe Safietou; Tordo Noel; da Luz Lima Mendonça Maria
EPI_ISL_1123287, EPI_ISL_1711649, EPI_ISL_1711653, EPI_ISL_1711654	Virology Laboratory of São Vicente	Institut Pasteur de Dakar	Dia Ndongo; Diagne Moussa Moïse; Diallo Amadou; Diop Mamadou; Faye Ousmane; Kevin Sanders; Loucoubar Cheikh; Mbengue Safietou Sankhe; Nadia Rodrigues; Ndiaye Ndack; Sall Amadou Alpha; Sanders Kevin; Sankhe Safietou; Tordo Noel; da Luz Lima Mendonça Maria
EPI_ISL_508862, EPI_ISL_508863, EPI_ISL_1660225, EPI_ISL_1660227, EPI_ISL_1660249, EPI_ISL_1660255, EPI_ISL_1660257, EPI_ISL_1660273, EPI_ISL_1660274, EPI_ISL_1660287, EPI_ISL_1660290, EPI_ISL_1660300, EPI_ISL_1660301, EPI_ISL_1660312, EPI_ISL_1660315, EPI_ISL_1660329, EPI_ISL_1660330, EPI_ISL_1660336, EPI_ISL_1672387			
see above	Virology Unit, Institut Pasteur de Madagascar	Virology Unit, Institut Pasteur de Madagascar	Angela Brisebarre; Camille Capel; Cara Brook; Cara E. Brook; Christian Ranaivoson; Christophe Malabat; Corinne Maufrais; Cristina M. Tato; Emmanuelle Permal; Etienne Simon-Lorière; Frédéric Lemoine; Helisoa Razafimanjato; Jean-Michel Heraud; Joseph L. DeRisi; Louise Lefrançois; Marion Barbet; Maud Vanpeene; Michelle Tan; Méline Bizard; Norosoa Razanajatovo; Philippe Dussart; Soa Fy Andriamandimby; Sylvie Behillil; Sylvie van der Werf; Tsiry Randriambolamanantsoa; Vida Ahyong; Vincent Enouf; Vololoniaina Raharinosy
EPI_ISL_918366, EPI_ISL_1532811, EPI_ISL_1534543, EPI_ISL_1711990, EPI_ISL_2106247	Virology Unit, Institut Pasteur du Cambodge	Virology Unit, Institut Pasteur du Cambodge	Cecile Troupin; Chau Darapheak; Chin Savuth; Erik A Karlsson; Etienne Simon-Loriere; Jurre Y Siegers; Kraing Sidonn; Leakhena Pum; Ly Sovann; Sokhoun Yann; Teyputita Ou; Veasna Duong; Yi Sengdoeurn
EPI_ISL_411902	Virology Unit, Institut Pasteur du Cambodge.	Virology Unit, Institut Pasteur du Cambodge (Sequencing done by: Jessica E Manning/Jennifer A Bohl at Malaria and Vector Research Research Laboratory, National Institute of Allergy and Infectious Diseases and Vida Ahyong from Chan-Zuckerberg Biohub)	Erik A Karlsson; Jennifer A Bohl; Jessica E Manning.; Philippe Dussart; Veasna Duong; Vida Ahyong
EPI_ISL_3115358, EPI_ISL_3115359	Virology laboratory, University of Maiduguri Teaching Hospital	Africa Centre for Excellence for Genomics of Infectious Diseases (ACEGID), Redeemer's University	A.T.; Abechi; Ajogbasile; Akano; C.A.; C.T.; Eromon; F.V.; Folarin, O.; Happi; I.B.; J.N.; J.U.; K.O.; Kayode; Nosamiefan, I.; Oguzie; Olawoye; Olumade; Oluniyi; P.E.; P.S.; T.J.; Ugwu; Uwanibe
EPI_ISL_2507964	Vita Laboratoriot Oy	Expert Microbiology, National Institute for Health and Welfare	Carita Savolainen-Kopra; Erika Lindh; Haider al-Hello; Jani Halkilahti; Kirsi Liitsola; Niina Ikonen; Olli Vapalahti; Pekka Ellonen; Phuoc Truong; Päivi Laurila; Ravi Kant; Sari Hannula; Soile Blomqvist; Teemu Smura
EPI_ISL_722201	Vitalis Mostar	Alea Genetic Center	Konjhodzic R.; Pecar D.; Salihefendic L.
EPI_ISL_2836853, EPI_ISL_2836861, EPI_ISL_2836864, EPI_ISL_2836868, EPI_ISL_2836871, EPI_ISL_2836872, EPI_ISL_2836875, EPI_ISL_2836880, EPI_ISL_2836883, EPI_ISL_2836885, EPI_ISL_2836886, EPI_ISL_2836887, EPI_ISL_2836889, EPI_ISL_2836890, EPI_ISL_2836894, EPI_ISL_2836898, EPI_ISL_2836901, EPI_ISL_2836903, EPI_ISL_2836904, EPI_ISL_2836906, EPI_ISL_2836907, EPI_ISL_2836909, EPI_ISL_2836910, EPI_ISL_2836919, EPI_ISL_2836920, EPI_ISL_2836923, EPI_ISL_2836927, EPI_ISL_2836931, EPI_ISL_2836936, EPI_ISL_2836942, EPI_ISL_2836951, EPI_ISL_2836958, EPI_ISL_2836966, EPI_ISL_2836969, EPI_ISL_2967998, EPI_ISL_2968008, EPI_ISL_2968018, EPI_ISL_2968019, EPI_ISL_2968020, EPI_ISL_2968023, EPI_ISL_2968029, EPI_ISL_2968030, EPI_ISL_3268115, EPI_ISL_3268120			
see above	WACCBIP, University of Ghana, Accra, Ghana	WACCBIP, University of Ghana, Volta Road, Legon-Accra, Ghana	; Bright K. Yemi; Collins M. Morang'a; Deborah N. A. Mettle; Dominic S. Y. Amuzu; Dominic S.Y. Amuzu; Evelyn B. Quansah; Evelyn Y. Bonney; Frederick M. Tei-Maya; Frederick Tei-Maya; Israel Osei-Wusu; Ivy A. Asante; Joe K. Mutungi; John K. Odoom; Joseph H.K. Bonney; Joyce M. Ngoi; Lawrence Ofori-Boadu; Lucas N. Amenga-Etego; Lucas N. Amenga-Etego and Gordon A. Awandare; Michael Owusu; Mildred Adusei-Poku; Nicaise T. Ndam; Oliver Commey; Paul Owusu-Oduro; Peter K. Quashie; Peter K. Quashie; Philip M. Soglo; Richard Odame Phillips; Samirah Saiid; Samuel Armoo; Sylvester Dassah; Victor Asoala; Vincent Appiah; Violette V. M'cormack; Violette V. M’cormack; William K. Ampofo; Yaw Bediako; and Gordon A. Awandare
EPI_ISL_427321, EPI_ISL_1797436, EPI_ISL_1797437, EPI_ISL_2523488	WHO National Influenza Centre Russian Federation	WHO National Influenza Centre Russian Federation	Andrey Komissarov; Anna Ivanova; Artem Fadeev; Daria Danilenko; Dmitry Lioznov; Elena Nabieva; Georgii Bazykin; Kirill Varchenko; Ksenia Safina; Kseniya Komissarova; Maria Pisareva; Maria Timofeeva; Mariia Sergeeva; Mikhail Bakaev; Nikita Yolshin; Oula Mansour; Tamila Musaeva; Veronika Eder
EPI_ISL_2301442	WVT Laboratory, Corvallis, Oregon	OSU Center for Genome Research and Biocomputing	Oregon State University TRACE Project
EPI_ISL_2365414, EPI_ISL_2365415, EPI_ISL_2365416, EPI_ISL_2365417	WWF Bayanga field laboratory	WWF Bayanga field laboratory	F. H. Leendertz; F. S. Niatou-Singa; M. Ulrich; S. Calvignac-Spencer; T. B. Tombolomako; T. Fuh-Neba; U. Vickos
EPI_ISL_417083	Washington State Department of Health	Seattle Flu Study	Chu etl al
EPI_ISL_2422509, EPI_ISL_2422515, EPI_ISL_2422517, EPI_ISL_2422532, EPI_ISL_2422536, EPI_ISL_2422595, EPI_ISL_2422605, EPI_ISL_2422628			
see above	West African Centre for Cell Biology of Infectious Pathogen, University of Ghana, Legon	WACCBIP, University of Ghana, Volta Road, Legon, Accra	Collins M. Morang'a; Dominic S. Y. Amuzu; Edward Danso Fenteng; Emmanuel Kudjo; Evelyn B. Quansah; Frederick Tei-Maya; Joe K. Mutungi; Joyce M. Ngoi; Lucas N. Amenga-Etego and Gordon A. Awandare; Nicaise T. Ndam; Patrick Tetteh Ababio; Peter K. Quashie; Philip M. Soglo; Samirah Saiid; Theophilus Odoom; Vincent Appiah; Violette M’cormack; William K. Ampofo; Yaw Bediako
EPI_ISL_1255107, EPI_ISL_1255109, EPI_ISL_1255110, EPI_ISL_1255160, EPI_ISL_1255163, EPI_ISL_1255164, EPI_ISL_1255166, EPI_ISL_1255168, EPI_ISL_1255186, EPI_ISL_1255231, EPI_ISL_1255234, EPI_ISL_1255238, EPI_ISL_1255250, EPI_ISL_1255277			
see above	West African Centre for Cell Biology of Infectious Pathogens (WACCBIP), University of Ghana, Accra, Ghana	West African Centre for Cell Biology of Infectious Pathogens (WACCBIP), University of Ghana, Volta Road, Legon-Accra, Ghana	; Abdoulaye B Diallo; Abdul-Karim Abass; Aisha Mohammed; Benjamin Demah Nuertey; Collins M. Morang'a; Dam Kenneth Mibut; Dominic S.Y. Amuzu; Emmanuella Amoako4; Evelyn B. Quansah; Frederick Kumi-Ansah; Frederick Tei-Maya; Gordon A Awandare; Joyce M. Ngoi; Kesego Tapela; Lucas N. Amenga-Etego; Nelson Kibinge; Oliver D Boakye5; Peter K Quashie; Philip M. Soglo; Samirah Saiid; Samuel Kaba Akoriyea; Theophilus Odoom; Vanessa Magnussen; Vincent Appiah; Yaw Bediako
EPI_ISL_1255105	West African Centre for Cell Biology of Infectious Pathogens (WACCBIP), University of Ghana, Volta Road, Legon-Accra, Ghana	West African Centre for Cell Biology of Infectious Pathogens (WACCBIP), University of Ghana, Volta Road, Legon-Accra, Ghana	; Abdoulaye B Diallo; Abdul-Karim Abass; Aisha Mohammed; Benjamin Demah Nuertey; Collins M. Morang'a; Dam Kenneth Mibut; Dominic S.Y. Amuzu; Emmanuella Amoako4; Evelyn B. Quansah; Frederick Kumi-Ansah; Frederick Tei-Maya; Gordon A Awandare; Joyce M. Ngoi; Kesego Tapela; Lucas N. Amenga-Etego; Nelson Kibinge; Oliver D Boakye5; Peter K Quashie; Philip M. Soglo; Samirah Saiid; Samuel Kaba Akoriyea; Theophilus Odoom; Vanessa Magnussen; Vincent Appiah; Yaw Bediako
EPI_ISL_1340864	West Bandung Public Health	West Java Health Laboratory; School of Life Sciences and Technology, Institut Teknologi Bandung	Azzania Fibriani; Cut Nur Cinthia Alamanda; Ema Rahmawati; Isak Solihin; Karimatu Khoirunnisa; Miftahul Faridl; Rifky Waluyajati Rachman; Rini Robiani; Ryan Bayusantika Ristandi
EPI_ISL_2382409	Wisma Atlet	National Institute of Health Research and Development	Arie Ardiansyah Nugraha; Hana Apsari Pawestri; Hartanti Dian Ikawati; Kartika Dewi Puspa; Krisna Pangesti; Nelly Puspandari; Subangkit; Triyani Soekarso; Vivi Setiawaty
EPI_ISL_1196002	Women correctional	National Institute for Communicable Diseases of the National Health Laboratory Service	Amoako DG; Bhiman JN; Ismail A; Mahlangu B; Maphalala GP; Mohale T; Ntuli N; Scheepers C

EPI_ISL_2547364	Worcester Hospital wc WOC	National Health Laboratory Service/University of Cape Town (NHLS/UCT)	Arash Iranzadeh; Bruna Galvao; Carolyn Williamson; Deelan Doolabh; Diana Hardie; Gert Marais; Innocent Mudau; Lynn Tyers; Marvin Hsiao; Stephen Korsman
EPI_ISL_2727335, EPI_ISL_2727347	ZARV/NHLS, Department Medical Virology, University of Pretoria	KRISP, KZn Research Innovation and Sequencing Platform	Adriano Mendes; Amy Strydom; Emmanuel SJ; Giandhari J; Lessells R; Micheala Davids; Naidoo Y; Pillay S; Ramphal U; Sim Mayaphi and Marietjie Venter; Tegally H; Wilkinson E; de Oliveira T
EPI_ISL_2674012	Zavod za javno zdravstvo Šibensko-Kninske Županije	Hrvatski zavod za javno zdravstvo	Irena Tabain; Ivana Ferenčak
EPI_ISL_660069	Zurita & Zurita Laboratorios	Zurita & Zurita Laboratorios	Gabriela Sevillano Camilo Zurita-Salinas Karen Loaiza David Ortega-Paredes Jeannete Zurita
EPI_ISL_2716621	aboratoire National de Référence pour les Fièvres Hémorragiques Virales, Centre Muraz	Centre Muraz	Ange Badjo; Arsène Zongo; Essia Belarbi; Fabian Leendertz; Grit Schubert; Jasmin Schlotterbeck; Saïdou Ouedraogo; Soumeya Ouangraoua; Thérèse Kagone
EPI_ISL_1644990	amedes MVZ Hannover	Robert Koch Institute	
EPI_ISL_2094433, EPI_ISL_2491373, EPI_ISL_2622014	cerballiance-IDF	Cerba lab	Aude Lessenne; Bénédicte Roquebert; Emmanuel Lecorche; Kader Merah; Laura Verdurme; Patrice Herisson; Sabine Trombert-Paolantoni; Stéphanie Haïm-Boukobza; Thierry Collin
EPI_ISL_828071, EPI_ISL_828706	deCODE genetics	deCODE genetics	Agnar Helgason; Alma Moller; Arna B Agustsdottir; Arnaldur Gylfason; Asgeir Sigurdsson; Aslaug Jonasdottir; Berglind Eiriksdottir; Bjarni Thorbjornsson; Brynjar O Jensson; Daniel F Gudbjartsson; Droplaug N Magnusdottir; Elisabet E Gardarsdottir; Emil A Thorarensen; Gardar Sveinbjornsson; Gisli Masson; Gudmundur Georgsson; Gudmundur L Norddahl; Gudrun Sigmundsdottir; Hakon Jonsson; Hannes Eggertsson; Hilma Holm; Ingileif Jonsdottir; Jona Saemundsdottir; Kamilla S Josefsdottir; Kari Stefansson; Karl G Kristinsson; Kjartan R Gudmundsson; Kristin E Sveinsdottir; Louise le Roux; Maney Sveinsdottir; Olafia S Gretarsdottir; Olafur T Magnusson; Pall Melsted; Patrick Sulem; Run Fridriksdottir; Solvi Rognvaldsson; Thora R Gunnarsdottir; Thordur Kristjansson; Thorolfur Gudnason; Unnur Thorsteinsdottir
EPI_ISL_872226, EPI_ISL_1336339, EPI_ISL_1336340	hopital	National Reference Center for Viruses of Respiratory Infections, Institut Pasteur, Paris	Angela Brisebarre; Camille Capel; Combe Patrice; Etienne Simon-Lorière; Gastli Nabil; Marion Barbet; Maud Vanpeene; Méline Bizard; Sylvie Behillil; Sylvie van der Werf; Vincent Enouf
EPI_ISL_1696154, EPI_ISL_1696182	laboratoire Belle Epine	Department of Virology, Henri Mondor University Hospital, Assistance Publique Hôpitaux de Paris, Université Paris-Est Créteil, INSERM U955	Alexandre Soulier; Christophe Rodriguez; Elisabeth Trawinski; Guillaume Gricourt; Jean-Michel Pawlotsky; Melissa N'Debi; Slim Fourati; Vanessa Demontant
EPI_ISL_1235687	laboratorio de salud pública (Bogotá)	Gencore- Universidad de los Andes	Alejandro Gómez; David González; Johana Hernandez Gabriela Delgado; Luisa Sacristán; Marcela Guevara-Suarez; Silvia Restrepo
EPI_ISL_458285	unknown	Bundeswehr Institute of Microbiology	Antwerpen; Bestehorn-Willmann; Eckstein, S.; Handrick, S.; M.C.; M.H.; M.S.; Naija, H.; R. and Ben Moussa, M.; Rehn, A.; Stoecker, K.; Walter; Woelfel
EPI_ISL_463001	unknown	Clinical virology	Fares, W.; Triki, H.
EPI_ISL_437612	unknown	Faculty of Medicine	Buathong, R.; Bunprakob, S.; Ghai, S.; Joyjinda, Y.; Mungaomklang, A.; Petcharat, S.; Plipat; Prasithsirikul, W.; Rodpan, A.; Sirichan, N.; T. and Hemachudha, T.; Wacharapluesadee, S.

Supplementary Table 3 Aggregated locations in the phylogeographic analysis. Several countries have reported only very few A.27 genomes on GISAID, which could lead to problems reliably estimating their incoming and outgoing transition rates in a phylogeographic analysis. To mitigate this, we have aggregated African countries according to the five United Nations subregions, and the European countries according to the United Nations geoscheme, with the exception of France and Germany, as they were the focal points of our study, having contributed many A.27 genomes.

**Location**	**Countries included in this location**	**Number of sequences**
Asia-Pacific (APAC)	India, Australia, Indonesia	4
Benelux	Belgium, The Netherlands, Luxembourg	33
Eastern Africa	Rwanda, Mayotte	13
Middle Africa	Cameroon, Gabon	2
Northern Africa	Tunisia	7
Southern Africa	South Africa	0
Western Africa	Togo, Côte d’Ivoire, Burkina Faso, Benin, Senegal, Nigeria	75
North America	USA	6
Eastern Europe	Slovenia	37
Northern Europe	Denmark, Sweden, UK, Ireland	27
Southern Europe	Greece, Spain, Italy, Turkey	24
Western Europe	Austria, Switzerland	28
France	France	112
Germany	Germany	193
