# Supplementary material for: Antibody escape and global spread of SARS-CoV-2 lineage A.27
Source: Nat Commun. 2022 Mar 3;13:1152. doi: 10.1038/s41467-022-28766-y (PMC8894356; doi:10.1038/s41467-022-28766-y)
Supplement: Supplementary file 3 — Description of Additional Supplementary Files [file 41467_2022_28766_MOESM3_ESM.pdf]

Description of Additional Supplementary Files

File Name: Supplementary Data 1
Description: Input XML files of the phylogeographic analysis.

File Name: Supplementary Data 2
Description: Accession numbers of all A.27 sequences and associated metadata obtained from the RKI (available via https://github.com/robert-koch-institut/SARS-CoV-2-Sequenzdaten_aus_Deutschland).
