# Supplementary material for: Antibody escape and global spread of SARS-CoV-2 lineage A.27
Source: Nat Commun. 2022 Mar 3;13:1152. doi: 10.1038/s41467-022-28766-y (PMC8894356; doi:10.1038/s41467-022-28766-y)
Supplement: Supplementary file 6 — Reporting Summary [file 41467_2022_28766_MOESM6_ESM.pdf]

nature portfolio

Corresponding author(s): Jonas Fuchs
Last updated by author(s): Jan 19, 2022

Reporting Summary

Nature Portfolio wishes to improve the reproducibility of the work that we publish. This form provides structure for consistency and transparency in reporting. For further information on Nature Portfolio policies, see our Editorial Policies and the Editorial Policy Checklist.

Statistics

For all statistical analyses, confirm that the following items are present in the figure legend, table legend, main text, or Methods section.

n/a	Confirmed	
☐	☒	The exact sample size (*n*) for each experimental group/condition, given as a discrete number and unit of measurement
☐	☒	A statement on whether measurements were taken from distinct samples or whether the same sample was measured repeatedly
☐	☒	The statistical test(s) used AND whether they are one- or two-sided<br>*Only common tests should be described solely by name; describe more complex techniques in the Methods section.*
☒	☐	A description of all covariates tested
☒	☐	A description of any assumptions or corrections, such as tests of normality and adjustment for multiple comparisons
☐	☒	A full description of the statistical parameters including central tendency (e.g. means) or other basic estimates (e.g. regression coefficient) AND variation (e.g. standard deviation) or associated estimates of uncertainty (e.g. confidence intervals)
☐	☒	For null hypothesis testing, the test statistic (e.g. *F*, *t*, *r*) with confidence intervals, effect sizes, degrees of freedom and *P* value noted<br>*Give P values as exact values whenever suitable.*
☐	☒	For Bayesian analysis, information on the choice of priors and Markov chain Monte Carlo settings
☒	☐	For hierarchical and complex designs, identification of the appropriate level for tests and full reporting of outcomes
☒	☐	Estimates of effect sizes (e.g. Cohen's *d*, Pearson's *r*), indicating how they were calculated

Our web collection on statistics for biologists contains articles on many of the points above.

Software and code

Policy information about availability of computer code

Data collection	Full-length SARS-CoV-2 genome sequences from Germany were downloaded from GISAID (www.gisaid.org). The GISAID accession numbers and originating laboratories are acknowledged in the manuscript. Sequence data submitted to the RKI was internally accessed.<br>Patient metadata were obtained from local health authorities as part of the genomic surveillance program of the RKI via the national electronic reporting system for surveillance of notifiable infectious diseases (SurvNet).
Data analysis	Pangolin assignment: pangolin (v.3.0.3), pangoLEARN: 2021-05-27.<br>Statistical analysis: GraphPad Prism (v.8.4.2) and R (v.4.0.2).<br>R packages: tmap v.3.3-1, pheatmap v1.0.12 , stringr v1.4.0 and dplyr v1.0.2.<br>Analysis of NGS data: SARS-CoV-2 NGS data was analyzed on the cloud computing bioinformatic platform Galaxy (usegalaxy.eu, covid19.galaxyproject.org/artic/) using the following pipline: fastqs were preprocessed with fastp (v.0.20.1) and mapped using BWA-MEM (v.0.7.17), ARTIC primer sequences were trimmed using ivar trim (v1.9), SNPs and INDELs were called with lofreq (v2.1.5) and annotated with snpeff (v.4.3.1). Consensus sequences were generated with bcftools (v.1.10).<br>Variant frequency visualization: github.com/jonas-fuchs/SARS-CoV-2-analyses (v.1.0).<br>Protein structure visualization: UCSF ChimeraX version: 1.1 (2020-09-09).<br>Phylogenetic tree construction: IQTREE2 v2.1.05, TreeTime v.0.7.4.<br>Phylogenetic tree visualization: https://github.com/evogytis/baltic (from 30-03-2021).<br>Phylogeographic reconstruction: BEAST v1.10.5, BEAGLE v.3.2.0, Tracer v.1.7.<br>Determination of nucleotide profiles: https://gitlab.com/s.fuchs/covsonar (v.1.1.3).<br>Image recording and processing: ZEN 2.6 blue edition v.2.6.76, ImageJ software v.1.53c

For manuscripts utilizing custom algorithms or software that are central to the research but not yet described in published literature, software must be made available to editors and reviewers. We strongly encourage code deposition in a community repository (e.g. GitHub). See the Nature Portfolio guidelines for submitting code & software for further information.

March 2021

Data

Policy information about availability of data

All manuscripts must include a data availability statement. This statement should provide the following information, where applicable:

- Accession codes, unique identifiers, or web links for publicly available datasets
- A description of any restrictions on data availability
- For clinical datasets or third party data, please ensure that the statement adheres to our policy

Full-length SARS-CoV-2 genome sequences: Downloaded from GISAID (www.gisaid.org) and accessable via https://github.com/robert-koch-institut/SARS-CoV-2-Sequenzdaten_aus_Deutschland. Accession numbers are given in the manuscript (Supplementary table 2 and Supplementary table 4).
Africa-focused Nextstrain build: (https://nextstrain.org/ncov/gisaid/africa).
Mutation frequency of different VOCs/VOIs: Downloaded from outbreak.info (2021-07-13).
EM structure of the closed trimeric SARS-CoV-2 spike protein (https://www.rcsb.org/structure/6vxx) and the dimeric 2.04Å crystal structure of ORF8 (https://www.rcsb.org/structure/7JTL) was downloaded from the protein data bank.

All necessary data and information are given in the manuscript.
Source data are provided with this paper.
Input XML files of the phylogeographic analysis is supplied in the Supplementary Files.
The sequence data was submitted to the GISAID data base and are publicly available (Supplementary table 2).
Raw sequencing data of the A.27 swab, isolate and stock have been submitted to the European Nucleotide Archive (https://www.ebi.ac.uk/ena/browser) under the study accession number: ERP134884.

For requests, please contact J.Fuchs, jonas.fuchs@uniklinik-freiburg.de, and M. Panning, marcus.panning@uniklinik-freiburg.de. Requests will be processed within a week.

Field-specific reporting

Please select the one below that is the best fit for your research. If you are not sure, read the appropriate sections before making your selection.

☒ Life sciences ☐ Behavioural & social sciences ☐ Ecological, evolutionary & environmental sciences

For a reference copy of the document with all sections, see nature.com/documents/nr-reporting-summary-flat.pdf

Life sciences study design

All studies must disclose on these points even when the disclosure is negative.

Sample size	Sample sizes were predetermined due to the limited number of vaccinees and convalescent COVID-19 patients that agreed to provide sera for this study. All available samples were used.<br><br>Patients were recruited and patient material was banked at the University Hospital Freiburg; inclusion criteria were: (1) 14 health care workers that received a prime and boost vaccination with the mRNA vaccine bnt162b2/Comirnaty (2) 16 convalescent individuals following a SARS-CoV-2 infection.<br><br>The sample size of the K18-hACE2 transgenic mice was estimated on the basis of experience with other respiratory viruses to give statistical power while minimizing animal use.
Data exclusions	No data were excluded.
Replication	Data were reproduced as biological triplicates (growth kinetics, mAB testing and CD16 activation). Sera of vaccinees and convalescent sera were tested for neutralization in independent duplicates because of limited sera availability.
Randomization	Vaccinated donors and donors with a history of natural SARS-CoV-2 infection were selected based on availability.<br>Age- and sex-matched K18-hACE2 transgenic mice were randomly assigned to the experimental groups.<br>To excluded sampling bias of the patient metadata a set was randomly selected and limited to not fully vaccinated patients for A.27 (100/329) and B.1.1.7 (17,512/56,453).
Blinding	Only objective parameters were included in the study design. Blinding was not applied as all available patient material/data were used and therefore blinding did not affect the experiments and analyses. Non-objective parameters were not included in the study design.

Reporting for specific materials, systems and methods

We require information from authors about some types of materials, experimental systems and methods used in many studies. Here, indicate whether each material, system or method listed is relevant to your study. If you are not sure if a list item applies to your research, read the appropriate section before selecting a response.

Materials & experimental systems

n/a	Involved in the study
☐	☒ Antibodies
☐	☒ Eukaryotic cell lines
☒	☐ Palaeontology and archaeology
☐	☒ Animals and other organisms
☐	☒ Human research participants
☒	☐ Clinical data
☒	☐ Dual use research of concern

Methods

n/a	Involved in the study
☒	☐ ChIP-seq
☒	☐ Flow cytometry
☒	☐ MRI-based neuroimaging

Antibodies

Antibodies used	SARS-CoV-2 N (Rockland #200-401-A50)<br>SARS-CoV-2 S (Rockland #600-401-MS8)<br>SARS-CoV-2 ORF3a (https://mrcppu-covid.bio; Immuno Sequence: GST-SARS-CoV2 ORF3A [DU 67698])<br>AF568-labeled goat-anti-rabbit (Invitrogen, #A11011)<br>AF488-labeled Phalloidin (Hypermol, #8813-01)
Validation	All antibodies were obtained from commercial vendors and specificity characteristics were based on descriptions and information provided in corresponding data sheets available and provided by the manufacturers.<br>For the SARS-CoV-2 N and S-specific antibodies: Bouhaddou et al., Cell. 2020 Aug 6;182(3):685-712.e19. doi: 10.1016/j.cell.2020.06.034. Epub 2020 Jun 28.<br>For ORF3a specific antibody: https://doi.org/10.1371/journal.pbio.3001091

Eukaryotic cell lines

Policy information about cell lines

Cell line source(s)	VeroE6 cells (ATCC CRL-1586), Calu-3 cells (ATCC-HTB-55), BW5147 (doi:10.1016/j.jim.2012.09.006)
Authentication	None of the cell lines were authenticated.
Mycoplasma contamination	all cell lines were tested monthly negative for mycoplasma
Commonly misidentified lines (See ICLAC register)	no commonly misidentified cell lines were used in the study

Animals and other organisms

Policy information about studies involving animals; ARRIVE guidelines recommended for reporting animal research

Laboratory animals	Transgenic (K18-hACE2)2Prlmn mice (Winkler et al., Nat Immunol. 2020 Nov;21(11):1327-1335. doi: 10.1038/s41590-020-0778-2. Epub 2020 Aug 24) were purchased from The Jackson Laboratory and bred locally. Hemizygous 8-12-week-old male animals were used.<br>Mice were housed at 14-hour light/10-hour dark cycles and temperatures of ~18-23°C with 40-60% humidity.
Wild animals	no wild animals were used in the study
Field-collected samples	no field collected samples were used in the study
Ethics oversight	All animal studies were performed in accordance with the guidelines of the Federation for Laboratory Animal Science Associations and the National Animal Welfare Body. All experiments were in compliance with the German animal protection law and approved by the animal welfare committee of the Regierungspraesidium Freiburg (permit G-20/91).

Note that full information on the approval of the study protocol must also be provided in the manuscript.

Human research participants

Policy information about studies involving human research participants

Population characteristics	Blood donors were selected due to their vaccination status (n=14, age range: 26-63, 4 female, 12 male) or their previous COVID-19 history (n=16, age range: 31-79, 6 female, 8 male).
Recruitment	Patients and vaccinees were recruited at the University Hospital Freiburg (in- and outpatient section); self-selection bias or other biases can be excluded since none of the recruited individuals were excluded based on e.g. sex, immunstatus or age.
Ethics oversight	The project has been approved by the ethical committee of the Albert-Ludwigs-Universität, Freiburg, Germany. Written informed consent was obtained from all participants and the study was conducted according to federal guidelines,

local ethics committee regulations (Albert-Ludwigs-Universität, Freiburg, Germany: No. F-2020-09-03-160428 and no. 322/20) and the Declaration of Helsinki (1975).

Note that full information on the approval of the study protocol must also be provided in the manuscript.
